# Supramolecular Complexation of Quenched Rosamines with Cucurbit[7]Uril: Fluorescence Turn-ON Effect for Super-Resolution Imaging

**DOI:** 10.1021/jacs.5c06406

**Published:** 2025-07-29

**Authors:** Dušan Kolarski, Mariano L. Bossi, Richard Lincoln, Juan C. Fuentes-Monteverde, Vladimir N. Belov, Stefan W. Hell

**Affiliations:** † Department of NanoBiophotonics, Max Planck Institute for Multidisciplinary Sciences (MPI-NAT), 37077 Göttingen, Germany; ‡ Department of Optical Nanoscopy, Max Planck Institute for Medical Research (MPI-MR), 69120 Heidelberg, Germany; § Department of NMR-Based Structural Biology, Max Planck Institute for Multidisciplinary Sciences (MPI-NAT), 37077 Göttingen, Germany

## Abstract

Here, we present a fluorogenic supramolecular host–guest system comprising cucurbit[7]­uril (CB7) and a rationally designed rosamine fluorescent dye (**P-ARose**) tailored for super-resolution imaging applications. By strategically designing the *meso*-aryl moiety of the guest, we concomitantly quenched the dye’s emission in its free form and achieved strong binding with the host. The formation of the complex suppresses quenching and encompasses a large fluorescence turn-ON effect. Experimental and theoretical studies revealed that CB7 complexation significantly improved the photophysical properties of **P-ARose**, with a 6.4-fold fluorescence increase and 4.2-fold enhanced emission quantum yield. Further functionalization of **P-ARose** with a HaloTag ligand or an NHS reactive group rendered it suitable for live-cell and immunofluorescence labeling, yielding specificity, negligible background, and a minimal fluorescence signal in the unbound state. The addition of CB7 drastically increased fluorescence, enabling conventional and stimulated emission depletion (STED) imaging with subdiffraction resolution. Furthermore, the turn-ON ability of the host–guest complex facilitated pseudo two-color sequential imaging of different protein combinations within the same acquisition channel. These results demonstrate the potential of this supramolecular system to enable an additional super-resolution imaging multiplexing modality through noncovalent chemistry.

## Graphical abstract

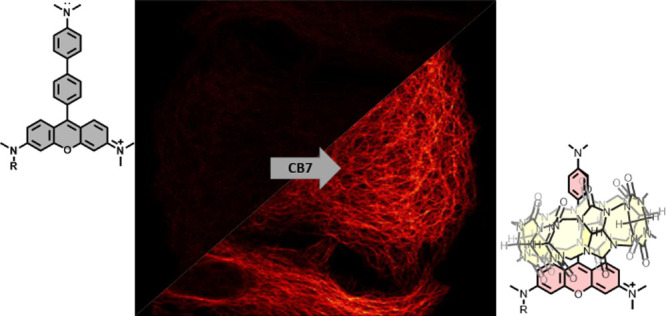

## Introduction

Breaking the diffraction barrier, super-resolution fluorescence microscopy methods strongly impacted the life sciences and discoveries in synthetic materials,^1–4^ allowing for unprecedented insights into cellular and material architectures, molecular interactions, and dynamic processes that were previously unresolvable by optical microscopy.^5–9^ To access the full breadth of these methods, optimized and thoughtfully chosen fluorescent probes are central to success.^10–12^ Challenged by expanding applications in fluorescence microscopy, the palette of small-molecule fluorophores has grown remarkably during the past two decades.^10,13–15^ Nevertheless, despite the rising number of fluorescent dyes, essential challenges in super-resolution microscopy persisted, such as on-demand fluorescence control (turn-ON probes are particularly useful) and multiplexing within a single detection channel.^16–18^ Organic chemistry offers three main approaches for addressing these challenges in fluorescence imaging caused by the scarcity of well-performing probes. Those are (a) preparing new tailor-made fluorescent dyes,^11^ (b) structurally (covalently) combining known moieties to sum up desired properties,^19^ and (c) tuning photophysical properties using noncovalent interactions.^20^ The design of new fluorophores according to approaches “a” and “b” requires critical analysis of the literature data and application methods, as well as detailed investigation of photophysical and photochemical properties. On the other hand, the use of known dyes and modulating their photophysical properties with an external stimulus (approach “c”), in particular by means of supramolecular chemistry, can offer a complementary approach.^21^

Since the discoveries of Lehn, Cram, and Pedersen,^22^ the concept of supramolecular chemistry has transformed a wide range of chemistries.^23–25^ The construction of self-assembly systems based on noncovalent interactions is beneficial as it avoids multistep synthesis, the process is reversible, and it can be externally controlled.^26^ Interestingly, while the host–guest approach has a long tradition of enhancing bioimaging,^27–31^ it has only recently emerged as a powerful super-resolution technique.^20,21,32–36^ The most common macrocyclic hosts for encapsulating fluorescent dyes are cyclodextrins (CD), calix­[n]­arenes (CX), and cucurbit­[n]­urils (CB), which, upon binding, can enhance photophysical properties of a dye and enable new applications in sensing, imaging, and materials science.^37–40^ In this work, we have focused on cucurbit[7]­uril (CB7) host due to its high water solubility (5–30 mM),^21,41^ suitable cavity size with exceedingly higher binding affinities (10^4^–10^15^ M^–1^), which is higher than that of other macrocycles (<10^4^ M^–1^),^38^ and its biocompatibility.^42,43^ The polar urea-covered portals and the hydrophobic interior cavity of CBs make them ideal hosts for positively charged alkyl amines (interacting with urea residues), or small and spherically symmetrical adamantane or ferrocene moieties (replacing water from the cavity).^11,41^ However, common dyes for super-resolution imaging,^10^ such as rhodamines and their carbo- or silicon-analogs, do not contain a suitable high-affinity binding motif. For instance, CB7 binds rhodamine 6G (Rh6G) via the positively charged *N,N*-dialkylamino xanthene moiety^44^ with a relatively good binding constant (5 × 10^4^ M^–1^) and induces a slight bathochromic shift and unaltered fluorescence quantum yield (89%). CB7 complexation has been limited to preventing Rh6G adhesion to glass and plastic surfaces. Other example combinations of host molecules with highly emissive dyes are scarce and usually result in quenching—or, for fluorescein-based dyes, feature only a small-to-moderate enhancement of photophysical properties.^21^ As a result, two approaches for making host–guest complexes with xanthene dyes have been established: (i) rhodamine extension with high-affinity host-binding linkers^21,41^ or (ii) the use of small, xanthene-like dyes that fit the host cavity (e.g., oxazines).^35^ In both cases, the distance of the host-binding domain from the dye as well as the use of already highly fluorescent dyes in their unbound state prevents their use as turn-ON systems (switching from a state in which the dye cannot be used for imaging to a strongly emissive state suitable for microscopy).

Therefore, we sought to design a xanthene-based dye that shows a turn-ON effect upon binding to an external host (CB7), enabling the application of the whole assembly in super-resolution microscopy (STED) and allowing for imaging two different protein targets within the same color channel (detection window).

## Results and Discussion

The guest dyes were based on rosamines, a class of xanthene fluorophores closely related to rhodamines, with similar photophysical properties such as high molar absorptivity (ε_max_), photostability, and quantum yields (Φ_fl_).^45^ Rosamines differentiate themselves by the lack of the carboxylic group in the *ortho*-position of the *meso*-aryl moiety (located at C9 of the xanthene core), making them preferable for applications where the steric hindrance and/or the lactonization equilibrium introduced by the carboxylic group is an issue.^46–50^ The main idea of our approach was bringing the binding domain to the *meso-*aryl position, allowing access to easily prepared fluorescent dyes, modular manipulation of photophysical properties, and improving the binding affinity (Figure 1).

**Figure 1:**
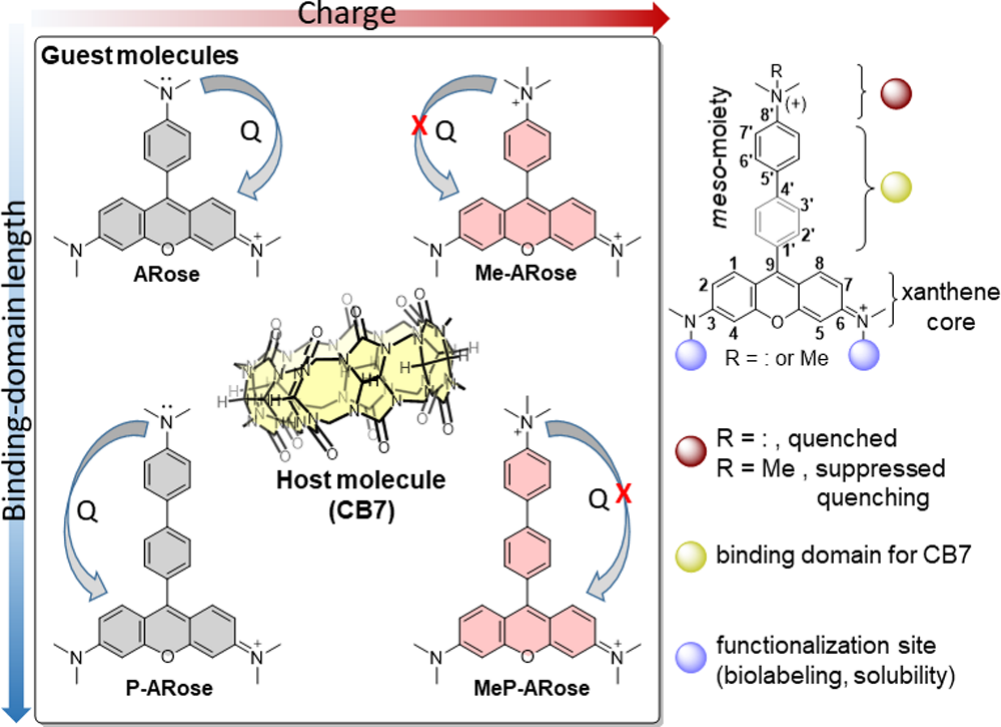
Design of four rosamine-based guests for CB7 binding. The binding domains differ in both length and quaternization of the 8′-*N*,*N*-dimethylamino group introduced as a quencher. Due to quenched fluorescence (Q), **ARose** and **P-ARose** are shown with gray rings, and **Me-ARose** and **MeP-ARose** are shown with pink rings indicating their strong emission. The host (CB7) is presented in yellow.

Furthermore, 4′-dimethyl**A**mino-**Ros**amine (**ARose**)^51,52^ was shown by So̷rensen et al. to act as a bichromophore,^53^ constituted of two nearly isoenergetic xanthylium and arylpyrylium subsystems. This results in weak fluorescence of **ARose**, arising from thermal deactivation of the arylpyrylium excited state and/or a twisted intramolecular charge transfer (TICT) state.^52^ This ‘bichromophore effect’ is abolished upon protonation of the 4′-dimethylamino, i.e., creating a pH-sensor—we reasoned this moiety-dependent fluorogenicity can also be advantageous for the desired fluorogenic response of our system upon complexation.

To ensure CB7 complexation, the 2′-position was left unsubstituted. We also assumed that the xanthene core, more precisely 1/8 and 2/7 C–H (Figure 1), could impose steric hindrance to CB7 binding at the C9-aryl substituent. Thus, two homologues with different binding domain lengths were prepared and named **ARose** and **P-ARose** (‘P’-indicates the additional phenyl ring) (Figure 1). DFT calculations suggested that the introduction of the additional phenyl bridge would favor quenching of the locally excited xanthene, by means of the bichromophore effect, similar to that reported for **ARose** (Figure S1A,B).^53^ Lastly, corresponding quaternized ammonium salts were prepared (**Me-ARose** and **MeP-ARose**) as model compounds to investigate the influence of CB7 interaction with positively charged analogs possessing a permanently suppressed quenching process.

The model guest compounds were prepared through a facile TFA-catalyzed condensation of 3-(*N*,*N*-dimethylamino)­phenol (**1**) and benzaldehydes **2a**–**b**, followed by chloranil oxidation of the intermediate leuco-compounds. The corresponding methylated guests were obtained by microwave-assisted alkylation with methyl iodide. To attach the carboxy handle for labeling, desymmetrization of **P-Arose** was conducted by cross-condensation that involved **2b** and two different aminophenols (**1** and **3**). All three condensation products (**4–6**) were isolated, and after hydrolysis, compounds **5** and **6** were converted to *N*-hydroxysuccinimide (NHS) esters (**7–9**). Under basic conditions, NHS esters reacted with the [O_2_]­HaloTag ligand for live-cell labeling. Compound **7** was also used for conjugation with antibodies, due to the relatively high hydrolytic instability of esters **8** and **9**.

With these compounds in hand, the photophysical properties of the free dyes and their supramolecular complexes with CB7 were investigated (Figure 2A–D, Table 1). The absorption maxima (λ_max_^abs^) of all dyes were within the green region (545–558 nm), with Stokes’ shifts of 26–32 nm. The extinction coefficients (ε) were somewhat lower for nonalkylated dyes, but increased upon quaternization of the amino group (Table 1). As anticipated, the emission of **ARose** and **P-ARose** was weak (emission quantum yields of 1.2 and 7.3%, respectively), while their methylated analogs **Me-ARose** and **MeP-ARose** exhibited fluorescence quantum yields of 30 and 29%, respectively. This observation suggested emission quenching of **P-ARose** via intramolecular bichromophore effect, albeit weaker than for **ARose** — potentially due to the larger distance between 8′-*N*,*N*-dimethylamine and the xanthene core or a higher steric barrier to excited-state planarization. DFT/TD-DFT calculations of the potential energy surface of **P-ARose** resulted in a similar profile to **ARose** (as reported by So̷rensen et al.),^53^ lending credence to this quenching pathway (Figure S2). We note a lower energy of S1, consistent with the extended conjugation of the arylpyrylium subsystem, and a greater S1–S2 gap that could be responsible for the less-efficient quenching of **P-Arose**.

**Figure 2:**
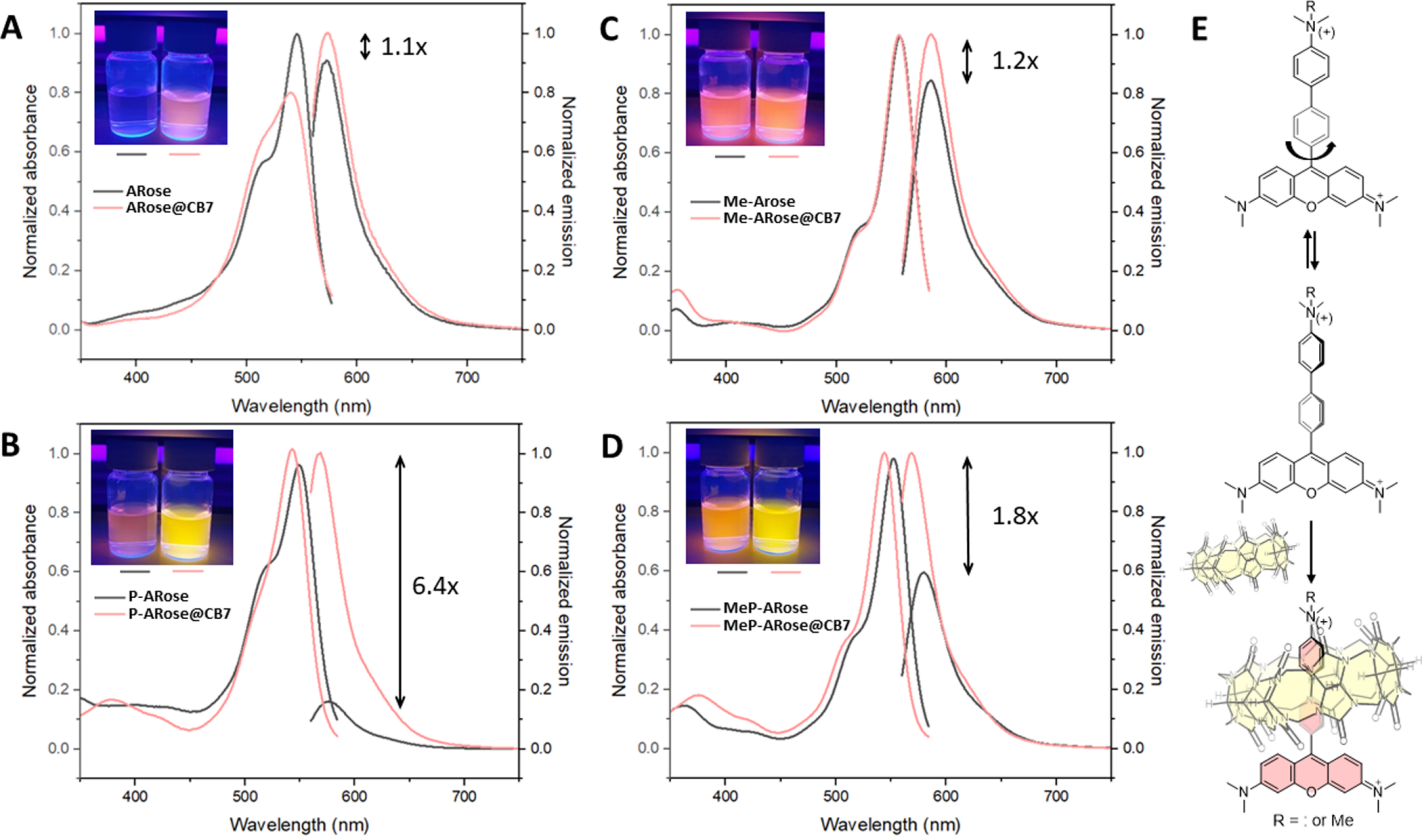
Absorption and emission spectra of **ARose** (A), **P-ARose** (B), **Me-ARose** (C), and **MeP-ARose** (D) (black) as well as their corresponding complexes with CB7 (pink). Insets show images of aqueous solutions under irradiation with UV light before (left vial) and after CB7 addition (right vial). All measurements, including images, were performed with 10 μM dye and 100 μM CB7 concentration in water. The excitation was conducted at the λ_max_^abs^. (E) Proposed binding geometry of the host–guest complex.

**Table 1:** Absorption Maxima (λ_max_^abs^), Extinction Coefficients (ε_max_), Emission Maxima (λ_max_^em^), Fluorescent Quantum Yields (Φ_fl_), Ratio of Fluorescent Quantum Yields before and after CB7 Binding, Fluorescent Lifetimes (τ_fl_), Radiative (*k*_r_) and Non-radiative Rate (*k*_nr_) Constants in Water, and Association Constant (Ka)

	λ_max_^abs^ [nm]	ε_max_ [10^3^ M^–1^cm^–1^]	λ_max_^em^ [nm]	Φ_fl_ ^a^ [%]	Φ_fl_ ratio^b^	τ_fl_ ^c^ [ns]	*k*_r_^d^ [10^8^s^–1^]	*k*_nr_^d^ [10^8^s^–1^]	*K*_a_ [10^5^ M^–1^]
**ARose**	545	64.6	577	1.2	1.6	1.77	0.07	5.58	ND
**ARose@CB7**	541	52.0	579	1.9		1.75	0.11	5.61	
**Me-ARose**	558	82.0	585	30	1.3	1.72	1.74	4.07	
**Me-ARose@CB7**	557	82.7	586	39		2.24	1.74	2.72	6.2
**P-ARose**	550	48.0	576	7.3	4.2	1.48	0.49	6.26	
**P-ARose@CB7**	543	50.8	568	31		1.69	1.83	4.08	8.0
**MeP-ARose**	553	78.4	579	29	1.3	1.55	1.87	4.58	
**MeP-ARose@CB7**	545	80.1	569	38		2.20	1.73	2.82	45.5

a The fluorescence quantum yields (absolute values) were measured using an integrating sphere.

b The ratio is given as Φ_fl_^@CB7^: Φ_fl_^dye^.

c The fluorescence lifetimes of **ARose** and **Me-ARose** were obtained using monoexponential decay, while for **P-ARose** and **MeP-ARose**, a 2nd-order fitting was applied, for which amplitude average lifetimes are given.

d The radiative and nonradiative rate constants were calculated by *k*_r_ = Φ_fl_/τ_fl_ and τ_fl_.

As amine functional groups can also contribute to fluorescent quenching through photoinduced-electron transfer (PeT) or twisted intramolecular charge transfer (TICT) states,^50–56^ we cannot rule out the role these nonradiative pathways may play in the quenching of **P-ARose**. An initial solvent screening (Figure S3) did not reveal significant differences in fluorescence quantum yield as a function of solvent polarity, which discredits PeT, but photobleaching studies (vide infra) suggest TICT. Additional low-temperature (e.g., anisotropy) or ultrafast characterization is required to elucidate the definite quenching pathway(s) of **P-ARose**, which remain beyond our means.

Nevertheless, as the (partial) fluorophore quenching was abolished by quaternization of the amino group, we reasoned that quenching, and in turn fluorogenicity, would be sensitive to pH.^57^ We measured the absorption and emission properties of **ARose** and **P-ARose** over a broad pH range (2–11). It was observed that the fluorescence of **Arose** and **P-ARose** was indeed strongly dependent on pH, whereas the photophysical properties of **Me-ARose** and **MeP-ARose** were almost unchanged (Figures S4–S21). Quenching suppression upon blocking the electron donor by protonation or methylation supports the idea of 8′-*N*,*N*-dimethylamine being fundamental for quenching.^50,57,58^

Next, we investigated the binding influence of CB7 on the photophysical properties of the rosamines. Initially, all compounds were titrated with the host macrocycle to determine the concentration needed for a complete binding (Figures S22 and S23). Owing to the high binding affinity of **P-ARose** and **MeP-ARose** (vide infra), only one equivalent of CB7 was needed to achieve full complexation, whereas **ARose** and **Me-Arose** needed up to five equivalents. For this reason, we used 10 equiv of the host to ensure the conditions are identical and comparable (Figure 2). In the presence of CB7, **ARose** exhibited a noticeable hypsochromic shift, and the band broadening was encompassed by a decrease in the extinction coefficient by 20% (Figure 2A and Table 1). The emission intensity rose by 10%, and the fluorescence quantum yield displayed a marginal increase, indicating an unproductive host–guest interaction (no turn-ON effect). On the other hand, **P-ARose@CB7** exhibited a large increase in fluorescence intensity and quantum yield (6.4- and 4.2-fold, respectively), while the absorption spectrum displayed a minor hypsochromic shift and ε_max_ increase (Figure 2B and Table 1). The large difference in responsiveness between **ARose** and **P-ARose** can be attributed to an extended binding domain of **P-ARose**, which can accommodate the CB7 host. This difference suggests that the host probably binds to the *meso*-aryl moiety of **P-ARose** rather than the xanthene fragment having two *N*,*N*-dimethylamino groups. It is worth noting that **P-ARose@CB7** has a quantum yield comparable to that of **MeP-ARose**, indicating that a reversible noncovalent supramolecular complexation can result in the same effect as an irreversible covalent transformation. Lastly, the emission intensities and fluorescence quantum yield of **Me-ARose@CB7** and **MeP-ARose@CB7** moderately increased (1.2- and 1.8-fold, and 1.6-fold in both cases) accompanied by a slight blue shift of their spectra (Figure 2C,D and Table 1). The values of fluorescence lifetimes also revealed that **ARose** and **P-ARose**, as well as their CB7-complexes, obey a double-exponential decay, with a shorter-lived component becoming less abundant upon complexation (Figures S24–S27 and Tables S1–S4). On the other hand, the fluorescence decays of **Me-ARose**, **MeP-ARose**, **Me-ARose@CB7**, and **MeP-ARose@CB7** were monoexponential (Figures S28–S31), implying that the short-lived components were suppressed by methylation. **ARose** was the only rosamine that exhibited no change in lifetime or radiative (*k*_r_), as well as nonradiative rate constants (*k*_nr_), upon CB7 addition. At the same time, the complexation of all other dyes showed a characteristic increase in lifetime and *k*_r_ (Table 1).^59,60^ Overall, these findings show the potential of using **P-ARose** dye for a CB7-induced turn-ON probe in fluorescent imaging.

As the **P-ARose** dye showed a strong turn-ON effect caused by the addition of CB7, we selected it for further characterizations (Figure 3). It was previously shown that guest–host complexation can significantly alter the guest’s p*K*_a_ value due to the change in the local environment and the noncovalent interactions between CB7 and binding motifs.^37,61,62^ To clarify if the fluorescence turn-ON effect from CB7 binding was merely due to protonation, we measured p*K*_a_ values of the most basic 8′-*N*,*N*-dimethylamino group in the absence or presence of CB7 excess, i.e., for the free and the bound fluorophore (Figure 3A). Consistent with reports on the CB7 binding effect, p*K*_a_ did slightly shift to more basic values in the supramolecular complex, but the data in Figure 3A indicate that the amino group is still completely deprotonated at a biologically relevant pH (ca. 7.4), indicating that protonation cannot account for the observed fluorogenic response.

**Figure 3:**
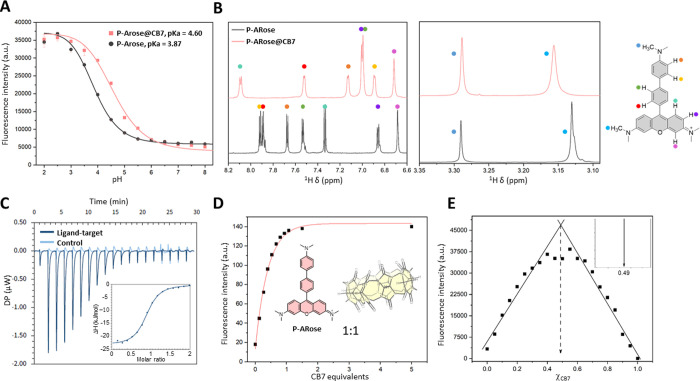
(A) Influence of CB7 addition on p*K*_a_ (black line -5 μM **P-ARose**, pink line -5 μM **P-ARose** and 500 μM CB7). (B) ^1^H NMR analysis of chemical shifts upon host–guest interaction (saturated D_2_O solutions of **P-ARose** and **P-ARose@CB7**). Stoichiometry of **P-ARose** interaction with CB7 monitored by (C) isothermal titration calorimetry (40 μM **P-ARose**), (D) fluorescence spectroscopy (5 μM **P-ARose**), as well as calculated from the (E) Job’s plot (the inset indicates the plot maximum at 0.49).

Furthermore, ^1^H NMR analysis of the **ARose@CB7** and **P-ARose@CB7** complexes (Figures 3B and S38–S90) revealed differences in CB7 binding to the corresponding dye. Host complexation with **ARose** induced significant change of the chemical shifts of all aromatic and methyl protons, indicating nondistinguishable binding to the xanthene and 9-aryl moiety (Figure S63). We presume that the 9-aryl substituent is too short to bind CB7 strongly, and therefore, the host also transiently and weakly binds the xanthene part. In contrast, pronounced chemical shift changes were detected for the aryl protons located on the *meso*-aryl unit of **P-ARose@CB7** (ΔδH2′ = 373 ppb; ΔδH3′ = 530 ppb; ΔδH6′ = 921 ppb; ΔδH7′ = 544 ppb), strongly indicating that this region of the molecule engages in host–guest interactions with the internal cavity of CB7. This assignment was corroborated by selective ROESY experiments (Figure S81), which showed clear cross-relaxation contacts between the inner cavity proton of CB7 (CB7-Ha at 5.6877 ppm) and the dimethylamino groups at positions 3 and 8′, as well as with protons H2′, H3′, H6′, and H7′ of the *meso*-aryl ring, but not with H4 or H5. This ROE pattern is consistent with an inclusion geometry in which the *meso*-aryl unit is embedded within the CB7 cavity, while the xanthene core remains external. Additionally, the 1,8-xanthene protons displayed a substantial chemical shift change (ΔδH1/8 = 755 ppb) and the 2,7-protons a moderate shift (ΔδH2/7 = 141 ppb), likely due to proximity to the CB7 portals and interaction with the host urea rims. In contrast, the H4 and H5 signals exhibited minimal variation (Δδ ≈ 31 ppb), indicating that this region is sterically distant from the host. Lastly, negligible chemical shift variation for the 8′-*N,N*-dimethylamino group (Δδ^1^H = 1.4 ppb) suggests that this moiety remains outside the host cavity and does not participate in direct interactions with CB7. Similarly, the *N,N*-dimethylamino substituents on the xanthene core displayed only minor changes (Δδ^1^H = 26 ppb), further supporting the conclusion that protonation is not responsible for the fluorescence turn-ON effect of **P-Arose**. Protonation of the 8′-dimethylamino group would be expected to cause a significant downfield shift, which was not observed. These data collectively define the *meso*-aryl ring as the primary binding site of CB7. Also, these findings are consistent with DFT calculations of the preferred binding orientation of CB7 to **P-ARose**, which favored binding to the biphenyl cleft, exposing the dimethylamino moiety (Figure S1C) and a decrease in planarity between the xanthene and biphenyl rings.

Next, we performed isothermal titration calorimetry (ITC) as well as spectrophotometric titration to determine the binding stoichiometry (Figures 3C,D, S22, S23, and S32–S36). Both titrations yielded a 1:1 binding ratio, and ITC revealed the dissociation constant of **P-ARose@CB7** to be 1.25 μM (*K*_a_ = 8 × 10^5^ M^–1^) as a sign of a very strong binding (Table 1). The ITC complexation study of the other dyes was also in agreement with our previous observations. While the NMR binding study of CB7 and **ARose** showed the host–guest complex formation (Figure S63), the spectrophotometric and ITC titrations clearly indicate a very weak association constant (Figures S23 and S33). On the contrary, due to a positive charge and sufficient binding-domain length, the *K*_a_ = 4.6 × 10^6^ M^–1^ for **MeP-ARose@CB7** was somewhat higher than for **P-ARose@CB7**. Interestingly, despite a positive charge, the binding of CB7 to **Me-ARose** was similar to that of a charge-neutral binding domain of **P-ARose** (Table 1). Lastly, Job’s plot yielded only one binding site on **P-ARose** (Figure 3E), in agreement with ITC results.

Taken together, our observations led us to propose a plausible mechanism for the binding-induced spectral changes of **P-ARose**. The absence of a 2′-substituent in the binding domain permits some flexibility of the *meso*-aryl group — DFT calculations predict that the optimized geometry of the ground-state is twisted between the xanthene and aryl moieties (Figure S1A,B). Upon binding to the CB7 host, the aryl moieties adopt a flat conformation (with respect to the xanthene plane) to minimize steric interactions in the complex (Figure S1C). This geometry change leads to a binding-induced hypsochromic shift of the xanthene absorption due to an increase in the orbital overlap between the two aromatic parts (i.e., electron donation from the aniline destabilizes the xanthene LUMO). CB7 binding in the biphenyl cleft ultimately disfavors the excited-state reorganization that leads to fluorescence quenching by the arylpyrylium excited state, which requires orthogonality between the xanthene and 8′-*N*,*N*-dimethylamino moiety (Figure S2, red curve) that would ultimately lead to a TICT intermediate.^52^

This idea is further supported by the main products observed after photolysis of **P-Arose** in water (Figures S91 and S92), revealing an oxidative photodealkylation of the 8′-*N,N*-dimethylamino moiety as the main decomposition path (Figure S92), occurring from an arylpyrylium-centered TICT state.^63^ In contrast, this path is considerably hindered when the chromophore is in the macromolecular complex (**P-ARose@CB7**), leading to a remarkable increase in its photostability (Figure S91).

With **P-Arose** and its fluorogenic interaction with CB7 established, we set to apply our system in fluorescence microscopy. We started by preparing compounds **10–12**, containing the HaloTag-specific chloroalkane ligand (Scheme 1). Following a detailed binding analysis, the ligand was purposely incorporated as one of the 3,6-di-(*N*,*N*-dialkylamino)-xanthene substituents to minimize interference with the CB7 interaction and the *meso*-aryl moiety. Apart from the desired compound **10**, the synthesis also yielded double substituted side products that were further modified and applied for cell labeling (compounds **11**-**12**). While **10** and **12** have a positive net charge, **11** bears a carboxylic group, leading to an overall neutral compound that can improve cell permeability.

**Scheme 1:**
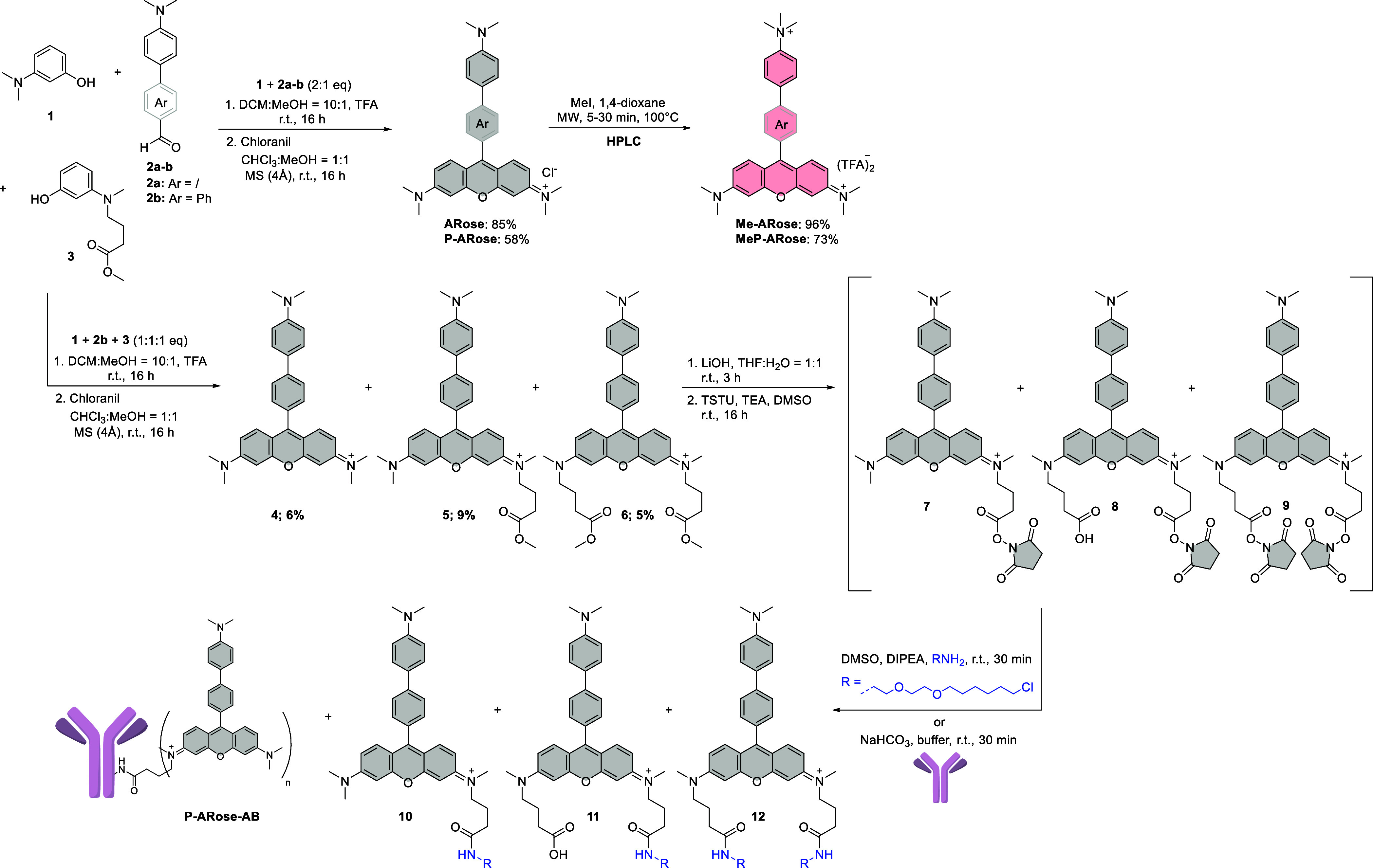
Synthesis of the Model Guest Dyes (**ARose**, **P-ARose**, **Me-ARose**, and **MeP-ARose**) and the Modified Analogues of **P-ARose** for Cell-Labeling (10-12 and **P-ARose-AB**)

To test the efficiency of our chloroalkanes for biological labeling, we initially screened all three probes in fixed samples. The cellular target used in these experiments was a vimentin-HaloTag fusion protein stably expressed in U2OS cells.^64,65^ The cells were labeled for 1 h, washed, and then imaged in water as mounting medium. All three probes showed a weak but specific signal consistent with that of vimentin filaments. However, upon supramolecular complexation with CB7, we successfully observed a large signal enhancement. Labeling with compound **11** yielded a much dimmer background (Figure 4A,B) than compounds **10** or **12**, probably due to the higher lipophilic character of these dyes that may increase aggregation or nonspecific binding to apolar structures (e.g., cell membranes) (Figures S93 and S94). The addition of the carboxy-butyl chain in compound **11** did not impede CB7 binding but appeared to reduce aggregation and increase solubility. Thus, we proceeded with compound **11** for labeling in live cells.

**Figure 4:**
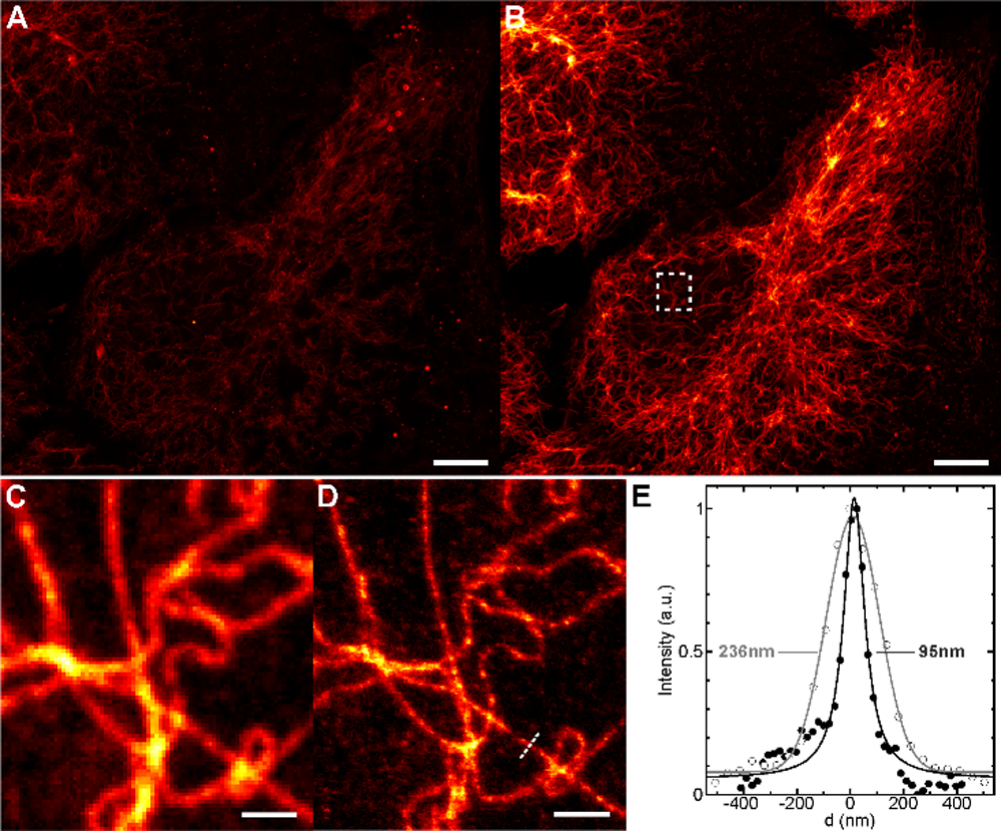
Emission turn-ON of compound **11** upon supramolecular complexation with CB7 on fixed U2OS-VimHalo cells. Samples were fixed, permeabilized, and then labeled with compound **11** (1 μM/1h) in PBS/BSA (2%). After washing, the sample was mounted and imaged in water (A) and immediately after the addition of CB7 (B) to a final concentration of 1.5 mM. Confocal (C) and STED (660 nm STED laser) (E) images of the ROI indicated in B, acquired in the presence of CB7. (E) Profiles along the line indicated in D, with the fwhm’s obtained from Gaussian (confocal, empty symbols) and Lorentzian (STED, filled symbols) fit. Scale bars: 10 μm (A, B) and 1 μm (C, D).

To this end, cells were incubated for 1 h with compound **11** incorporated in the cell medium, washed with fresh medium, and then fixed by a standard method. Imaging before and after complexation was performed the same way as indicated before, revealing that the labeling of live cells was also successful with a slightly reduced signal-to-noise ratio (Figure S95). It is worth noting that the overall charge-neutral **11** did not exhibit unspecific labeling, and it was not accumulated in mitochondria, as is usually the case with positively charged dyes suited for CB7 interaction.^20^ Lastly, these HaloTag-modified **P-ARose** dyes were found to be compatible with a 660 nm STED laser, allowing for the observation of the individual filaments with subdiffraction resolution (Figures 4C–E and S95C).

Next, we tested our dyes for application in immunofluorescence labeling, preparing a secondary antibody **P-ARose-AB** (Scheme 1), tagged with NHS ester **7** to a degree of labeling (DOL) of 3–4. We applied **P-ARose-AB** for the indirect immunofluorescence labeling of two different structures in fixed U2OS cells. First, the translocase of the outer membrane complex of mitochondria TOM20 (Figure 5) and, second, vimentin intermediate filaments were used (Figure S96). The initial signal was very low due to efficient quenching of the dye on the antibody. After the addition of CB7, clear structures appeared, as the brightness increased significantly upon supramolecular complexation (Figures 5B and S96B). Again, the application of the 660 nm STED laser was successful and produced high-quality super-resolution STED images (Figure 5D), with a clear resolution enhancement with respect to its conventional confocal counterpart (Figure 5C). Quantification of the signal enhancement on immunostained samples upon the host binding (Figure 6) yielded a signal ratio of 4.5–6-fold, correlating well with the enhancement observed in cuvette experiments with the model compound **P-ARose** (Figure 2B). Thus, we did not observe a substantial effect of the conjugated protein on the fluorogenic behavior of **P-ARose**.

**Figure 5:**
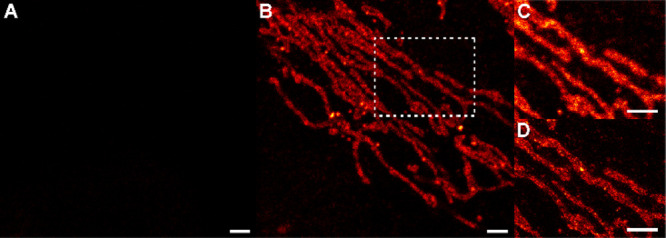
Emission turn-ON of **P-ARose-AB** upon supramolecular complexation with CB7. Fixed U2OS cells were immunostained with primary antibody against TOM20, and a secondary antibody labeled with **P-ARose**-modified NHS ester (compound **7**). The sample was mounted and imaged in water (A) and then imaged again immediately after the addition of CB7 to a final concentration of 1.5 mM (B). Confocal (C) and STED (660 nm STED laser) (D) images of the ROI indicated in part B, acquired in the presence of CB7. Scale bars: 5 μm.

**Figure 6:**
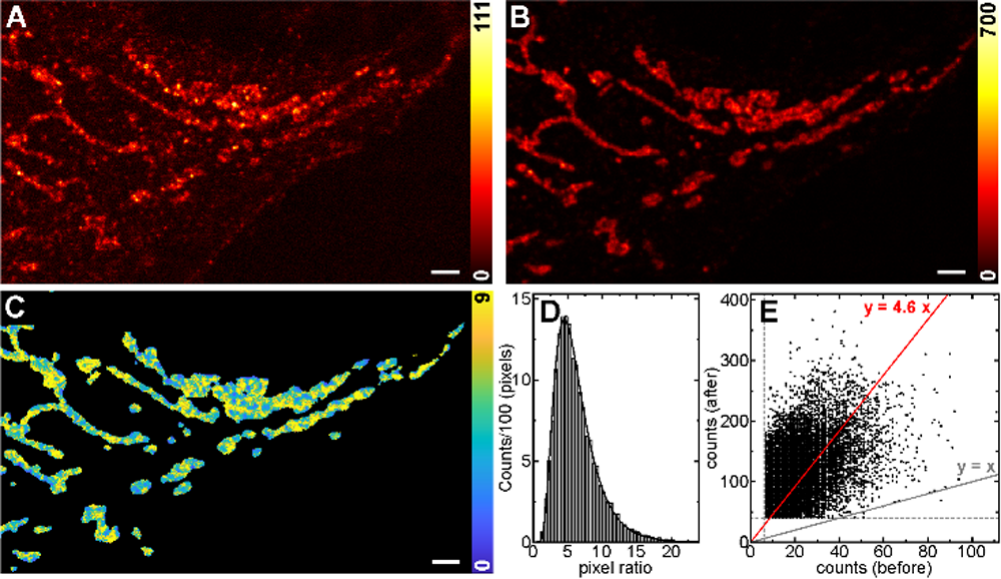
Emission turn-ON effect of **P-ARose-AB** upon supramolecular complexation with CB7 on fixed U2OS cells, labeled with primary antibody against TOM20, and a secondary antibody labeled with **P-ARose**-modified NHS ester. The sample was mounted and imaged in water (A) and then imaged again immediately after the addition of CB7 to a final concentration of 1.5 mM (B). Note the different scales for the signal level used for each image (110 in A, and 700 in B). An overview of the image presented with the same scale in given in Figure S97. (C) Map of the signal ratios obtained from the images in parts A and B. (D) Distribution of the ratios obtained in C (zero values were eliminated) with a fit to a log-normal distribution (*x*_c_ = 5.6). (E) Signal correlation between the two images in A and B with a linear fit, indicating a 4.6 average signal increase. Scale bars: 2 μm.

Lastly, the ability to activate the dye by the supramolecular suppression of quenching was demonstrated for single-channel multiplexing. For this purpose, we chose a combination of proteins and performed their indirect immunofluorescence labeling with a combination of **P-ARose-AB** and a nanobody tagged with Cy3B (Cy3B-NB), an established bright and photostable fluorophore with spectral properties similar to our dyes. Due to the ability of **P-ARose** to stay fluorescent-silent before CB7 activation, we designed the imaging sequence where Cy3B was imaged first (protein of interest 1, POI1), then bleached, and finally, **P-ARose** was activated by supramolecular complexation and imaged (protein of interest 2, POI2) (Figure 7). In the first experiment, fixed U2OS cells were labeled with primary antibodies against vimentin and TIM23 (inner mitochondrial membrane protein), followed by a secondary antibody labeled with **P-ARose-AB** and Cy3B-NB. After TIM23 (POI1) was imaged, Cy3B was photobleached (Figure 7A,B) with the 560 nm laser at the maximum power for 10 image scan repetition, preparing the sample for **P-ARose-AB** activation. Next, CB7 addition induced signal enhancement of the labeled vimentin (POI2), allowing for the second imaging sequence within the same excitation and detection spectral window (Figure 7C). This single channel imaging process yielded a pseudo-two-color image (Figure 7D). The same procedure was successfully applied in two more protein combinations—vimentin (**P-ARose-AB**)/NUP153 (Cy3B-NB) and TOM20 (**P-ARose-AB**)/NUP153 (Cy3B-NB) (Figures S98 and S99). All three examples demonstrate the power of supramolecular chemistry to turn on probes for multiplexing within a single acquisition channel.

**Figure 7:**
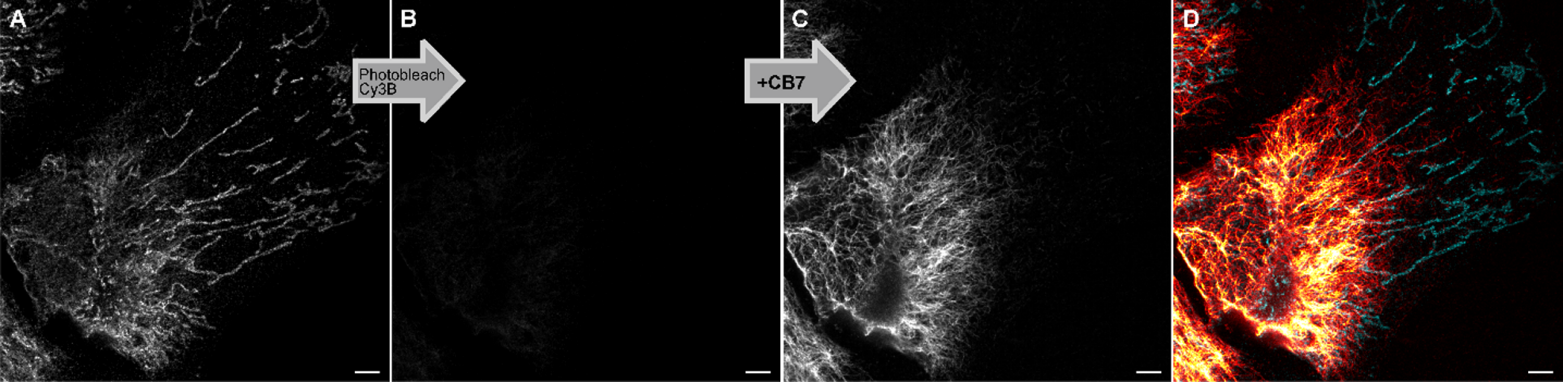
Sequential imaging of Cy3B (A) followed by photobleaching (B) and then imaging of **P-ARose-AB** (C) after its signal was turned-ON by supramolecular complexation with CB7 (1.5 mM). The pseudo two-color image acquired by this single channel process, rendered with distinct colormaps, is shown in D (combined images A and C). U2OS cells were fixed and labeled with two primary antibodies against TIM23 and Vimentin, and a secondary nanobody labeled with Cy3B was combined with a secondary antibody labeled with **P-ARose**. Scale bars: 5 μm.

## Conclusions

In summary, we have developed a supramolecular host–guest system based on CB7 and rosamine fluorescent dye that can be applied as a turn-ON responsive marker in super-resolution imaging. Through a judicious design of the rosamine dye, we successfully achieved a strong CB7 binding (*K*_binding_ = 8 × 10^5^ M^–1^) and relocated the binding domain from the xanthene to the *meso*-aryl moiety. Our approach to regulate emission with an external noncovalent stimulus was based on previously described emissive decay deactivation due to the orthogonal orientation of the *meso*-aryl moiety with the xanthene core in the excited state of a free dye.^53^ We suppressed this quenching pathway using supramolecular interactions with CB7, changing the environment of the binding moiety and inducing its planarization.

A detailed complexation study involving prepared dyes and CB7 revealed that **P-ARose** is a dye with the strongest binding and a positive influence of CB7 on its photophysical properties. We observed a 6.4-fold fluorescence increase and 4.2-fold enhancement in emission quantum yield. The spectral properties of this dye matched the 660 nm STED laser, so **P-ARose** was decorated with a handle which was further converted to NHS ester and HaloTag ligand (compounds **10**–**12**). NHS ester was used for tagging secondary antibodies for indirect immunofluorescence labeling (**P-ARose-AB**). Compound **11** successfully labeled vimentin-HaloTag fusion protein in live and fixed U2OS cells with minimal initial fluorescence and clean background. Upon cell fixation, the addition of CB7 strongly increased fluorescence, allowing for STED imaging with optical resolution below the diffraction limit. Immunolabeling of TOM20 and vimentin was conducted with **P-ARose-AB**, and up to a 6-fold signal enhancement upon CB7 complexation was achieved. Ultimately, the turn-ON ability upon formation of the host–guest complex was demonstrated for imaging of two different targets within the same detection channel. This pseudo-two-color imaging was conducted using three different protein combinations. It was achieved by labeling the proteins of interest (POIs) with Cy3B-NB and **P-ARose-AB**, and performing their sequential imaging. After the first POI labeled with Cy3B was imaged, this dye was bleached, allowing for CB7 activation and imaging of **P-ARose** on the second POI.

To the best of our knowledge, this is a unique system bearing the structure of common xanthene dyes suitable for STED microscopy with new features, including a turn-ON effect solely based on CB7 binding and a charge-neutral and lactonization-free host-binding moiety exhibiting a strong complexation with the CB7 host. For high-affinity CB7 complexation, our system does not require additional bulky moieties (e.g., positively charged amines, ferrocene, and adamantane),^11,41^ neither does it require other external additives to regulate the photophysical properties of a dye.^66,67^ The fluorescent dyes are the smallest “photon sources” for optical imaging (smaller than fluorescent proteins and nanoparticles), offering the most precise structural information in optical nanoscopy methods that today reach localization precision down to the angstrom scale.^5^ Thus, keeping the advantages of organic fluorophores, tuning and enhancing their photophysical properties using supramolecular chemistry, and avoiding any covalent modifications that would influence their size offer valid strategies for nanoscopy to reach its full physical potential. Overall, all these experiments emphasize the strength of the present host–guest system, exploiting the chosen dye design combined with power of noncovalent supramolecular chemistry applied to super-resolution microscopy for expanded imaging possibilities.

## Supplementary Material



## Notes

Experimental section including synthesis and characterization data of all new compounds, copies of spectra, photophysical characterizations, titration experiments, lifetime decay profiles, ITC raw data, Job plots, and microscopic images (PDF)
