# Supplementary material for: Supramolecular Complexation of Quenched Rosamines with Cucurbit[7]Uril: Fluorescence Turn-ON Effect for Super-Resolution Imaging
Source: J Am Chem Soc. 2025 Jul 29;147(32):28893–902. doi: 10.1021/jacs.5c06406 (PMC12356589; doi:10.1021/jacs.5c06406)
Supplement: Supplementary file 1 [file ja5c06406_si_001.pdf]

Supramolecular Complexation of Quenched Rosamines with Cucurbit[7]Uril: Fluorescence Turn-ON Effect for Super-Resolution Imaging

Dušan Kolarski,[1]* Mariano L. Bossi,[2]* Richard Lincoln,[2] Juan C. Fuentes-Monteverde,[3] Vladimir N. Belov,[1] Stefan W. Hell[1]*

[1] Department of NanoBiophotonics, Max Planck Institute for Multidisciplinary Sciences (MPI-NAT), 37077 Göttingen, Germany

[2] Department of Optical Nanoscopy, Max Planck Institute for Medical Research (MPI-MR), 69120 Heidelberg, Germany

[3] Department of NMR-Based Structural Biology, Max Planck Institute for Multidisciplinary Sciences (MPI-NAT), 37077 Göttingen, Germany

Table of Contents

General procedures and methods 4
Synthetic procedures 7
ARose 7
P-ARose 7
Me-ARose 8
MeP-ARose 9
Compounds 4-6 9
Compound 10 11
Compound 11 12
Compound 12 13
DFT/TD-DFT calculation of P-ARose 14
Solvatochromism 16
pH influence on photophysical properties 17
Change of the absorption spectra with pH 18
The pH-dependent absorption at 550 nm 19
pKa determination: Fluorescence intensity across the pH range 20
Spectrophotometric titration 36
Fluorescence lifetimes 38
ARose 38
ARose@CB7 39
P-ARose 40
P-ARose@CB7 41
Me-ARose 42
Me-ARose@CB7 43
MeP-ARose 44
MeP-ARose@CB7 45
Binding study: ITC 46
Instrument control titration 46
ITC analysis 47
ARose 47
Me-ARose 48
MeP-ARose 49
P-ARose 50

Solubility test 51
NMR analysis in aqueous solution 52
ARose 53
ARose@CB7 59
P-ARose 66
P-ARose@CB7 71
Photobleaching of P-ARose 81
Imaging 83
NMR spectra 90
Spectra 90
ARose 90
P-ARose 92
Me-ARose 94
MeP-ARose 97
Compound 5 100
Compound 6 106
Compound 10 112
Compound 11 115
Compound 12 118
References 121

General procedures and methods

General remarks for synthesis: Unless stated otherwise, chemicals and solvents were purchased from suppliers and used as received. Cucurbit[7]uril (CB7) was purchased from Sigma-Aldrich or BLD Pharma. According to the Sigma-Aldrich producer, this material contains residual amounts of salts and acid, and the real content of CB7 is lower than 100%. The actual content of CB7 (M = 1163 Da) was quantified by ^{1}H-NMR spectroscopy. For that, 35.7 mg of CB7 were dissolved in 2.25 mL D_2O (by applying gentle heating and an ultrasound bath; the calculated concentration at saturation c = 13.6 mM), the solution centrifuged, and the small amount of the insoluble residue separated. The concentration was measured to be c^* = 12.0 mM (calibration of the ^{1}H-NMR device with external standard). The supernatant solution was lyophilized, the residue redissolved in water, lyophilized and dried in vacuo (0.02 mbar), until the constant weight was reached. The concentration control by means of ^{1}H-NMR spectroscopy gave results consistent with the gravimetry within ± 7-8%.

All glassware and magnetic stirrer bars were cleaned and dried in an oven before use. The reactions were carried out with magnetic stirring, and evaporations in vacuum were performed in a rotary evaporator with bath temperature not exceeding 45 °C. Analytical RP-HPLC were carried out with a Knauer Azura (Ultimate 3000) systems equipped with a 250 × 4 mm column (Eurospher II, 100-5 C18); flow rate 1.2 mL/min. Solvent A: H_2O + 0.1% v/v TFA, solvent B: MeCN + 0.1% v/v TFA. Preparative RP-HPLC separations were performed on an Interchim puriFlash 4250 device with a 250 × 16 mm column (Eurospher II, 100-5 C18); flow rate 20 mL/min, gradient of acetonitrile in 0.1% aq. TFA.

Nuclear magnetic resonance (NMR): NMR Spectra were recorded on an Agilent 400MR DD2 (400 MHz for ^{1}H), Bruker Advance Neo 500 (500 MHz) or Bruker Neo 800 (800 MHz for binding studies in water). All ^{1}H- and ^{13}C- NMR spectra are referenced to the signals of the residual protons and ^{13}C in $CDCl_3$ (^{1}H: 7.26, ^{13}C: 77.16 ppm) and CD_3OD (^{1}H: 3.31, ^{13}C: 49.00 ppm). The FIDs were processed with the software MestReNova. Multiplicities of the signals are described as follows: s = singlet, d = doublet, t = triplet, q = quartet, p = pentet, m = multiplet or overlap. Coupling constants (J) are given in Hz. All compounds purified using HPLC are obtained as TFA salts, and their ^{19}F NMR spectra contain the TFA peak between -77.1 and -77.3 ppm.

High resolution mass spectrometry (HR-MS): Mass spectra with electro-spray ionization (ESI-MS) were recorded on a Varian 500MS spectrometer (Agilent). ESI-HRMS spectra were acquired using a MICROTOF spectrometer (Bruker) equipped with an Apollo ion source and a direct injector with a Cautosampler Agilent RR 1200.

Absorption spectra, emission spectra, fluorescence lifetime, and fluorescence quantum yield: UV-Vis absorption spectra were recorded in a Cary 4000 UV-Vis spectrometer (Agilent Technologies), and emission spectra in a Cary Eclipse Fluorescence Spectrophotometer (Agilent Technologies), in 4 mL quartz cuvettes (model 119F-10-40, Hellma Analytics). Fluorescence lifetimes were measured with a Quantaurus-Tau fluorescence lifetime spectrometer (model C11367-32, Hamamatsu), and with a FluoTime 300 Fluorescence Lifetime Spectrometer (Pico Quant). The absolute values of the fluorescence quantum yields were obtained with a Quantaurus-QY spectrometer (model C11347-12, Hamamatsu). Any extinction coefficient measurement was repeated three times as three independent experiments. All the measurements were performed at room temperature.

Computational Methods: All quantum mechanical calculations were performed using Gaussian 16 Rev. B. 01.[1] All DFT and TD-DFT calculations were conducted with the B3LYP functional in combination with the 6-31g(d) basis set with an applied PCM model solvation of water unless otherwise noted. Molecular structures and orbitals were visualized using GaussView 6. Frequency checks were carried out after each geometry optimization to ensure that the minima on the potential energy surfaces were found.

Conjugation of antibodies: Amino-reactive NHS-ester was coupled to a goat anti-rabbit (111-005-003, Jackson ImmunoResearch Europe Ltd., LOT 169755, 2.4 mg/mL) secondary antibody using a standard coupling protocol. The amino-reactive dye was dissolved in anhydrous DMSO (5 mM), and mixed with 1 mg antibody in a 30:1 dye/protein ratio. The pH of the solution was adjusted to 8.2 with carbonate buffer (45 µL, 1 M), and stirred for 1 h at room temperature, protected from light. The mixture was purified using a PD MiniTrap G-25 size exclusion column (28918007, GE Healthcare), and stored in the PBS buffer at pH 7.4. The DOL (degree of labelling) was determined by UV-Vis absorption measurements in a "Nano Drop" small volume spectrometer (DS-11+, DeNovix).

Indirect Immunofluorescence Labelling: U2OS cells were grown for 12-72 h on glass coverslips, rinsed with warm (37°C) PBS (pH 7.4), and fixed with 4% PFA at room temperature for 20 minutes. Then, the samples were incubated with quenching solution (0.1 M NH_4Cl and 0.1 M Glycine in PBS) for 5 min, follow by permeabilization with triton 0.1 % for 10 minutes. Then, the samples were treated with blocking buffer (2% BSA in PBS) for 30 minutes, and overlaid with the primary antibody or a mixture of two primary antibodies diluted in blocking buffer and incubated in a humid chamber for 1 h at room temperature. The samples were washed with blocking buffer (3×5 min), and incubated with the secondary antibody **P-ARose-AB** or a mixture with the secondary nanobody **Cy3B-NB** (Nanotag biotechnologies, N1202, contumely labelled with Cy3B) in blocking buffer for 1 h at room temperature. The samples were then washed with blocking buffer (2×5 min), PBS (2×5 min), water (2×5 min) and mounted in an open magnetic chamber (Live Cell Instrument Co. Ltd., cat. # CM-B18-1) filled with 250 µL of water for imaging. For the formation of the host-guest complex, 750 µL of a 2 mM CB7 solution was added *in situ*. The secondary antibodies used were: rabbit Anti-TOM20 [EPR15581-54] monoclonal antibody (Abcam, ab186735), mouse Anti-Tim23, Clone 32/Tim23, monoclonal antibody (BD Biosciences, 611222), rabbit anti-Vimentin [EPR3776] monoclonal antibody (Abcam ab92547), and mouse anti Nup153 monoclonal antibody (Abcam, ab24700).

Halotag Labelling: U2OS-Vim-Halo cells were grown for 12-72 h on glass coverslips. The cells were incubated for 1 h with the corresponding Halo-Tag ligand (compounds **10-12**) diluted in the cell medium at 1 µM concentration (from a 1 mM DMSO stock solution), and washed with fresh medium for 20 minutes. Then, the cells were fixed and permeabilized as described for immunolabeelling experiments. Alternatively, cells were fixed first with the same protocol, and then labelled with the corresponding Halo-Tag compound for 1h at 1 µM concentration in blocking buffer.

Confocal and STED microscopy: Imaging was performed with an Abberior expert line microscope (Abberior Instruments GmbH) built on a motorized inverted microscope IX83 (Olympus). The microscope is equipped with 560 nm excitation pulsed laser, and a 660 nm pulsed STED laser. Spectral detection was performed with avalanche photodiodes (APD) with a 571 – 650 nm window. Images were acquired with a 60x/1.42 Oil immersion objective lens (UPLXAPO60XO, Olympus), and the pinhole set to 1 Airy unit. A z-focus drift compensation unit was used to minimized axial drift during measurement. The pixel size for all images was 70 nm (confocal) and 30 nm (STED/confocal, simultaneous recording on the same ROI, line by line). Laser powers and dwell times were optimized for each sample.

Image analysis: Images were analyzed and rendered with Imspector software (Abberior), or with dedicated routines in MATLAB (version R2021a). Line-profiles were plotted and fitted with Origin (version 2020b). In general, raw images (confocal or STED) are presented without any filter or smoothing. For fluorescence enhancement of the fluorophores upon CB7 binding (Figure 6), images were first registered (if necessary) using an intensity-based image registration process. Then, a pixel threshold was set on both images (registered before and after CB7 addition) to approximately 10% of the pixel with the maximum signal on each image, and the ratio for all remaining pixels was calculated.

List of abbreviations:

AB = antibody
APD = avalanche photodiodes
BSA = bovine serum albumin
CB7 = cucurbit[7]uril,
COSY = homonuclear correlation spectroscopy
Cy3B = cyanine3B
DMSO = dimethyl sulfoxide
HMBC = heteronuclear multiple bond correlation spectroscopy
HSQC = heteronuclear single quantum coherence spectroscopy
NB = nanobody
NHS = *N*-Hhydroxysuccinimide
Nup153 = nucleoporin 153
ITC = Iisothermal titration calorimetry
PBS = phosphate buffered saline
PFA = paraformaldehyde
POI = protein of interest
ROESY = rotating frame overhauser effect spectroscopy
ROI = region of interest
STED = stimulated emission depletion
TFA = trifluoroacetic acid
Tim23 = translocase of the inner mitochondrial membrane 23 complex
TOM20 = Translocase of the Outer Mitochondrial membrane 20
THF = tetrahydrofuran
TLC = thin-layer chromatography
TOCSY = total correlation spectroscopy
TSTU = O-(*N*-succinimidyl)-*N*,*N*,*N*',*N*'-tetramethyluronium tetrafluoroborate
TMR = tetramethylrhodamine
U2OS = human osteosarcoma cell line
Vim = vimentin

Synthetic procedures

ARose

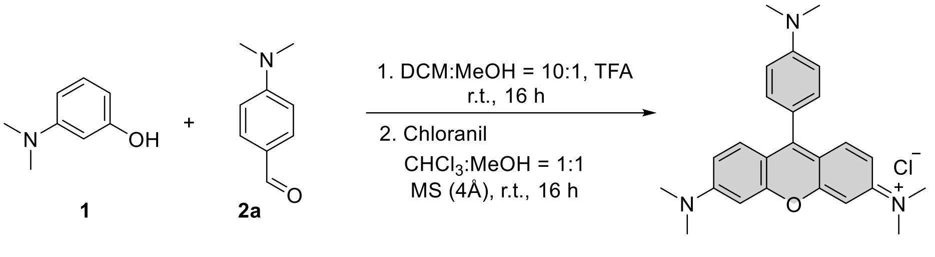

ARose: 85%

In a round bottom flask, compounds **1** (0.75 g, 5.0 mmol, 2.1 equiv) and **2a** (1.44 g, 10.5 mmol, 1.0 equiv) were dissolved in a solvent mixture of CH_2Cl_2 (30 mL) and MeOH (3.0 mL), and TFA (250 µL) was added. The reaction mixture was stirred overnight at room temperature. After the TLC analysis showed completion, the solvents were removed under the *vacuum*. The crude mixture was used in the next step.

The crude oil was dissolved in a solvent mixture of $CHCl_3$ (30 mL) and MeOH (30 mL), and then chloranil (1.35 g, 5.5 mmol, 1.1 equiv) and molecular sieves (4Å) were added. The reaction mixture was stirred at room temperature overnight. The solvents were removed under *vacuum*, and the crude mixture applied to silica gel. Purification by automated flash column chromatography using CH_2Cl_2/MeOH = 98:2 to 90:10 as a gradient yielded the final product as a purple solid (1.79 g, 4.24 mmol, 85%).

^{1}H NMR (400 MHz, CD_3OD) δ = 7.58 (d, *J*=9.6, 2H), 7.33 (d, *J*=8.9, 2H), 7.01 (dd, *J*=9.5, 2.6, 2H), 6.97 (d, *J*=8.9, 2H), 6.81 (d, *J*=2.6, 2H), 3.26 (s, 12H), 3.14 (s, 6H) ppm. **^{13}C NMR** (100 MHz, CD_3OD) δ = 159.0, 157.6, 156.9, 152.3, 132.0, 131.9, 118.3, 113.4, 112.6, 111.3, 96.1, 39.4, 38.9 ppm. **HRMS** (ESI+, *m/z*): M^+ calcd for $C_{25}H_{28}N_3O^+$, 386.2227; found, 386.2230.

P-ARose

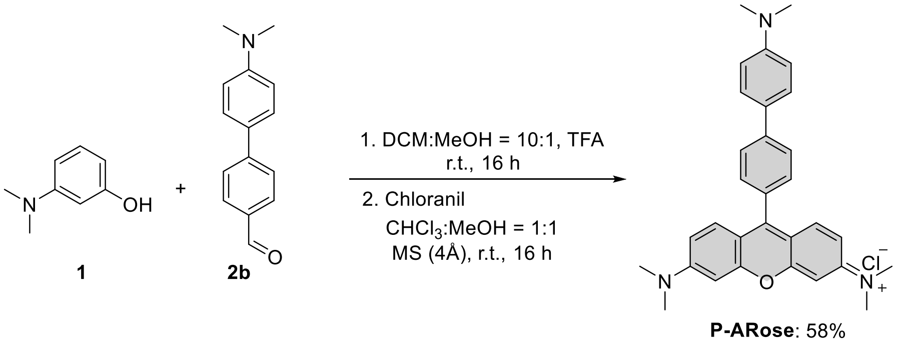

P-ARose: 58%

In a round bottom flask, compounds **1** (0.19 g, 1.4 mmol, 2.1 equiv) and **2b** (0.15 g, 0.67 mmol, 1.0 equiv) were dissolved in a solvent mixture of CH_2Cl_2 (10 mL) and MeOH (1.0 mL), and TFA (50 µL) was added. The reaction mixture was stirred overnight at room temperature. After the TLC analysis showed completion, the solvents were removed under *vacuum*. The crude mixture was used in the next step.

The crude oil was dissolved in a solvent mixture of $CHCl_3$ (8.0 mL) and MeOH (8.0 mL), and then chloranil (0.18 g, 0.74 mmol, 1.1 equiv) and molecular sieves (0.25 g, 4Å) were added. The reaction mixture was stirred at room temperature overnight. The solvents were removed under *vacuum* and the crude mixture applied to silica gel. Purification by automated flash column chromatography using CH_2Cl_2/MeOH = 98:2 to 90:10 as a gradient yielded the final product as a purple solid (0.19 g, 0.39 mmol, 58%).

^{1}H NMR (400 MHz, CD_3OD) δ = 7.93 – 7.89 (m, 2H), 7.72 – 7.69 (m, 2H), 7.56 (d, *J*=9.6, 2H), 7.53 (d, *J*=8.6, 2H), 7.14 (dd, *J*=9.6, 2.5, 2H), 7.01 (d, *J*=2.5, 2H), 6.99 (d, *J*=8.9, 2H), 3.34 (s, 12H), 3.06 (s, 6H) ppm. **^{13}C NMR** (100 MHz, CD_3OD) δ = 161.6, 158.1, 157.8, 157.4, 148.9, 142.7, 131.4, 130.2, 129.7, 127.6, 125.94, 114.5, 114.0, 113.0, 96.2, 40.7, 39.5 ppm. **HRMS** (ESI+, *m/z*): M^+ calcd for $C_{31}H_{32}N_3O^+$, 462.2540; found, 462.2541.

Me-ARose

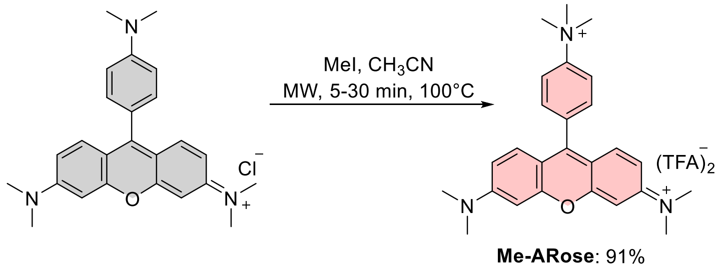

In a 5 mL Biotage® microwave reaction vial, **ARose** (50 mg, 0.12 mmol) was dissolved in CH_3CN (1.0 mL) and MeI (1.0 mL). The reaction mixture was heated at 100 °C for 30 min, cooled to room temperature and the solvents removed under *vacuum*. The crude mixture was purified by RP-HPLC (CH_3CN/H_2O = 2:98 to 80:20, 0.1% TFA, 30 min), yielding 65 mg of the pure purple solid (0.11 mmol, 91%).

^{1}H NMR (400 MHz, CD_3OD) δ = 8.25 (d, *J*=9.1, 1H), 7.80 (d, *J*=9.1, 1H), 7.32 (d, *J*=9.6, 1H), 7.12 (dd, *J*=9.6, 2.5, 2H), 7.04 (d, *J*=2.5, 2H), 3.82 (s, 9H), 3.35 (s, 12H) ppm. **^{13}C NMR** (100 MHz, CD_3OD) δ = 157.9, 157.7, 155.1, 148.3, 134.7, 131.5, 130.8, 120.8, 114.4, 113.1, 96.4, 56.5, 39.6 ppm. **^{19}F NMR** (376 MHz, CD_3OD) δ = -77.2 ppm. **HRMS** (ESI+, *m/z*): M^{2+} calcd for $C_{26}H_{31}N_3O^{2+}$, 200.6228; found, 200.6231.

MeP-ARose

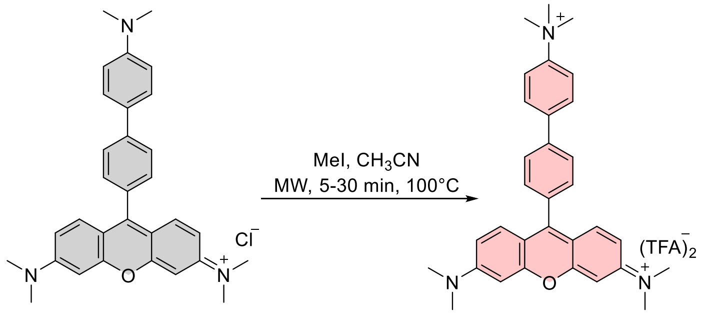

MeP-ARose: 73%

In a 5 mL Biotage® microwave reaction vial, **P-ARose** (50 mg, 0.11 mmol) was dissolved in CH_3CN (1.0 mL) and MeI (1.0 mL). The reaction mixture was heated at 100 °C for 30 min, cooled to room temperature and the solvents removed under *vacuum*. The crude mixture was purified by RP-HPLC (CH_3CN/H_2O = 2:98 to 80:20, 0.1% TFA, 30 min), yielding 54 mg of the pure purple solid (80 µmol, 73%).

^{1}H NMR (400 MHz, CD_3OD) δ = 8.09 (d, *J*=3.0, 2H), 8.03 (d, *J*=8.5, 2H), 7.67 – 7.64 (m, 2H), 7.48 (d, *J*=9.6, 2H), 7.14 (dd, *J*=9.6, 2.5, 2H), 7.02 (d, *J*=2.5, 2H), 3.76 (s, 9H), 3.34 (s, 12H) ppm. **^{13}C NMR** (100 MHz, CD_3OD) δ = 157.9, 157.6, 157.5, 146.8, 141.9, 140.4, 132.3, 131.3, 130.3, 128.7, 127.3, 120.6, 114.2, 113.2, 96.2, 56.4, 39.5 ppm. **^{19}F NMR** (376 MHz, CD_3OD) δ = -77.1 ppm. **HRMS** (ESI+, *m/z*): M^{2+} calcd for $C_{32}H_{35}N_3O^{2+}$, 238.6385; found, 238.6387.

Compounds 4-6

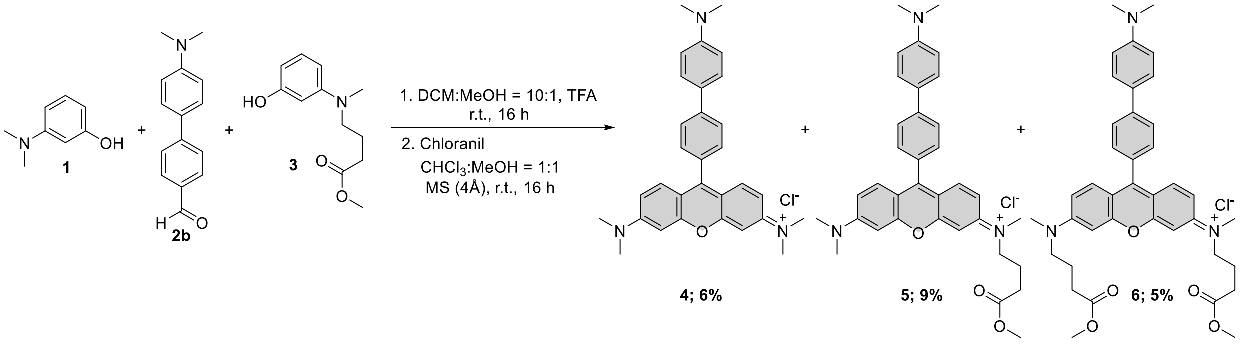

Compound **4** was identical to **P-ARose**. Thus, its full characterization is given above.

Due to a complex mixture of products, the purification of each compound was performed twice, but still yielded slightly impure compounds **4-6**. Therefore, **5** and **6** were fully characterized after ester hydrolysis and HPLC purification (*vide infra*).

In a round bottom flask, compounds **1** (0.14 g, 1.0 mmol, 1.0 equiv), **2b** (0.23 g, 1.0 mmol, 1.0 equiv) and **3** (223 mg, 1.0 mmol, 1.0 equiv) were dissolved in a solvent mixture of CH_2Cl_2 (5.0 mL) and MeOH (0.5 mL), and TFA (50 µL) was added. The reaction mixture was stirred overnight at room temperature. After the TLC analysis showed completion, the solvents were removed under *vacuum*. The crude mixture was used in the next step.

The crude oil was dissolved in a solvent mixture of $CHCl_3$ (5.0 mL) and MeOH (5.0 mL), and chloranil (0.30 g, 1.2 mmol, 1.2 equiv) and molecular sieves (0.35 g, 4Å) were added. The reaction mixture was stirred at room temperature overnight. The solvents were removed under *vacuum* and the crude mixture applied to silica gel. Two purifications by automated flash column chromatography using CH_2Cl_2/MeOH = 98:2 to 90:10 as a gradient yielded compound **4** (30 mg, 0.06 mmol, 6%), **5** (53 mg, 0.09 mmol, 9%) and **6** (32 mg, 0.05 mmol, 5%) as purple solids.

Compound **5**

^{1}H NMR (400 MHz, CD_3OD) δ = 7.92 (d, *J*=8.6, 2H), 7.76 (d, *J*=9.0, 2H), 7.58 – 7.50 (m, 4H), 7.24 – 6.98 (m, 6H), 3.76 – 3.68 (m, 8H), 3.34 (s, 6H), 3.30 (m, 6H), 3.12 (s, 6H), 2.48 (t, *J*=6.8, 2H), 2.01 (p, *J*=7.0, 2H) ppm. **HRMS** (ESI+, *m/z*): M^+ calcd for $C_{35}H_{38}N_3O_3^+$, 548.2908; found, 548.2924.

Compound **6**

^{1}H NMR (400 MHz, CD_3OD) δ = 7.92 (d, *J*=8.6, 2H), 7.80 (d, *J*=8.8, 2H), 7.54 (d, *J*=8.6, 2H), 7.50 (d, *J*=9.5, 2H), 7.25 (d, *J*=8.9, 2H), 7.15 (dd, *J*=9.5, 2.3, 2H), 7.05 (s, 2H), 3.70 (s, 10H), 3.29 (s, 6H), 3.17 (s, 6H), 2.48 (t, *J*=6.8, 4H), 2.00 (p, *J*=7.0, 4H) ppm. **HRMS** (ESI+, *m/z*): M^+ calcd for $C_{39}H_{44}N_3O_5^+$, 634.3275; found, 634.3281.

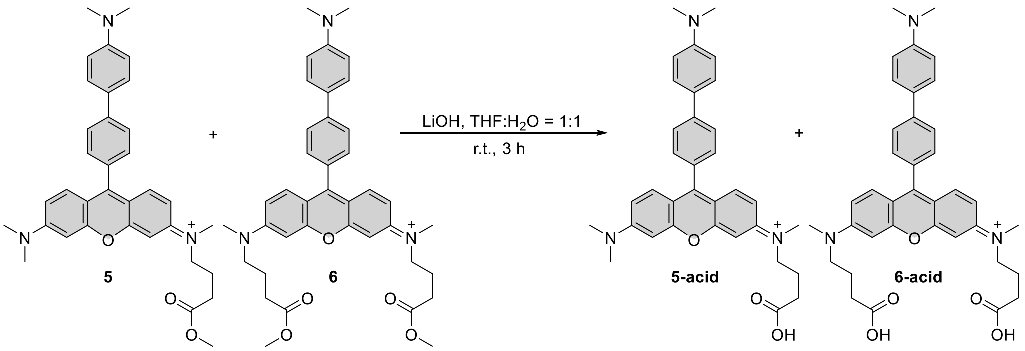

Compound **5** (25 mg, 43 µmol, 1.0 equiv) was dissolved in THF/H_2O = 1:1 solvent mixture (2.0 mL total volume) and LiOH monohydrate (3.8 mg, 0.09 mmol, 2.0 equiv) was added. The reaction mixture was stirred at room temperature for 3 hours, at which point LCMS analysis showed a complete conversion. The organic solvent was removed under *vacuum*, and the crude salt purified by HPLC (CH_3CN/H_2O = 5:95 to 100:0, 0.1% TFA, 30 min), yielding a pure product as a purple solid (21 mg, 33 µmol, 77%).

^{1}H NMR (400 MHz, CD_3OD) δ = 7.91 (d, *J*=8.6, 2H), 7.76 (d, *J*=8.8, 2H), 7.55 – 7.47 (m, 4H), 7.19 – 6.96 (m, 6H), 3.74 – 3.66 (m, 2H), 3.32 (d, *J*=0.9, 6H), 3.29 (s, 3H), 3.13 (s, 6H), 2.44 (t, *J*=6.8, 2H), 1.98 (p, *J*=7.0, 2H) ppm. **^{13}C NMR** (100 MHz, CD_3OD) δ = 175.0, 158.1, 158.0, 157.9, 157.5, 156.9, 148.5, 142.6, 131.6, 131.5, 130.2, 129.9, 127.7, 126.1, 115.0, 114.1, 113.2, 113.1, 96.3, 96.2, 51.8, 41.1, 39.5, 38.1, 29.9, 21.8 ppm (some carbons are missing due to overlap). **^{19}F NMR** (376 MHz, CD_3OD) δ = -77.1 ppm. **HRMS** (ESI+, *m/z*): M^+ calcd for $C_{34}H_{36}N_3O_3^+$, 534.2751; found, 534.2751.

Compound **6** (50 mg, 79 µmol, 1.0 equiv) was dissolved in THF:H_2O = 1:1 (2 mL total volume) solvent mixture and LiOH monohydrate (10 mg, 0.24 mmol, 3.0 equiv) was added. The reaction mixture was stirred at room temperature for 3 hours, at which point LCMS analysis showed a complete conversion.

The organic solvent was removed under *vacuum*, and the crude salt purified by HPLC (CH_3CN/H_2O = 5:95 to 100:0, 0.1% TFA, 30 min), yielding a pure product as a purple solid (41 mg, 0.058 mmol, 74%).

^{1}H NMR (400 MHz, CD_3OD) δ = 7.92 (d, *J*=8.3, 1H), 7.81 (d, *J*=8.8, 1H), 7.54 (d, *J*=8.3, 1H), 7.49 (d, *J*=9.6, 1H), 7.28 (d, *J*=8.8, 1H), 7.20 – 7.13 (m, 1H), 7.04 (s, 1H), 3.74 – 3.66 (m, 3H), 3.30 (s, 4H), 3.18 (s, 4H), 2.44 (t, *J*=6.8, 3H), 1.98 (p, *J*=6.9, 3H) ppm. **^{13}C NMR** (100 MHz, CD_3OD) δ = 175.0, 158.0, 157.9, 156.9, 147.4, 142.3, 131.6, 130.3, 127.9, 126.3, 116.1, 114.2, 113.2, 96.3, 51.8, 41.9, 38.1, 29.9, 21.8 ppm (some carbons are missing due to overlap). **^{19}F NMR** (376 MHz, CD_3OD) δ = -77.22 ppm. **HRMS** (ESI+, *m/z*): M$^+$ calcd for $C_{37}H_{40}N_3O_5^+$, 606.2962; found, 606.2958.

Compound 10

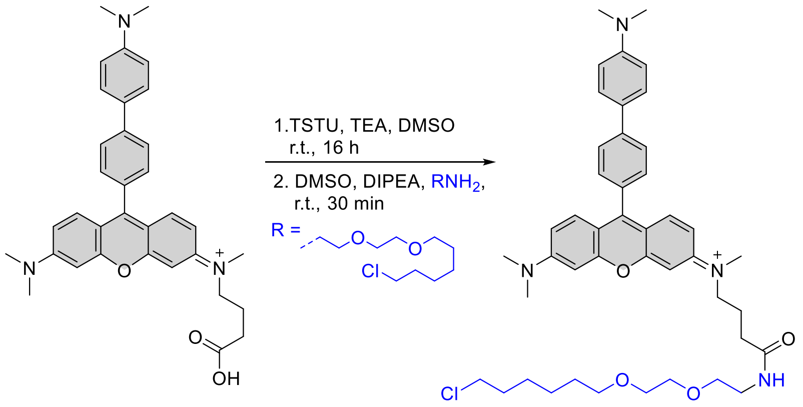

Compound **5-acid** (11 mg, 17 µmol, 1.0 equiv) and TSTU (7.7 mg, 26 µmmol, 1.5 equiv) were dissolved in DMSO (2.0 mL) followed by triethylamine addition (7.1 µL, 51 µmol, 3.0 equiv). The reaction mixture was stirred at room temperature overnight. After 16 hours, TLC and LCMS analysis (the product is poorly ionizable) showed full conversion. The crude mixture was diluted with water and CH_3CN, and purified by HPLC (CH_3CN/H_2O = 10:90 to 100:0, 0.1% TFA, 30 min), yielding a pure compound **7** as a purple solid (11 mg, 15 µmol, 88%).

NHS ester **7** (3.5 mg, 4.8 µmol, 1.0 equiv) was dissolved in CH_2Cl_2 (1.0 mL) followed by addition of O_2-HaloTag ligand hydrochloride salt (1.4 mg, 5.3 µmol, 1.1 equiv) and DIPEA (4.2 µL, 24 µmol, 5.0 equiv). After 30 minutes, LCMS analysis showed full conversion, CH_2Cl_2 was evaporated and the crude mixture purified by RP-HPLC (CH_3CN/H_2O = 20:80 to 100:0, 0.1% TFA, 30 min), yielding a pure compound **10** as a purple solid (3.2 mg, 3.8 µmol, 80%).

^{1}H NMR (400 MHz, CD_3OD) δ = 7.93 (d, *J*=8.6, 2H), 7.76 (d, *J*=8.9, 2H), 7.57 – 7.51 (m, 4H), 7.23 – 6.99 (m, 6H), 3.74 – 3.65 (m, 2H), 3.60 – 3.47 (m, 9H), 3.43 (t, *J*=6.6, 2H), 3.38 (t, *J*=5.3, 2H), 3.34 (s, 6H), 2.33 (t, *J*=6.9, 2H), 2.02 (p, *J*=7.1, 2H), 1.78 – 1.65 (m, 2H), 1.59 – 1.49 (m, 2H), 1.47 – 1.27 (m, 6H) ppm. **^{19}F NMR** (376 MHz, CD_3OD) δ = -77.2 ppm. **HRMS** (ESI+, *m/z*): M$^+$ calcd for $C_{44}H_{56}N_4O_4Cl^+$, 739.3985; found, 739.4017.

Compound 11

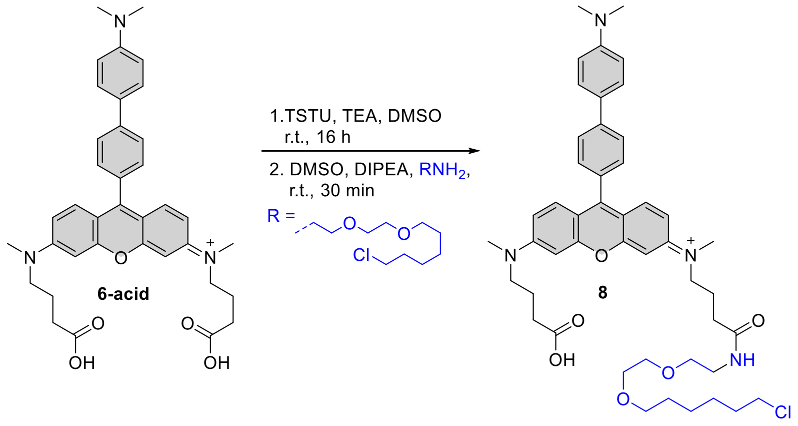

Compound **6-acid** (11 mg, 16 µmol, 1.0 equiv) and TSTU (5.7 mg, 19 µmol, 1.2 equiv) were dissolved in DMSO (2.0 mL) followed by triethylamine addition (7.1 µL, 47 µmol, 3.0 equiv). The reaction mixture was stirred at room temperature overnight. After 16 hours, TLC and LCMS analysis (the product is poorly ionizable) showed full conversion. Half of the crude mixture was directly used in the next step.

To NHS ester **8** (5.5 mg, 7.8 µmol, 1.0 equiv) in DMSO (1.0 mL) was added O_2-HaloTag ligand hydrochloride salt (3.0 mg, 12 µmol, 1.5 equiv) and DIPEA (4.0 µL, 24 µmol, 3 equiv). After 30 minutes, LCMS analysis showed full conversion, and the crude mixture was directly purified by RP-HPLC (CH_3CN/H_2O = 20:80 to 100:0, 0.1% TFA, 30 min), yielding a pure compound **11** as a purple solid (3.6 mg, 4.0 µmol, 51%).

^{1}H NMR (400 MHz, CD_3OD) δ = 7.93 (d, *J*=8.6, 2H), 7.75 (d, *J*=8.9, 2H), 7.55 (dd, *J*=9.1, 1.8, 4H), 7.20 (t, *J*=8.7, 2H), 7.12 – 7.04 (m, 4H), 3.77 – 3.67 (m, 4H), 3.60 – 3.47 (m, 8H), 3.43 (t, *J*=6.6, 2H), 3.38 (t, *J*=5.4, 2H), 3.35 (s, 3H), 3.32-3.31 (m, 2H), 3.10 (s, 6H), 2.45 (t, *J*=6.8, 2H), 2.33 (t, *J*=6.9, 2H), 2.01 (h, *J*=7.5, 4H), 1.71 (dq, *J*=7.9, 6.7, 2H), 1.53 (dt, *J*=13.9, 6.8, 2H), 1.46 – 1.28 (m, 5H) ppm. **^{19}F NMR** (376 MHz, CD_3OD) δ = -77.3 ppm. **HRMS** (ESI+, *m/z*): M^+ calcd for $C_{47}H_{60}N_4O_6Cl^+$, 811.4196; found, 811.4231.

Compound 12

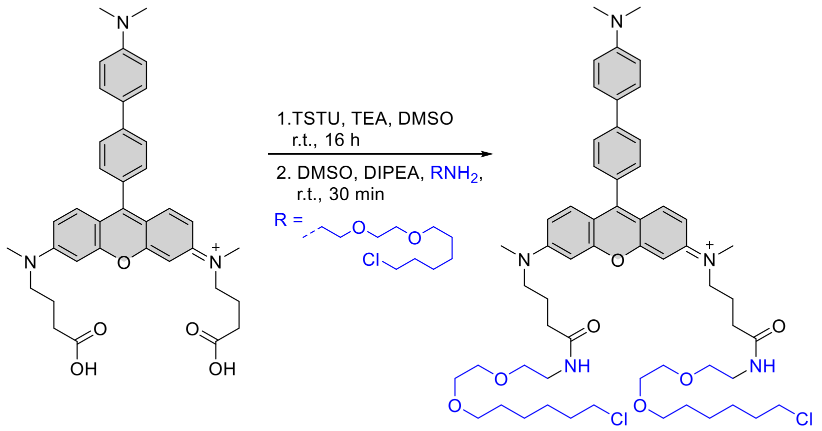

Compound **6-acid** (5.0 mg, 7.1 µmol, 1.0 equiv) and TSTU (5.6 mg, 18 µmol, 2.4 equiv) were dissolved in DMSO (1.0 mL) followed by addition (3 µL, 21 µmol, 3.0 equiv). The reaction mixture was stirred at room temperature overnight. After 16 hours, TLC and LCMS analysis (the product is poorly ionizable) showed full conversion. The crude mixture was directly used in the next step.

To NHS ester **9** (6.4 mg, 7.1 µmol, 1.0 equiv) in DMSO (1.0 mL) was added O_2-HaloTag ligand hydrochloride salt (4.4 mg, 17 µmol, 2.4 equiv) and DIPEA (6.1 µL, 35 µmol, 5 equiv). After 30 minutes, LCMS analysis showed full conversion, and the crude mixture was directly purified by HPLC (CH_3CN/H_2O = 20:80 to 100:0, 0.1% TFA, 30 min), yielding a pure compound **12** as a purple solid (4.1 mg, 4.6 µmol, 64%).

^{1}H NMR (400 MHz, CD_3OD) δ = 7.92 (d, *J*=8.6, 2H), 7.73 (d, *J*=8.9, 2H), 7.58 – 7.51 (m, 4H), 7.19 (d, *J*=9.5, 2H), 7.09 – 7.00 (m, 4H), 3.74 – 3.67 (m, 4H), 3.60 – 3.48 (m, 20H), 3.43 (t, *J*=6.6, 4H), 3.40 – 3.34 (m, 6H), 3.08 (s, 6H), 2.33 (t, *J*=6.9, 4H), 2.08 – 1.97 (m, 4H), 1.76 – 1.66 (m, 4H), 1.59 – 1.49 (m, 4H), 1.46 – 1.28 (m, 10H) ppm. **^{19}F NMR** (376 MHz, CD_3OD) δ = -77.1 ppm. **HRMS** (ESI+, *m/z*): M^+ calcd for $C_{57}H_{80}N_5O_7Cl_2^+$, 1016.5429; found, 1016.5454.

DFT/TD-DFT calculation of P-ARose

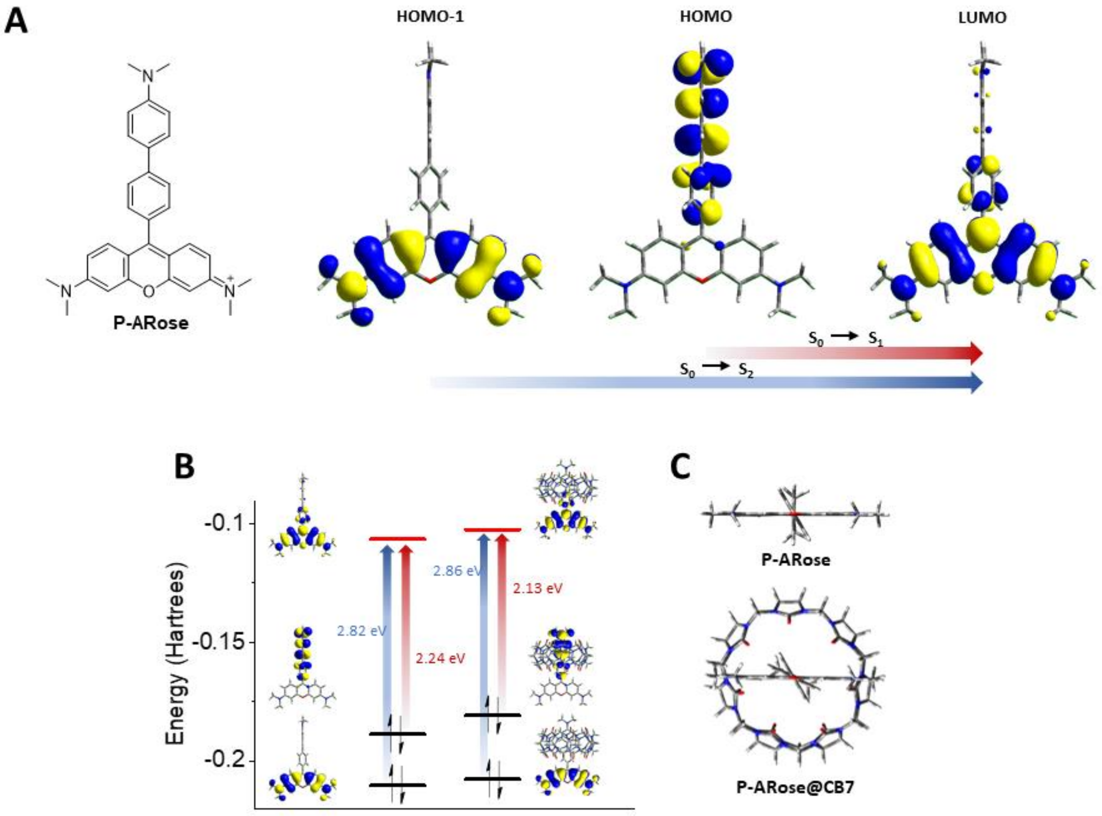

Figure S1. (A) Molecular orbitals involved in the primary transitions in **P-ARose** as calculated using TD-DFT. (B) Effect of CB7 binding on the orbital energies of **P-ARose**. (C) Effect of CB7 binding on the biphenyl-xanthene angle in **P-ARose**. The angle D (C2', C1', C9, C7) is 59.7° for **P-ARose** and 32.3° for **P-ARose@CB7**.

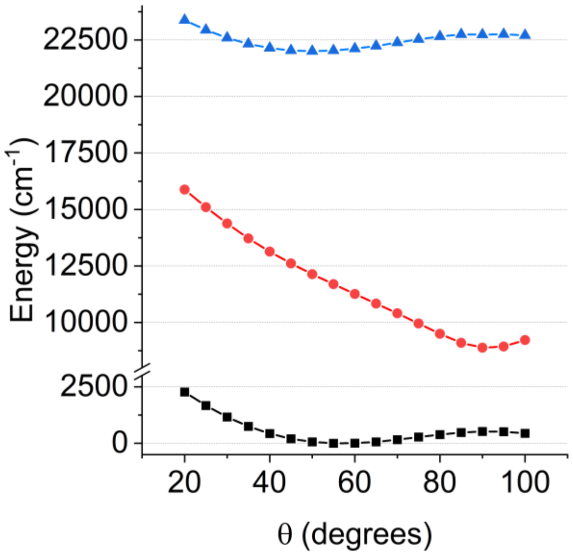

Figure S2. Potential energy surfaces for S_0 (black), S_1 (red, arylpyrylium), and S_2 (blue, xanthylium) of **P-ARose** as a function of the twist angle, θ, generated from the geometries calculated using DFT B3LYP 6-31+G* and vertical excitation energies calculated using TD-DFT. The lowest-energy ground state geometry (θ = 55°) defines the zero-point energy and no relaxation of the molecular structure was allowed after excitation. No solvation was included for consistency with reference.[2]

Solvatochromism

Procedure: The stock solution of **ARose** (2 mM) was prepared in DMSO. For the measurements, 5 µL of the stock solution was added in 3 mL of the corresponding solvent (3.33 µM, 0.17% DMSO) to measure the absorption and emission spectra, and obtain the quantum yields.

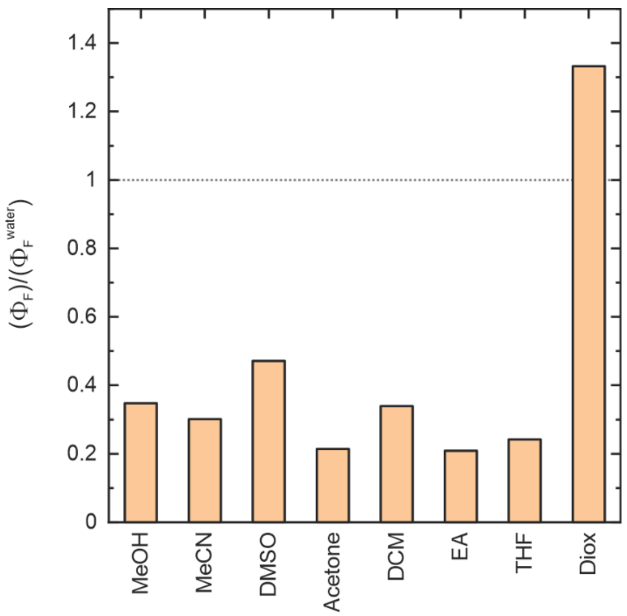

Figure S3. Relative emission quantum yields in series of solvents, relative to the value observed in water. Solvents are ordered by decreasing ET30 value, from left to right.

pH influence on photophysical properties

Buffer preparation. For all pH-dependent measurements a multicomponent buffer was used, and it was prepared as described in literature.[3]

Universal buffer was prepared from 50 mL of the aqueous solution consisting of 0.1 M citric acid (21.01 g/l), 0.1 M KH_2PO_4 (13.61 g/l), 0.1 M sodium tetraborate (19.07 g/l), 0.1 M Tris (12.11 g/l), 0.1 M KCl (7.46 g/l) followed by adjusting the pH to the required values (adding x mL of 0.4 M HCl or 0.4 M NaOH), and finally by the dilution to 200 ml. Buffers in the range of pH 2-11 were prepared in 0.5 increments.

Sample preparation. Stock solutions of dyes were prepared in DMSO (2 mM and 0.4 mM). Additionally, a range of CB7 solutions (0.5 mM) with different pH values (2-11) was prepared by dissolving CB7 in a corresponding buffer. *Note*: buffers with pH between 7.5 and 8.5 were slightly cloudy and therefore filtered before the application making the effective concentration of CB7 slightly lower.

In a glass bottom 96-well plate, the dye solution (1 µl) was pipetted and then buffers (300 µl) of different pH (2-11, increments of 0.5) were added. The 2 mM dye stock solution was used for the CB7-free measurements while 0.5 mM stock solutions were for buffers containing CB7 to ensure full complexation and a reliable change in pKa values. *Note*: due to high dilution, only emission was followed.

Measurements. The measurements were performed on a multiwell plate reader Spark® 20M (Tecan) in glass bottom 96-well plates at room temperature (25°C). Absorption of solutions was recorded from 300 nm to 800 nm with wavelength step size of 1 nm. The background absorption of the glass bottom plate was measured in wells containing only buffer with similar amount of DMSO and subtracted from the spectra of the samples. Fluorescence intensity was recorded using the excitation at 550 nm (5 nm excitation bandwidth) and emission at 570 nm (5 nm emission bandwidth). All the measurements were performed in three technical replicates.

Analysis. The obtained pH vs. fluorescence intensity graphs were fitted using nonlinear curve fit (Logistic function, and fitted until converged), and the pKa values were determined from these fits for **ARose** and **P-ARose**. For the alkylated dyes (**Me-ARose** and **MeP-ARose**) the emission signal did not depend on pH.

Change of the absorption spectra with pH

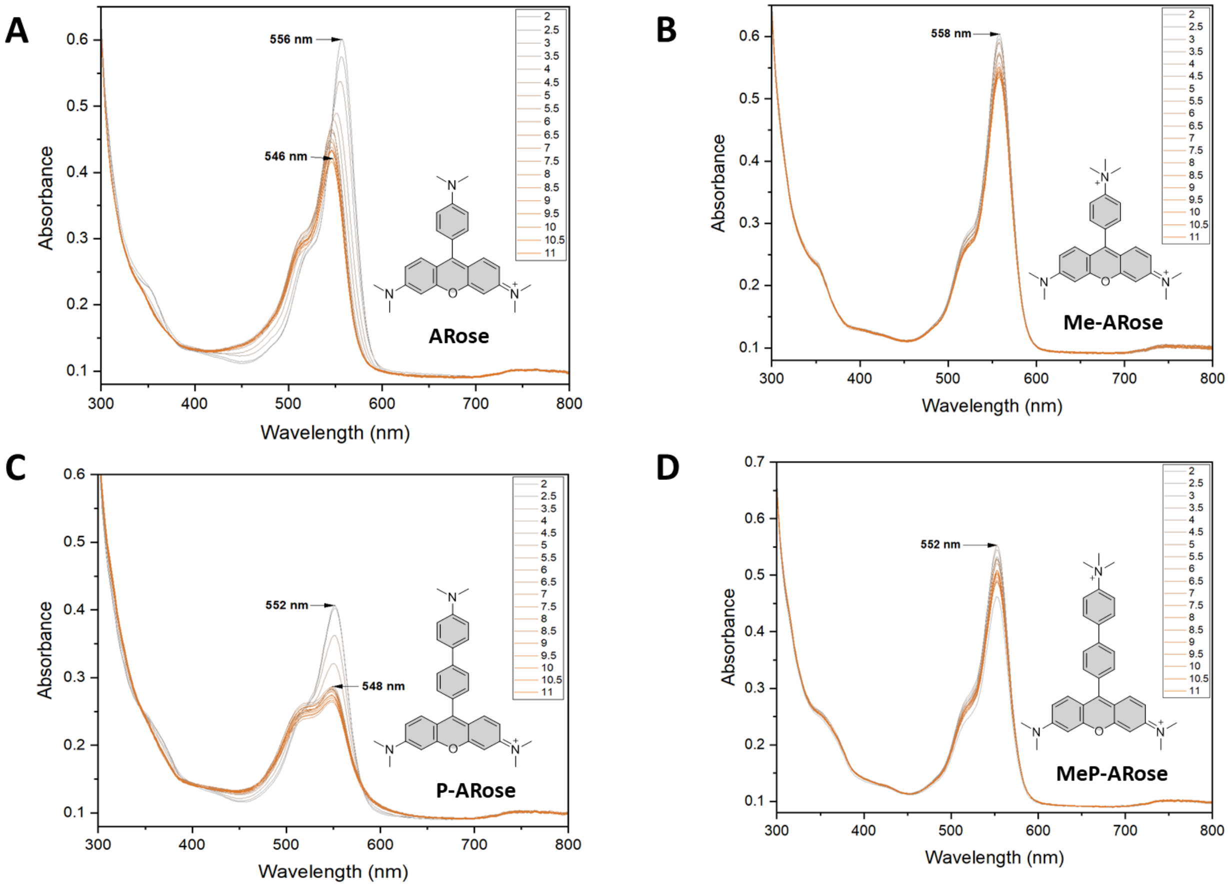

Figure S4. UV-Vis absorption spectra throughout the pH range (2-11) for (**A**) **Arose**, (**B**) **Me-Arose**, (**C**) **P-Arose**, and (**D**) **MeP-Arose**.

The pH-dependent absorption at 550 nm

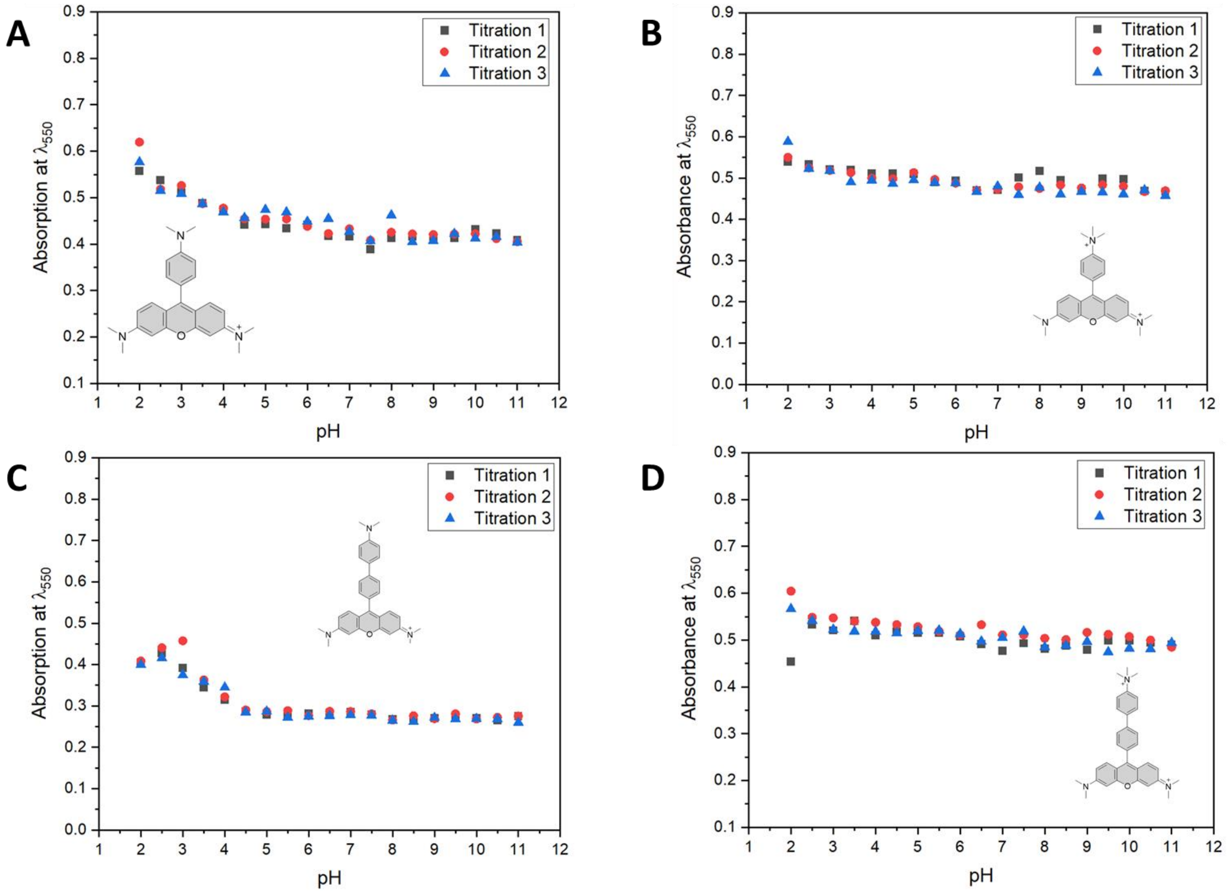

Figure S5. The pH-dependent absorption at 550 nm for (**A**) **ARose**, (**B**) **Me-ARose**, (**C**) **P-ARose**, and (**D**) **MeP-ARose**.

pKa determination: Fluorescence intensity across the pH range

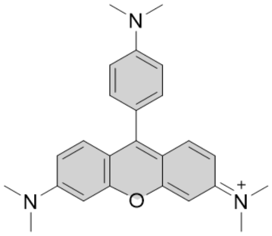

ARose

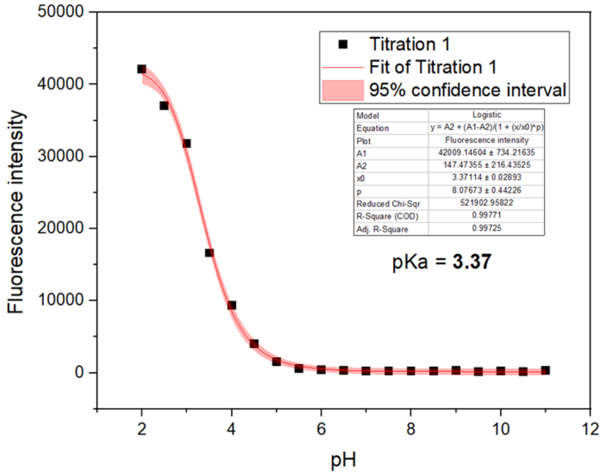

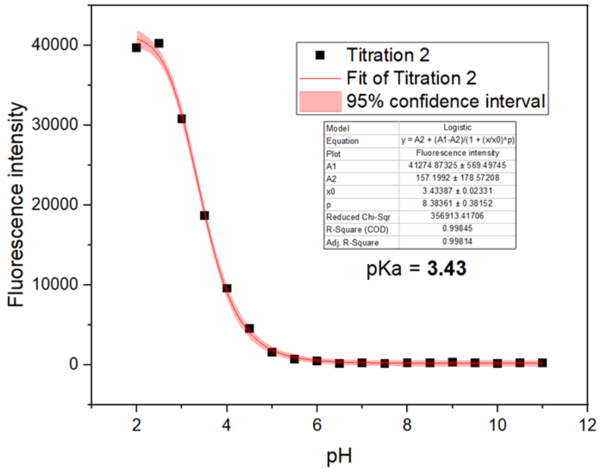

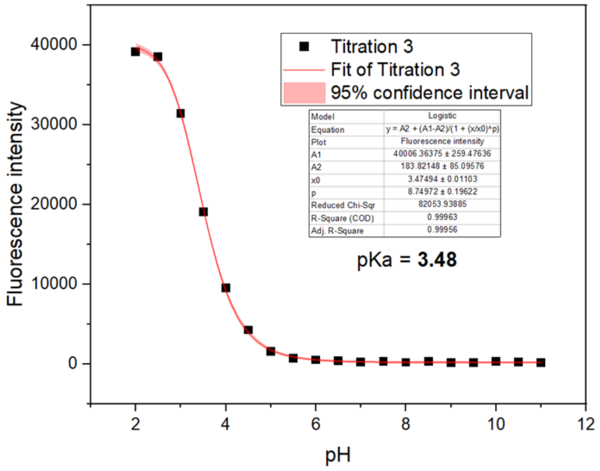

Figure S6. Titration curves of three technical replicates of **ARose** titration and the corresponding pKa values.

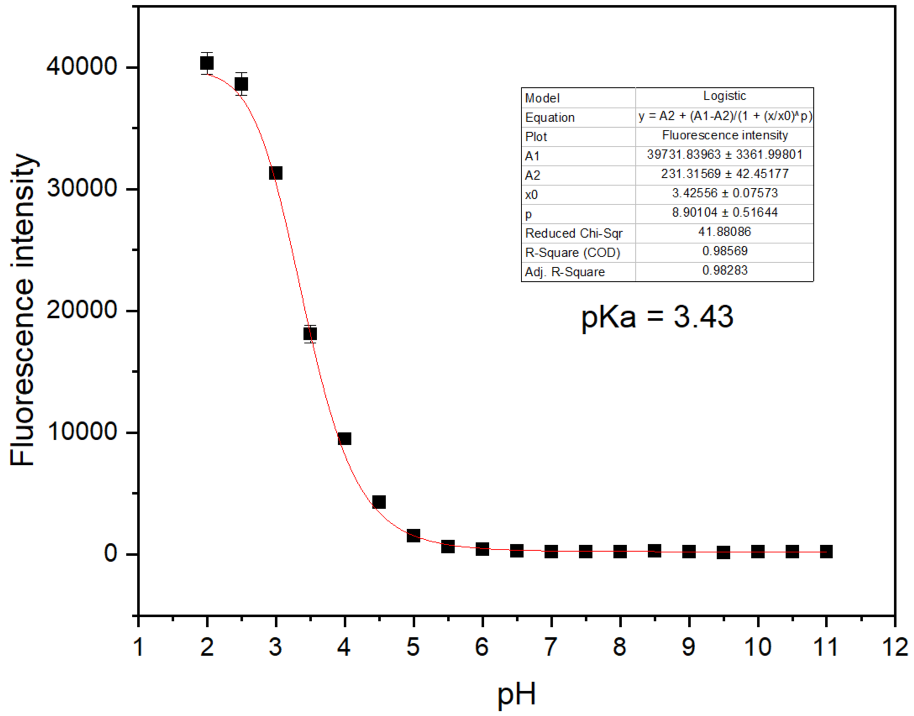

Figure S7. Determination of pKa of **ARose** by titration. The reported value represents the mean of three independent titrations. The error bars represent the standard deviation.

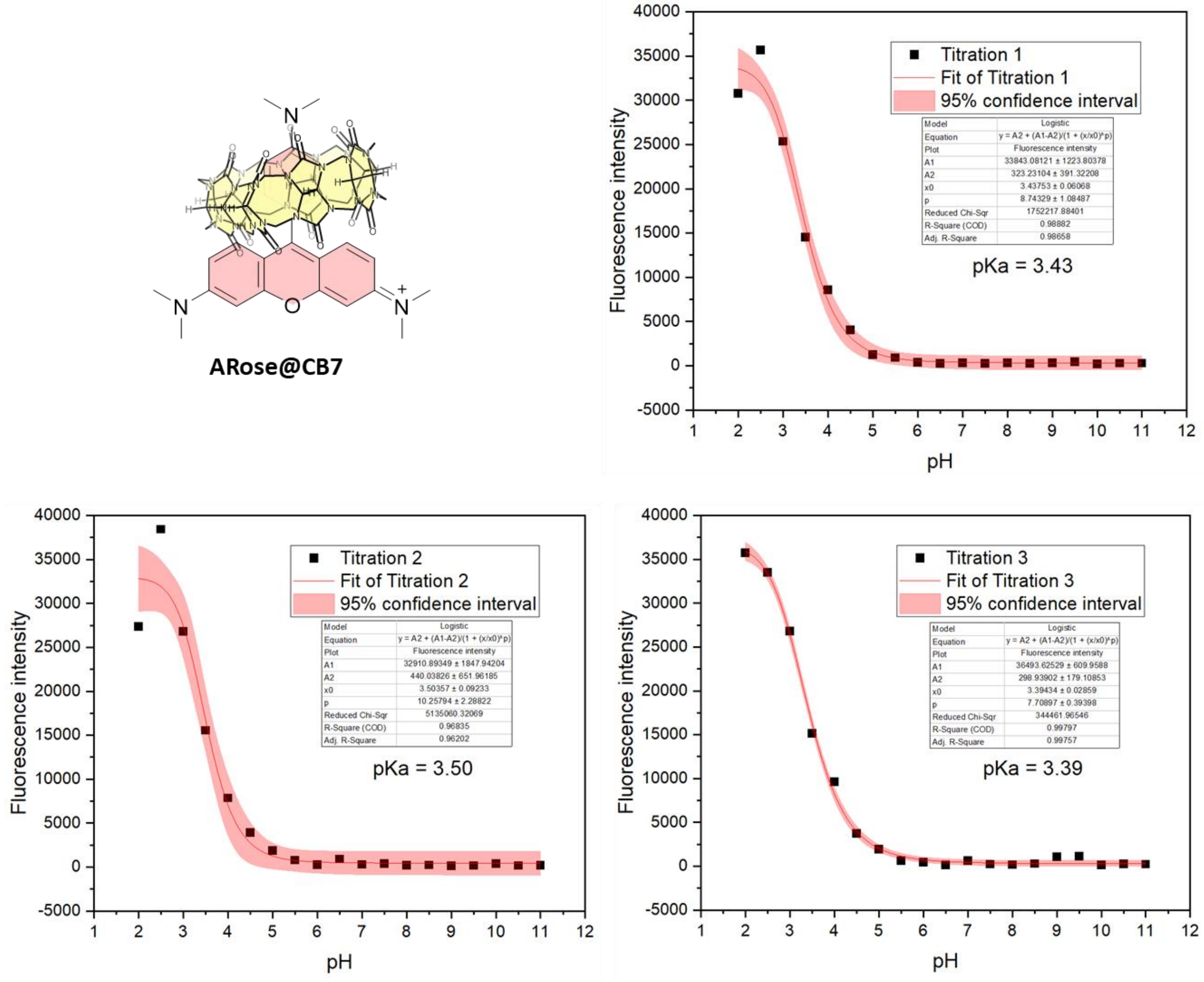

Figure S8. Titration curves of three curves of three technical replicates of **ARose@CB7** titration and the corresponding pKa values.

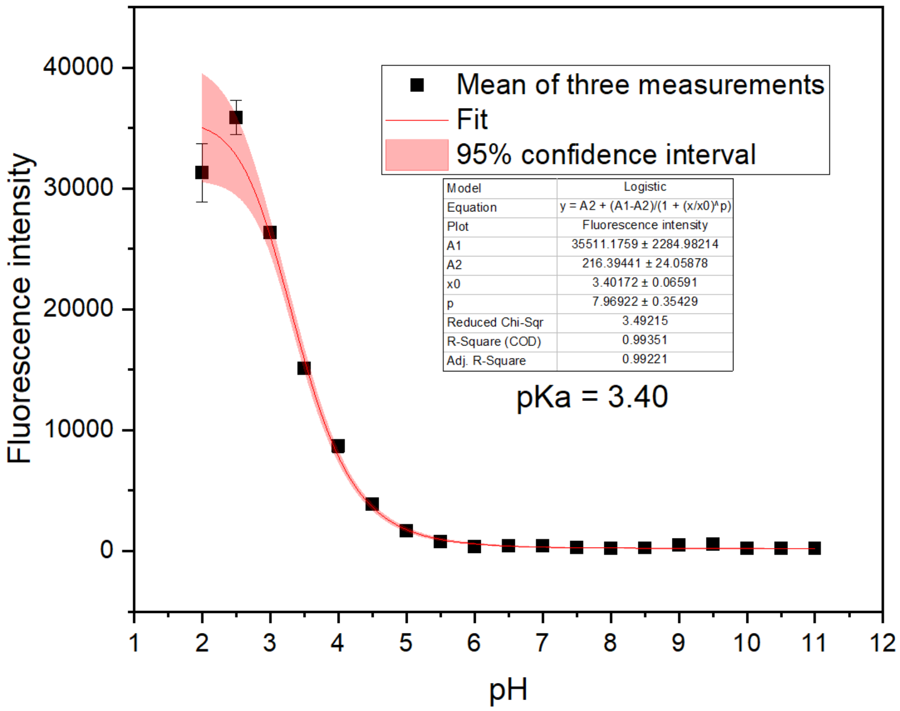

Figure S9. Determination of pKa of **ARose@CB7** by titration. The reported value represents the mean of three independent titrations. The error bars represent the standard deviation.

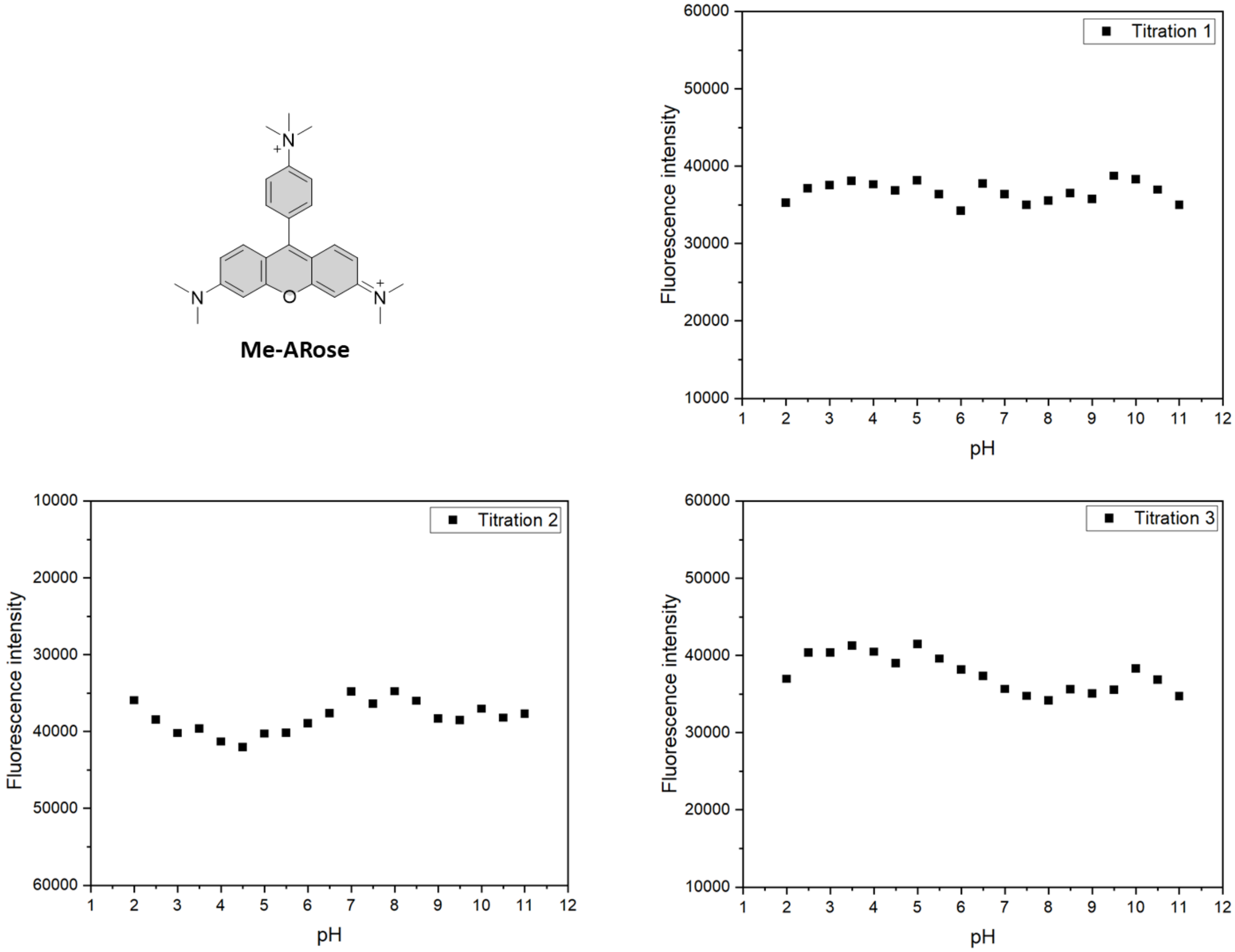

Figure S10. Titration curves of three technical replicates of **Me-ARose** titration.

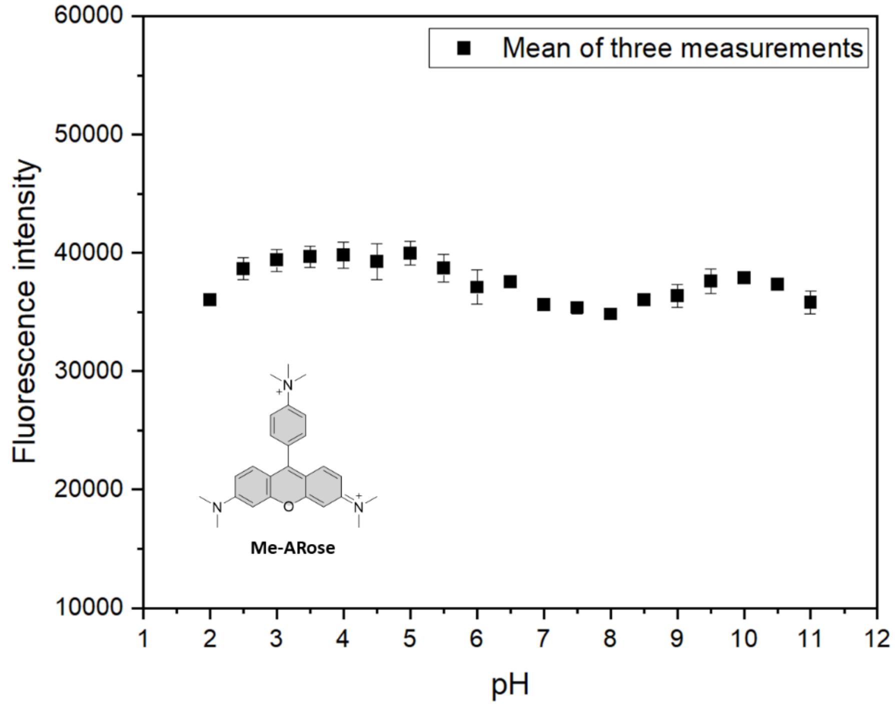

Figure S11. The emission change of **Me-ARose** at 550 nm. The data points represent the mean of three independent measurements. The error bars represent the standard deviation.

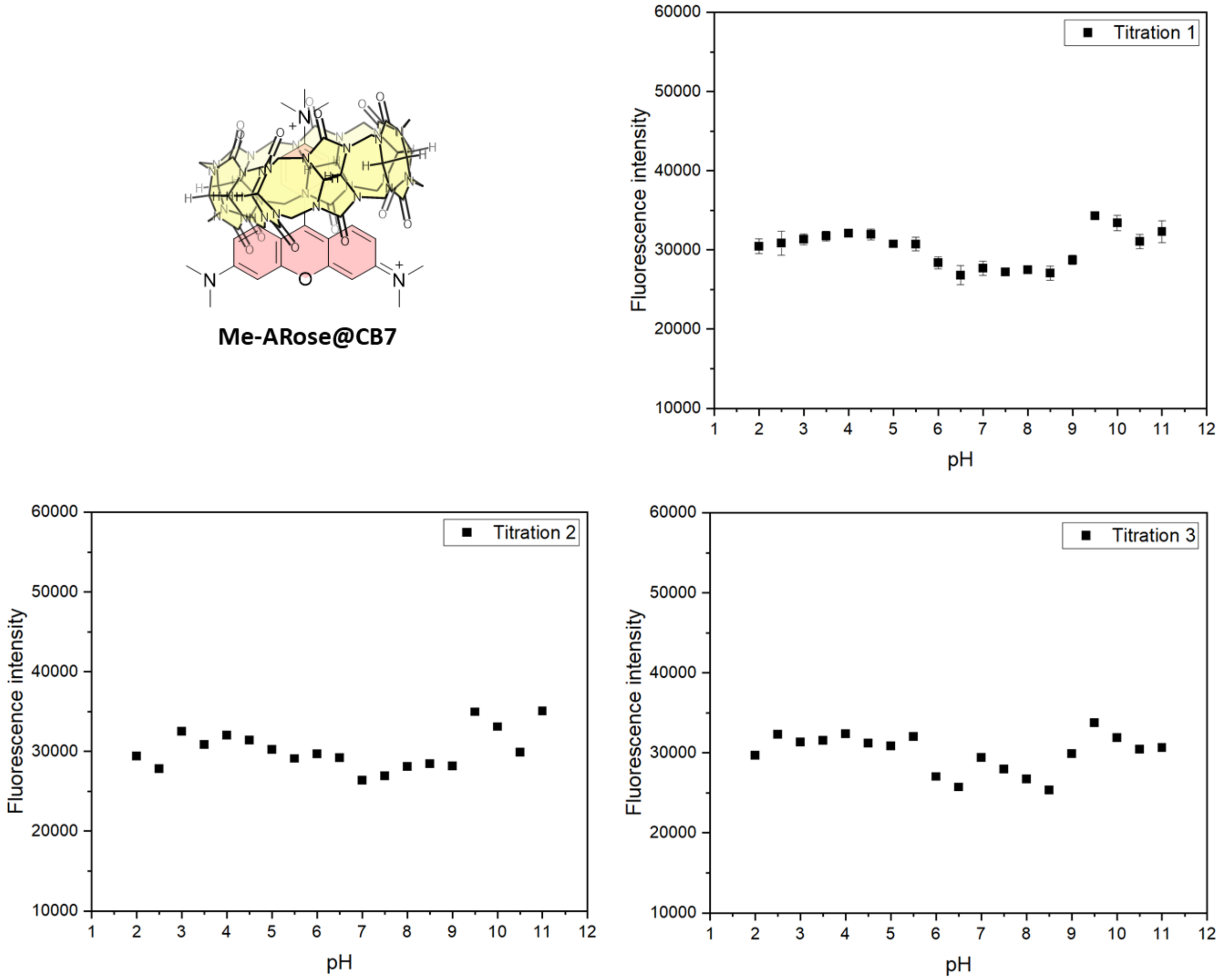

Figure S12. Titration curves of three technical replicates of **Me-ARose@CB7** titration. The error bars represent the standard deviation.

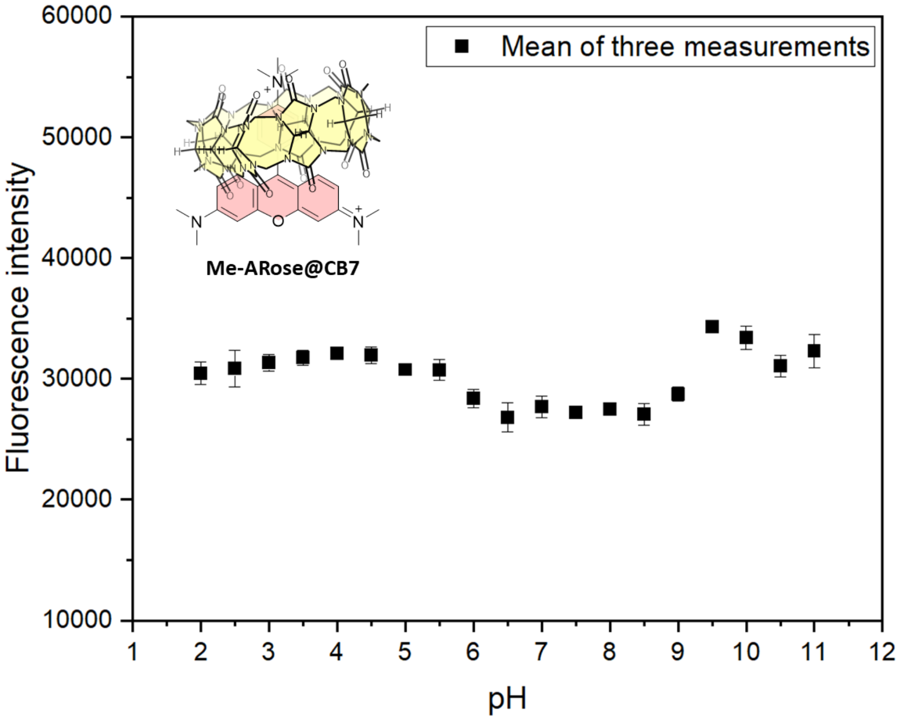

Figure S13. The emission change of **Me-ARose@CB7** at 550 nm. The data points represent the mean of three independent measurements. The error bars represent the standard deviation.

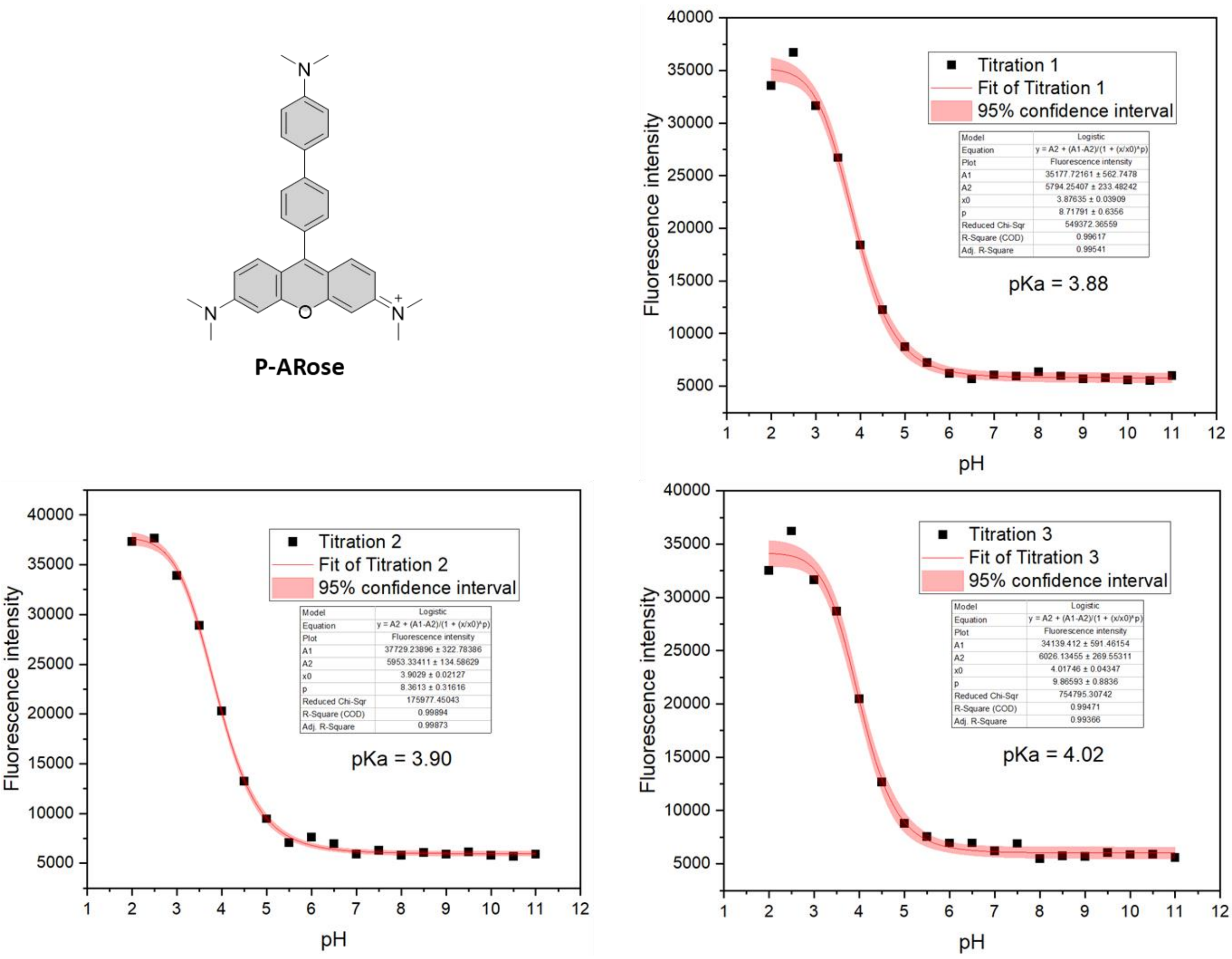

Figure S14. Titration curves of three technical replicates of **P-ARose** titration and the corresponding pKa values.

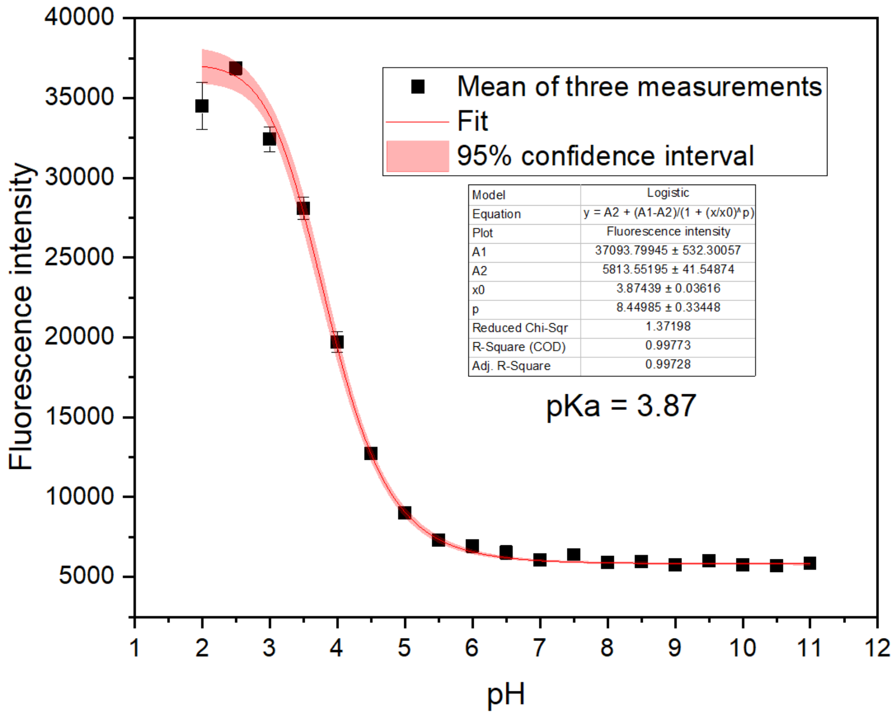

Figure S15. Determination of the pKa of **P-ARose** by titration. The value represents the mean of three independent titrations.

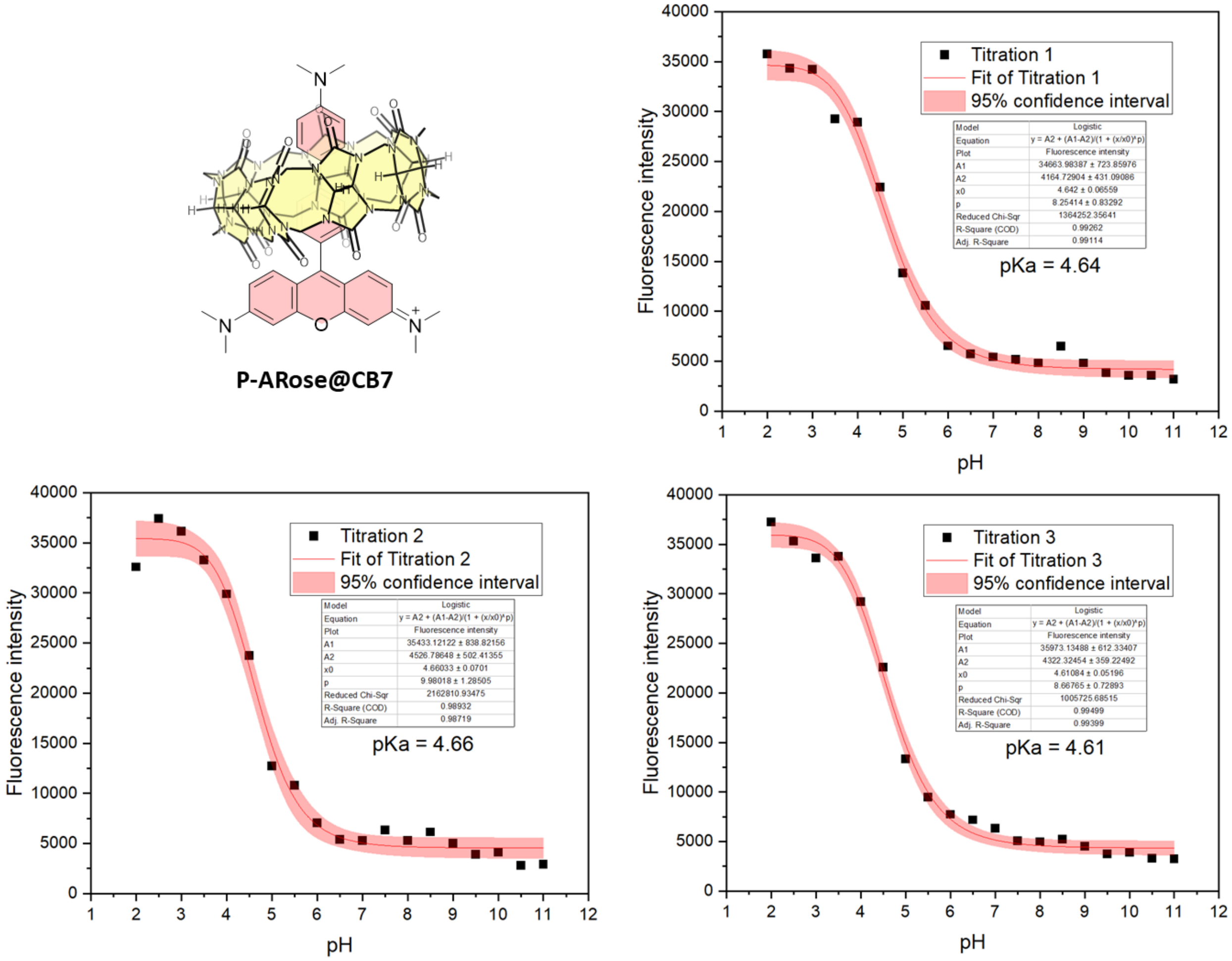

Figure S16. Titration curves of three technical replicates of **P-ARose@CB7** titration and the corresponding pKa values.

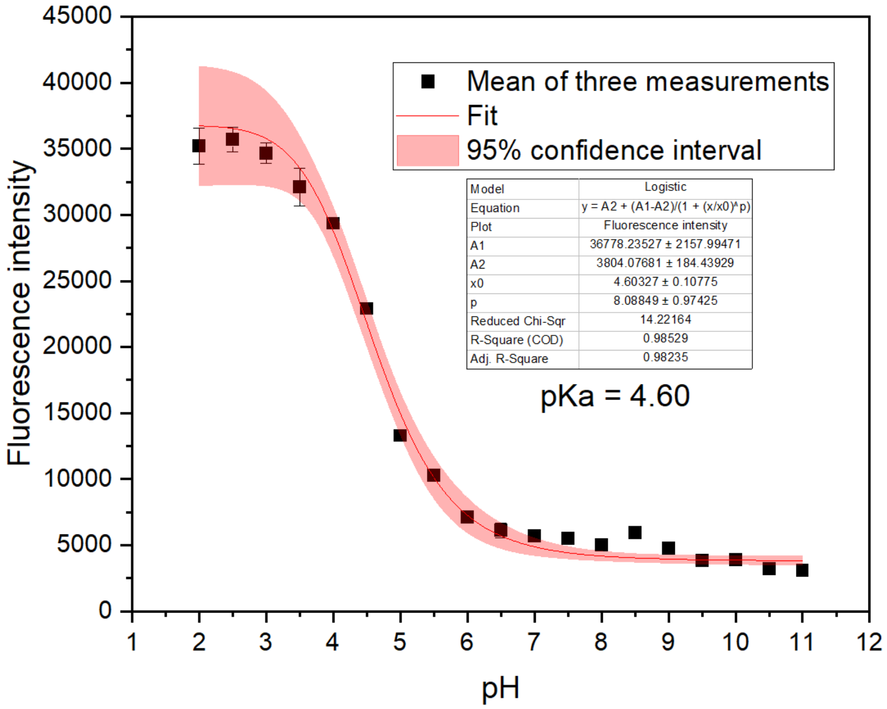

Figure S17. Determination of pKa for **P-ARose@CB7** by titration. The given value represents the mean of three independent titrations. The error bars represent the standard deviation.

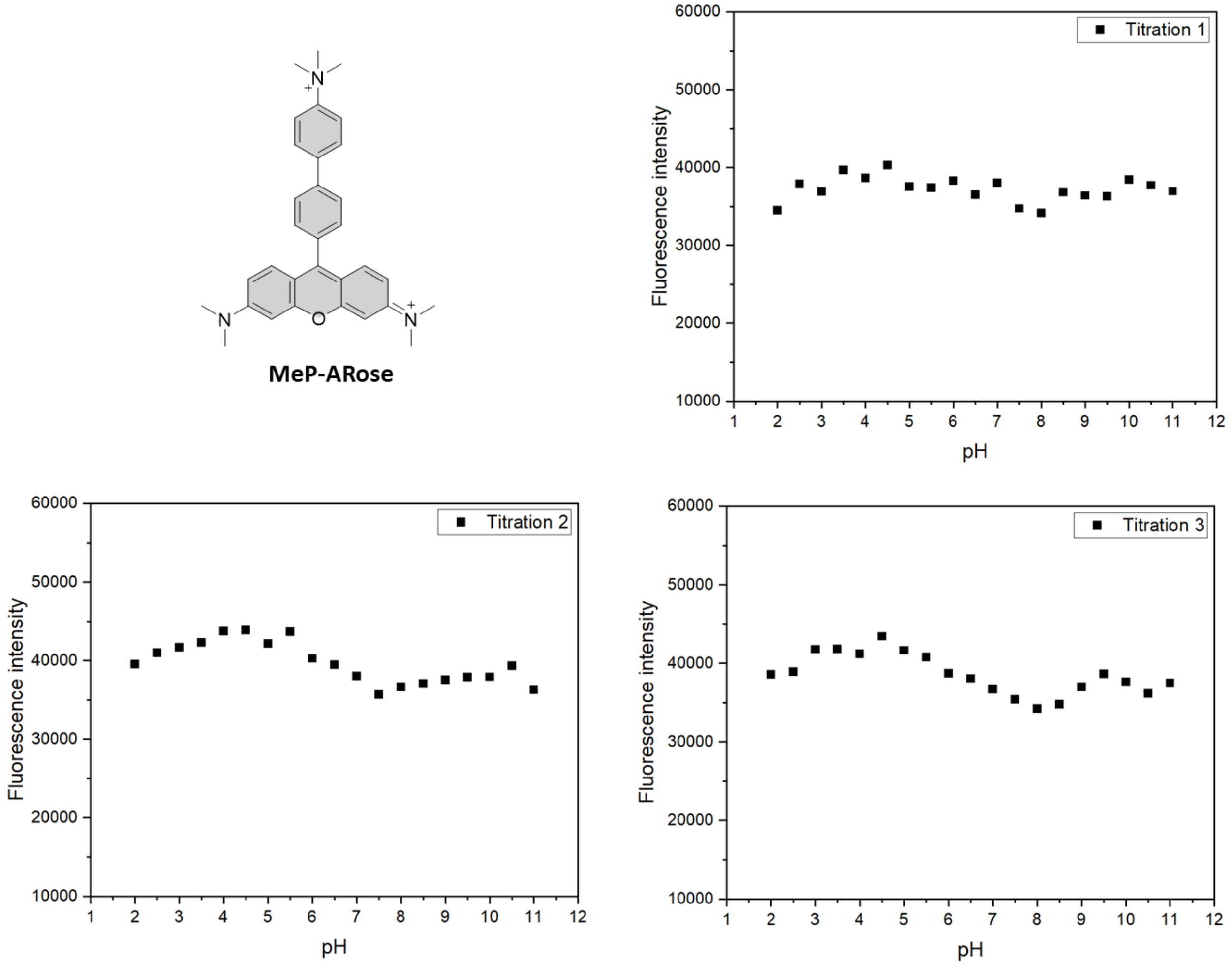

Figure S18. Titration curves of three technical replicates of **MeP-ARose** titration and change of emission.

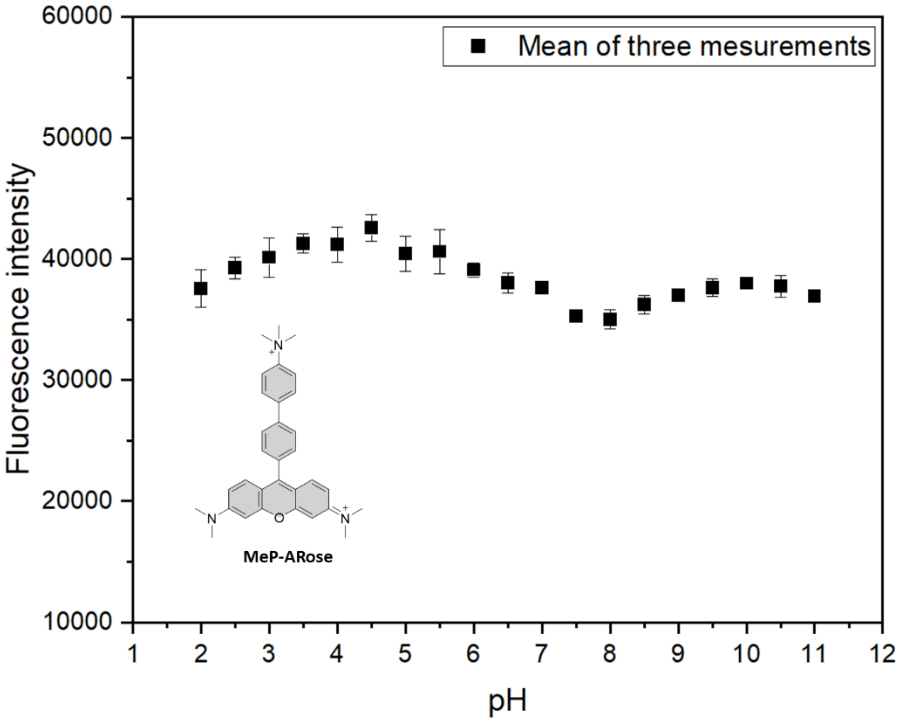

Figure S19. The mean of three measurements of **MeP-ARose** titration. The error bars represent the standard deviation.

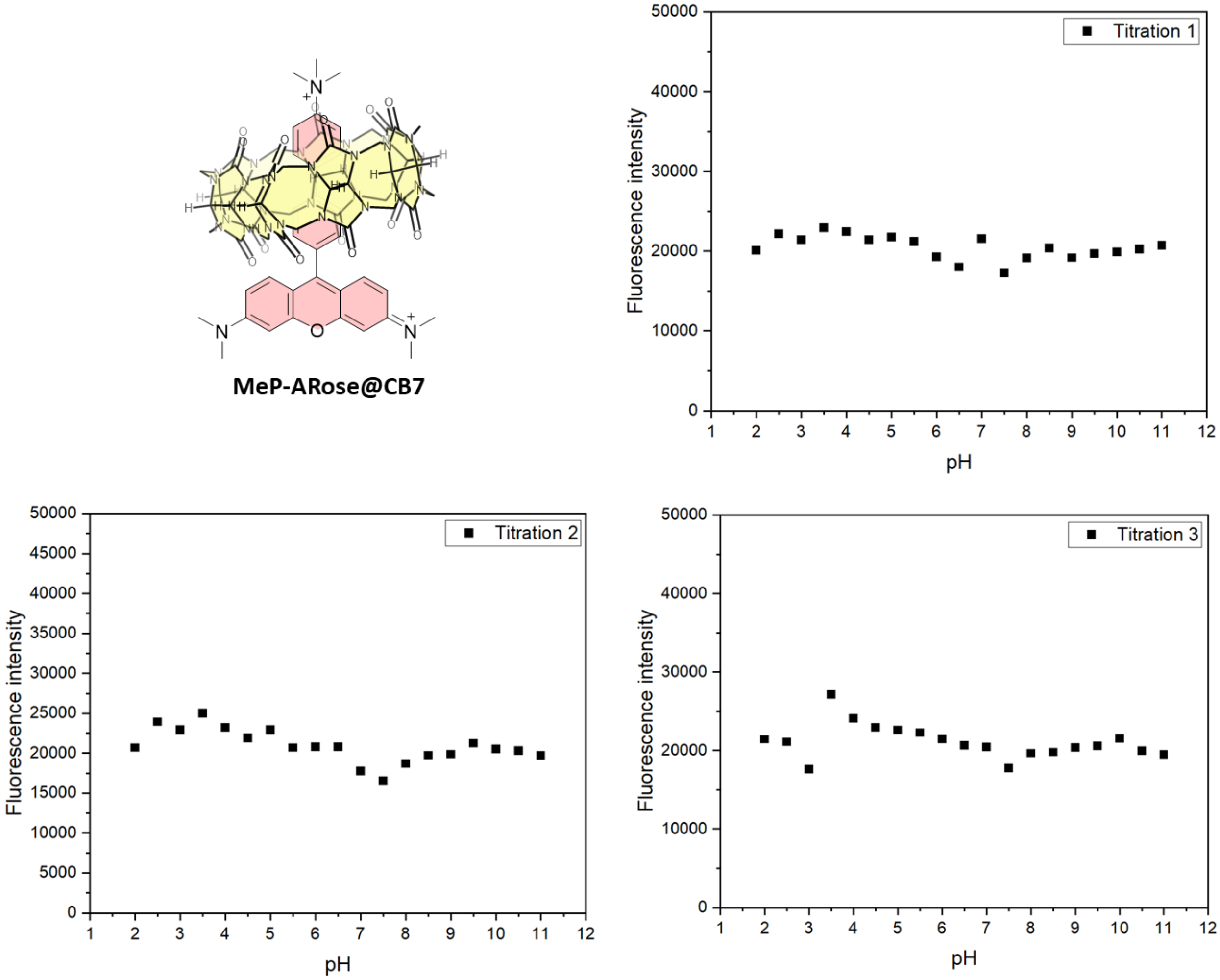

Figure S20. Titration curves of three technical replicates of **MeP-ARose@CB7** titration.

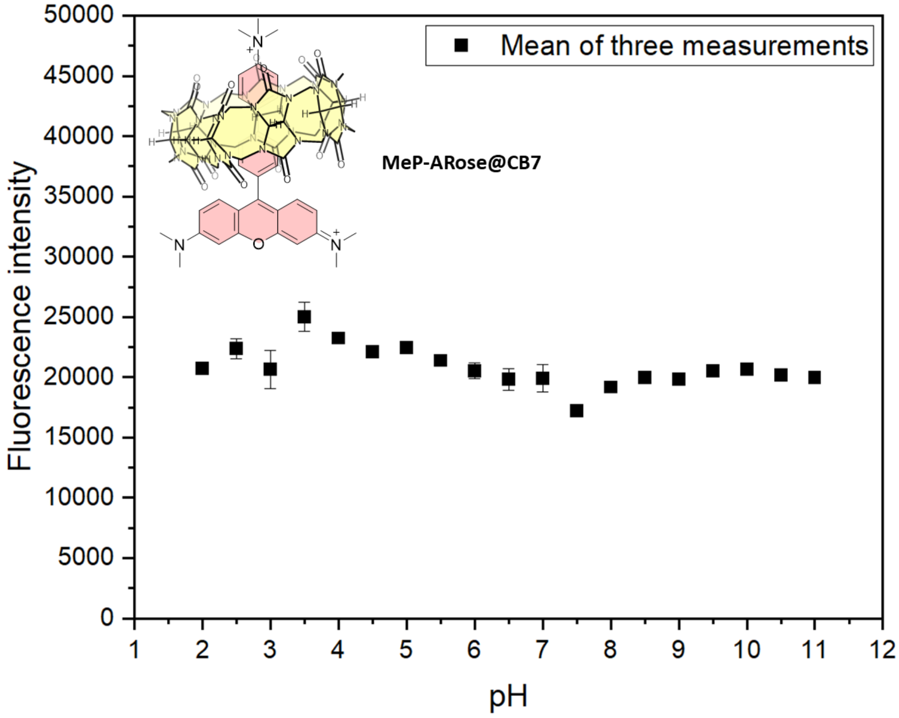

Figure S21. The emission change of **MeP-ARose@CB7** at 550 nm. The reported data points represent the mean of three independent measurements. The error bars represent the standard deviation.

Spectrophotometric titration

Detailed procedure explained on **P-ARose** titration: An aliquot of the **P-ARose** DMSO stock solution (15 µL, 2 mM) was pipetted into water (3 mL) to yield a 10 µM solution containing 0.5% DMSO. To keep the solution homogeneous during the experiment, the stirring in the cuvette holder was turned to a maximum (1200 rpm), and the temperature was kept constant (20 °C). The excitation was executed at 545 nm (slit opening 2.5 nm), while the emission was followed at 560 nm (slit opening 2.5 nm). The experiment was measured in the kinetic mode to follow the diffusion-limited stabilization of the fluorescence before adding the next CB7 portion. Aliquots of CB7 (5 µL, 0.8 mM) were added stepwise, leading to a sharp increase in fluorescence. The amplitude of the signal enhancement dampened with the addition progression, and at the end, an excess of CB7 was added to ensure the completion of titration.

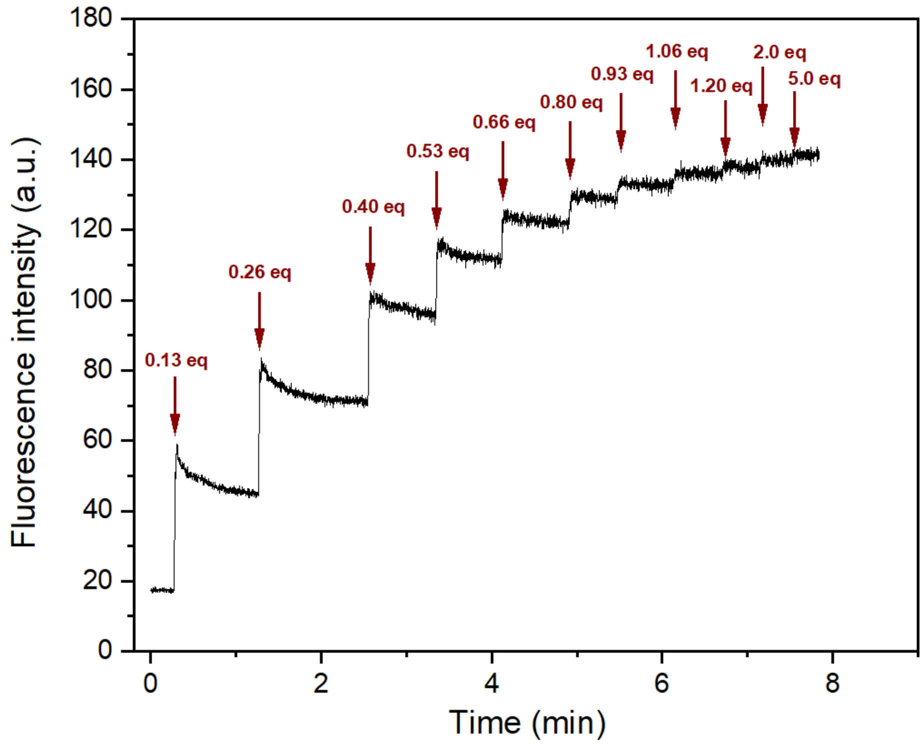

Figure S22. The kinetic measurement of the **P-ARose** titration with CB7 in water (10 µM of the dye, 1% DMSO). The experiment was performed at ambient temperature (20 °C) with maximal stirring (1200 rpm).

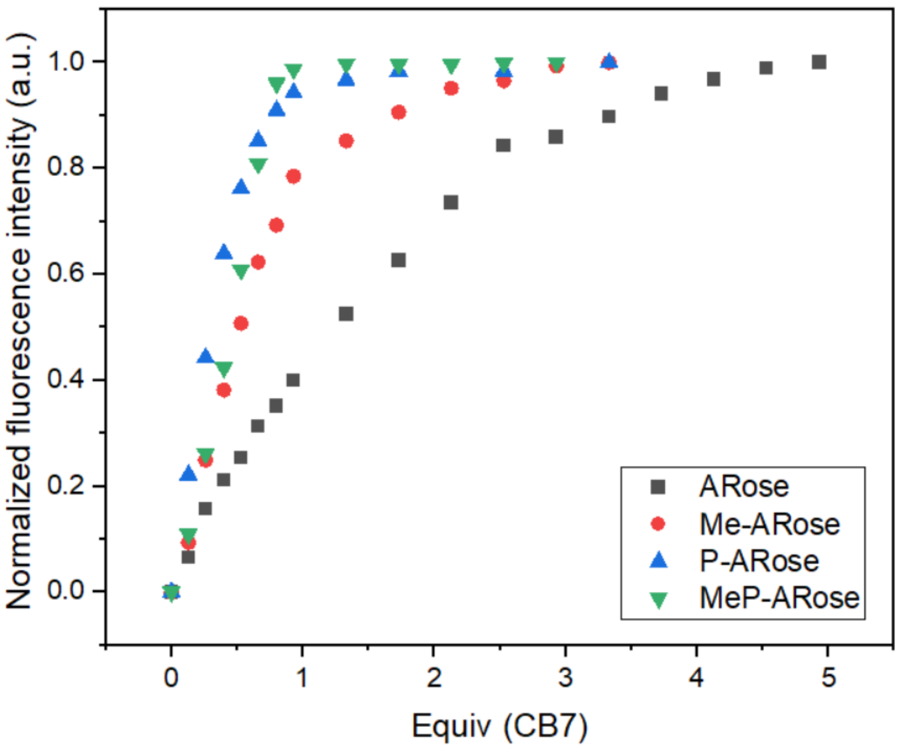

Figure S23. Titration curves of all four compounds with CB7 in water (10 µM of the dye, 1% DMSO). The experiment was performed at ambient temperature (20 °C) with maximal stirring (1200 rpm).

Fluorescence lifetimes

The samples were prepared in water (10 µM, 0.5% DMSO), and the lifetime was measured upon excitation at 470 nm. To ensure complete binding, the lifetime of the host-guest complex was measured in the presence of 10 equivalents of CB7.

Legend:

χ^2 - a statistical measure used to assess the goodness of fit between experimental data and a theoretical model

A – amplitude, represent the relative contribution of each exponential component to the overall decay

ARose

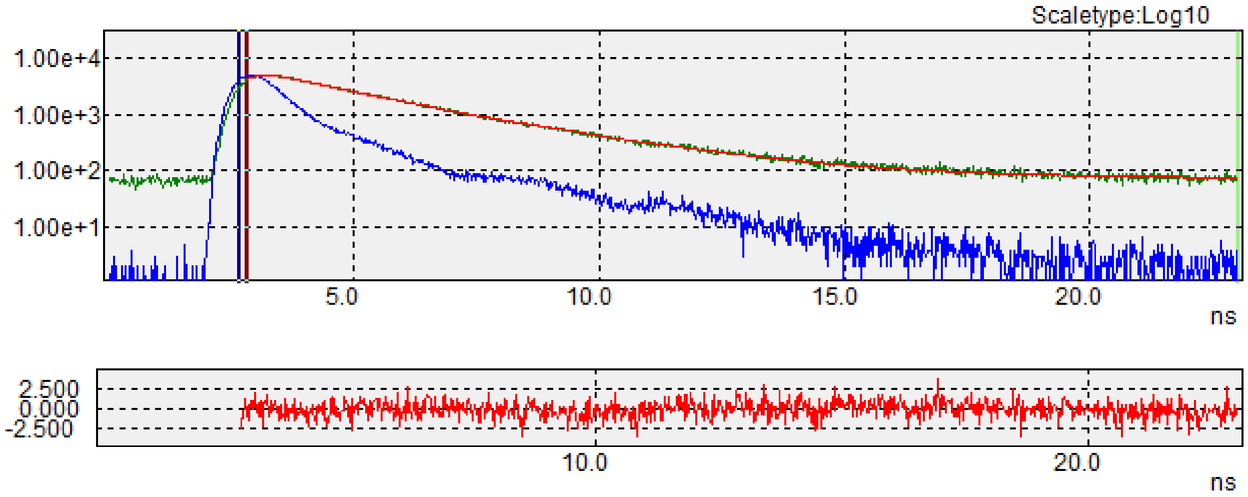

Figure S24. Lifetime decay profiles of **ARose** in water. The excitation wavelength was 470 nm and emission wavelength 560 nm. The green, blue, and red lines represent lifetime decay, instrumental response functions (IRF), and fitted curve (2nd order), respectively. The lower red line represents the residual.

Table S1. A summary of the obtained χ value, fluorescence lifetimes and corresponding amplitudes.

χ^2	τ_{fl}	τ_1	τ_2	A1	A2
1.13	1.78	0.71	2.22	130	99

ARose@CB7

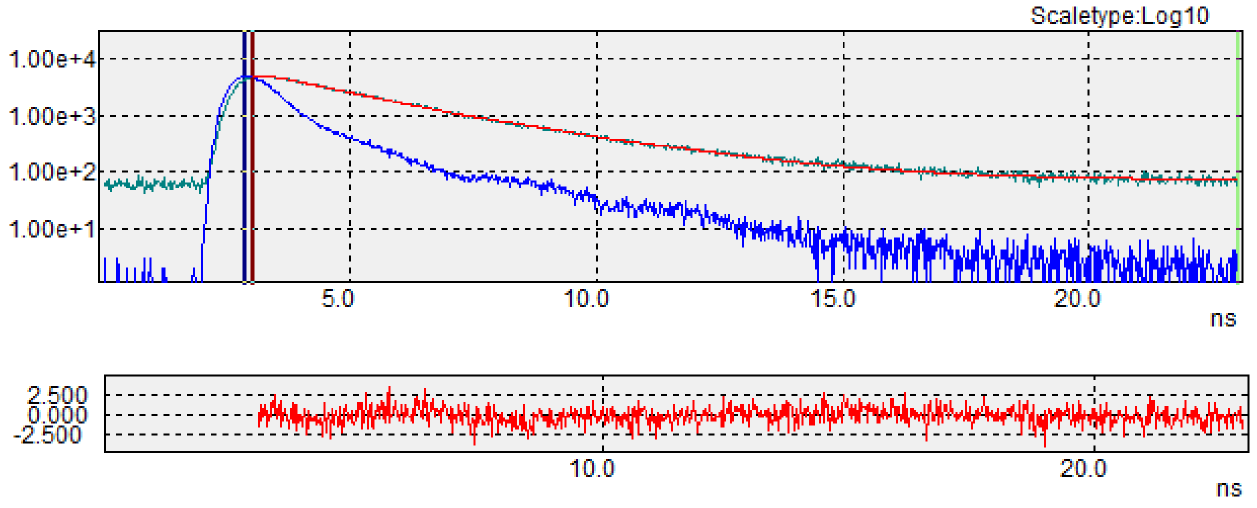

Figure S25. Lifetime decay profiles of **ARose@CB7** in water. The excitation wavelength was 470 nm and emission wavelength 560 nm. The green, blue, and red lines represent lifetime decay, instrumental response functions (IRF), and fitted curve (2nd order), respectively. The lower red line represents the residual.

Table S2. A summary of the obtained χ value, fluorescence lifetimes and corresponding amplitudes.

χ^2	τ_{fl}	τ_1	τ_2	A1	A2
1.13	1.75	0.75	2.28	173	106

P-ARose

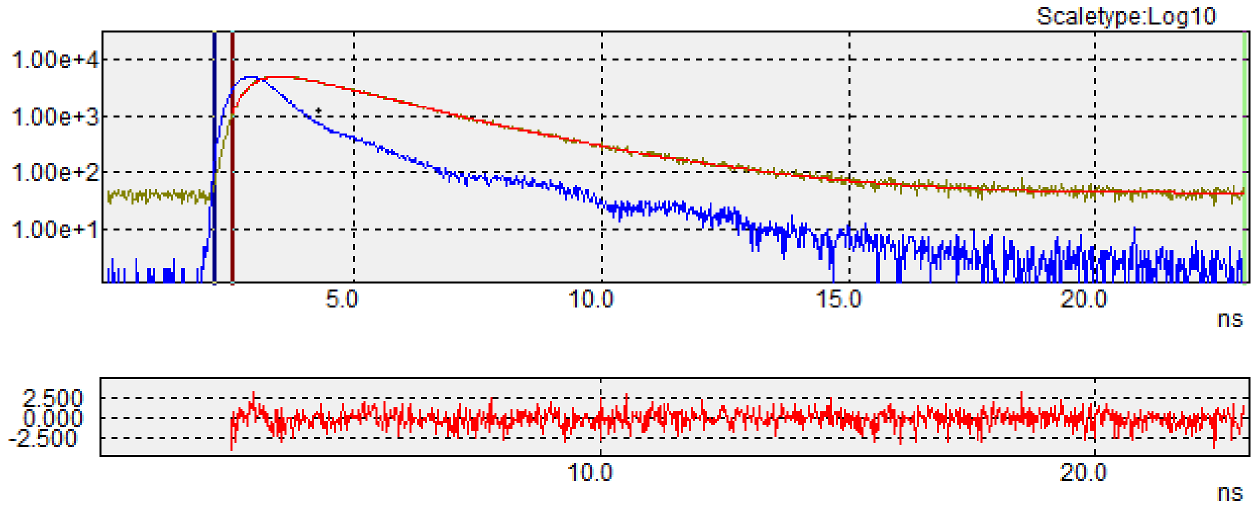

Figure S26. Lifetime decay profiles of **P-ARose** in water. The excitation wavelength was 470 nm and emission wavelength 560 nm. The green, blue, and red lines represent lifetime decay, instrumental response functions (IRF), and fitted curve (2nd order), respectively. The lower red line represents the residual.

Table S3. A summary of the obtained χ value, fluorescence lifetimes and corresponding amplitudes.

χ^2	τ_{fl}	τ_1	τ_2	A1	A2
1.06	1.48	0.69	1.63	61	136

P-ARose@CB7

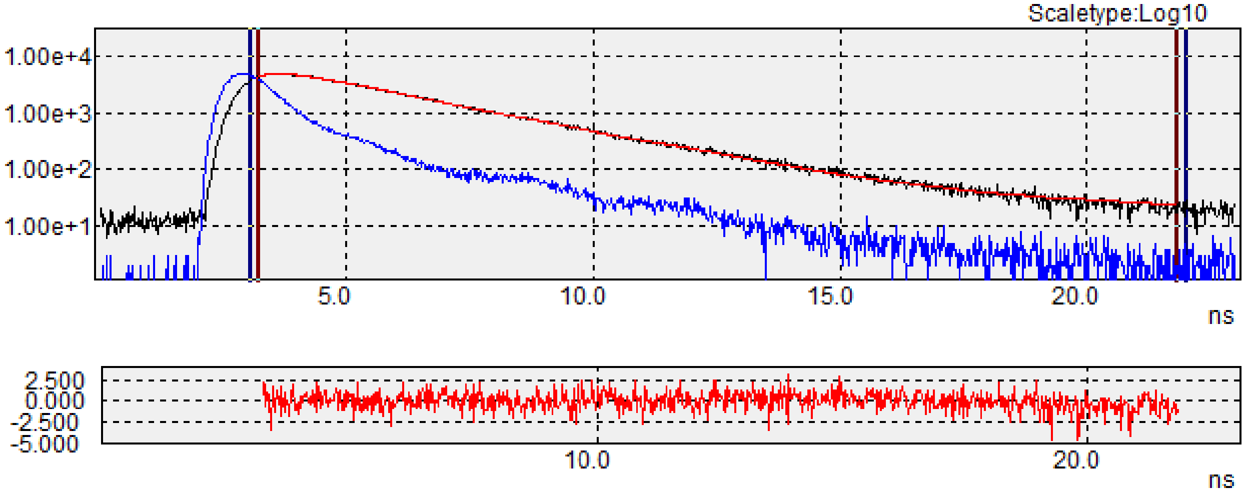

Figure S27. Lifetime decay profiles of **P-ARose@CB7** in water. The excitation wavelength was 470 nm and emission wavelength 560 nm. The green, blue, and red lines represent lifetime decay, instrumental response functions (IRF), and fitted curve (2nd order), respectively. The lower red line represents the residual.

Table S4. A summary of the obtained χ value, fluorescence lifetimes and corresponding amplitudes.

χ^2	τ_{fl}	τ_1	τ_2	A1	A2
1.18	1.69	0.62	1.85	106	238

Me-ARose

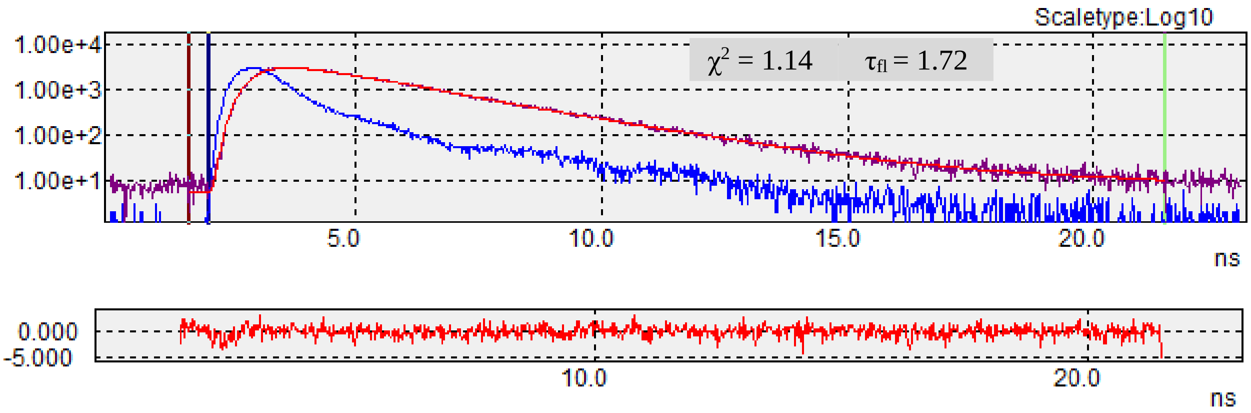

Figure S28. Lifetime decay profiles of **Me-ARose** in water. The excitation wavelength was 470 nm and emission wavelength 560 nm. The green, blue, and red lines represent lifetime decay, instrumental response functions (IRF), and fitted curve (1st order), respectively. The lower red line represents the residual. Values for χ and τ are also given.

Me-ARose@CB7

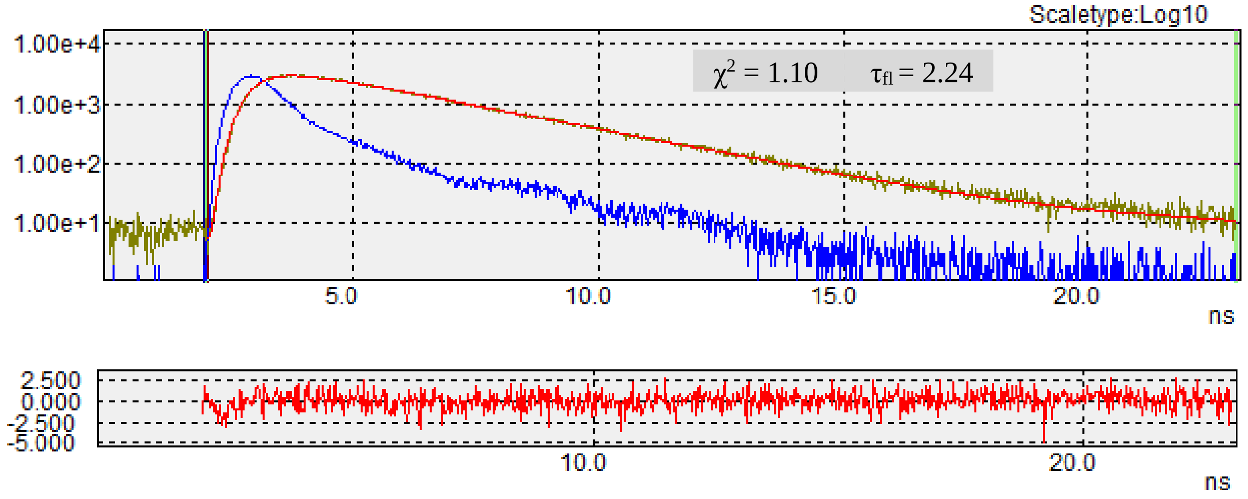

Figure S29. Lifetime decay profiles of **Me-ARose@CB7** in water. The excitation wavelength was 470 nm and emission wavelength 560 nm. The green, blue, and red lines represent lifetime decay, instrumental response functions (IRF), and fitted curve (1st order), respectively. The lower red line represents the residual. Values for χ and τ are also given.

MeP-ARose

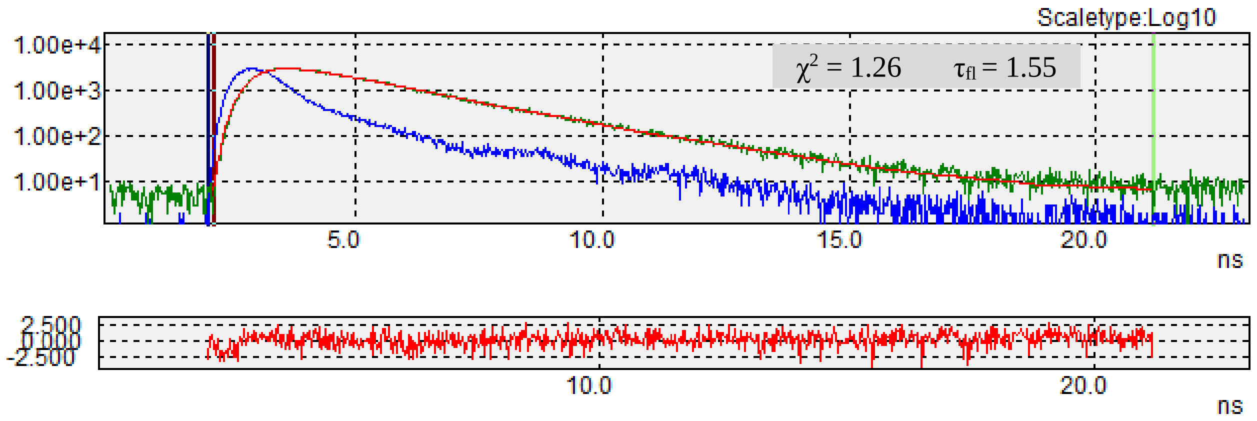

Figure S30. Lifetime decay profiles of **MeP-ARose** in water. The excitation wavelength was 470 nm and emission wavelength 560 nm. The green, blue, and red lines represent lifetime decay, instrumental response functions (IRF), and fitted curve (1st order), respectively. The lower red line represents the residual. Values for χ and τ are also given.

MeP-ARose@CB7

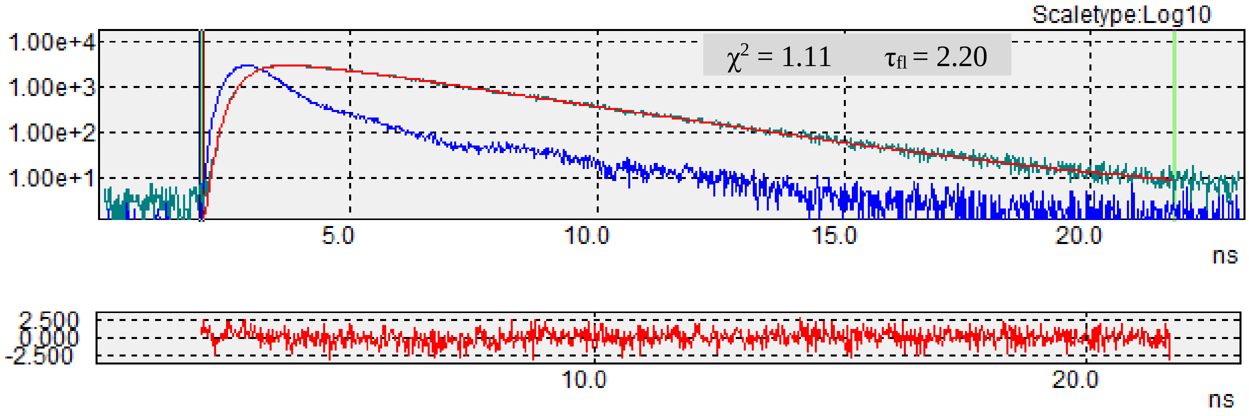

Figure S31. Lifetime decay profiles of **MeP-ARose@CB7** in water. The excitation wavelength was 470 nm and emission wavelength 560 nm. The green, blue, and red lines represent lifetime decay, instrumental response functions (IRF), and fitted curve (1st order), respectively. The lower red line represents the residual. Values for χ and τ are also given.

Binding study: ITC

Instrument control titration

The buffer check ensures that no significant enthalpy signals emerge from assay buffer mixing. Generally, concentrations of background solutes in the ITC cell and ITC syringe should match precisely, to avoid dilution heats masking binding signals. Furthermore, high viscosity and low surface tension can impact cell loading and DP baseline propagation (e.g., by causing foaming or bubble formation).

Measurement setup:

Target (cell): mQ-H2O, 1% DMSO
Ligand (syringe): mQ-H2O, 1% DMSO
Instrument: Malvern PEAQ-ITC
Injections: 13 (0.4 µL + 12 x 3.0 µL)
Ref-power: 10 µcal/s
Stirring: 750 rpm
Temperature: 25°C

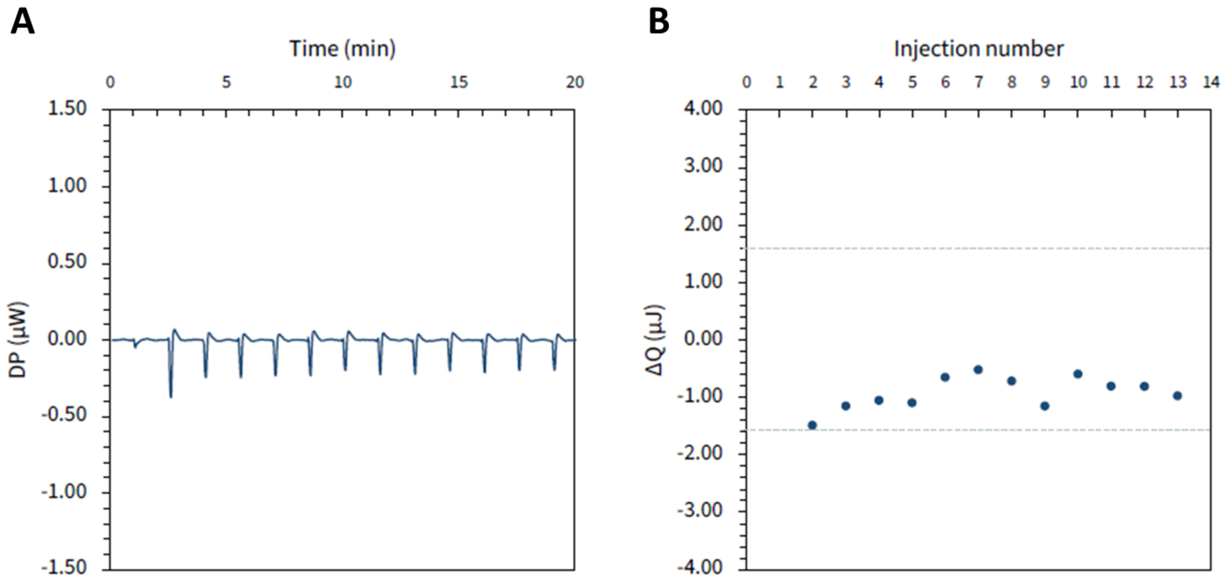

Figure 32. (**A**) Raw heat plot, baseline corrected and (**B**) an integrated heat plot.

Result of the control titration: A constant DP injection amplitude and a steady DP baseline were observed, confirming applicability of the assay buffer.

Injection heats, resulting from integration of the DP peaks, indicate a median ΔQ below |1.6| µJ, thus complying with the ideal instrument specifications. ΔQ variance was in a typical range for buffer-buffer injections.

ITC analysis

The affinity between dye and CB7 was measured by ITC using a MicroCal PEAQ-ITC instrument (Malvern). All titrations were performed at 25 °C and in pure water. Stock solution of the dye was prepared in DMSO (4 mM) while the stock solution of CB7 was made in water (1 mM) and incubated at room temperature until fully dissolved. For the titration, a stock solution of the dye was diluted in water (1% DMSO, 40 µM). The dye solution (40 µM) was titrated with 19 injections of 2 µL to CB7 (500 µM) having an injection spacing of 150 seconds. As reference run, a titration of dye into pure water was performed. The obtained thermogram was baseline corrected, and the binding signals were corrected for the control measurement. The analysis was conducted using the software supplied by the manufacturer.

ARose

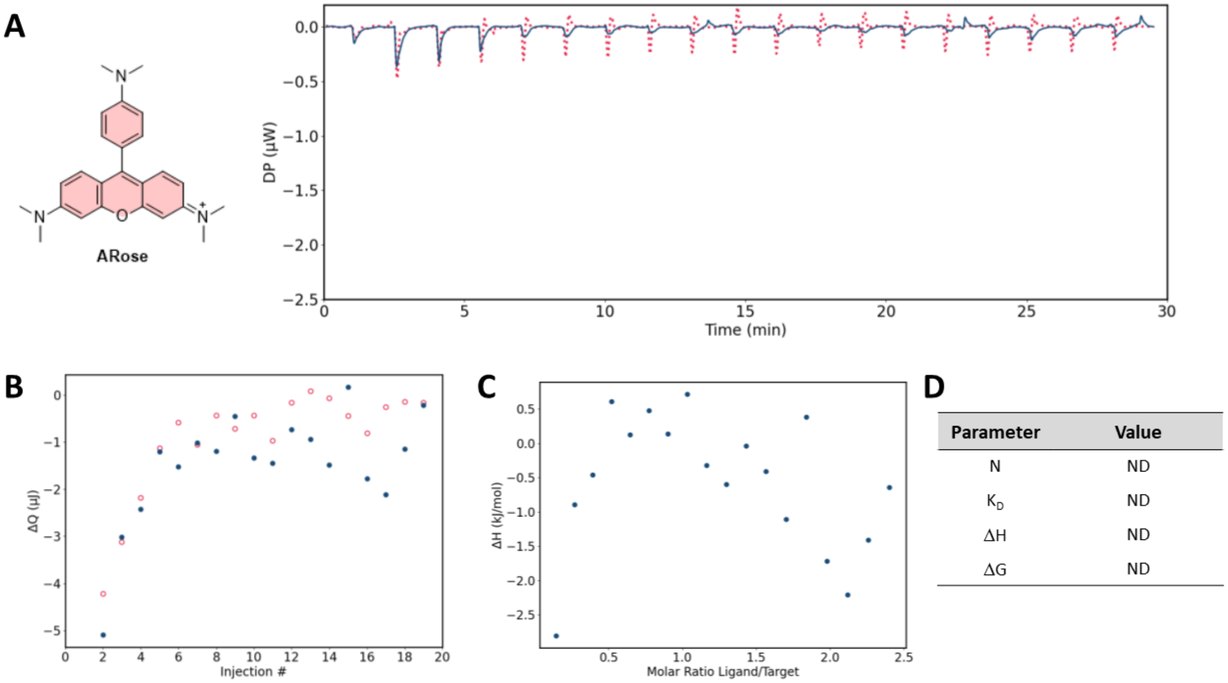

Figure S33. (**A**) The **DP signal plot** shows no significant development of injection peaks. DP signals of ligand-target titration and ligand-buffer titration have a very similar profile, with similar peak sizes. (**B**) The **Raw Heat plot,** obtained from integration of the DP signals, indicates no target-specific heat release. (**C**) The **Normalized heat plot** shows no clear trend towards zero enthalpy with increasing molar ratio Ligand/Target. (**D**) No fit could be applied, so the corresponding parameters were not determined.

Me-ARose

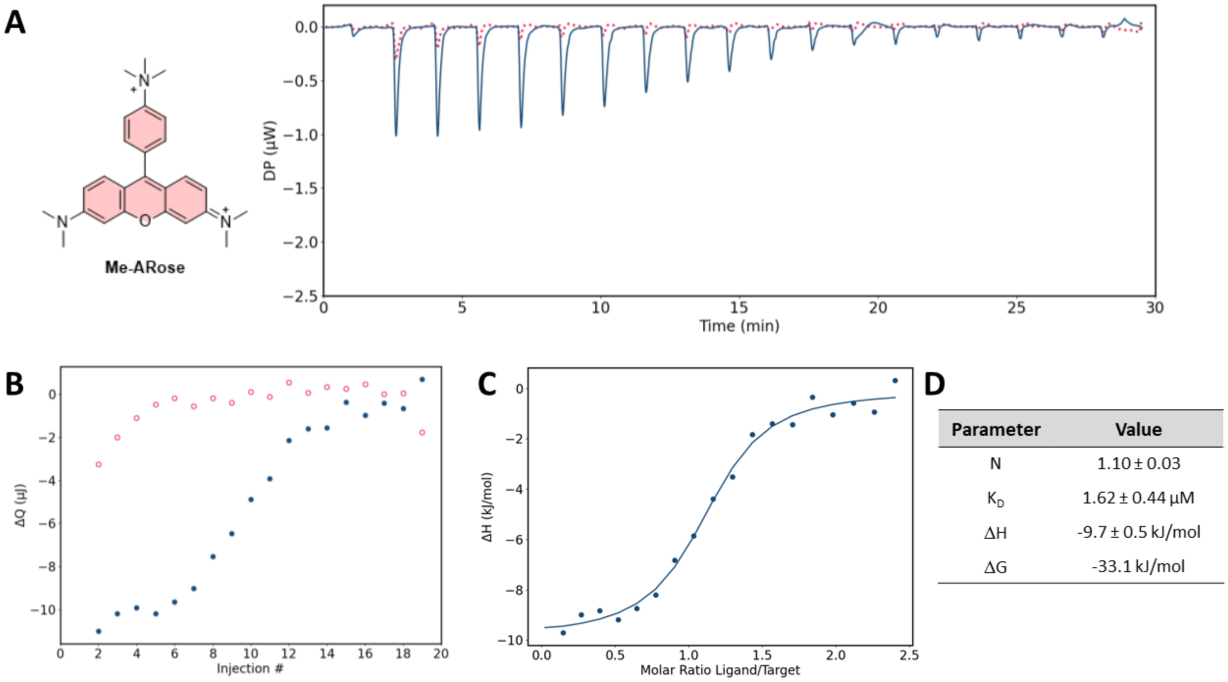

D

Parameter	Value
N	1.10 ± 0.03
K_D	1.62 ± 0.44 μM
ΔH	-9.7 ± 0.5 kJ/mol
ΔG	-33.1 kJ/mol

Figure S34. (**A**) The **DP signal plot** shows a saturating trend for the ligand-target titration, and a flat signal for the ligand-buffer titration. (**B**) The **Raw Heat plot,** obtained from integration of the DP signals, indicates target-specific heart release. (**C**) The **Normalized heat plot** shows a clear trend towards zero enthalpy with increasing molar ratio Ligand/Target and thus indicates interaction. The data distribution is sigmoidal, implying that the fit can accurately determine fit parameters. A fit model considering one set of binding sites was applied. (**D**) The obtained parameters.

MeP-ARose

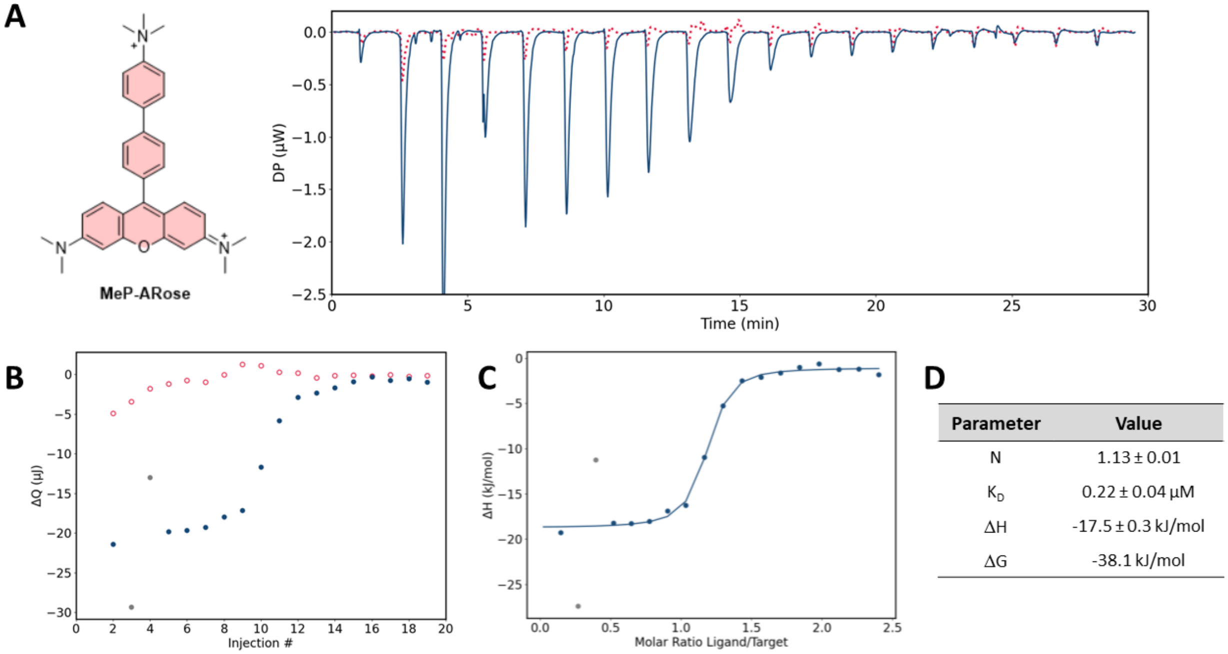

D

Parameter	Value
N	1.13 ± 0.01
K_D	0.22 ± 0.04 µM
ΔH	-17.5 ± 0.3 kJ/mol
ΔG	-38.1 kJ/mol

Figure S35. (**A**) The **DP signal plot** shows a saturating trend for the ligand-target titration, and a flat signal for the ligand-buffer titration. *Second and third 2 µL injection peak seem to be outliers (possibly affected by an air bubble artifact).* (**B**) The **Raw Heat plot,** obtained from integration of the DP signals, indicates target-specific heart release. (**C**) The **Normalized heat plot** shows a clear trend towards zero enthalpy with increasing molar ratio Ligand/Target and thus indicates interaction. The data distribution is sigmoidal, implying that the fit can accurately determine fit parameters. A fit model considering one set of binding sites was applied. Two outlier data points were excluded from the fit. (**D**) The obtained parameters.

P-ARose

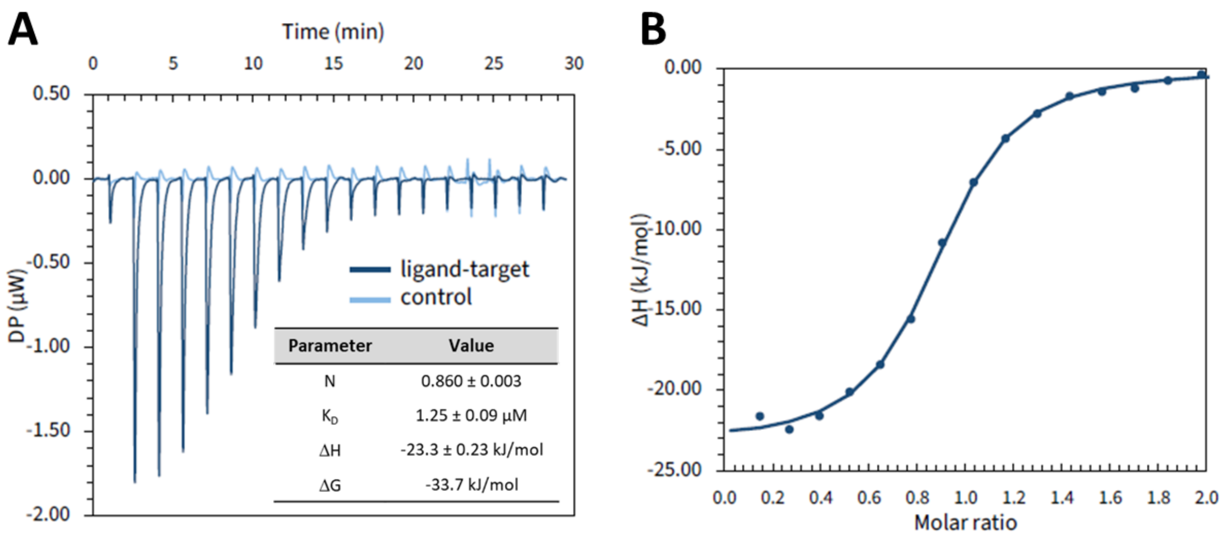

Figure S36. (**A**) Raw heat plots, baseline corrected and (**B**) integrated heat plot, control subtracted.

Solubility test

Due to the often-required high concentration of the dye, a solubility test in pure water was performed. The dye was dissolved at 40 μM concentration (1% DMSO) and the absorption spectra measured every 5 minutes for more than 2 hours (135 minutes). The absorption changed insignificantly indicating a good solubility at this concentration (Figure S31).

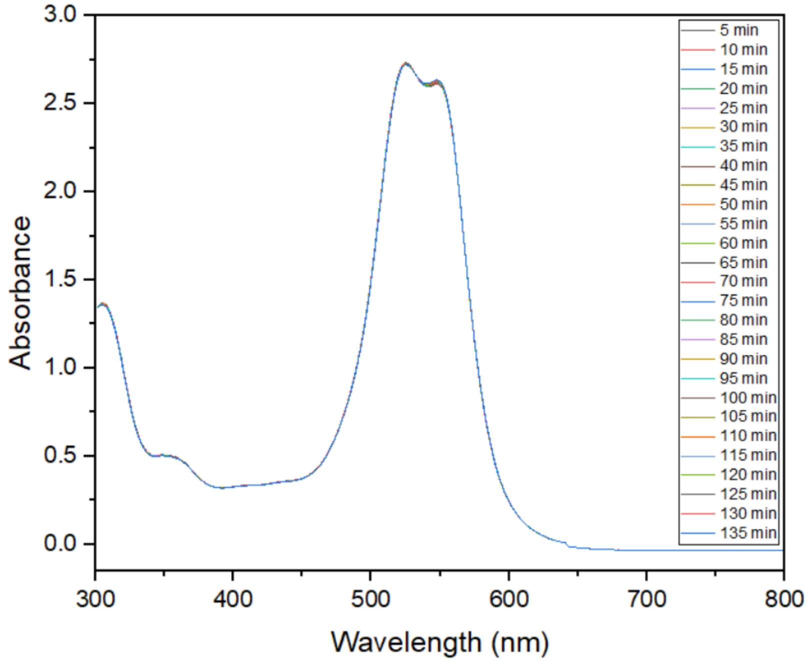

Figure S37. The solubility test of **P-ARose** at the concertation applied in ITC experiment (1% DMSO, 40 μM). The absorption spectra were collected every 5 minutes over 135 minutes.

NMR analysis in aqueous solution

ARose and **P-ARose** solution was prepared as saturated in D_2O (the concentration was not determined, but according to the NMR signal intensity it is safe to assume to be significantly lower than mM). A complete complexation was achieved by adding CB7 until the solution was saturated. *Note*: It was observed that CB7 increases the solubility of the dye. Thus, the effective concentration of **ARose** and **P-ARose** in the presence of CB7 is higher compared to the original dye solution.

Chemical shifts of **ARose**:

1H NMR (800 MHz, D_2O) δ 7.07 – 6.97 (m, 2H), 6.97 – 6.90 (m, 2H), 6.84 – 6.76 (m, 2H), 6.45 – 6.39 (m, 2H), 6.08 – 6.00 (m, 2H), 3.07 (s, 6H), 2.91 (s, 12H).

Chemical shifts of **ARose@CB7**:

^{1}H NMR (950 MHz, D_2O) δ 7.95 (br s, 2H), 7.52 (br s, 2H), 6.89 (br s, 2H), 6.55 (br s, 4H), 3.07 (s, 12H), 2.58 (br s, 6H).

Chemical shifts of **P-ARose**:

^{1}H NMR (800 MHz, D_2O) δ 7.92 (d, *J* = 8.7 Hz, 2H), 7.89 (d, *J* = 8.3 Hz, 2H), 7.67 (d, *J* = 8.7 Hz, 2H), 7.53 (d, *J* = 8.3 Hz, 2H), 7.34 (d, *J* = 10.0 Hz, 2H), 6.86 (dd, *J* = 10.0, 2.3 Hz, 2H), 6.69 (d, *J* = 2.3 Hz, 2H), 3.29 (s, 6H), 3.13 (s, 12H).

Chemical shifts of **P-ARose@CB7**:

^{1}H NMR (800 MHz, D_2O) δ 8.09 (d, J = 10.2 Hz, 2H), 7.52 (d, *J* = 7.5 Hz, 2H), 7.13 (d, *J* = 7.5 Hz, 2H), 7.02 (m, 4H), 6.89 (d, J = 8.0 Hz, 2H), 6.72 (bs, 2H), 3.29 (s, 6H), 3.16 (s, 12H).

ARose

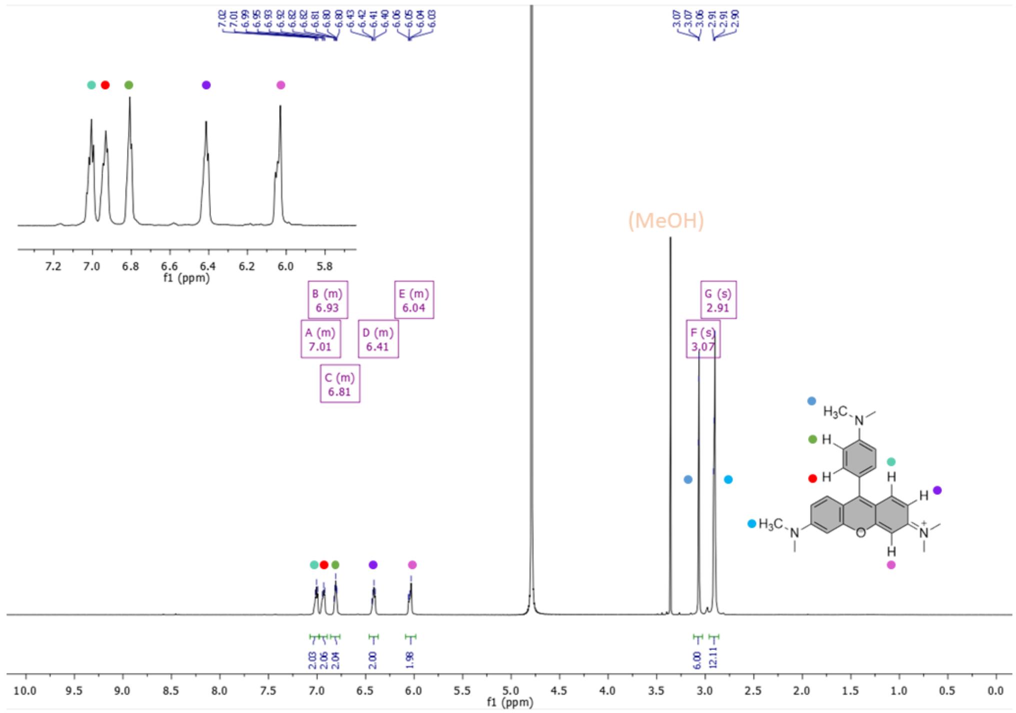

Figure S38. 1D ^{1}H NMR experiment in D_2O (800 MHz). Number of scans: 24.

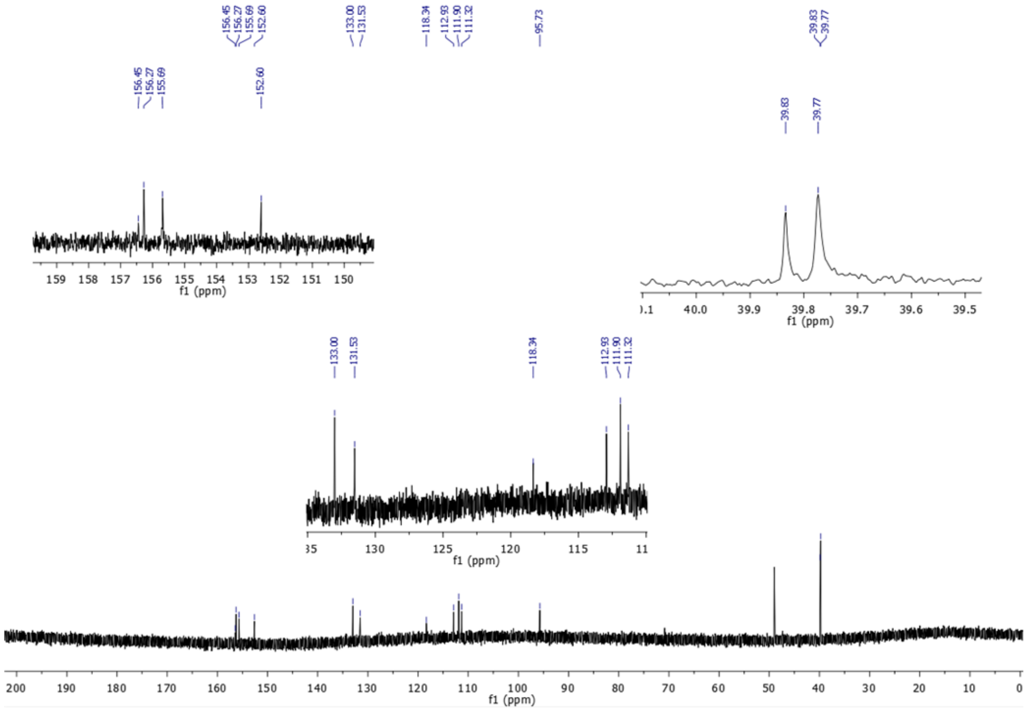

Figure S39. ^{13}C {^{1}H} NMR experiment in D_2O (200 MHz). Number of scans: 3200.

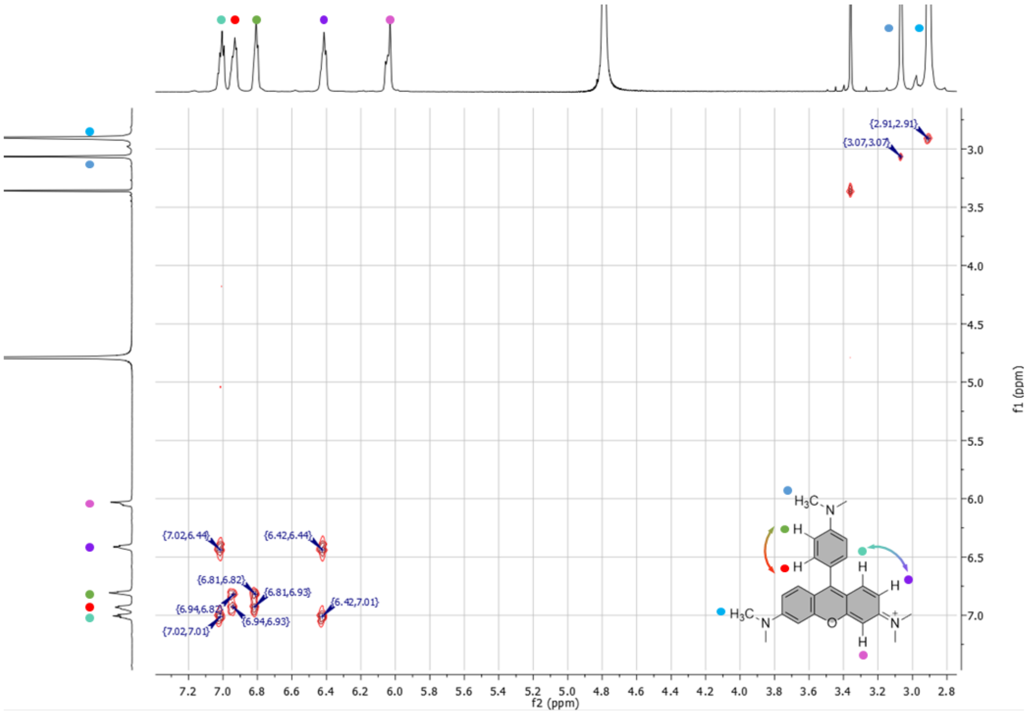

Figure S40. ^{1}H-^{1}H COSY NMR experiment (*cosydfgpph19*) in D_2O (800 MHz). Number of scans 8. Solvent signal was suppressed using watergate.

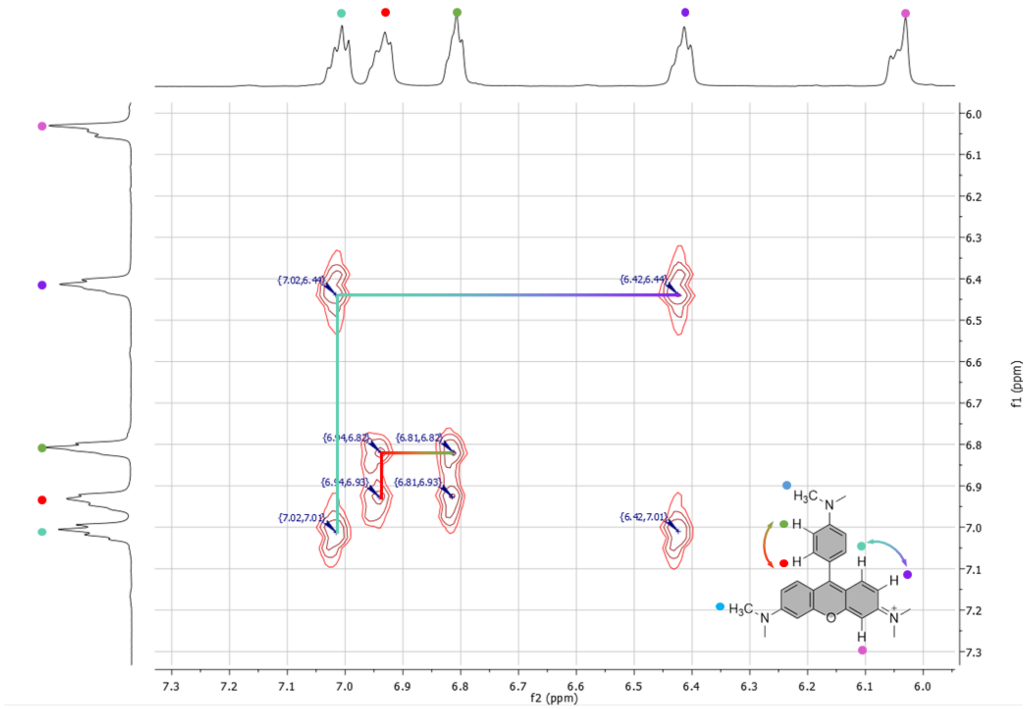

Figure S41. ^{1}H-^{1}H COSY NMR experiment. The aromatic area is enlarged. The color coding is used to indicate specific correlations.

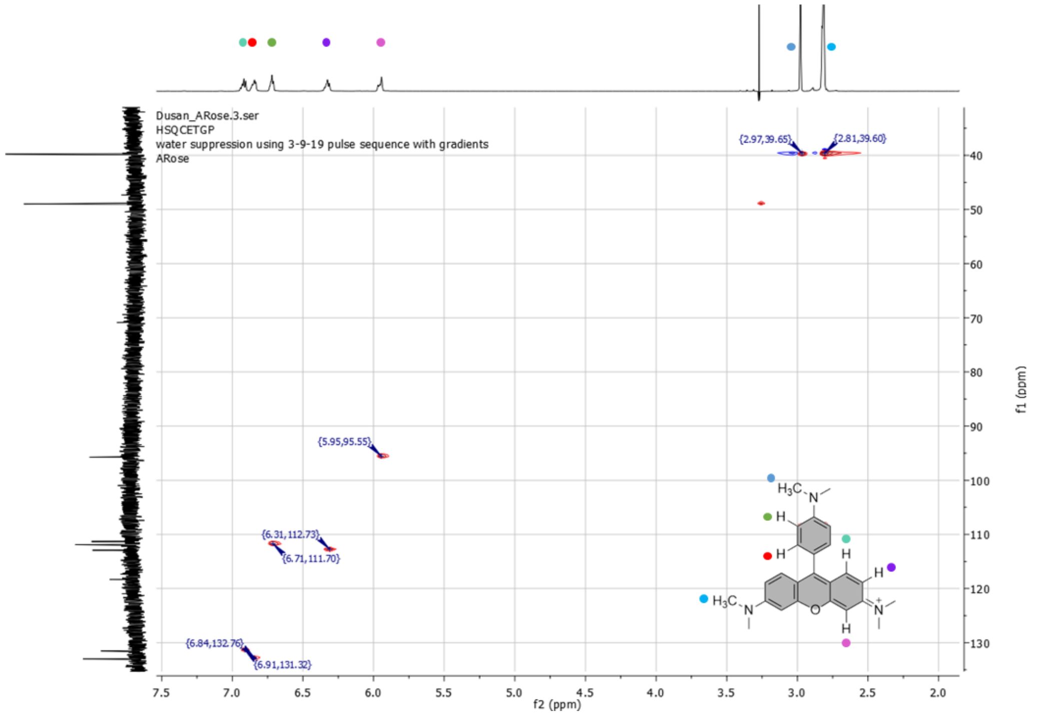

Figure S42. ^{1}H-^{13}C HSQC NMR experiment (*hsqcf3gpph19*) in D_2O (800 MHz). Number of scans 16. Solvent signal was suppressed using watergate.

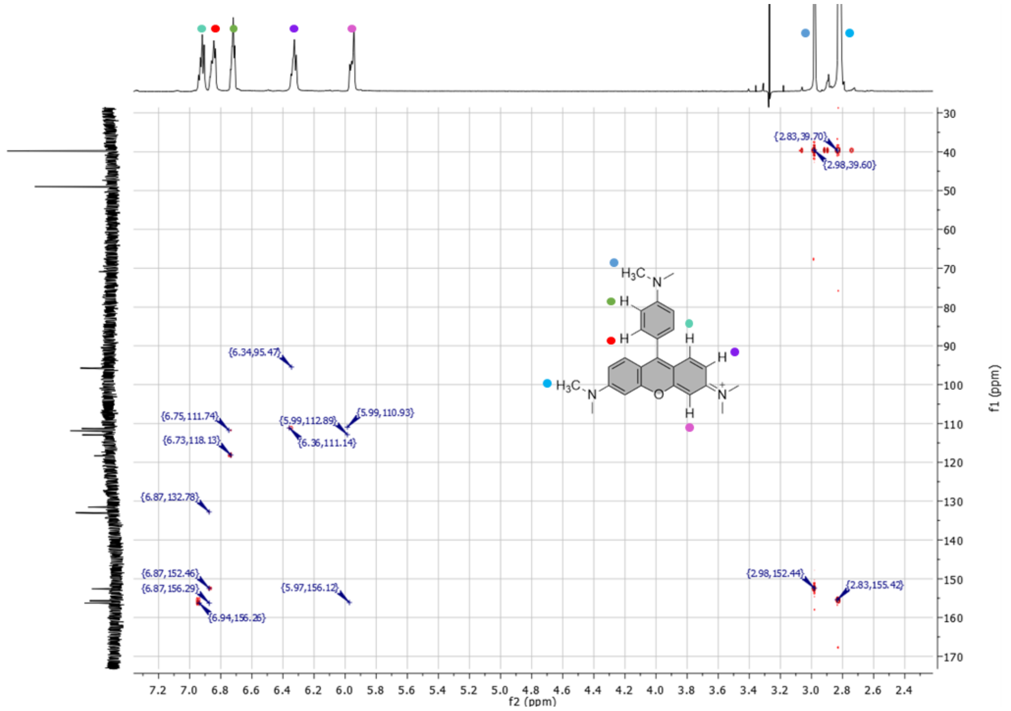

Figure S43. ^{1}H-^{13}C HMBC NMR experiment (*hmbcgpl2ndwg*) in D_2O. Number of scans 32. Solvent signal was suppressed using watergate.

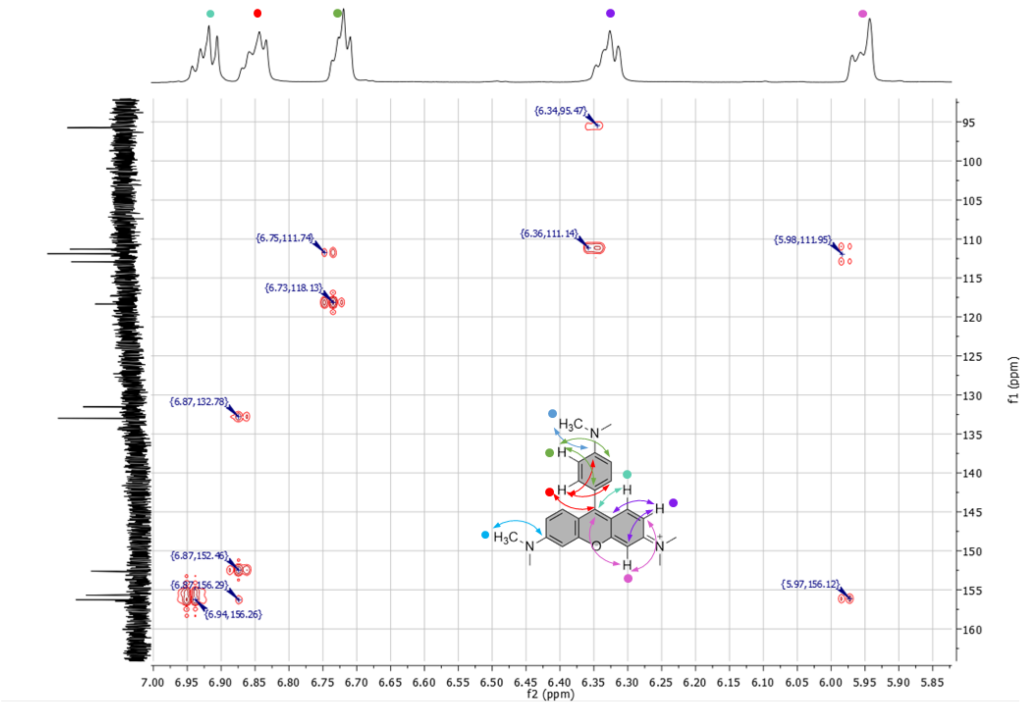

Figure S44. ^{1}H-^{13}C HMBC NMR experiment The aromatic area is enlarged. The color coding is used to indicate specific correlations.

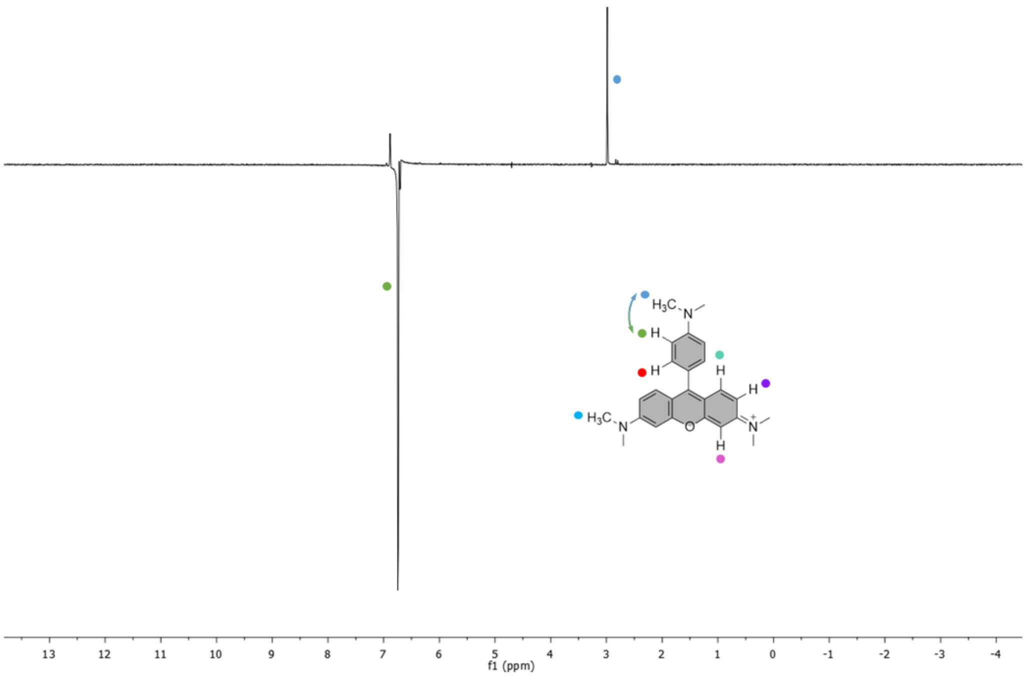

Figure S45. 1D ROESY spectrum of **ARose** in D_2O (*selrogp*/ 800 MHz), with selective irradiation at 6.8173 ppm. Number of scans: 80; mixing time: 190 ms.

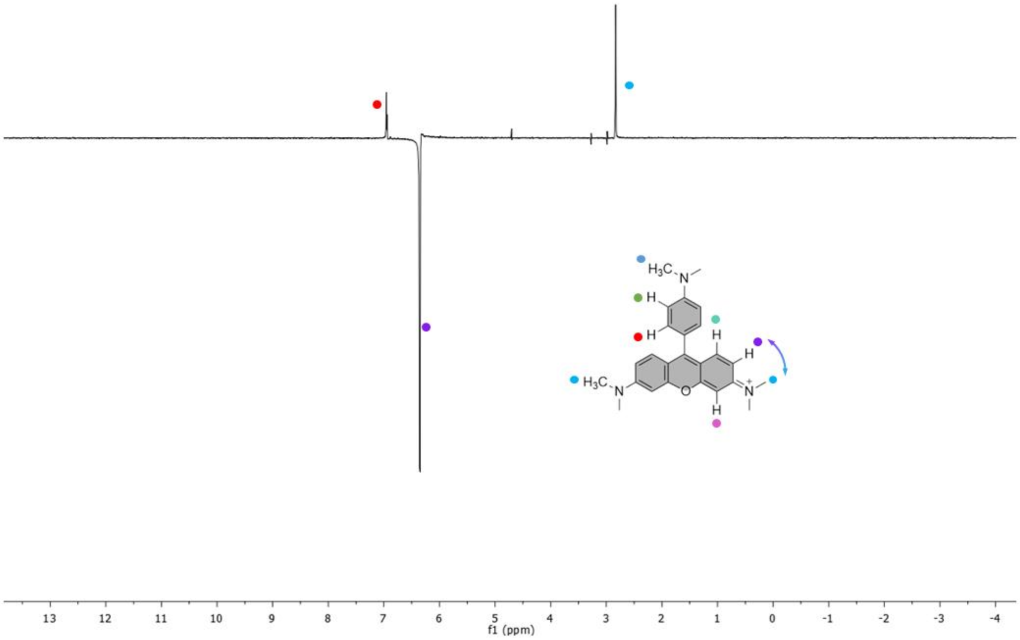

Figure S46. 1D ROESY spectrum of **ARose** in D_2O (*selrogp*/ 800 MHz), with selective irradiation at 6.4254 ppm. Number of scans: 80; mixing time: 190 ms.

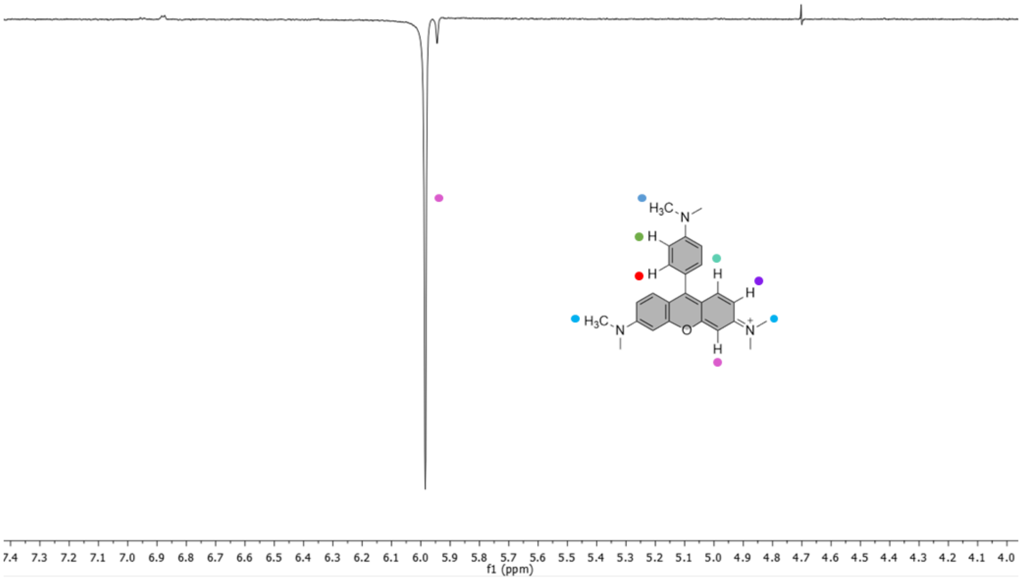

Figure S47. 1D ROESY spectrum of **ARose** in D_2O (*selrogp*/ 800 MHz), with selective irradiation at 6.0544 ppm. Number of scans: 80; mixing time: 190 ms.

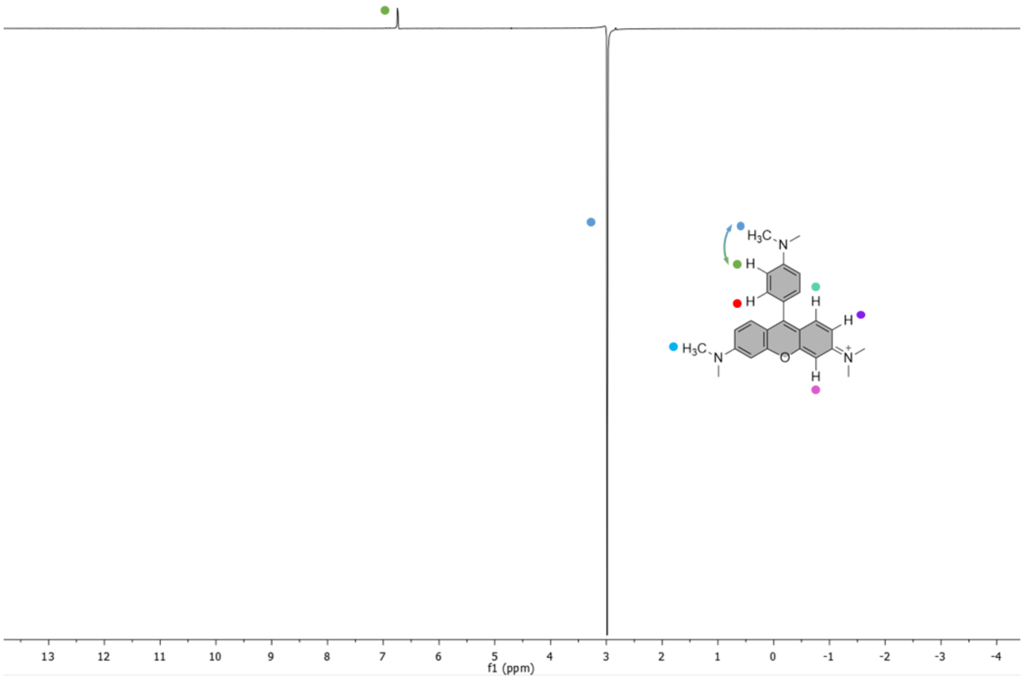

Figure S48. 1D ROESY spectrum of **ARose** in D_2O (*selrogp*/ 800 MHz), with selective irradiation at 3.0762 ppm. Number of scans: 80; mixing time: 190 ms.

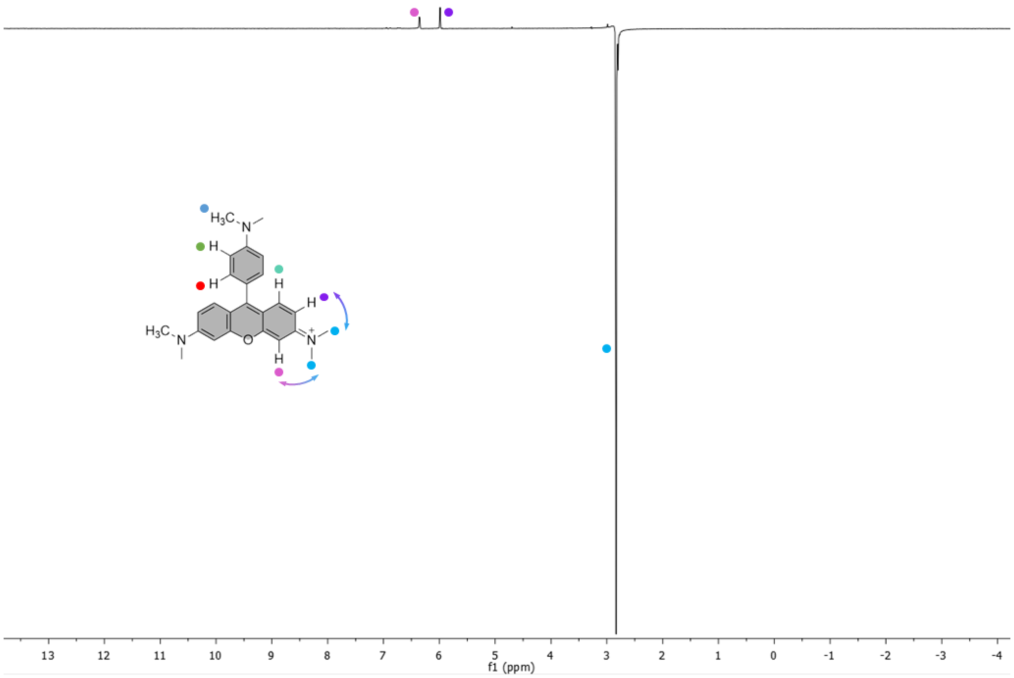

Figure S49. 1D ROESY spectrum of **ARose** in D_2O (*selrogp*/ 800 MHz), with selective irradiation at 2.9169 ppm. Number of scans: 80; mixing time: 190 ms.

ARose@CB7

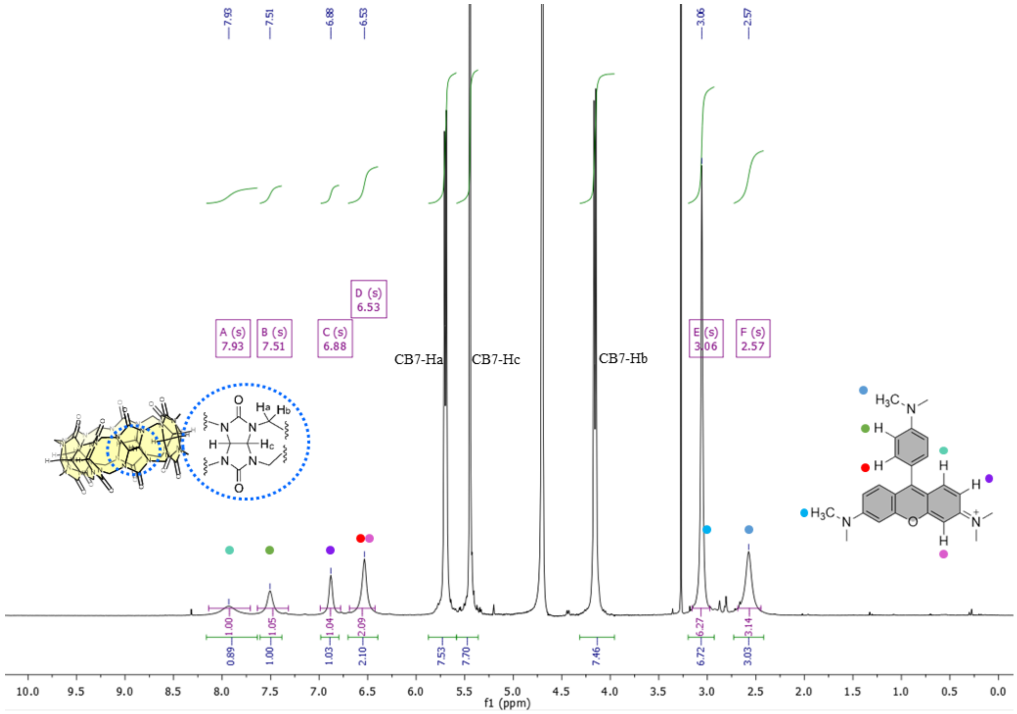

Figure S50. 1D ^{1}H NMR experiment of **ARose** in D_2O (800 MHz). Number of scans: 16.

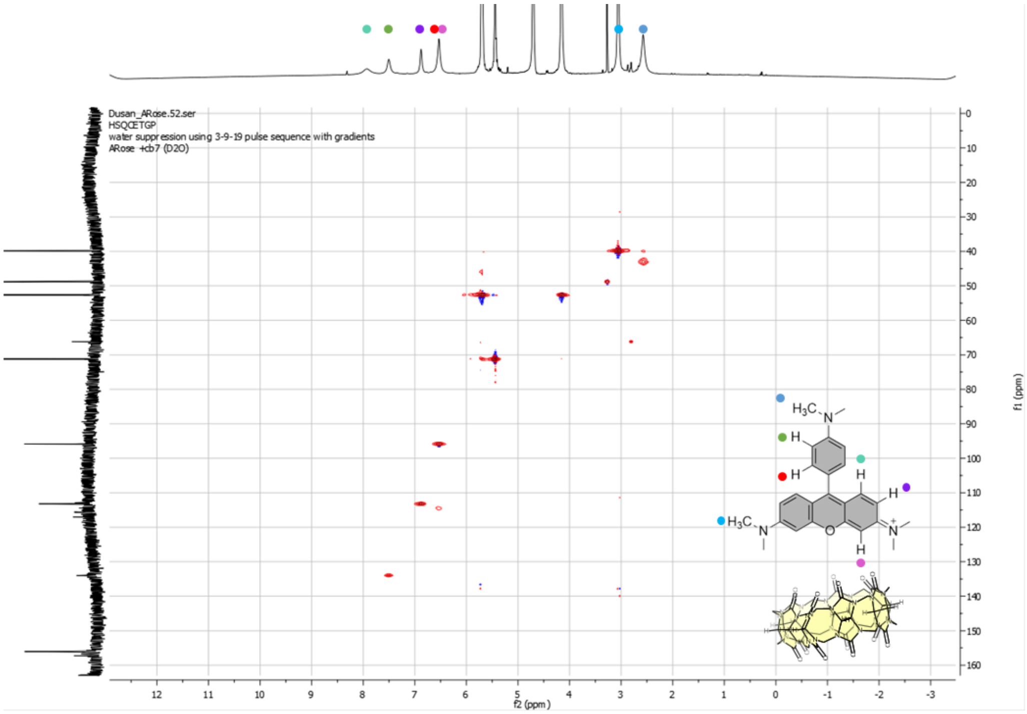

Figure S51. ^{1}H-^{13}C HSQC NMR experiment of **ARose** in D_2O (*hsqcf3gpph19*/800 MHz). Number of scans: 32. Solvent signal was suppressed using watergate.

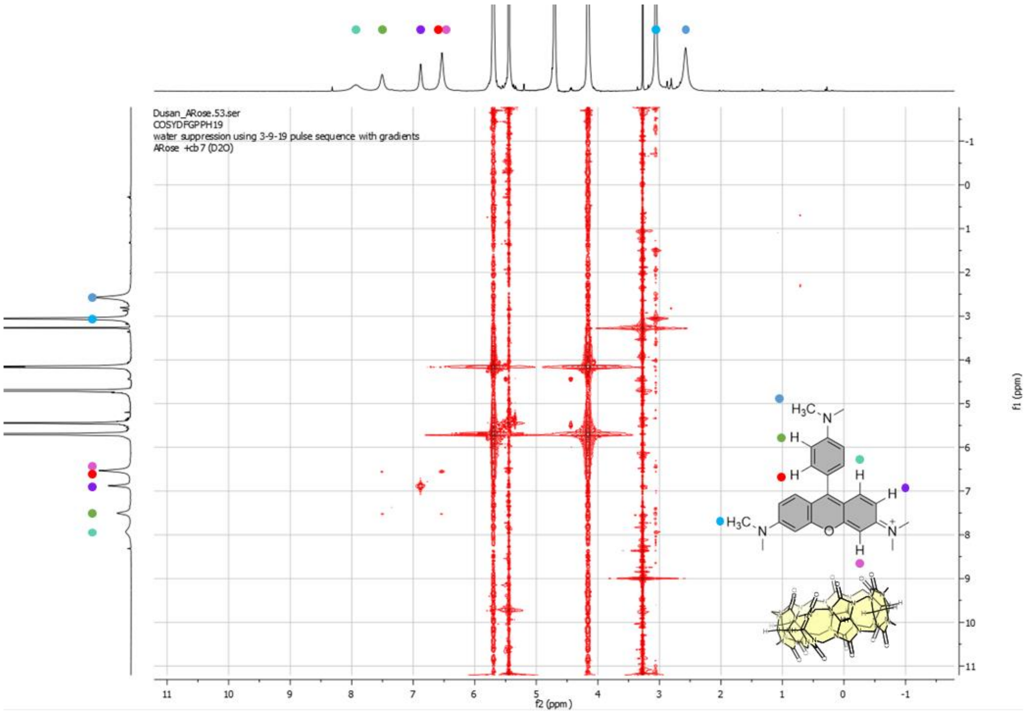

Figure S52. ^{1}H-^{1}H COSY spectrum of **ARose@CB7** in D_2O (800 MHz), using Watergate-based 3-9-19 suppression for water suppression (*cosydfgpph19*). Number of scans: 8.

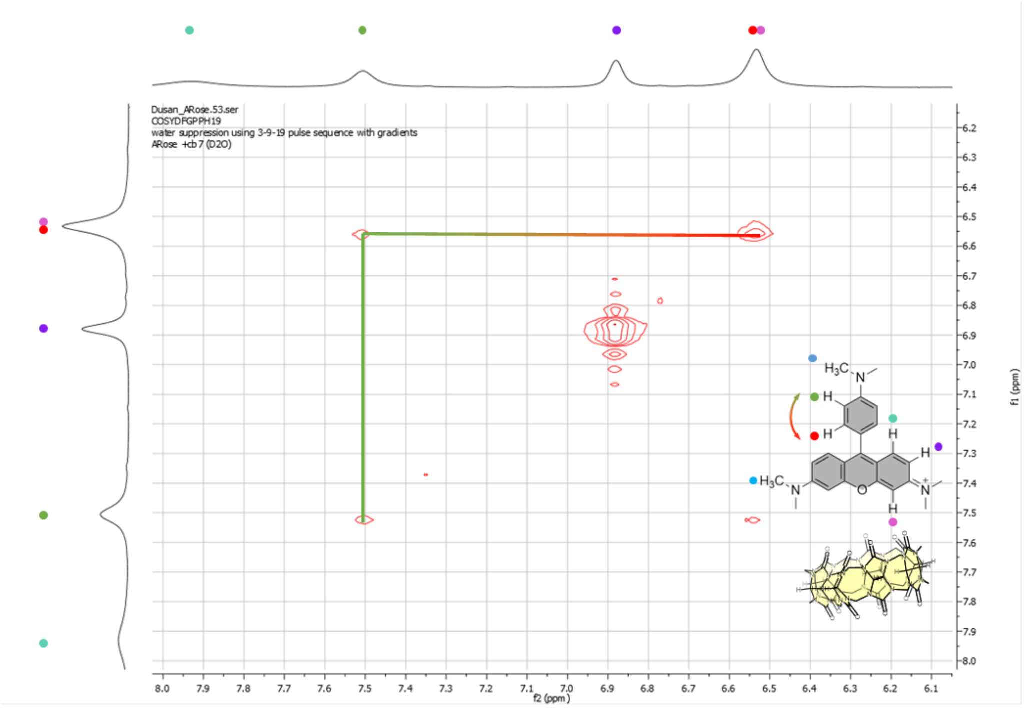

Figure S53. ^{1}H-^{1}H COSY NMR experiment. The aromatic area is enlarged. The color coding is used to indicate specific correlations.

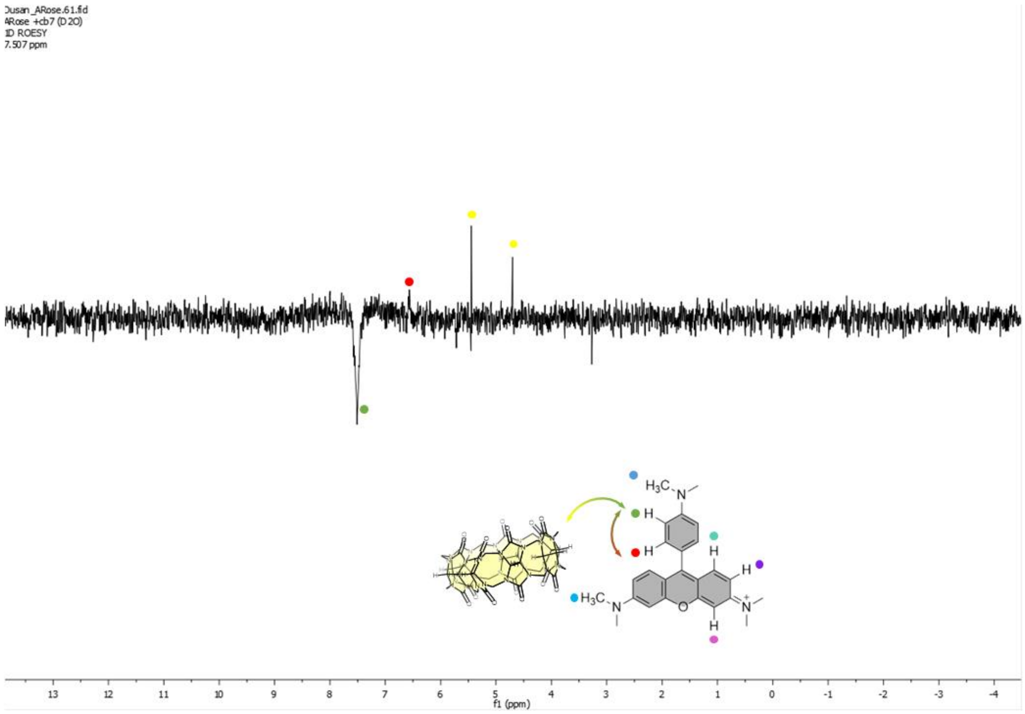

Figure S54. 1D ROESY spectrum of **ARose@CB7** in D_2O (*selrogp*/ 800 MHz), with selective irradiation at 7.60 ppm. Number of scans: 80; mixing time: 190 ms.

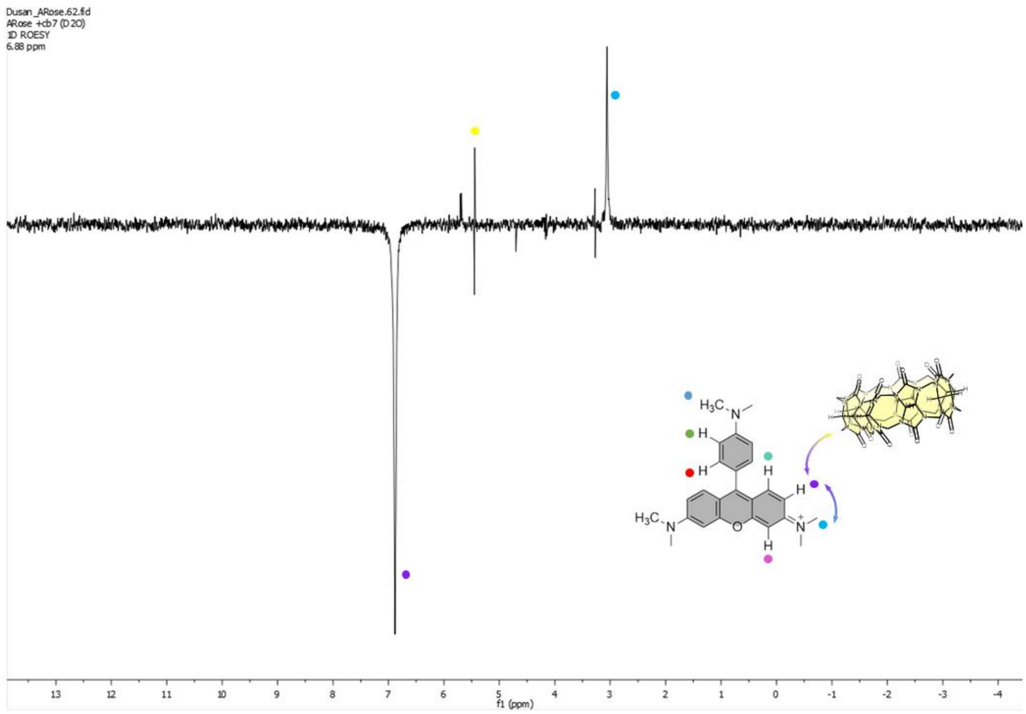

Figure S55. 1D ROESY spectrum of **ARose@CB7** in D_2O (*selrogp*/ 800 MHz), with selective irradiation at 6.97 ppm. Number of scans: 80; mixing time: 190 ms.

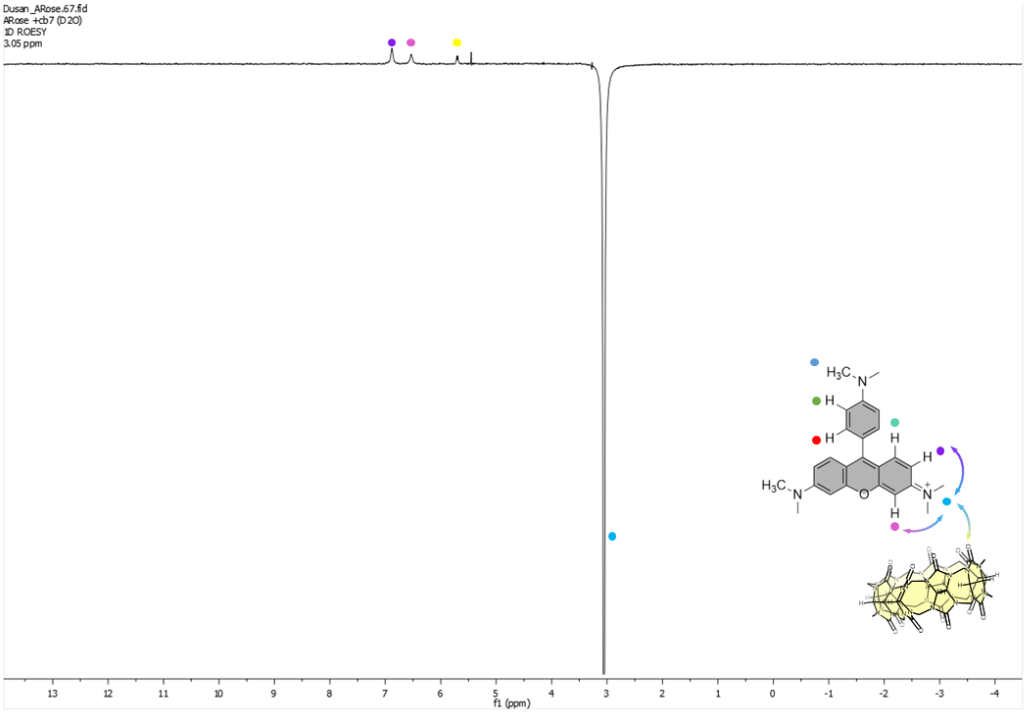

Figure S56. 1D ROESY spectrum of **ARose@CB7** in D_2O (*selrogp*/ 800 MHz), with selective irradiation at 3.15 ppm. Number of scans: 80; mixing time: 190 ms.

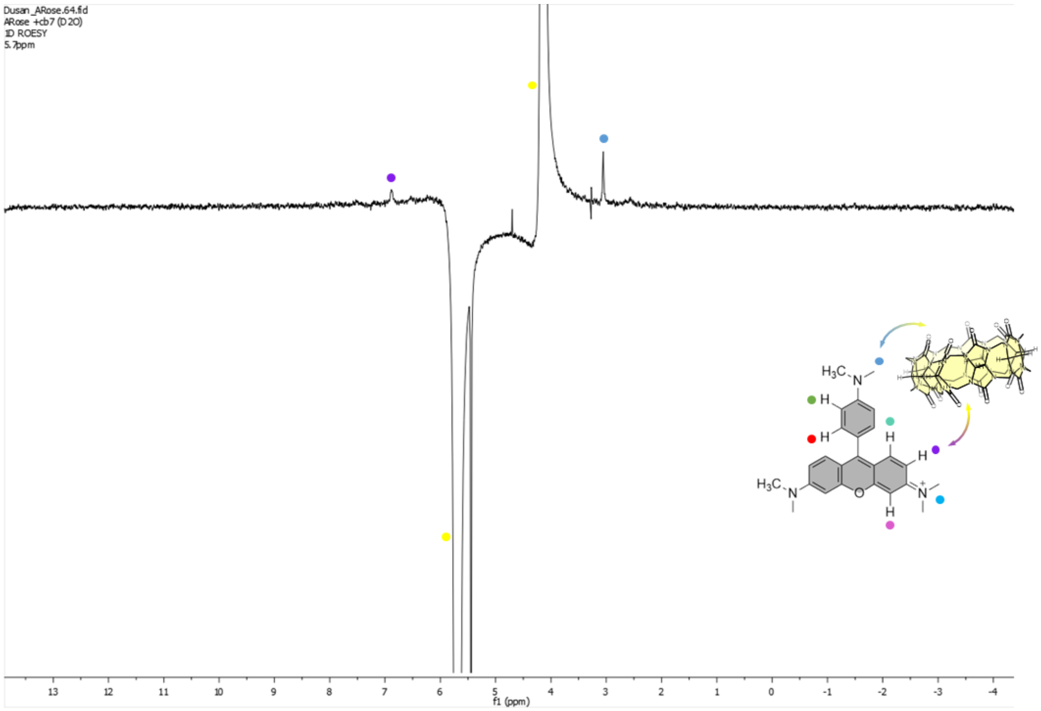

Figure S57. 1D ROESY spectrum of **ARose@CB7** in D_2O (*selrogp*/ 800 MHz), with selective irradiation at 5.80 ppm. Number of scans: 80; mixing time: 190 ms.

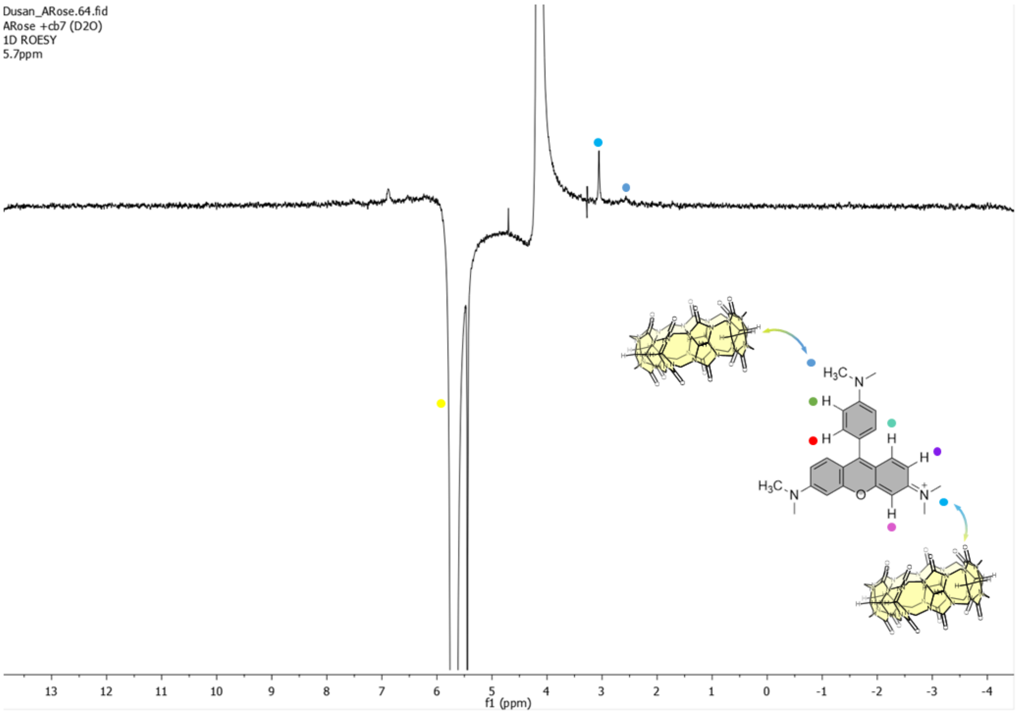

Figure S58. 1D ROESY spectrum of **ARose@CB7** in D_2O (*selrogp*/ 800 MHz), with selective irradiation at 5.54 ppm. Number of scans: 80; mixing time: 190 ms.

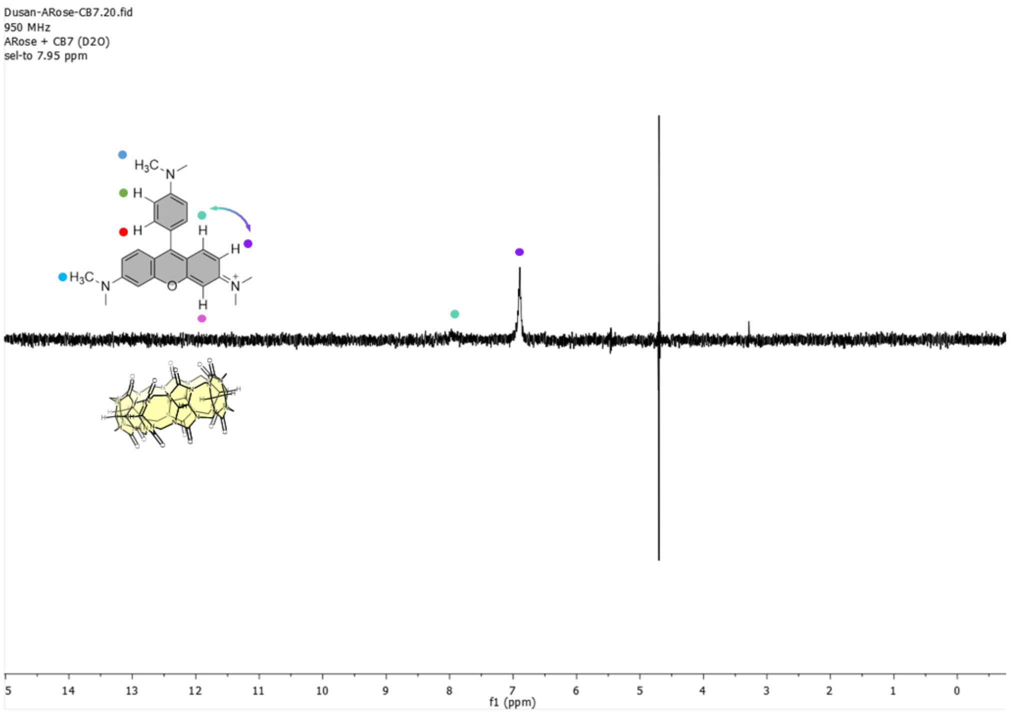

Figure S59. 1D TOCSY spectrum of **ARose@CB7** in D_2O (*selrogp*/ 800 MHz), with selective irradiation at 8.02 ppm. Number of scans: 120; mixing time: 40 ms.

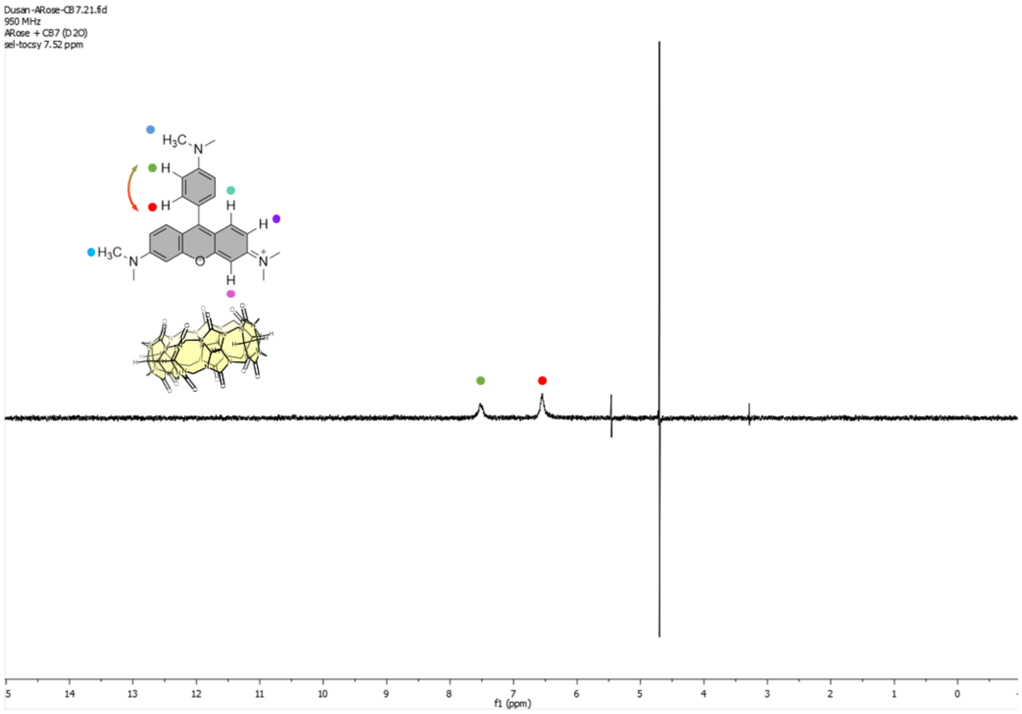

Figure S60. 1D TOCSY spectrum of **ARose@CB7** in D_2O (*selrogp*/ 800 MHz), with selective irradiation at 7.60 ppm. Number of scans: 120; mixing time: 40 ms.

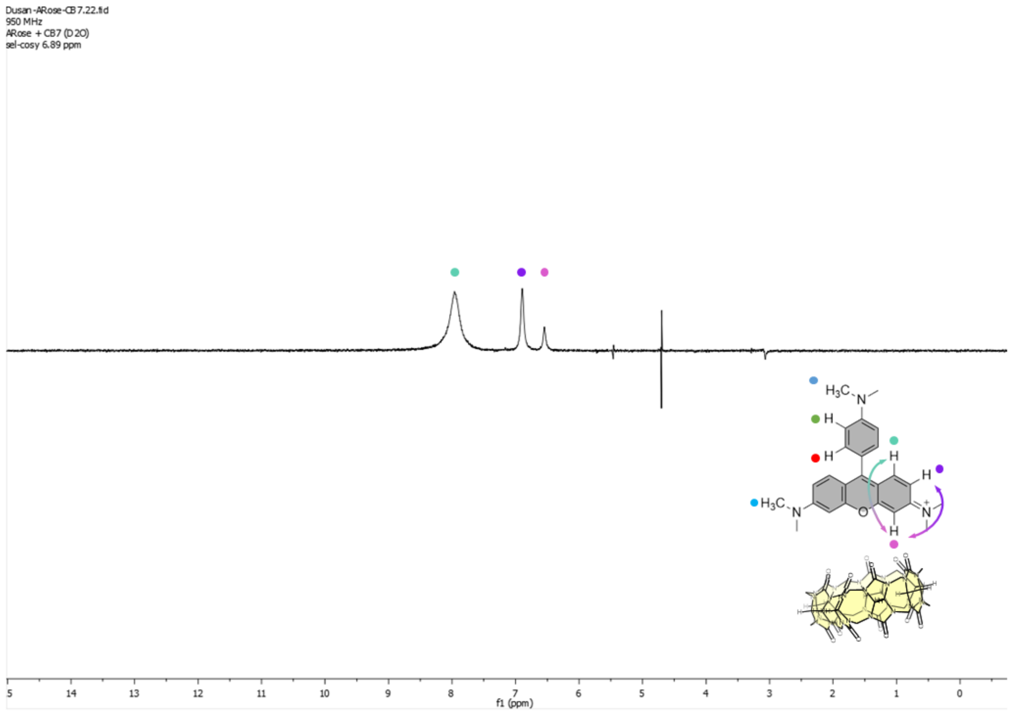

Figure S61. 1D TOCSY spectrum of **ARose@CB7** in D_2O (*selrogp*/ 800 MHz), with selective irradiation at 6.97 ppm. Number of scans: 120; mixing time: 40 ms.

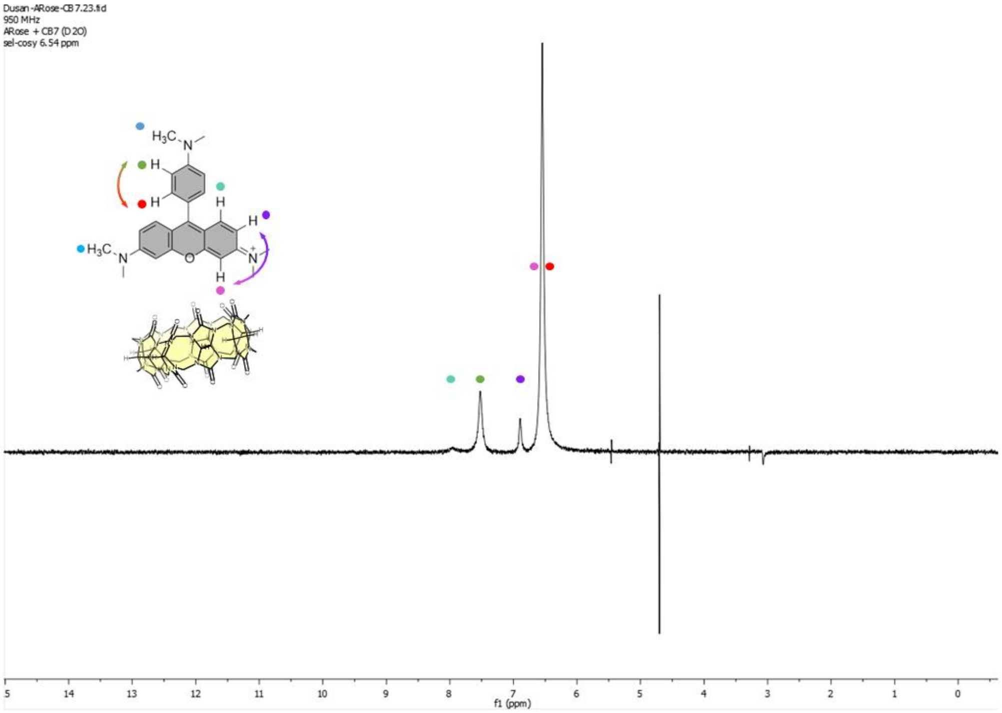

Figure S62. 1D TOCSY spectrum of **ARose@CB7** in D_2O (*selrogp*/ 800 MHz), with selective irradiation at 6.54 ppm. Number of scans: 120; mixing time: 40 ms.

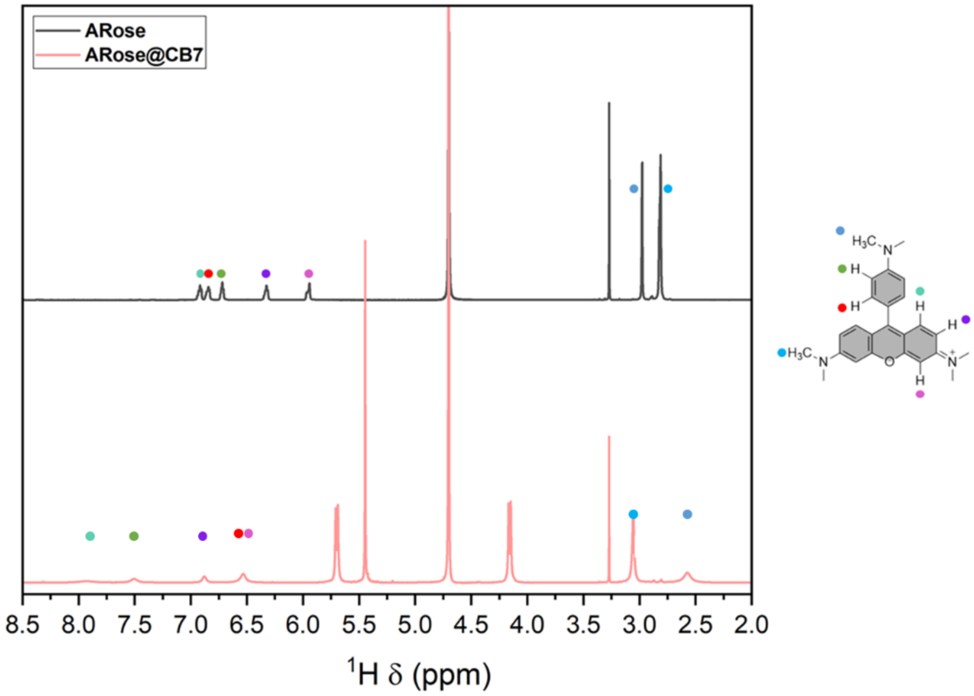

Figure S63. ^{1}H NMR analysis of chemical shifts upon host-guest interaction (saturated D_2O solutions of **ARose** and **ARose@CB7**).

P-ARose

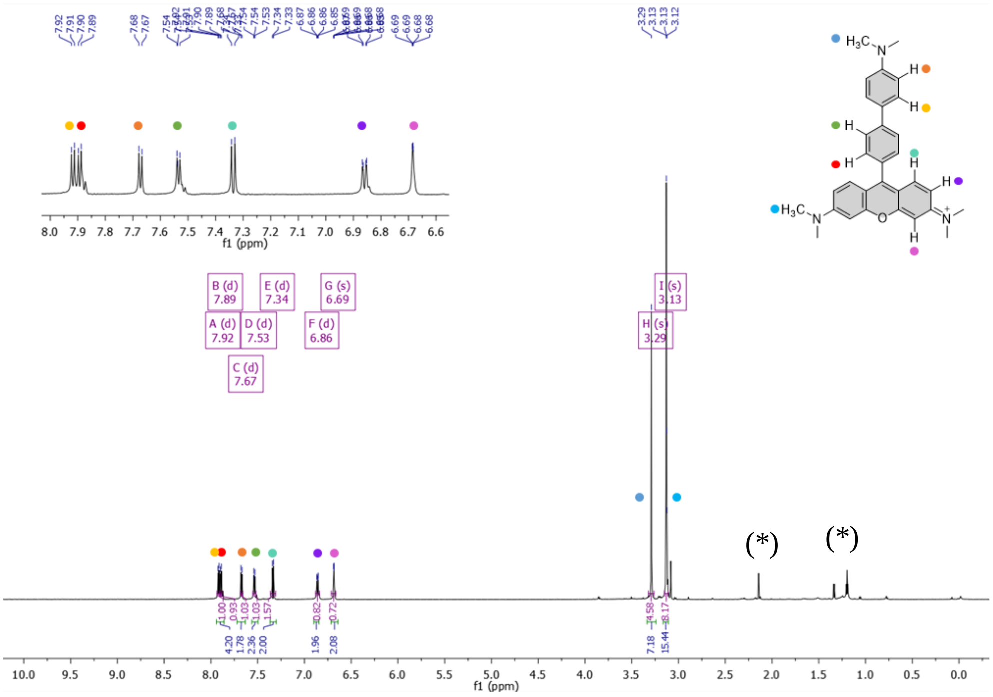

Figure S64. ^{1}H NMR experiment in D_2O (800 MHz), using watergate for water suppression (*zggpw5*). Impurities are denoted by (*). Number of scans: 16.

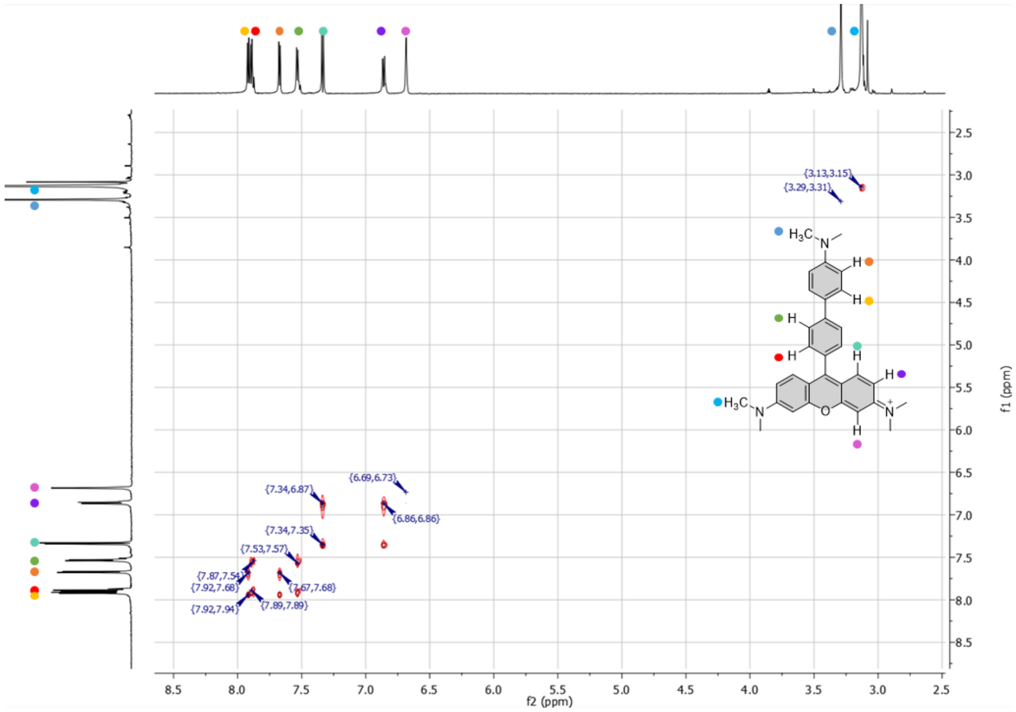

Figure S65. ^{1}H-^{1}H COSY NMR experiment.

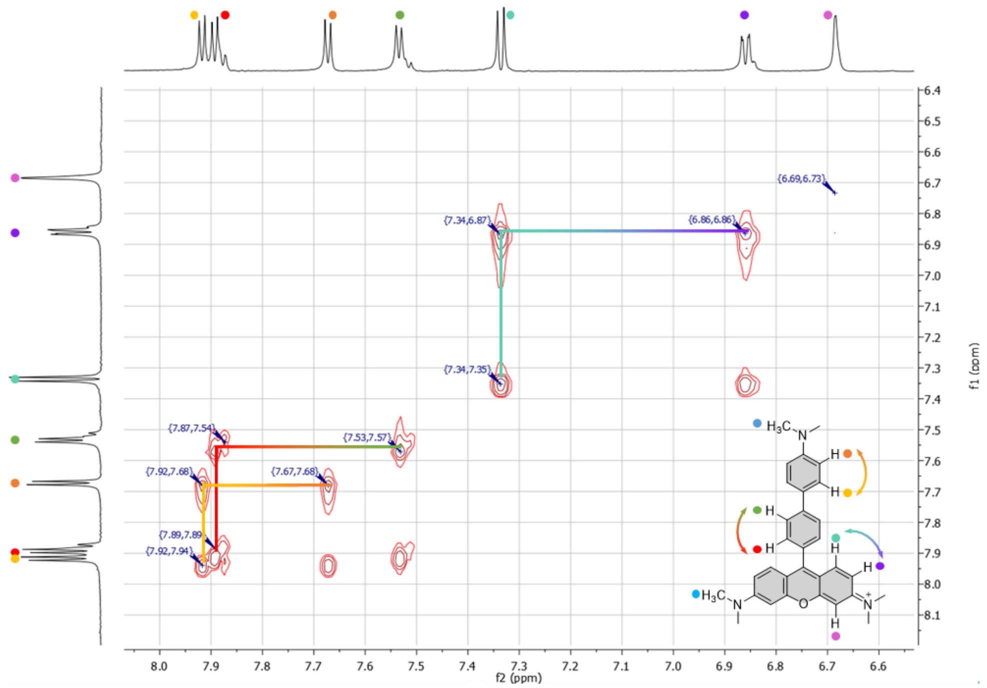

Figure S66. ^{1}H-^{1}H COSY NMR experiment. The aromatic area is enlarged. The color coding is used to indicate specific correlations.

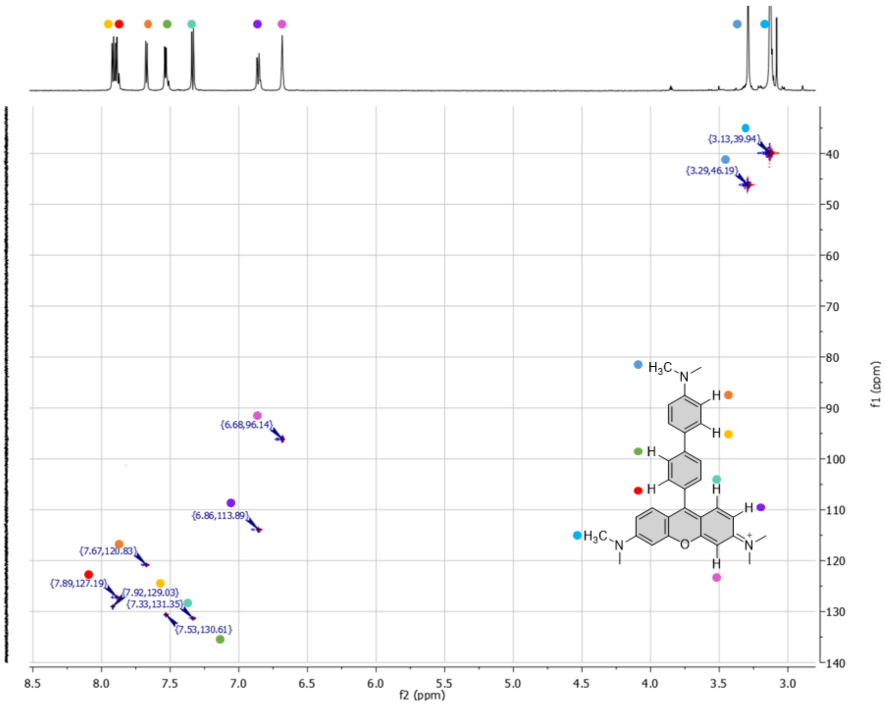

Figure S67. ^{1}H-^{13}C HSQC NMR experiment.

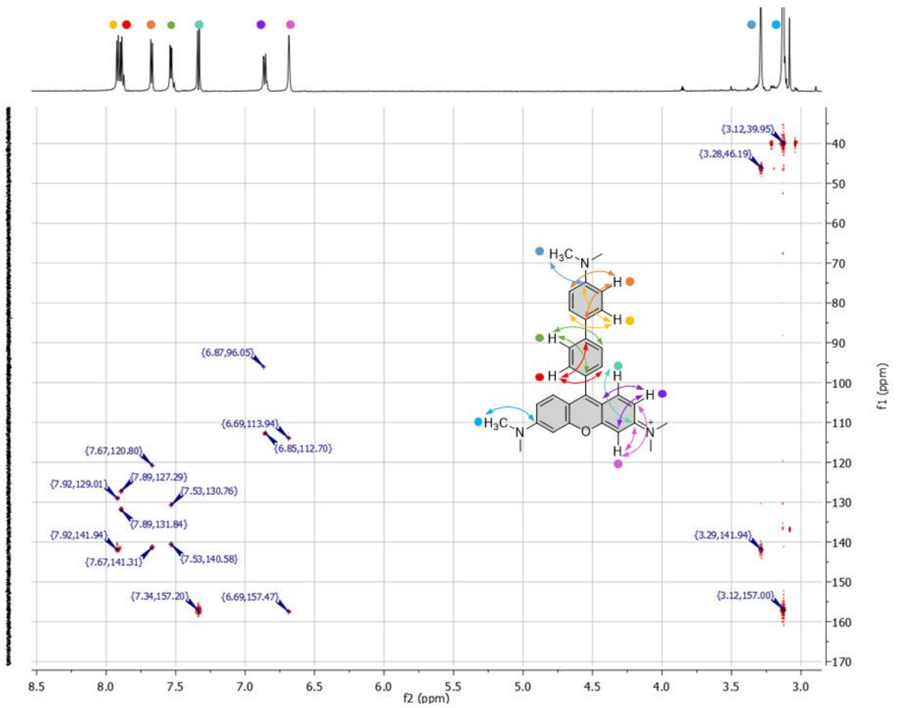

Figure S68. ^{1}H-^{13}C HMBC NMR experiment. The color coding is used to indicate specific correlations.

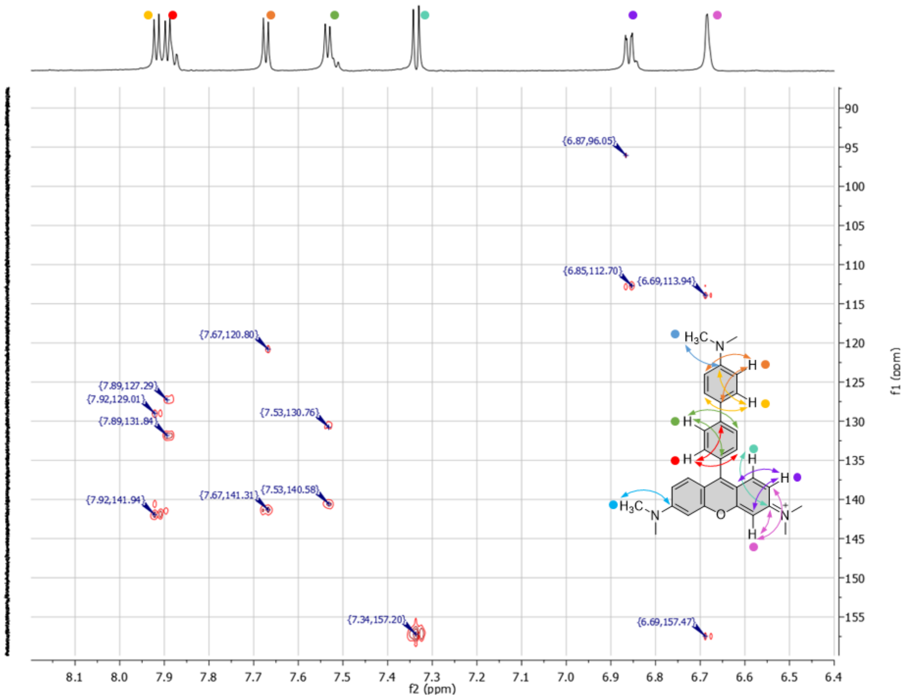

Figure S69. ^{1}H-^{13}C HMBC NMR experiment The aromatic area is enlarged. The color coding is used to indicate specific correlations.

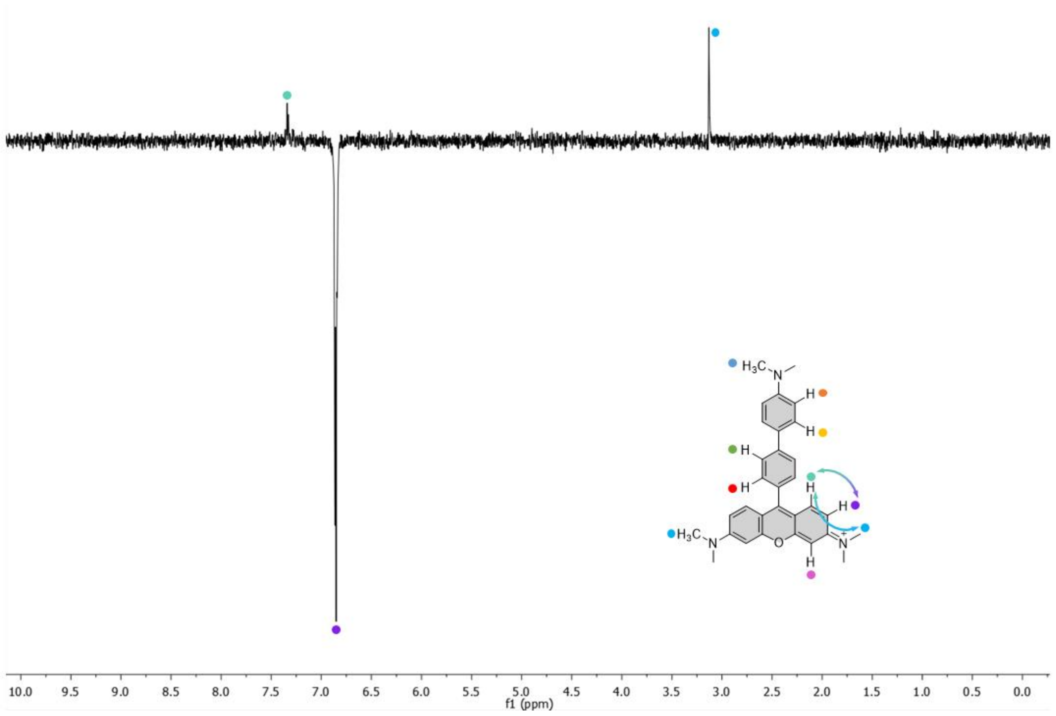

Figure S70. 1D ROESY spectrum of **P-ARose** in D_2O (*selrogp*/ 800 MHz), with selective irradiation at H7 (6.68 ppm). Number of scans: 320; mixing time: 200 ms.

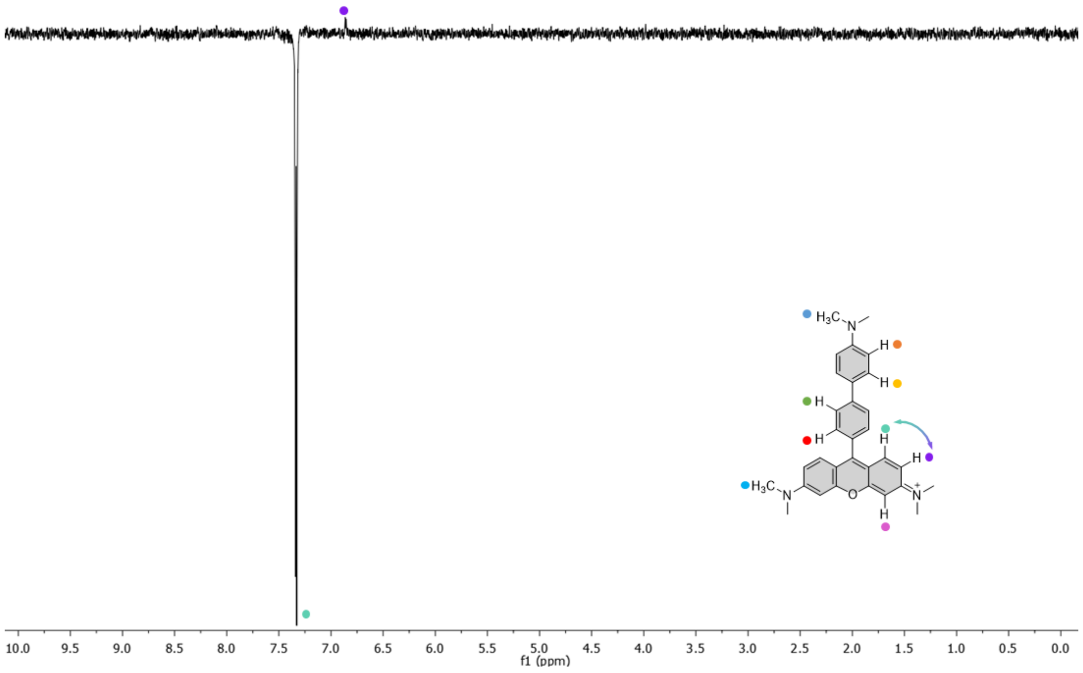

Figure S71. 1D ROESY spectrum of **P-ARose** in D_2O (*selrogp*/ 800 MHz), with selective irradiation at H7 (7.34 ppm). Number of scans: 320; mixing time: 200 ms.

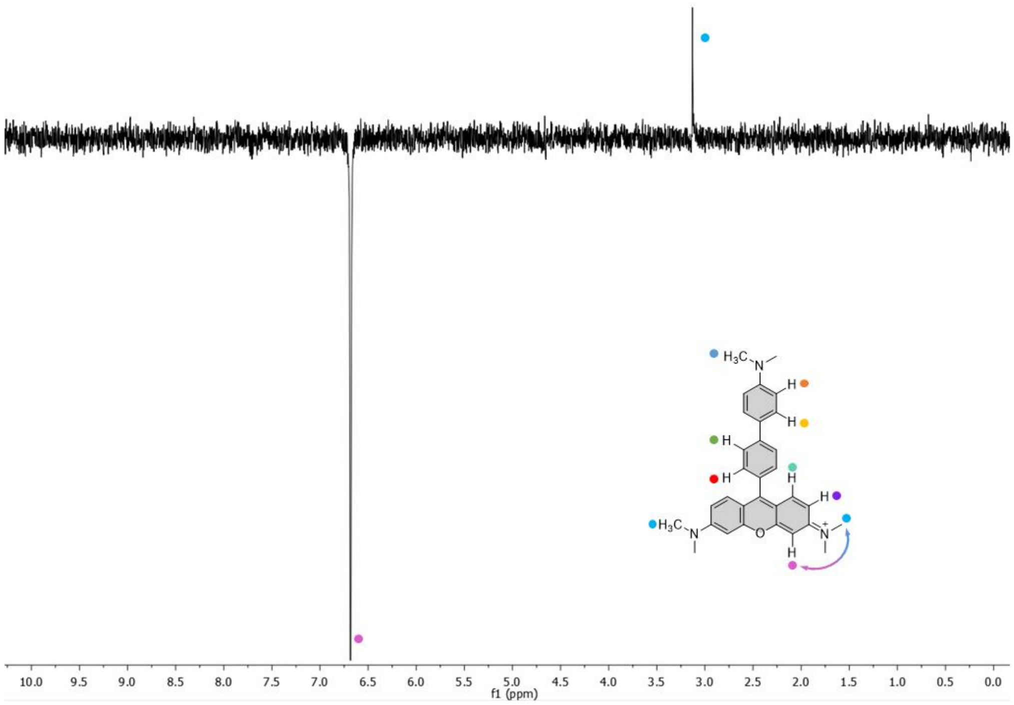

Figure S72. 1D ROESY spectrum of **P-ARose** in D_2O (*selrogp*/ 800 MHz), with selective irradiation at H7 (7.69 ppm). Number of scans: 320; mixing time: 200 ms.

P-ARose@CB7

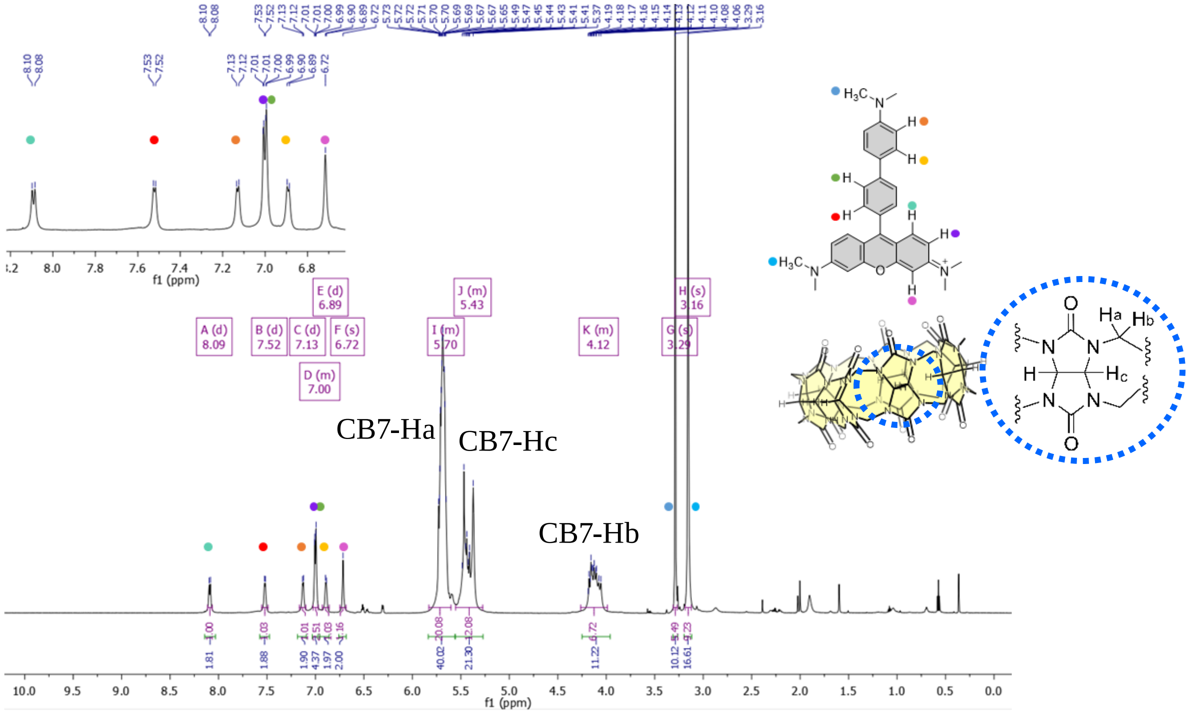

Figure S73. ^{1}H 1D spectrum of **P-ARose@CB7** in D_2O (800 MHz), using watergate for water suppression (*zggpw5*). Number of scans: 16.

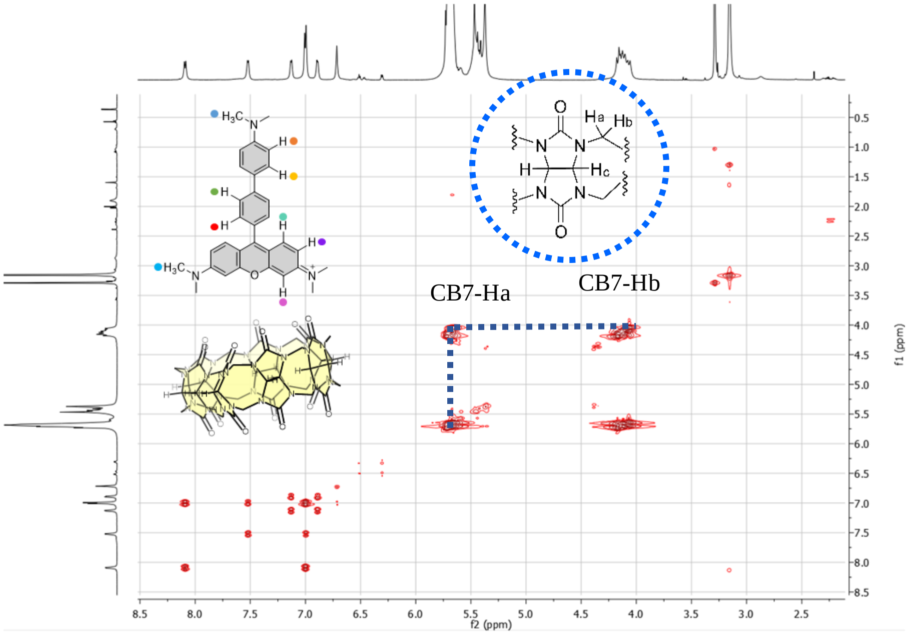

Figure S74. ^{1}H-^{1}H COSY spectrum of **P-ARose@CB7** in D_2O (800 MHz), using Watergate-based 3-9-19 suppression for water suppression (*cosydfgpph19*). Relevant COSY correlation is indicated with a dashed blue line. Number of scans: 16.

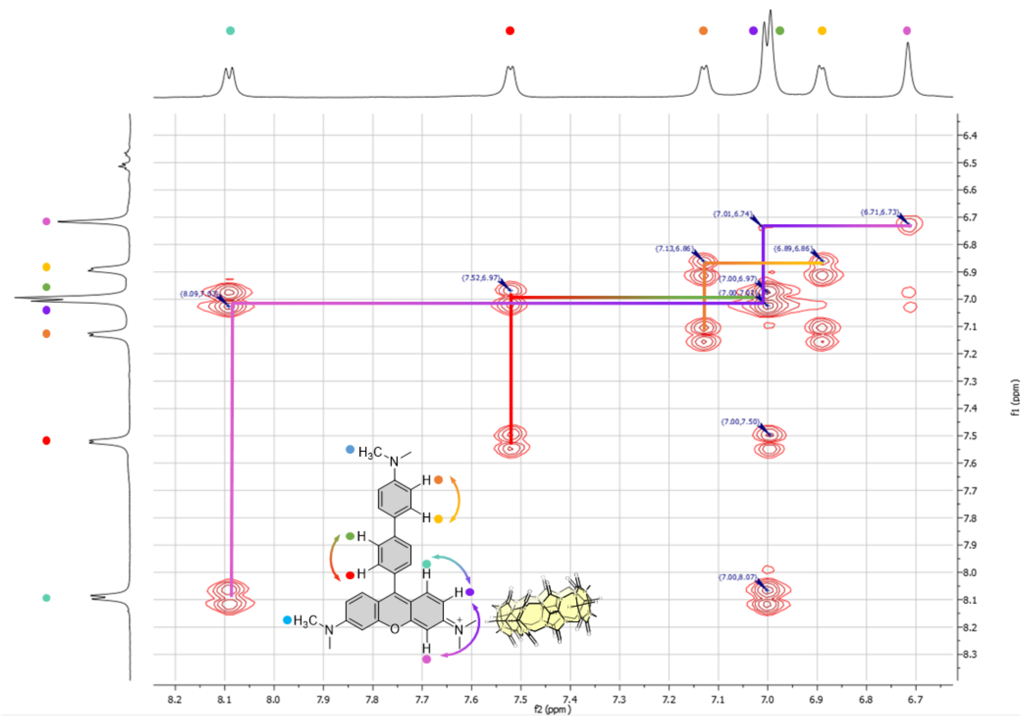

Figure S75. ^{1}H-^{1}H COSY spectrum of **P-ARose@CB7** in D_2O (800 MHz), using Watergate-based 3-9-19 suppression for water suppression (*cosydfgpph19*). Number of scans: 16. The color coding is used to indicate specific correlations.

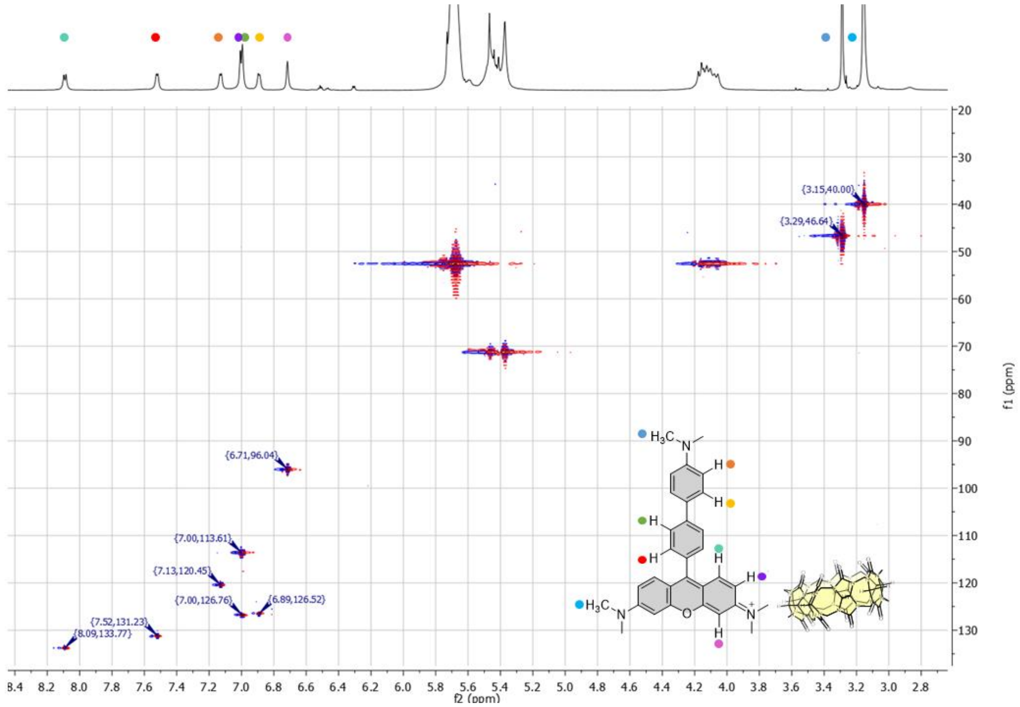

Figure S76. HSQC spectrum of **P-ARose@CB7** in D_2O (800 MHz), using Watergate suppression for water suppression (*hsqcf3gpph19*). $^1J_{CH}$ = 145 Hz; Number of scans: 48.

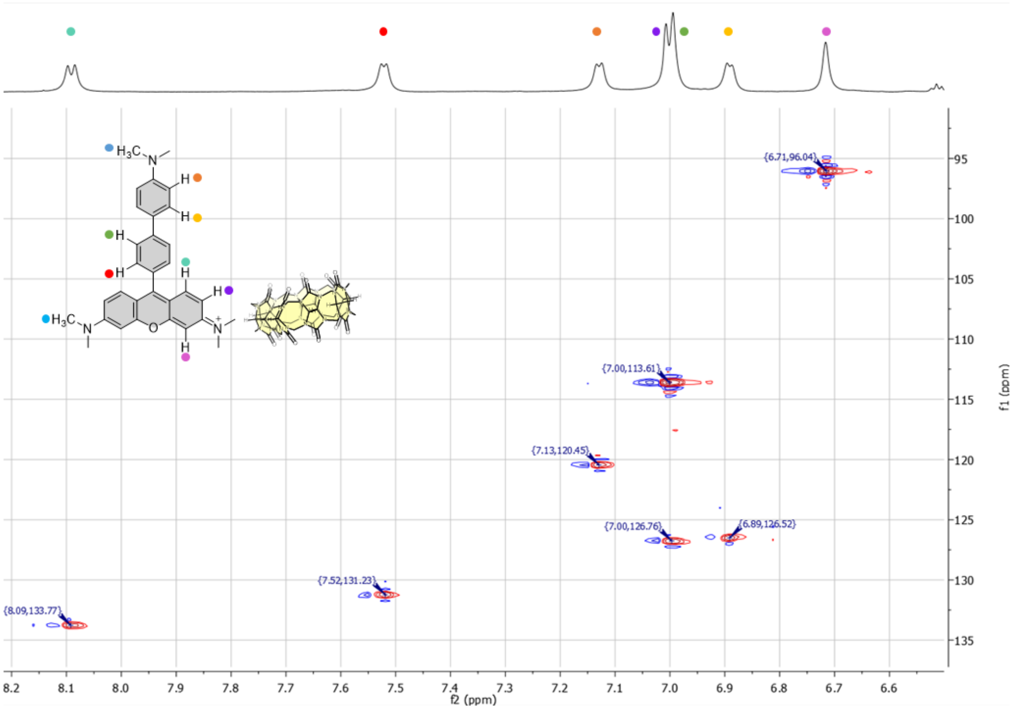

Figure S77. HSQC spectrum of **P-ARose@CB7** in D_2O (800 MHz), using Watergate suppression for water suppression (*hsqcf3gpph19*). $^1J_{CH}$ = 145 Hz; Number of scans: 48.

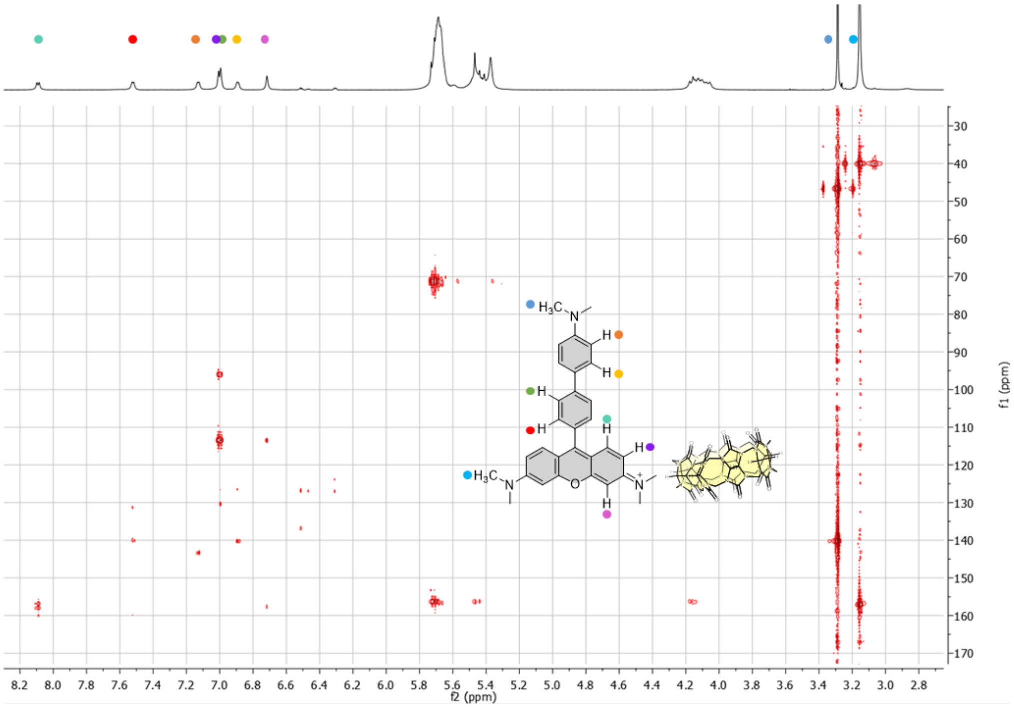

Figure S78. HMBC spectrum of **P-ARose@CB7** in D_2O (800 MHz), using Watergate suppression for water suppression (*hmbcgpl2ndwg*). $^nJ_{CH}$ = 8 Hz; $^1J_{CH}$Min/Max = 120/165 Hz; Number of scans: 80.

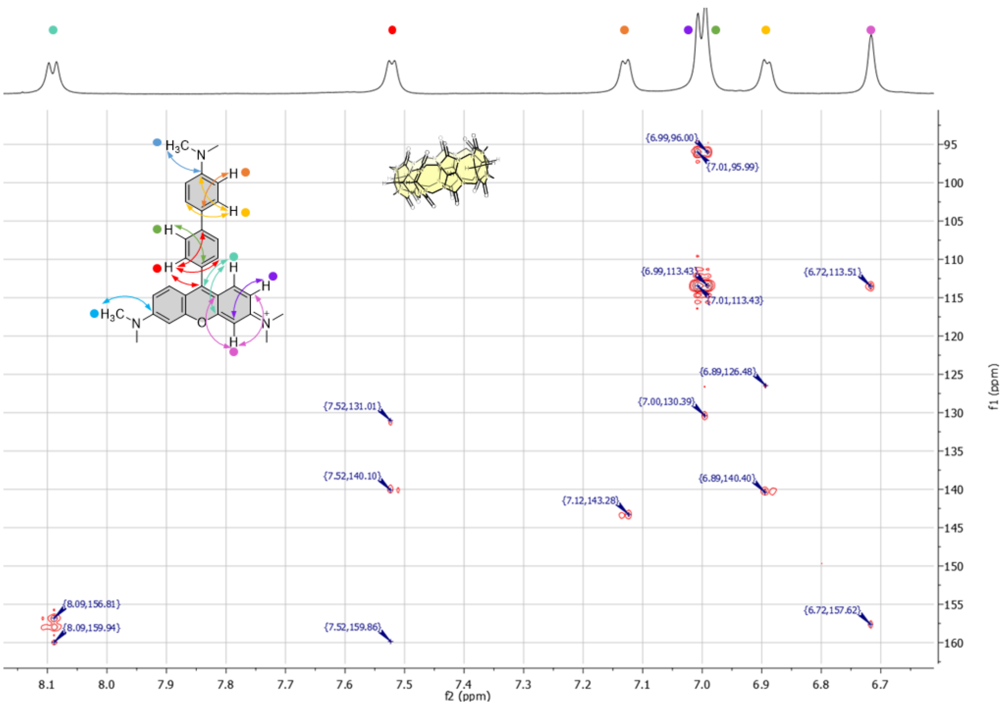

Figure S79. HMBC spectrum of **P-ARose@CB7** in D_2O (800 MHz), using Watergate suppression for water suppression (*hmbcgpl2ndwg*). $^nJ_{CH}$ = 8 Hz; $^1J_{CH}$Min/Max = 120/165 Hz; Number of scans: 80. The color coding is used to indicate specific correlations.

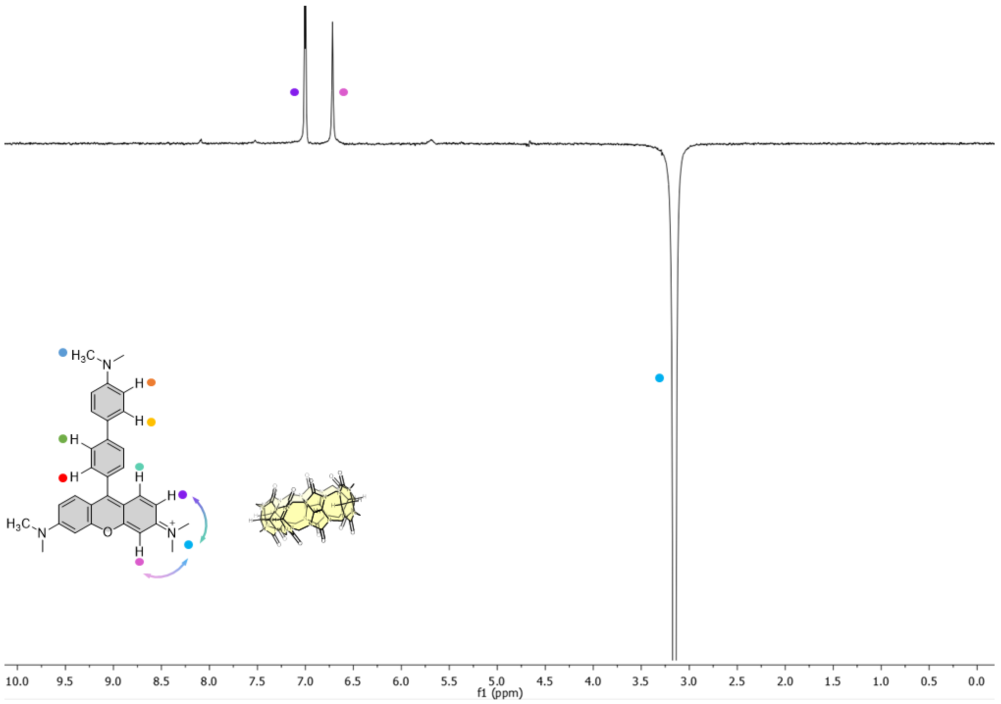

Figure S80. 1D ROESY spectrum of **P-ARose@CB7** in D_2O (*selrogp*/ 800 MHz), with selective irradiation at Me-N^+ (3.1705 ppm). Number of scans: 320; mixing time: 200 ms.

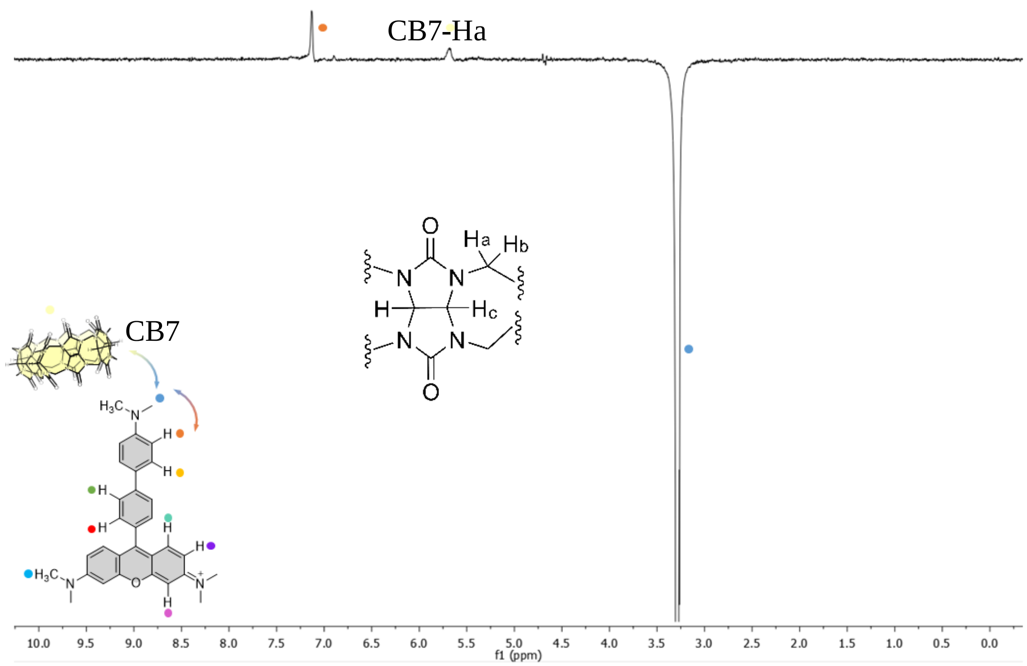

Figure S81. 1D selective ROESY spectrum of **P-ARose@CB7** in D_2O (Bruker *selrogp*, 800 MHz), with selective inversion at Me–N (3.3010 ppm). The observed ROE contact between Me–N and CB7 cavity protons (CB7-Ha 5.6801 ppm) confirms their spatial proximity and supports inclusion complex formation.

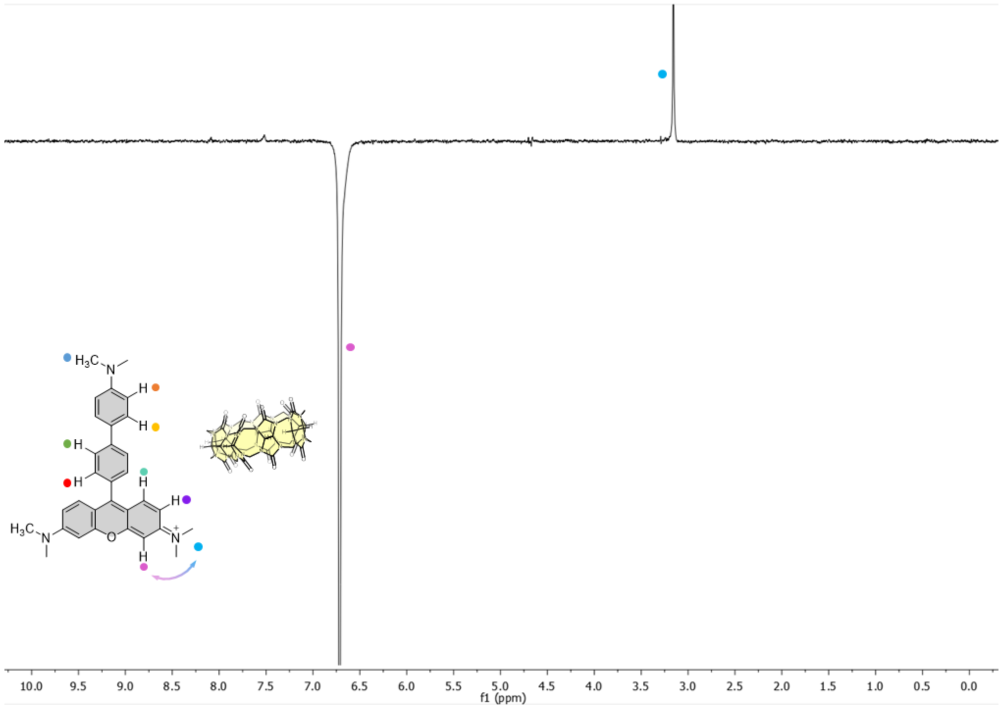

Figure S82. 1D selective ROESY spectrum of **P-ARose@CB7** in D_2O (Bruker *selrogp*, 800 MHz), with selective inversion at H5 (5.7020 ppm). The observed ROE contact between H5 and Me–N-C6 (3.1568 ppm) allows its assignment.

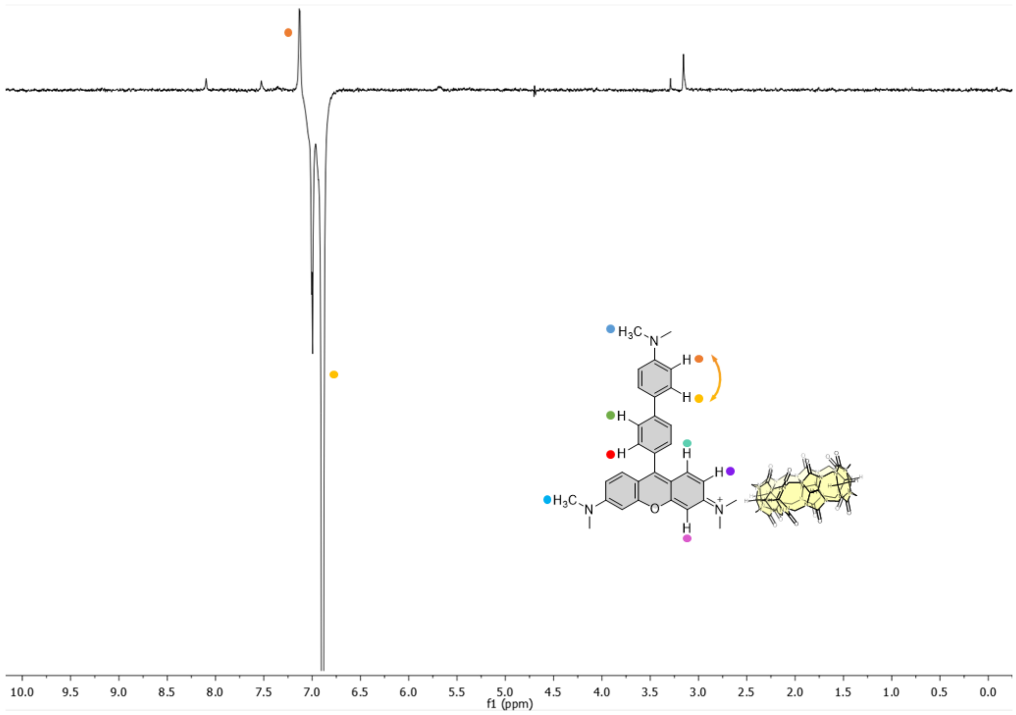

Figure S83. 1D ROESY spectrum of **P-ARose@CB7** in D_2O (*selrogp*/ 800 MHz), with selective irradiation at H6' (6.8922ppm). Number of scans: 320; mixing time: 200 ms.

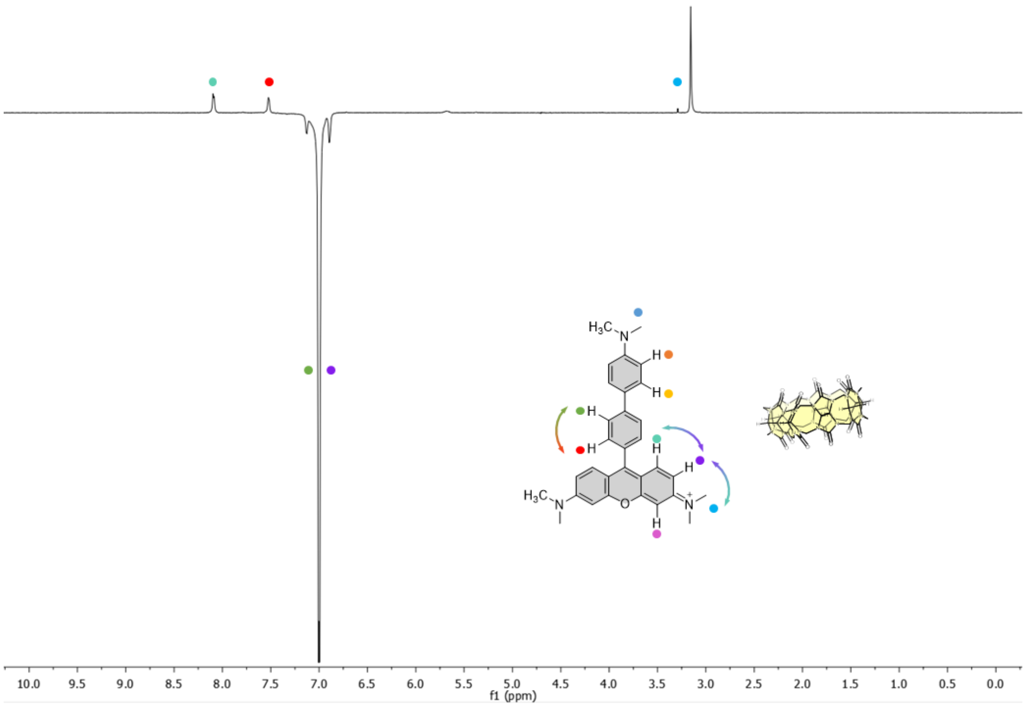

Figure S84. 1D selective ROESY spectrum of **P-ARose@CB7** in D_2O (Bruker *selrogp*, 800 MHz), with selective inversion at H7/H3' (7.0020 ppm). The observed ROE contact between H7 and Me–N-C6 (3.1581 ppm) allows its assignment.

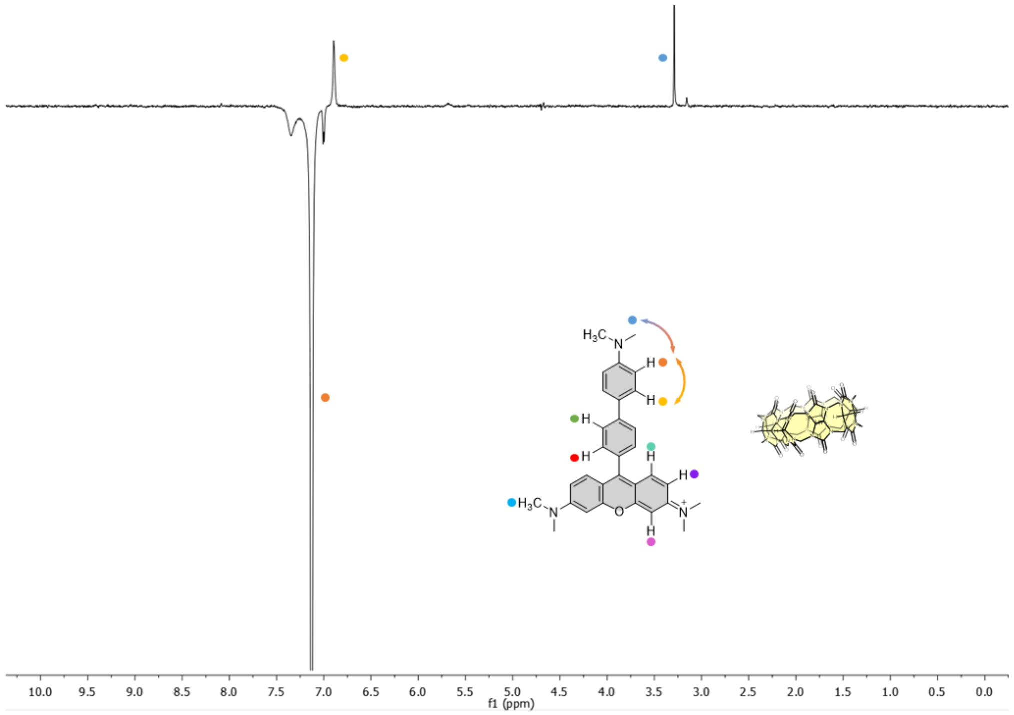

Figure S85. 1D selective ROESY spectrum of **P-ARose@CB7** in D_2O (Bruker *selrogp*, 800 MHz), with selective inversion at H7' (7.1332 ppm). The observed ROE contact between H5' and Me–N-C8' (3.2888 ppm) at meso-moiety allows its assignment.

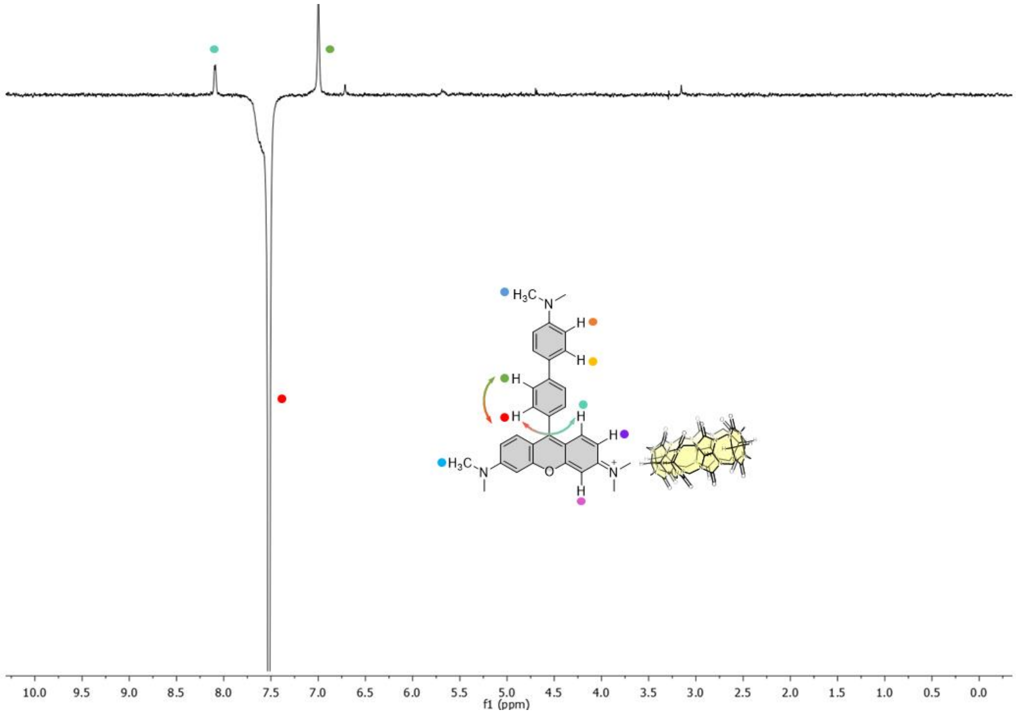

Figure S86. 1D selective ROESY spectrum of **P-ARose@CB7** in D_2O (Bruker *selrogp*, 800 MHz), with selective inversion at H2' (7.5221 ppm).

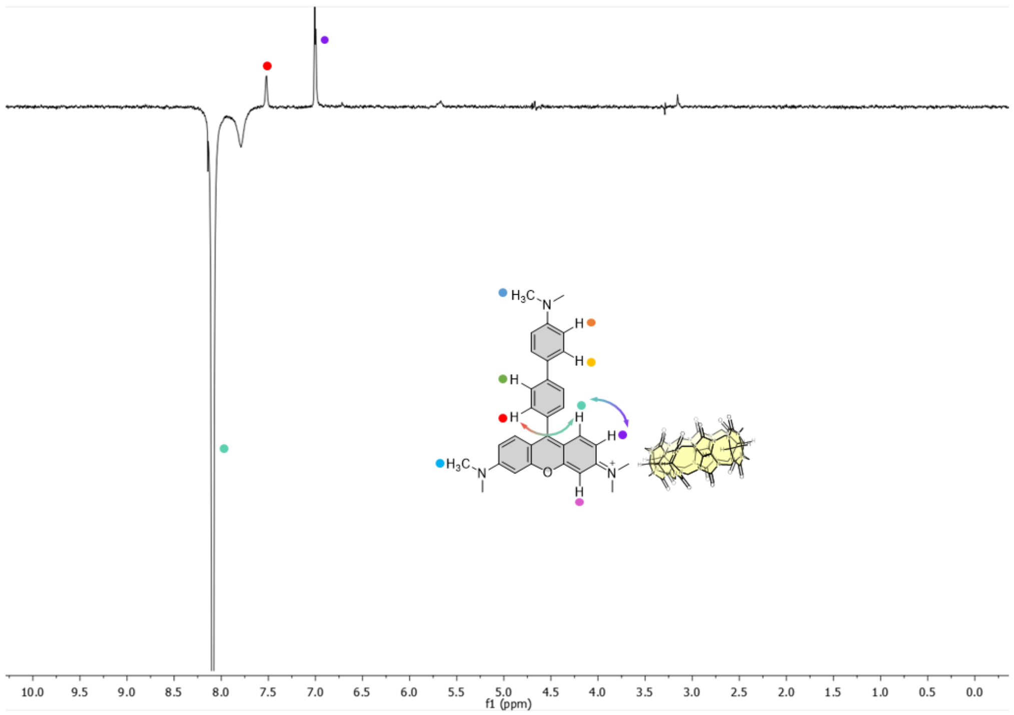

Figure S87. 1D selective ROESY spectrum of **P-ARose@CB7** in D_2O (Bruker *selrogp*, 800 MHz), with selective inversion at H8 (8.0910 ppm).

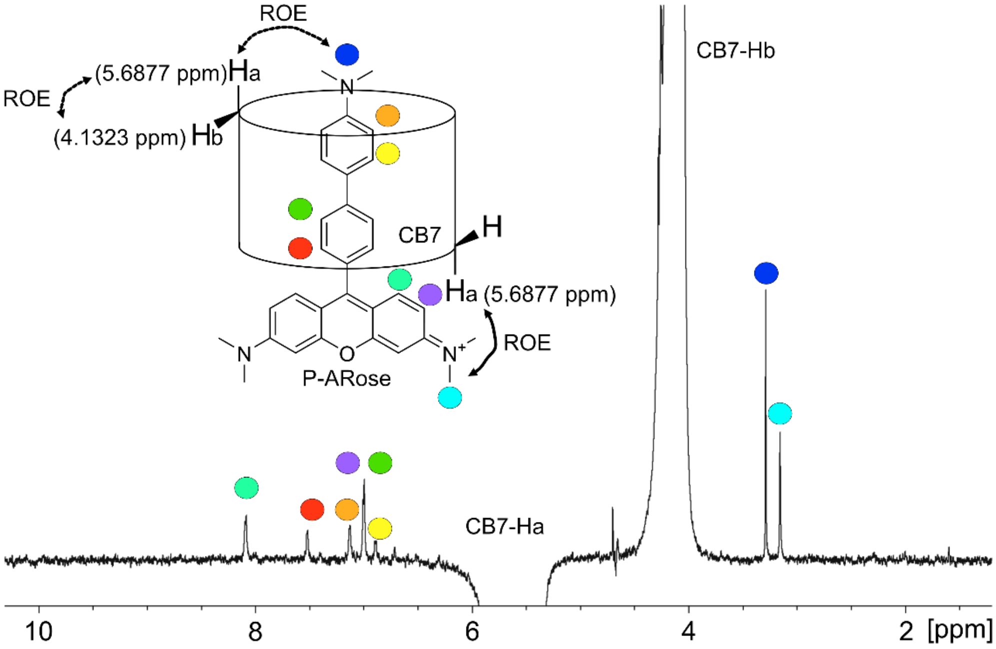

Figure S88. 1D selective ROESY spectrum of **P-ARose@CB7** in D_2O (Bruker *selrogp*, 800 MHz), with selective inversion at CB7-Ha (5.6878 ppm). The observed ROE contact between CB7-Ha and several protons of **P-Arose** confirms their spatial proximity and supports inclusion complex formation.

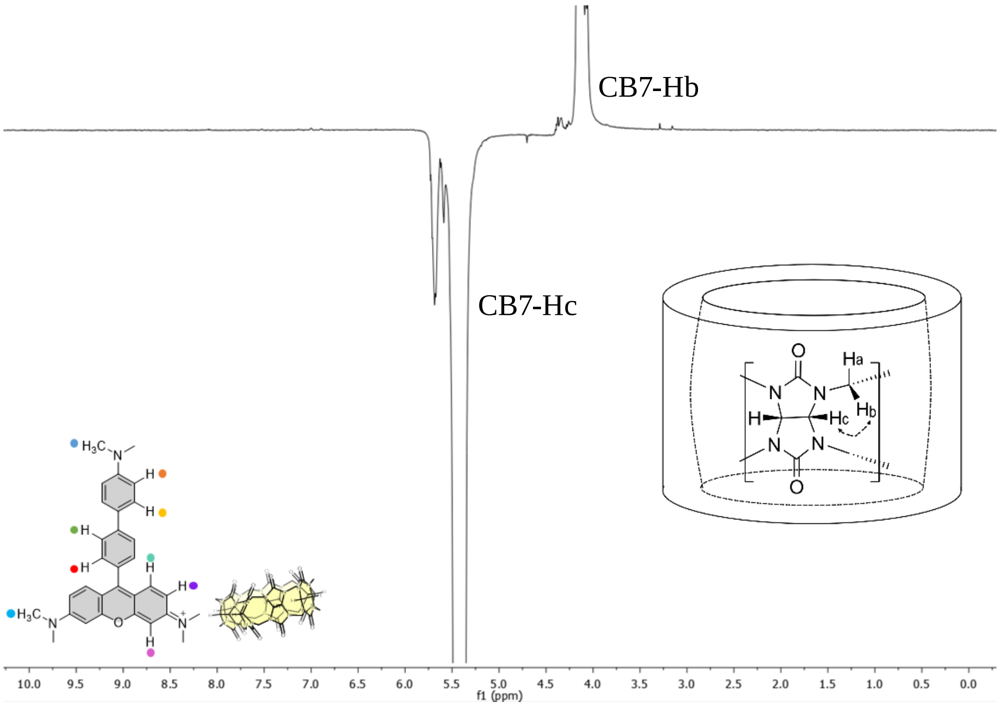

Figure S89. 1D selective ROESY spectrum of **P-ARose@CB7** in D_2O (Bruker *selrogp*, 800 MHz), with selective inversion at CB7-Hc (5.421 ppm).

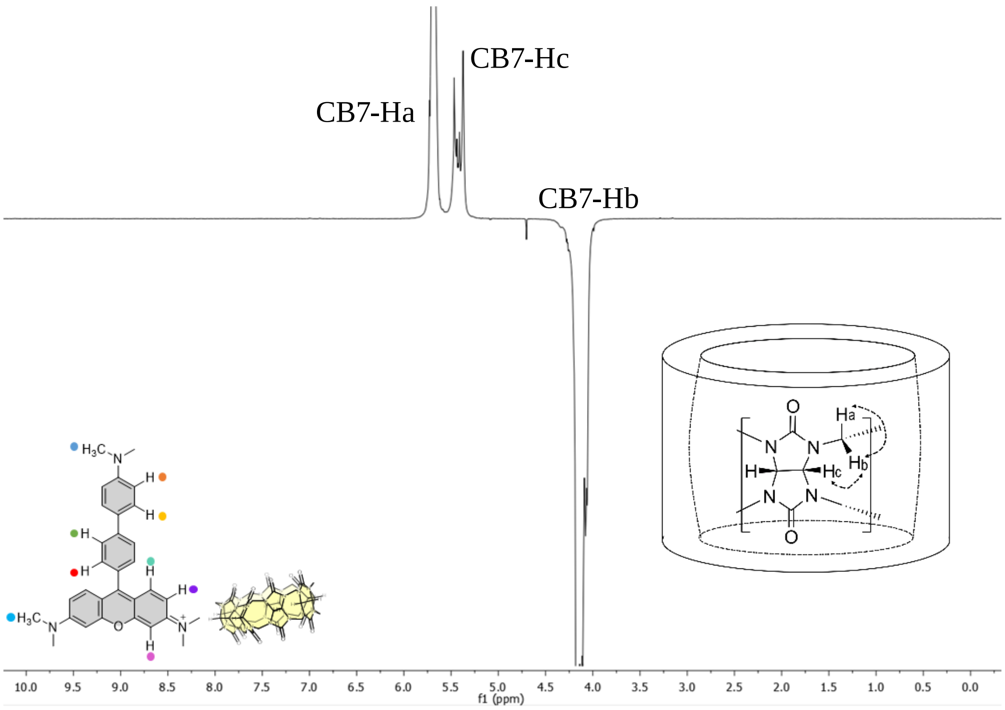

Figure S90. 1D selective ROESY spectrum of **P-ARose@CB7** in D_2O (Bruker *selrogp*, 800 MHz), with selective inversion at CB7-Hb (4.118 ppm).

Photobleaching of P-ARose

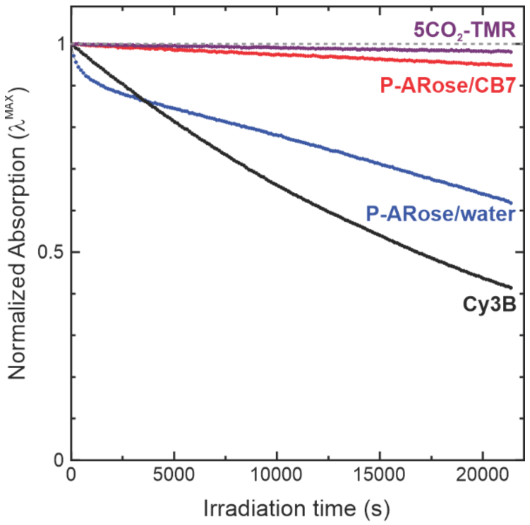

Figure S91. Comparative photobleaching of compound **P-ARose** in aqueous solution and excess CB7 (20x), Cy3B and 5CO2-TMR. CB7 considerably increase the fatigue resistance of **P-ARose**, reaching a similar value to TMR, with Cy3B showing worse performance (even than **P-ARose** unbound).

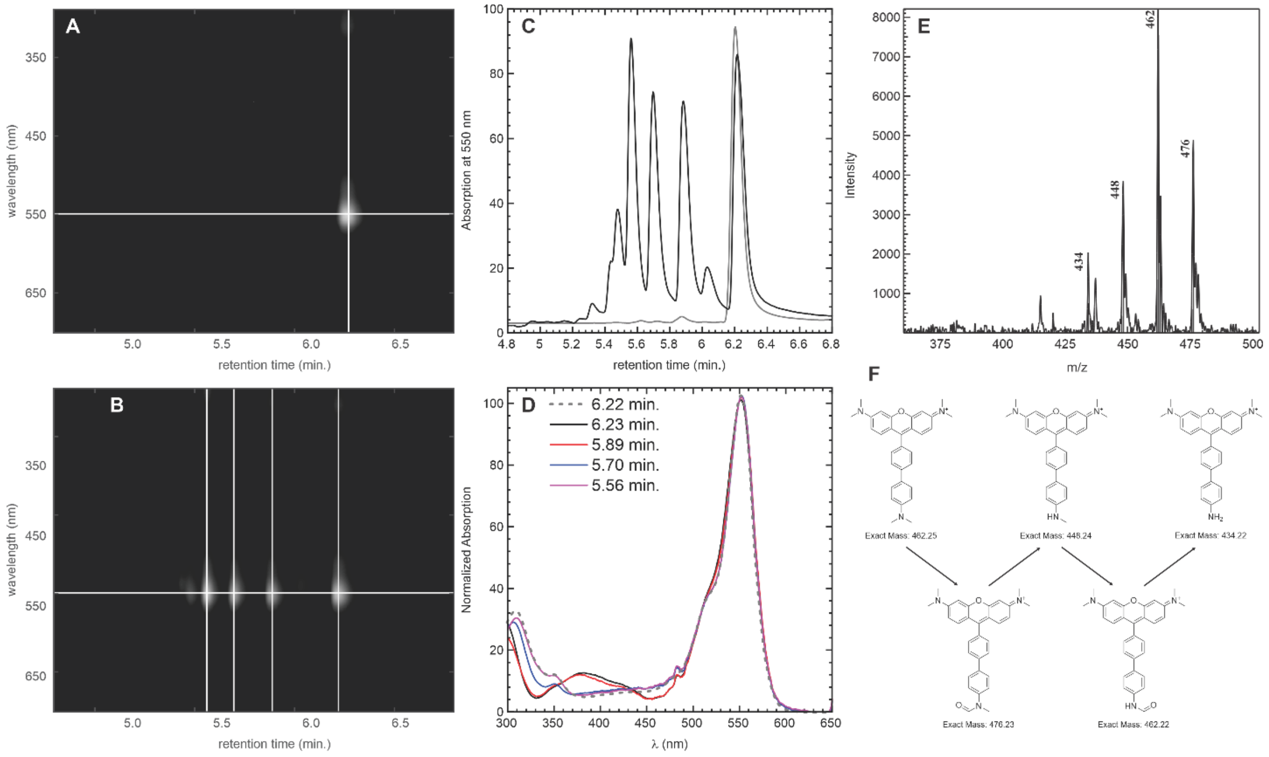

Figure S92. 2D LC-MS maps of **P-ARose** before (A) and after (B) photobleaching with 550 nm light in aqueous solution (without CB7); (C) Chromatogram at 550 nm of the solution before (gray) and after irradiation (black); (D) Normalized absorption of the starting compound (dotted line) and the four main peaks after irradiation, at the indicated retention times; (E) MS spectra integrated for the range containing the four peaks of the irradiated solution; (F) Proposed bleaching intermediates based on the observed masses, the normalized spectra, and published reports.[4]

Imaging

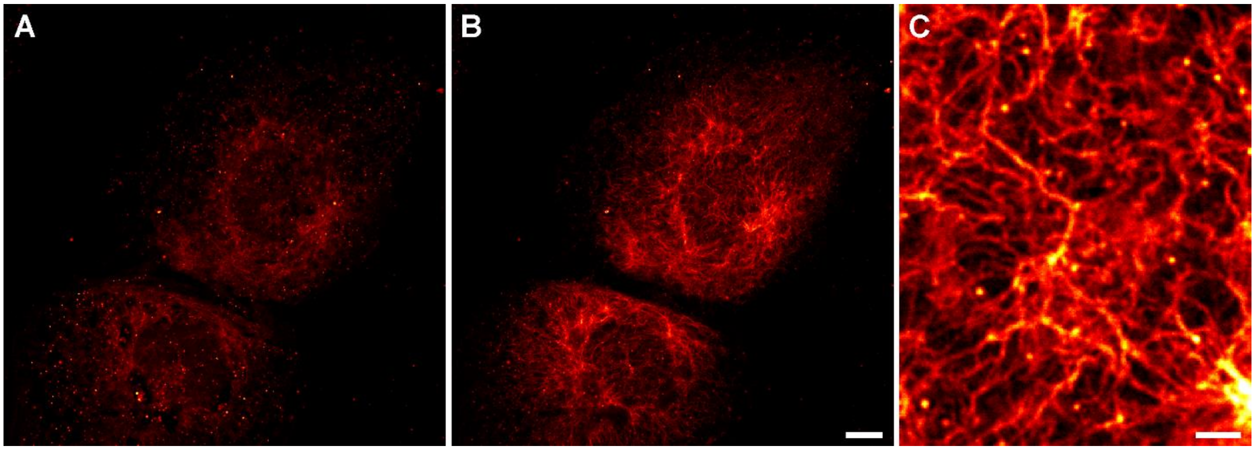

Figure S93. Emission turn-ON of compound **10** upon labeling HaloTag-fused vimentin in fixed U2OS cells. The samples were fixed and permeabilized, and then labeled with at a dye concentration of 1 µM for 1 h in PBS/BSA (2%). Confocal images acquired before (**A**) and after (**B**) CB7 addition (1.5 mM final concentration). (**C**) Confocal image of a zoomed ROI, recorded in the presence of CB7 addition. Scale bars: 10 µm (A-B) and 2 µm (C).

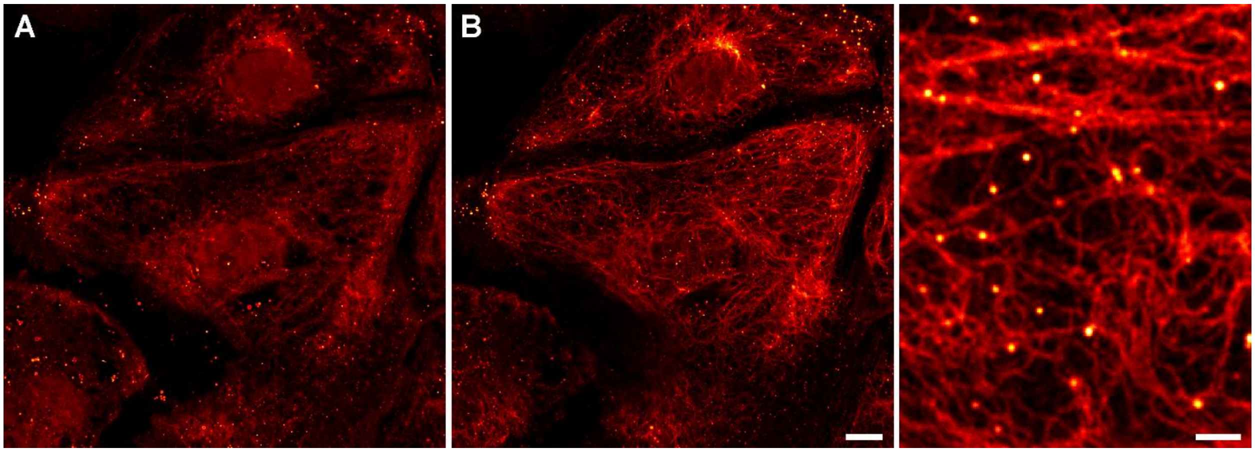

Figure S94. Emission turn-ON of compound **12** upon labeling HaloTag-fused vimentin in fixed U2OS cells. The samples were fixed and permeabilized, and then labeled with at a dye concentration of 1 µM for 1 h in PBS/BSA (2%). Confocal images acquired before (**A**) and after (**B**) CB7 addition (1.5 mM final concentration). (**C**) Confocal image of a zoomed ROI, recorded in the presence of CB7 addition. Scale bars: 10 µm (A-B) and 2 µm (C).

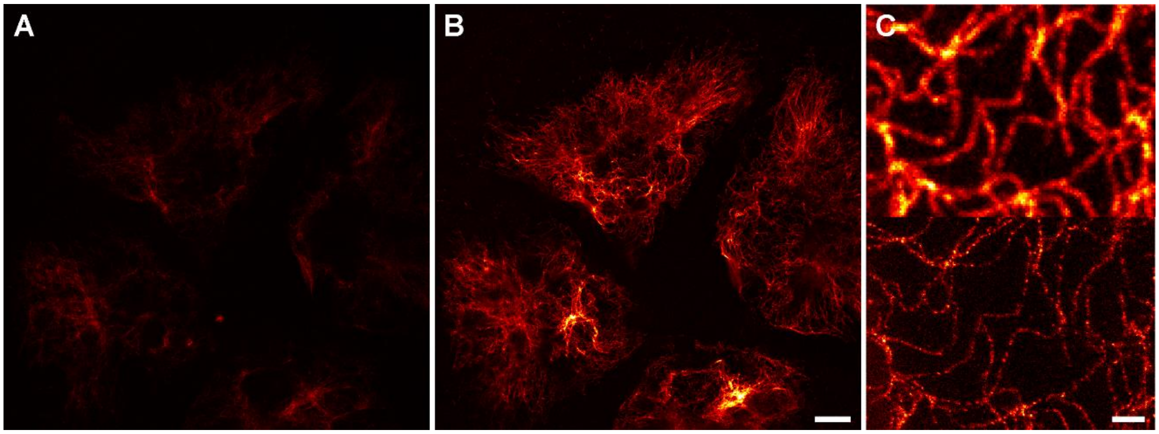

Figure S95. Emission turn-ON of compound **(11)** upon labeling HaloTag-fused vimentin in live U2OS cells. Labelling was performed at a dye concentration of 1 µM for 1 h in the cell medium, and then fixed before imaging. Confocal images acquired before (**A**) and after (**B**) CB7 addition (1.5 mM final concentration). (**C**) Confocal (top) and STED (bottom) images of a zoomed ROI, recorded in the presence of CB7 addition. Scale bars: 10 µm (A-B) and 1 µm (C).

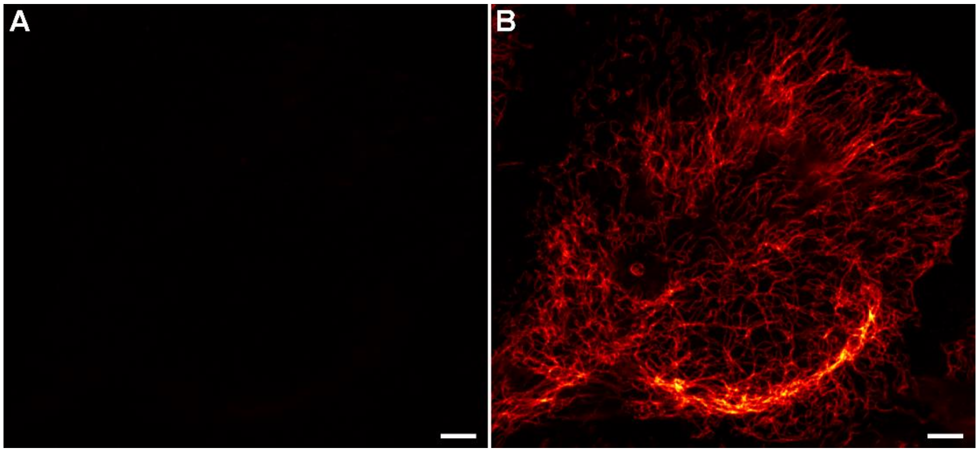

Figure S96. Emission turn-ON of **P-ARose-AB** upon supramolecular complexation with CB7 on fixed U2OS cells, labeled with primary antibody against Vimentin, and a secondary antibody labeled with **P-ARose**-modified NHS ester. The sample was mounted and imaged in water (**A**), and then imaged again immediately after the addition of CB7 to a final concentration of 1.5 mM (**B**). Scale bars: 5 μm

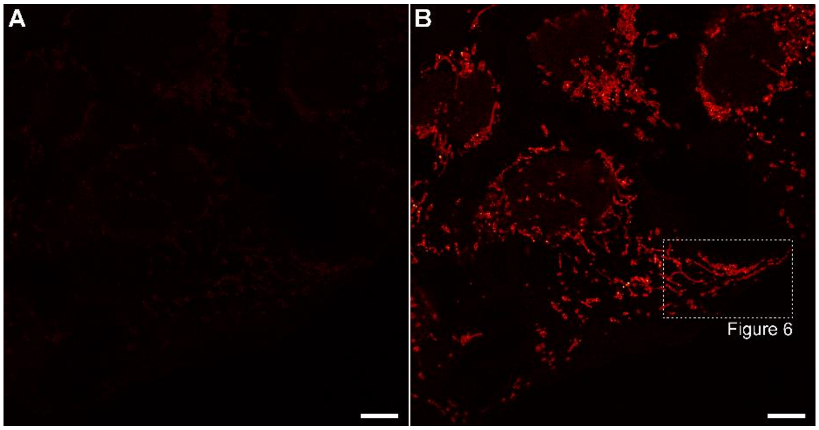

Figure S97. Emission turn-ON of **P-ARose-AB** upon supramolecular complexation with CB7 on fixed U2OS cells, labelled with primary antibody against TOM20, and a secondary antibody labelled with **P-ARose**-modified NHS ester. The sample was mounted and imaged in water (**A**) and then imaged again immediately after the addition of CB7 to a final concentration of 1.5 mM (**B**). Images are presented in the same scale. The ROI used for quantification is indicated (Figure 6). Scale bars: 10 µm.

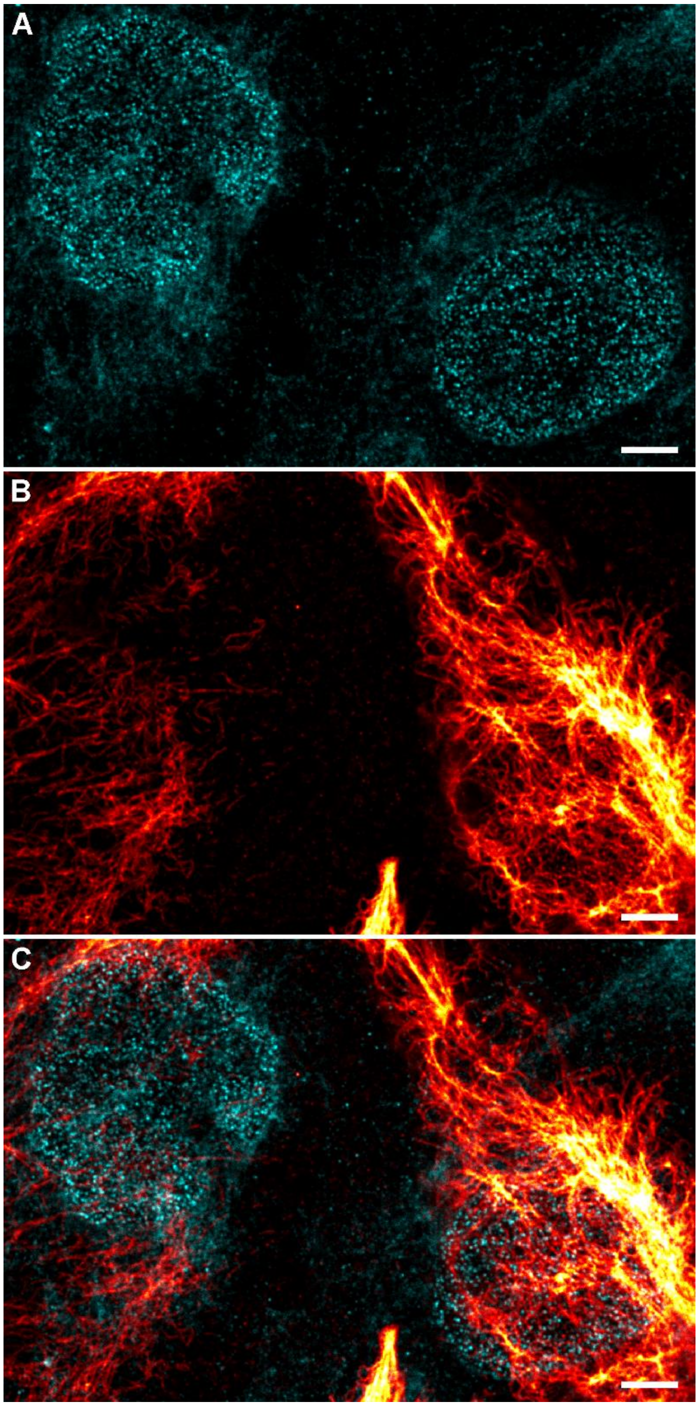

Figure S98. False two-color image acquired by sequential imaging Cy3B (**A**), bleaching, and then imaging **P-ARose-AB** (**B**) after its signal was turned on by supramolecular complexation with CB7 (1.5 mM). The combined image is shown in **C**. U2OS cells were fixed and labeled with two primary antibodies against NUP153 and Vimentin, and a secondary nanobody labeled with Cy3B combined with a secondary antibody labeled with **P-ARose-AB**. Scale bars: 5 µm.

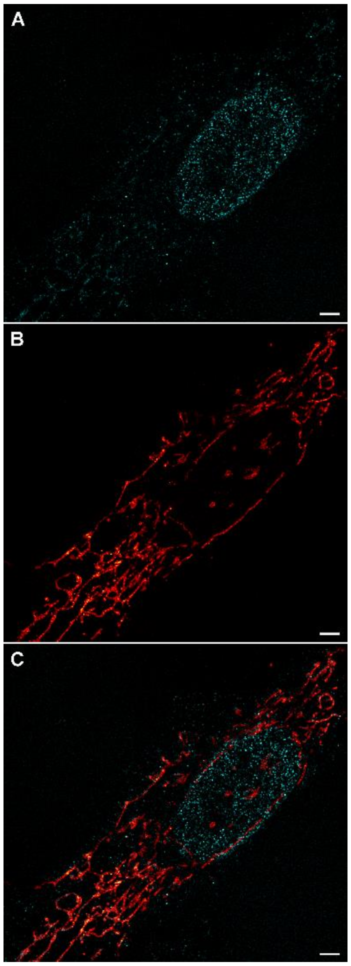

Figure S99. False two-color image acquired by sequential imaging Cy3B (**A**), bleaching, and then imaging **P-ARose-AB** (**B**) after its signal was turned on by supramolecular complexation with CB7 (1.5 mM). The combined image is shown in **C**. U2OS cells were fixed and labeled with two primary antibodies against TOM20 and NUP153, and a secondary nanobody labeled with Cy3B combined with a secondary antibody labeled with **P-ARose-AB**. Scalebars: 5 µm.

NMR spectra

Spectra

ARose

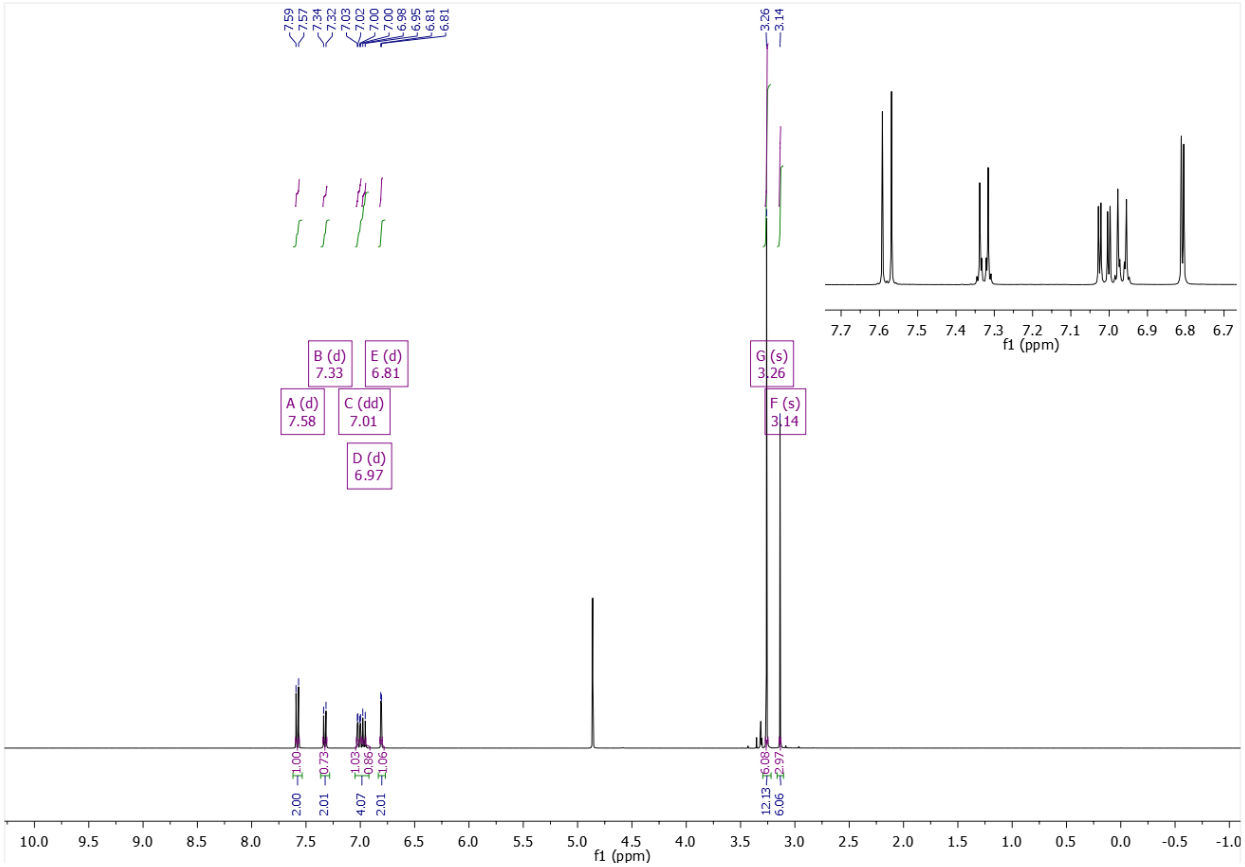

Figure S100. ^{1}H NMR spectrum (CD_3OD) of compound **ARose**.

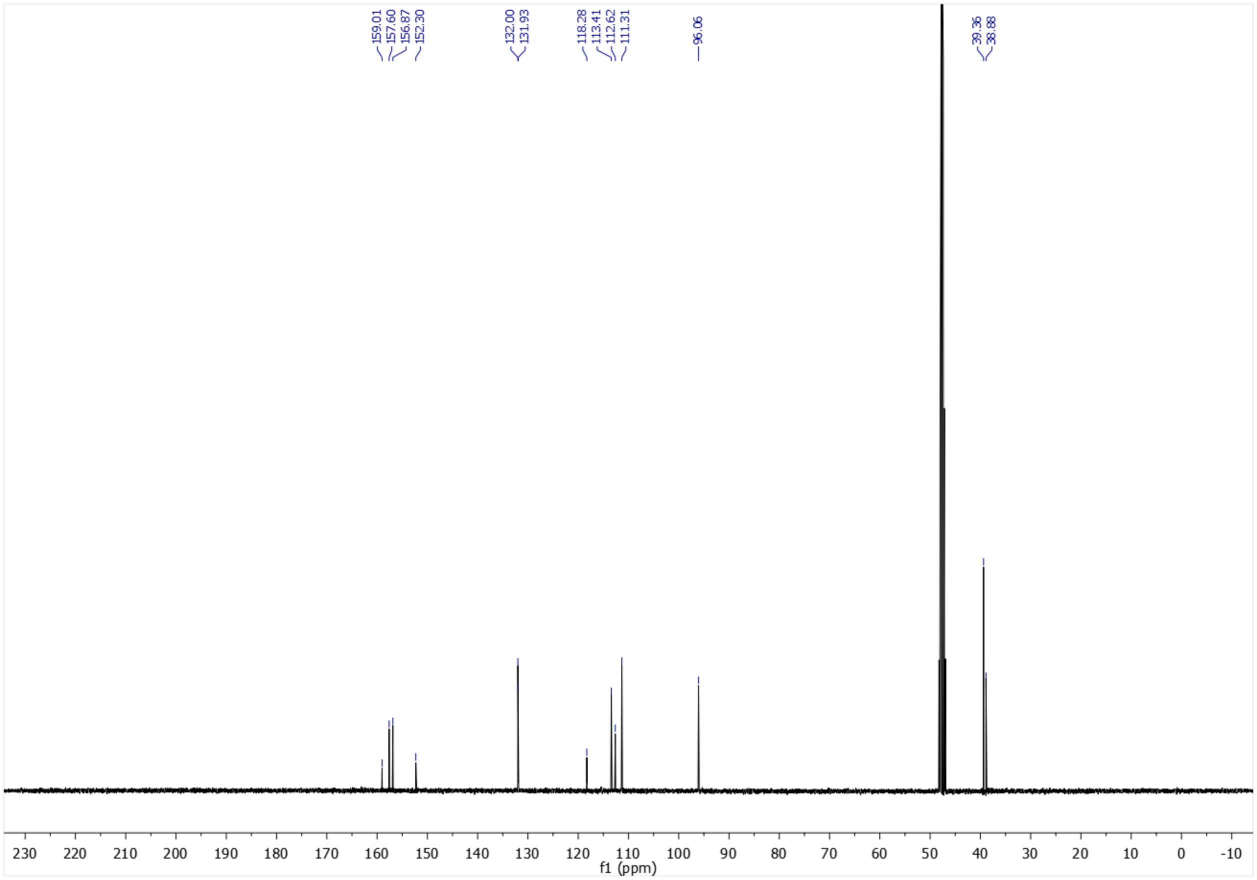

Figure S101. ^{13}C NMR spectrum (CD_3OD) of compound **ARose**.

P-ARose

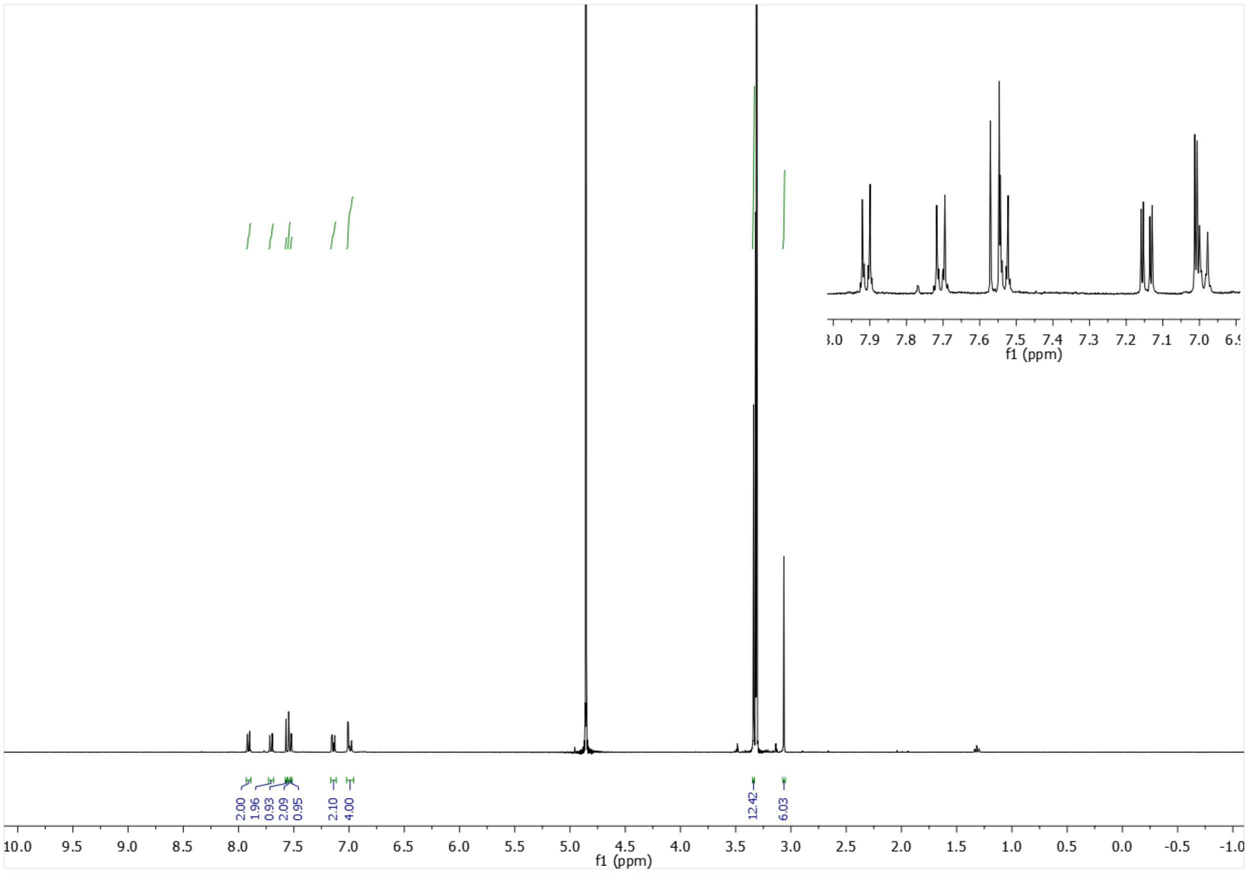

Figure S102. ^{1}H NMR spectrum (CD_3OD) of compound **P-ARose**.

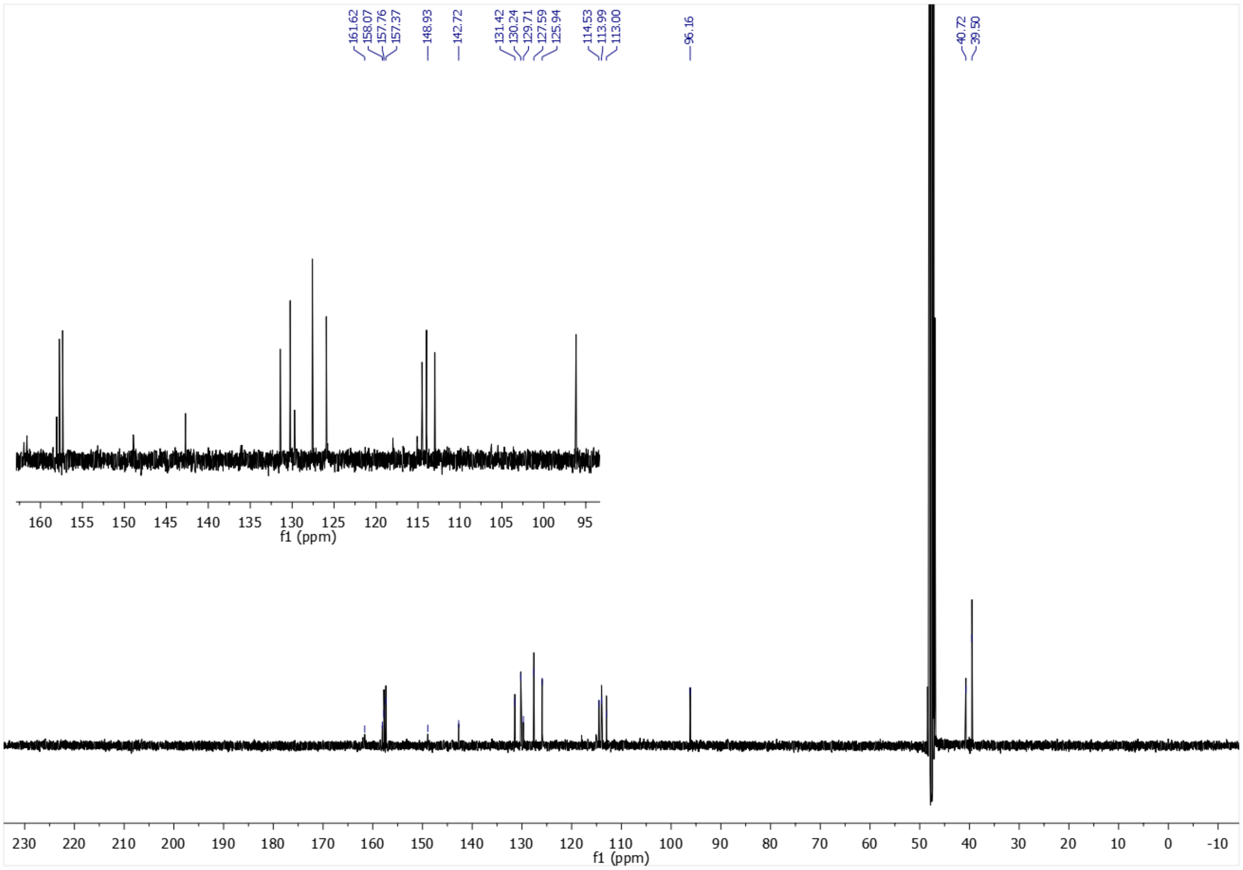

Figure S103. ^{13}C NMR spectrum (CD_3OD) of compound **P-ARose**.

Me-ARose

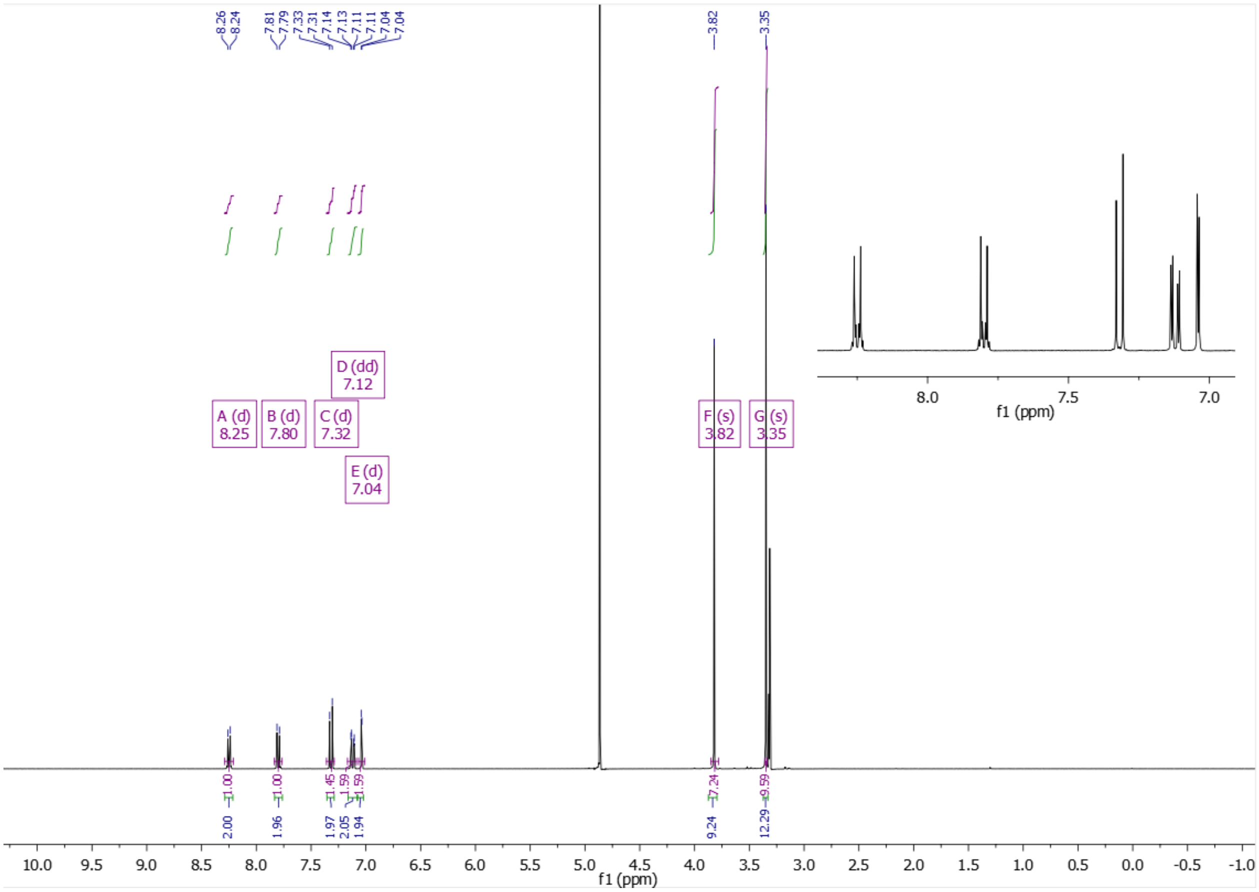

Figure S104. ^{1}H NMR spectrum (CD_3OD) of compound **Me-ARose**.

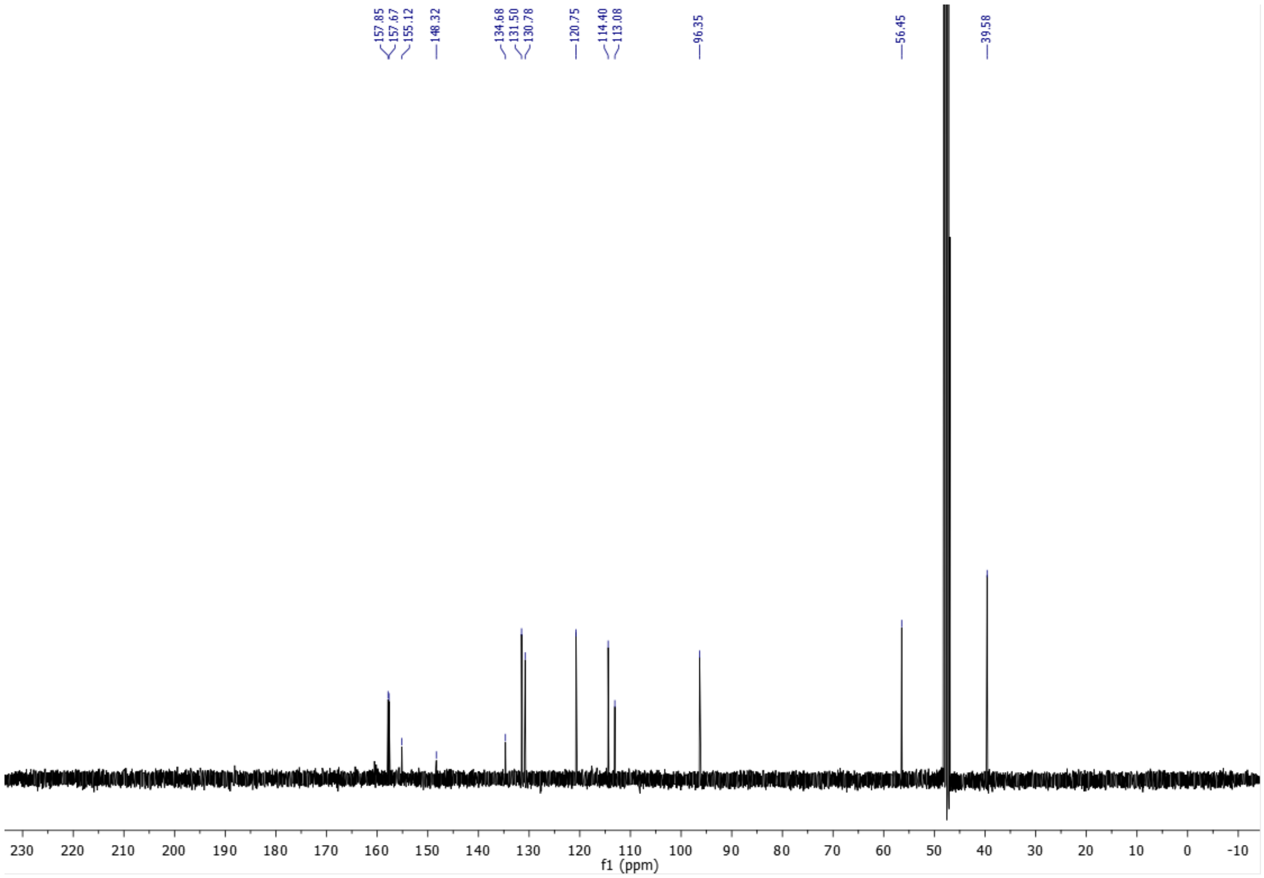

Figure S105. ^{13}C NMR spectrum (CD_3OD) of compound **Me-ARose**.

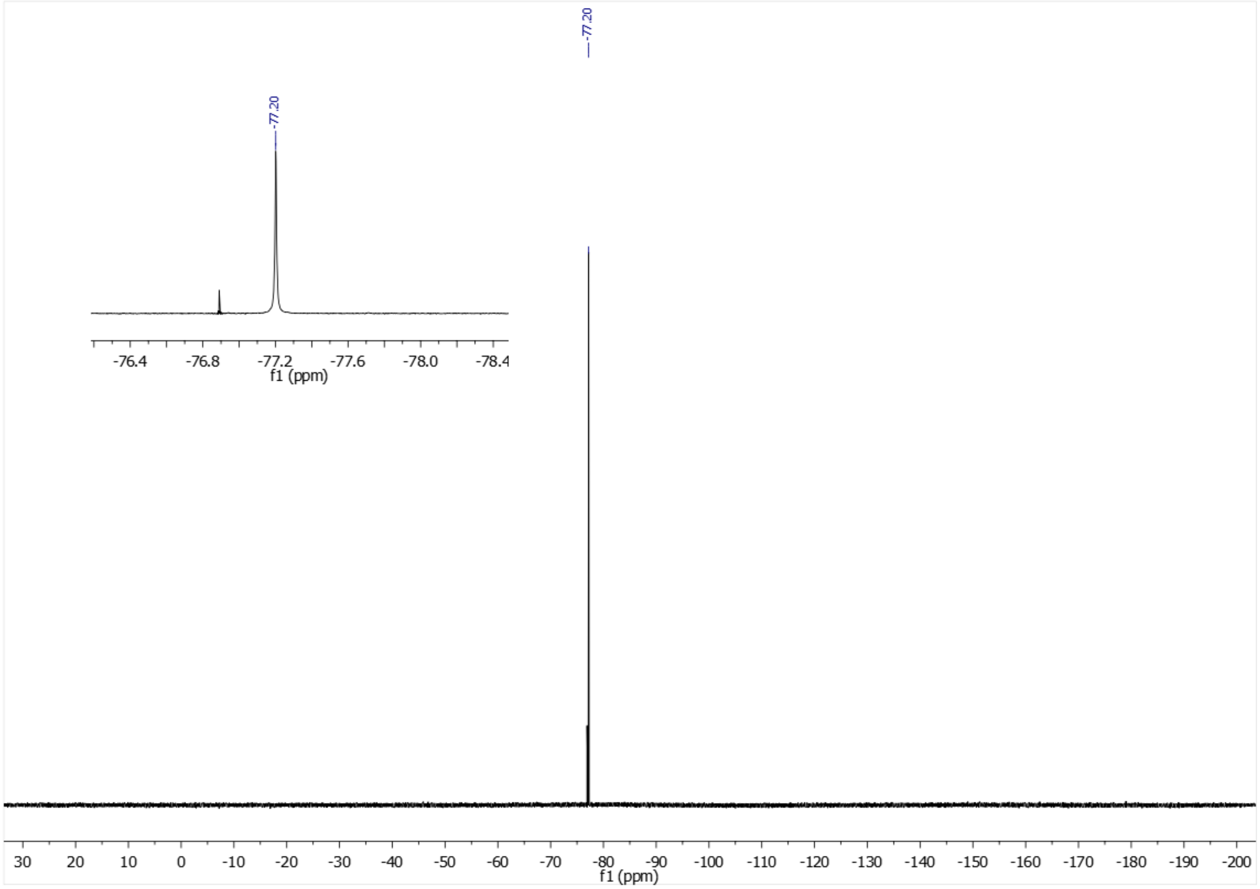

Figure S106. ^{19}F NMR spectrum (CD_3OD) of compound **Me-ARose**.

MeP-ARose

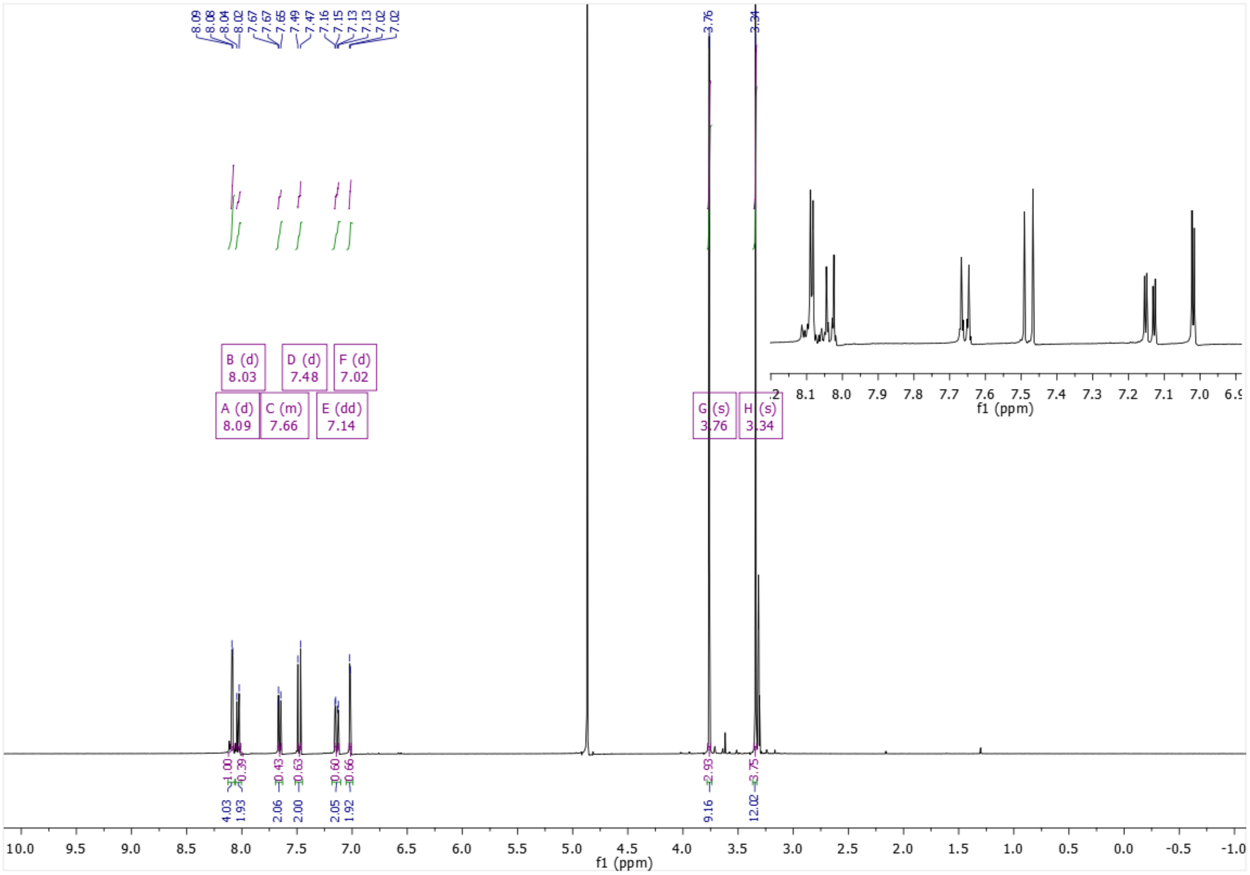

Figure S107. ^{1}H NMR spectrum (CD_3OD) of compound **MeP-ARose**.

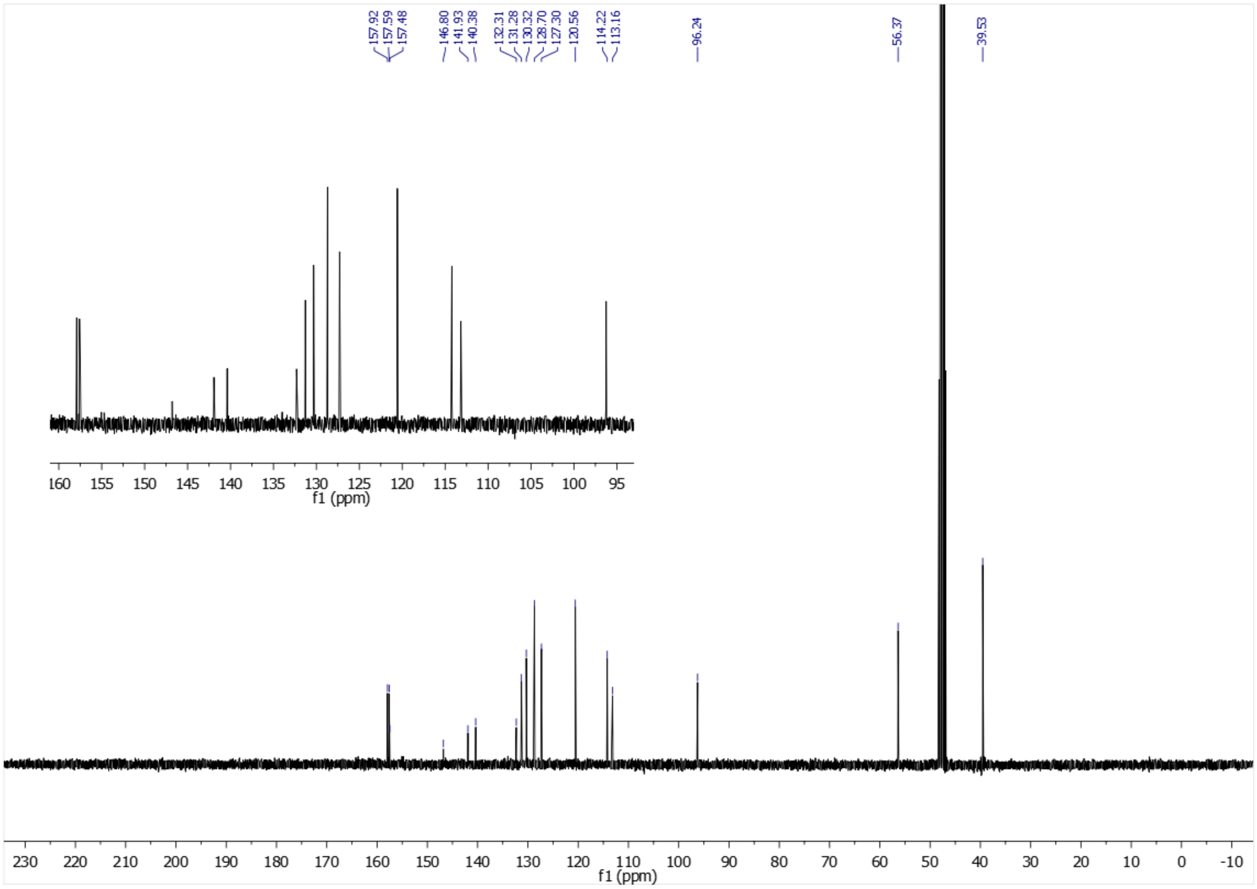

Figure S108. ^{13}C NMR spectrum (CD_3OD) of compound **MeP-ARose**.

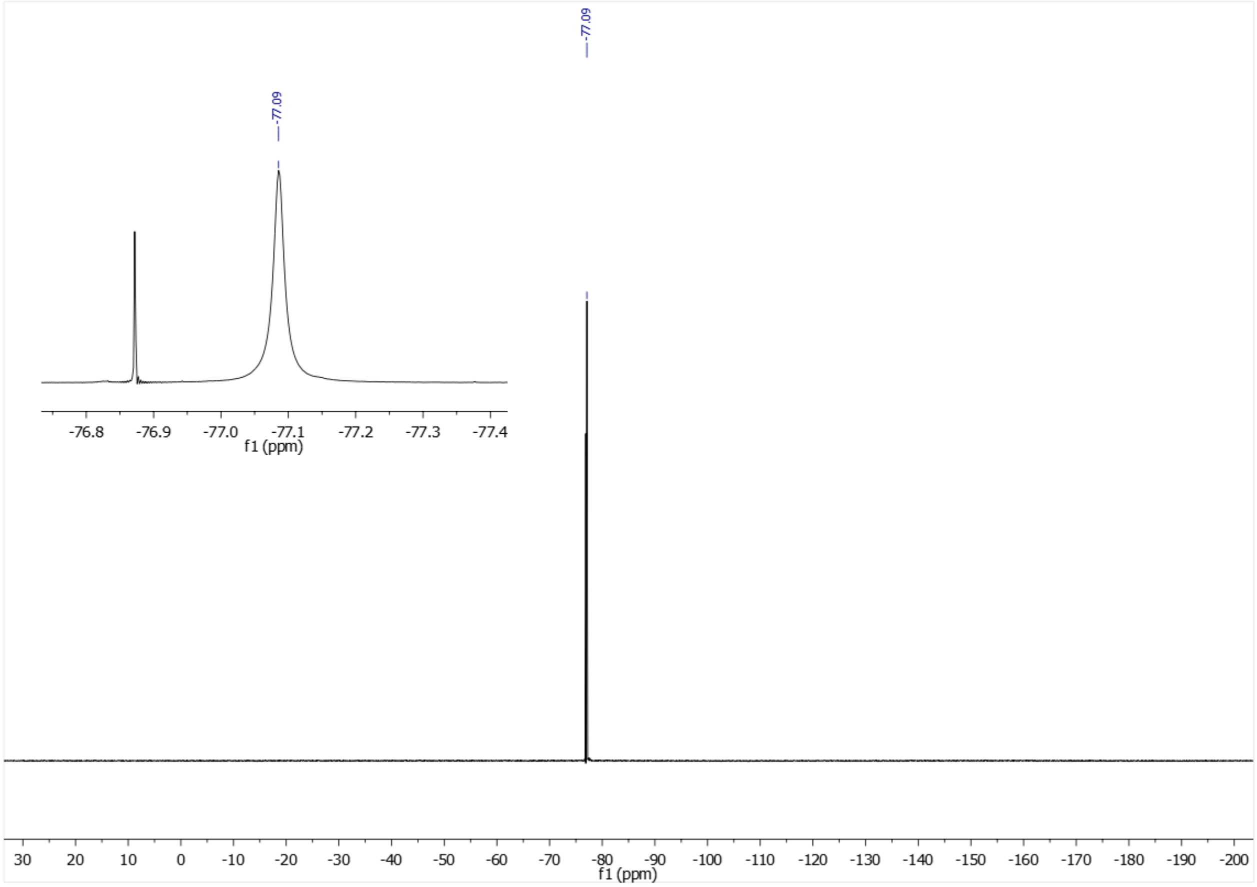

Figure S109. ^{19}F NMR spectrum (CD_3OD) of compound **MeP-ARose**.

Compound 5

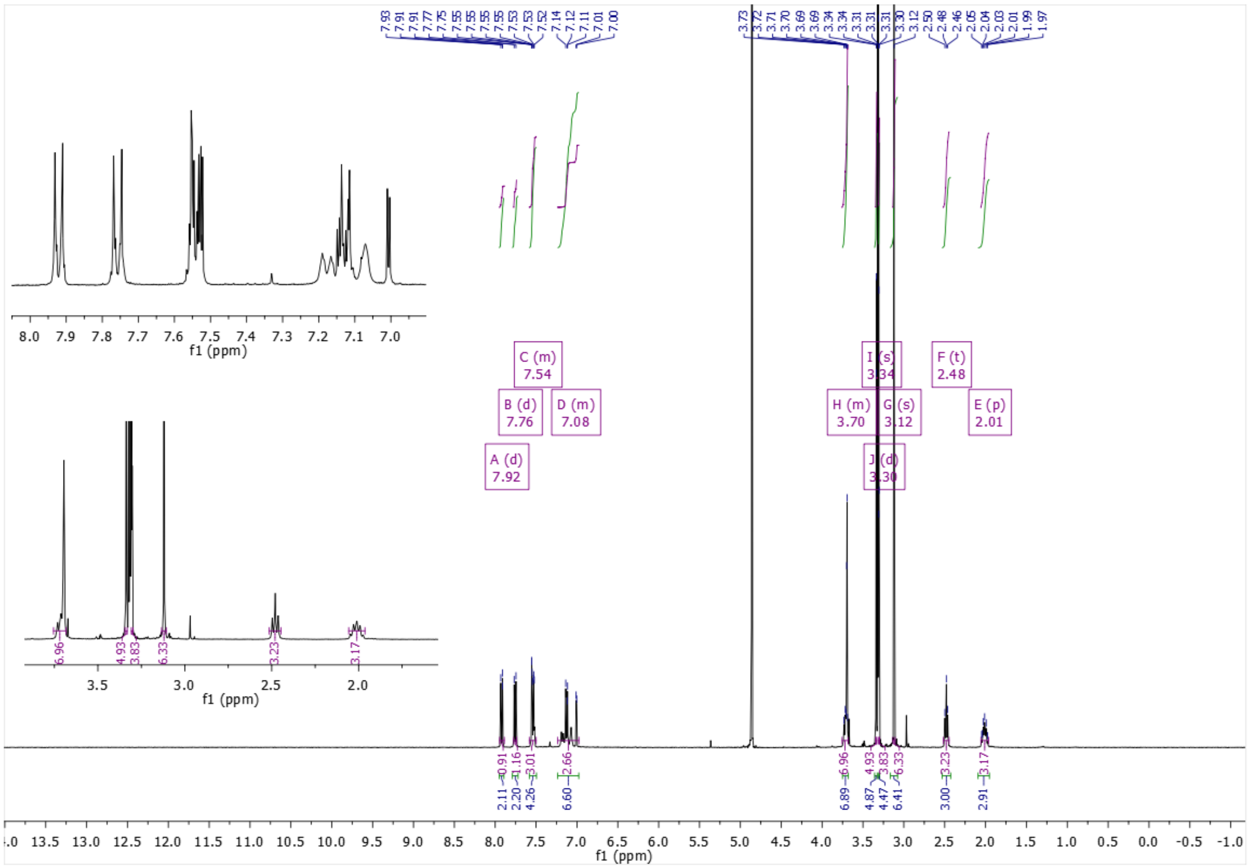

Figure S110. ^{1}H NMR spectrum (CD_3OD) of compound **5**.

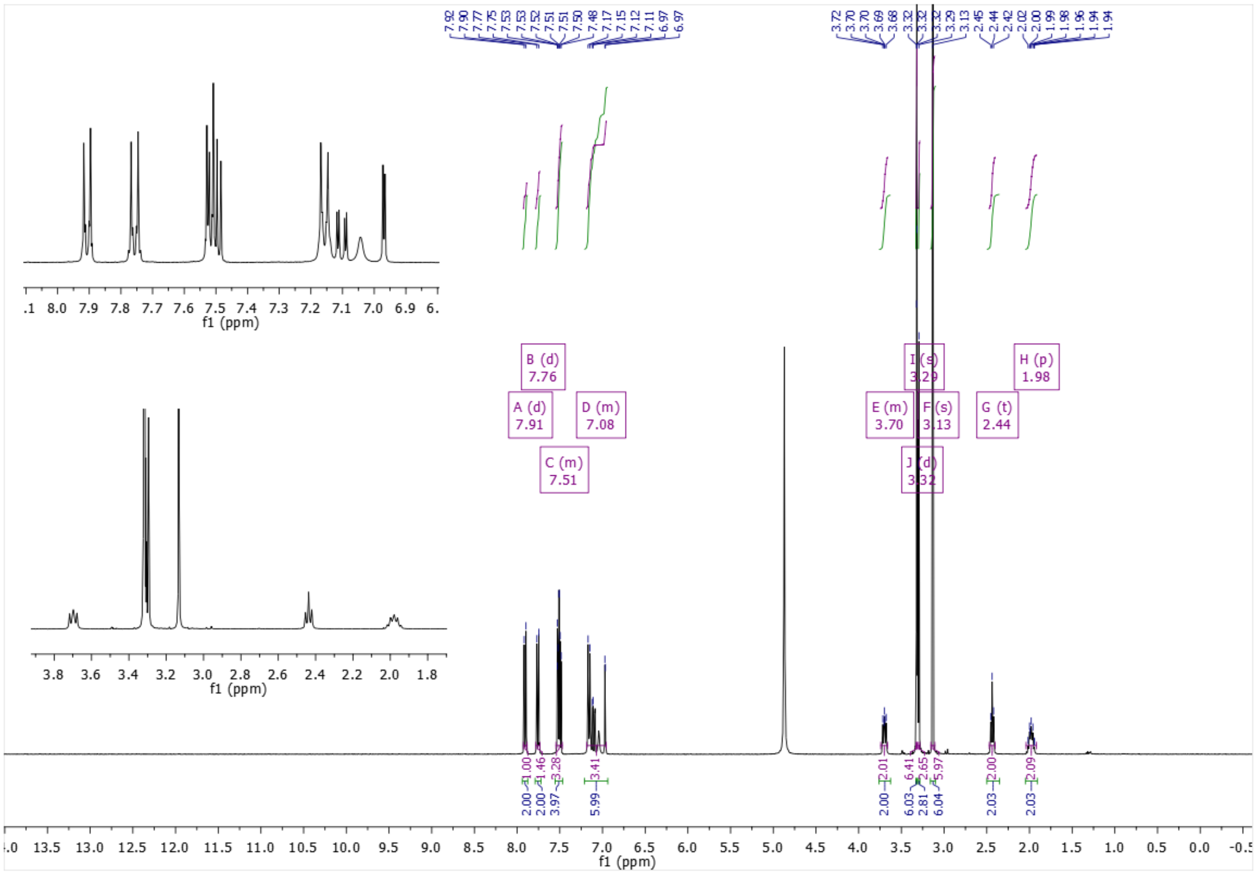

Figure S111. ^{1}H NMR spectrum (CD_3OD) of compound **5-acid**.

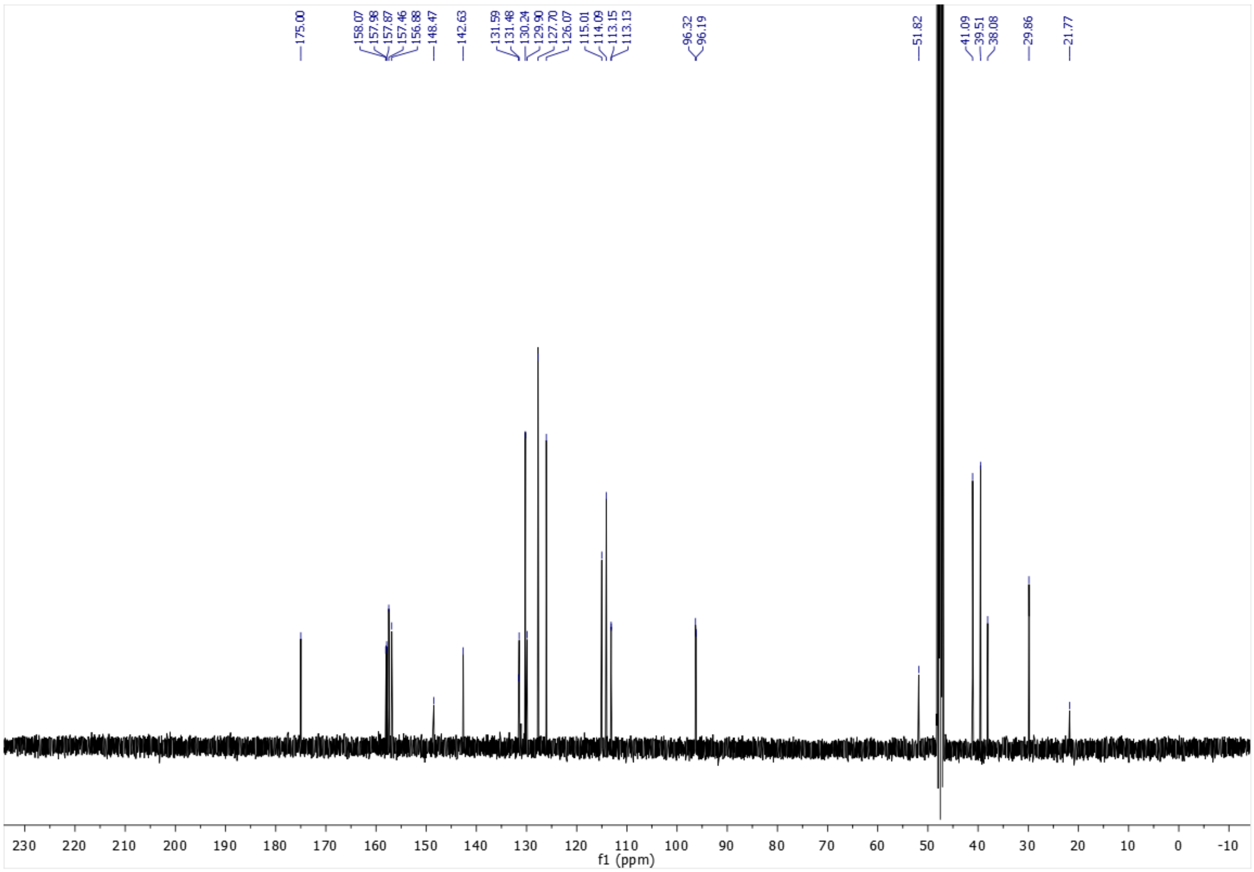

Figure S112. ^{13}C NMR spectrum (CD_3OD) of compound **5-acid**.

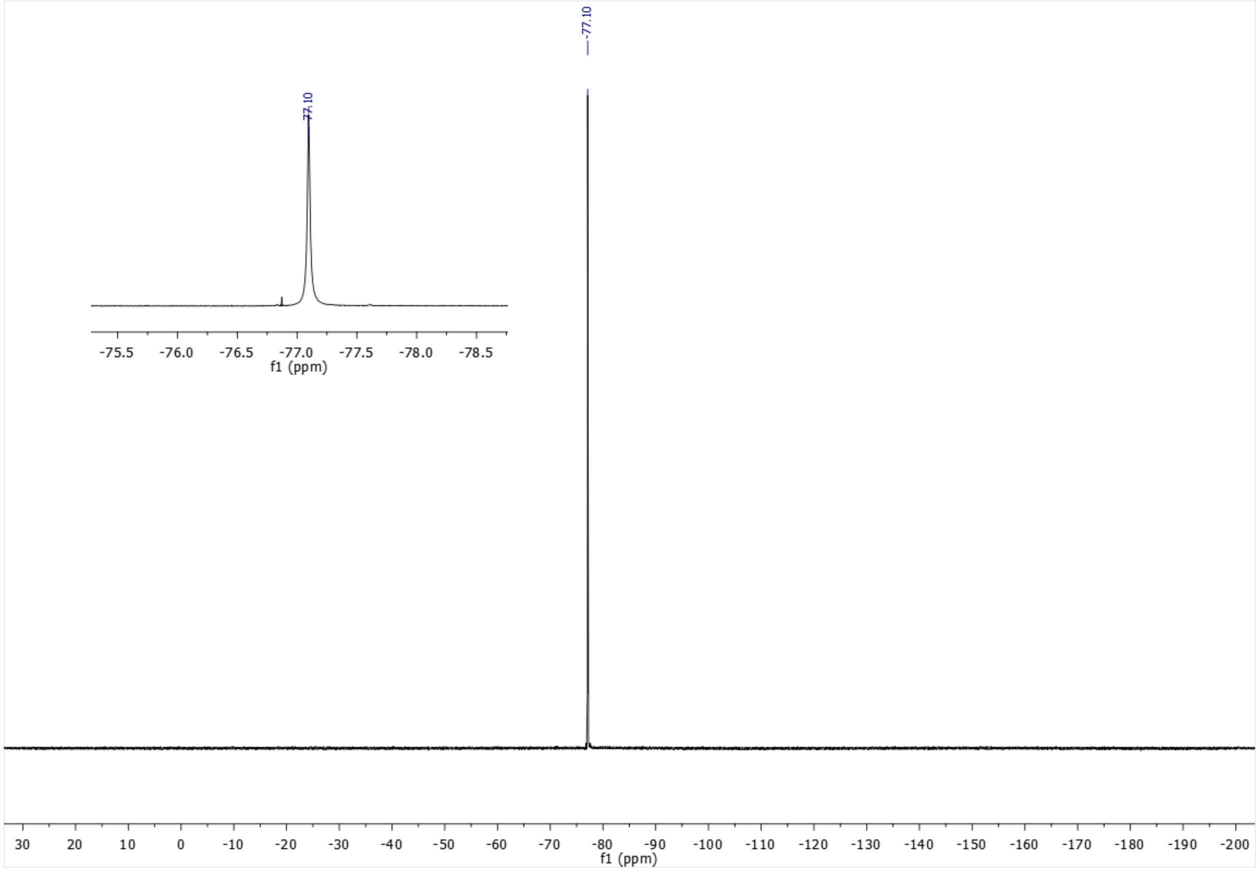

Figure S113. ^{19}F NMR spectrum (CD_3OD) of compound **5-acid**.

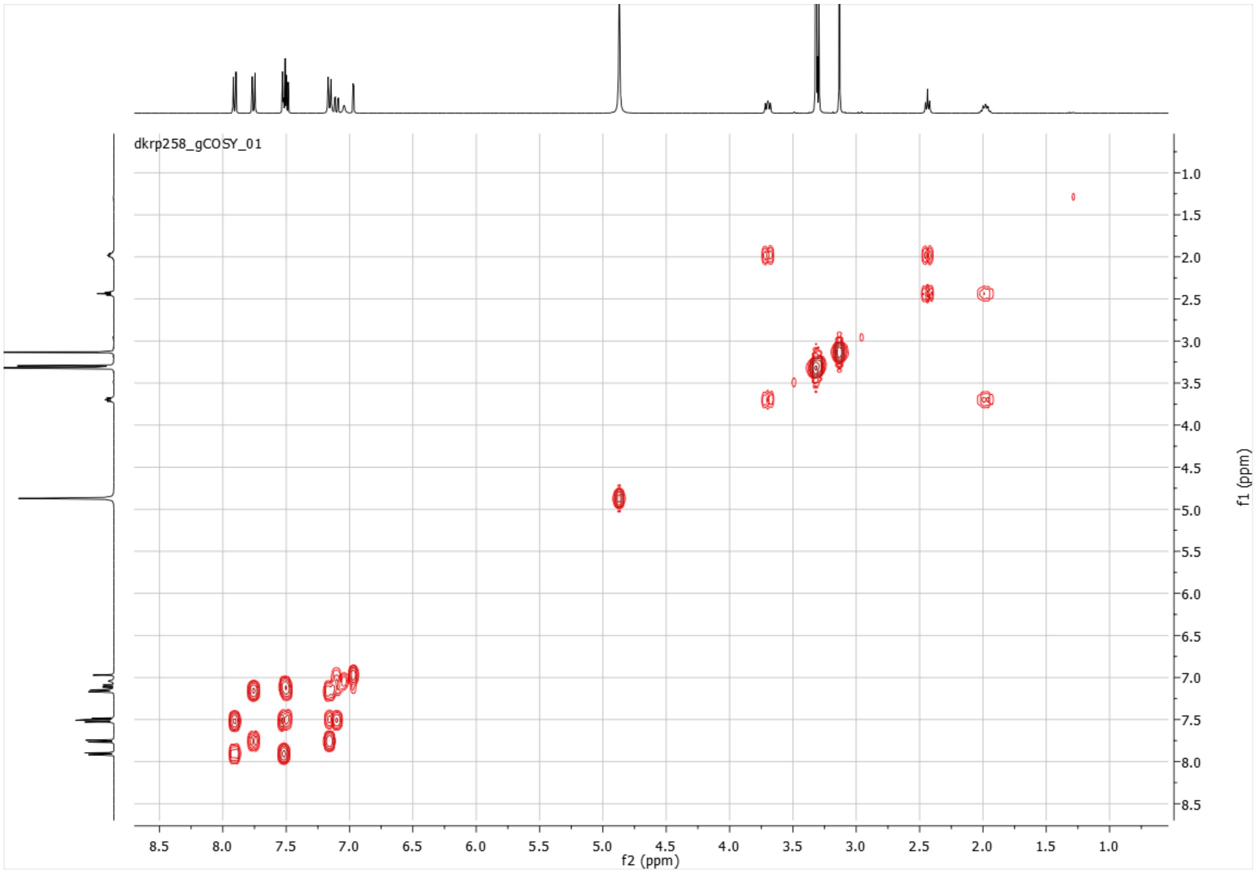

Figure S114. COSY (CD_3OD) of compound **5-acid**.

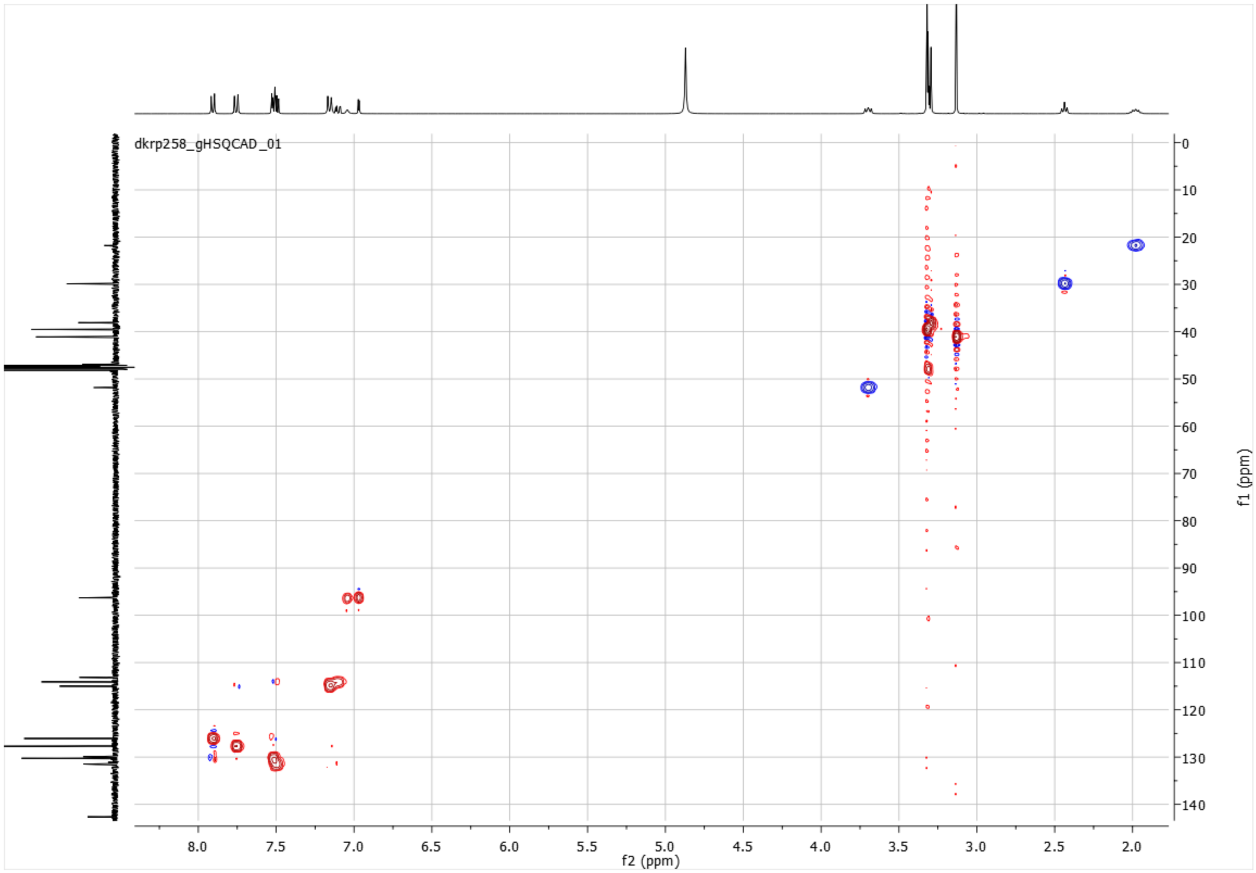

Figure S115. HSQC (CD_3OD) of compound **5-acid**.

Compound 6

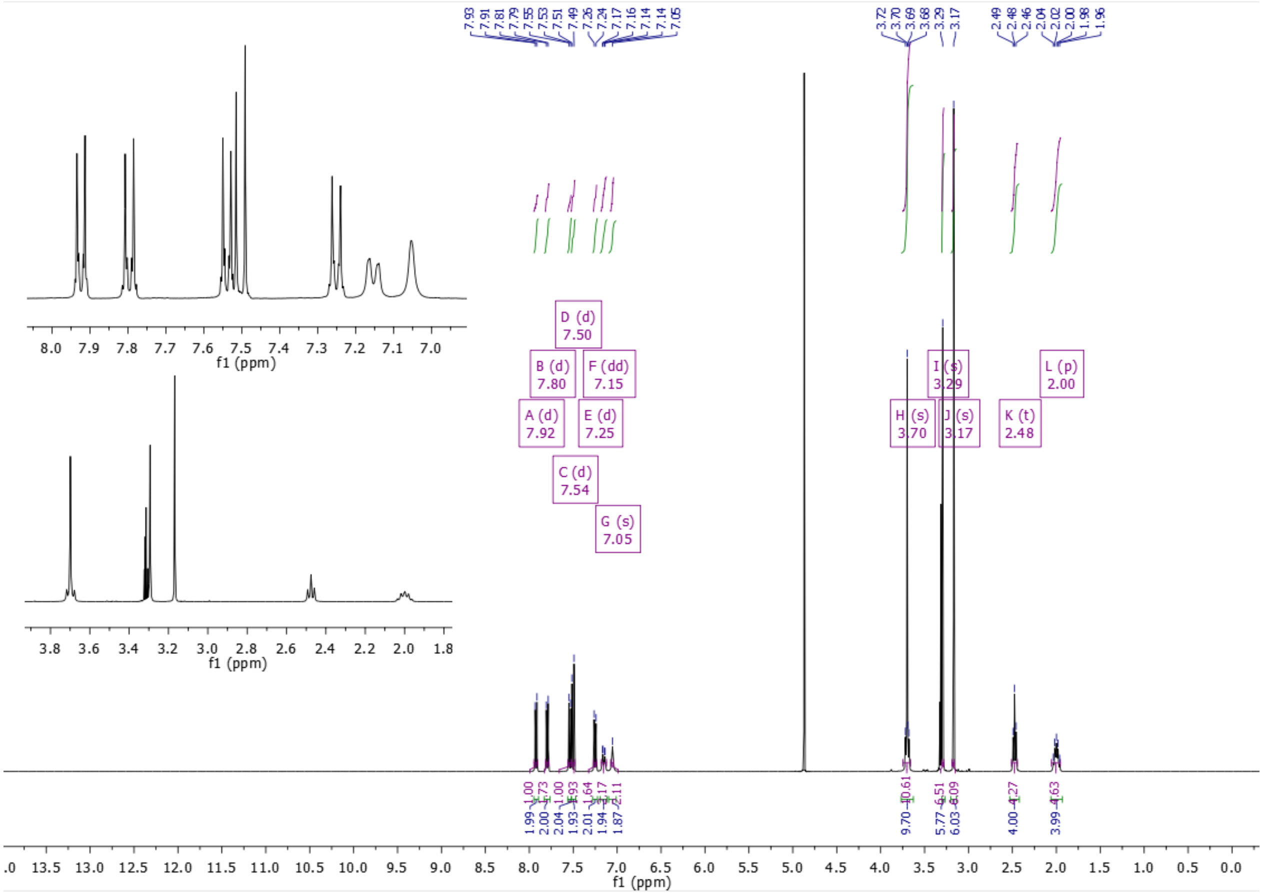

Figure S116. ^{1}H NMR spectrum (CD_3OD) of compound **6**.

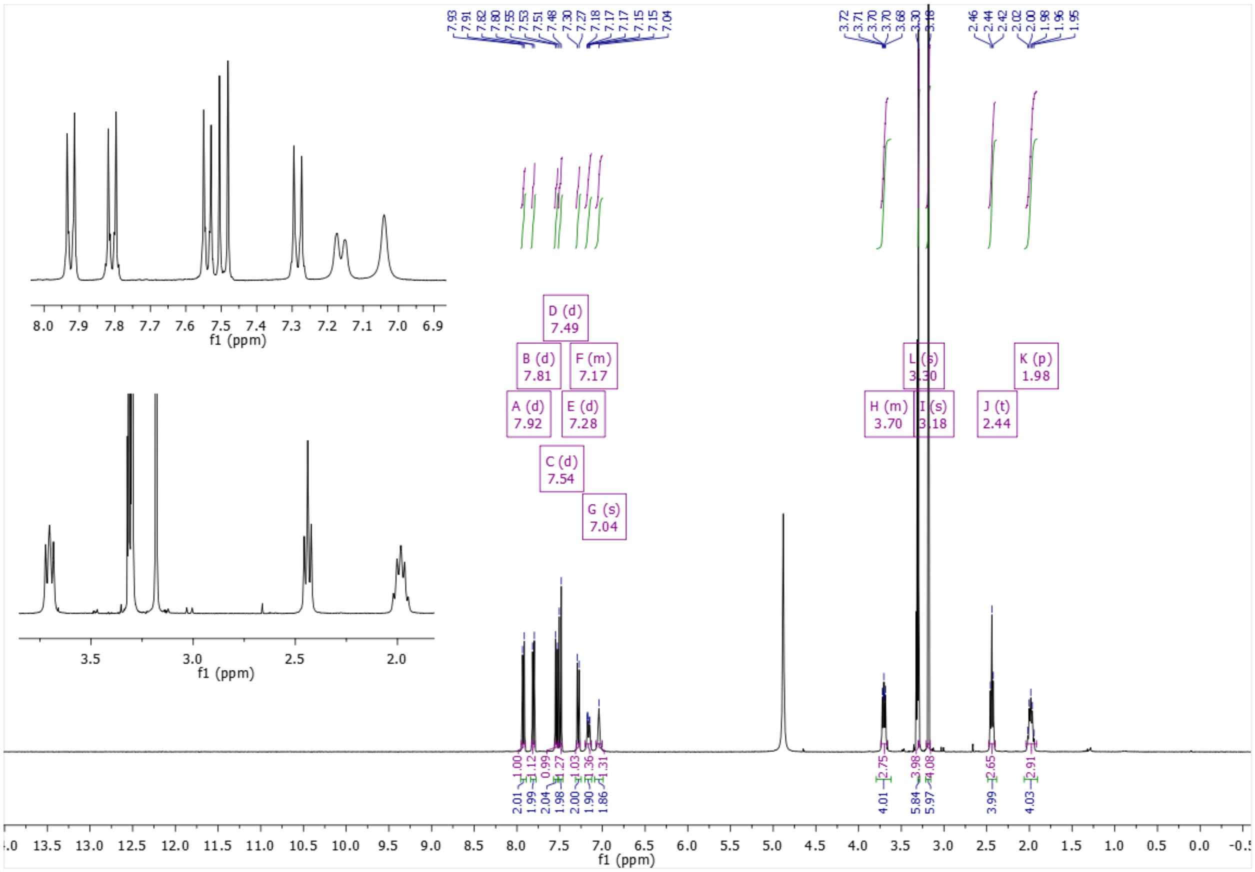

Figure S117. ^{1}H NMR spectrum (CD_3OD) of compound **6-acid**.

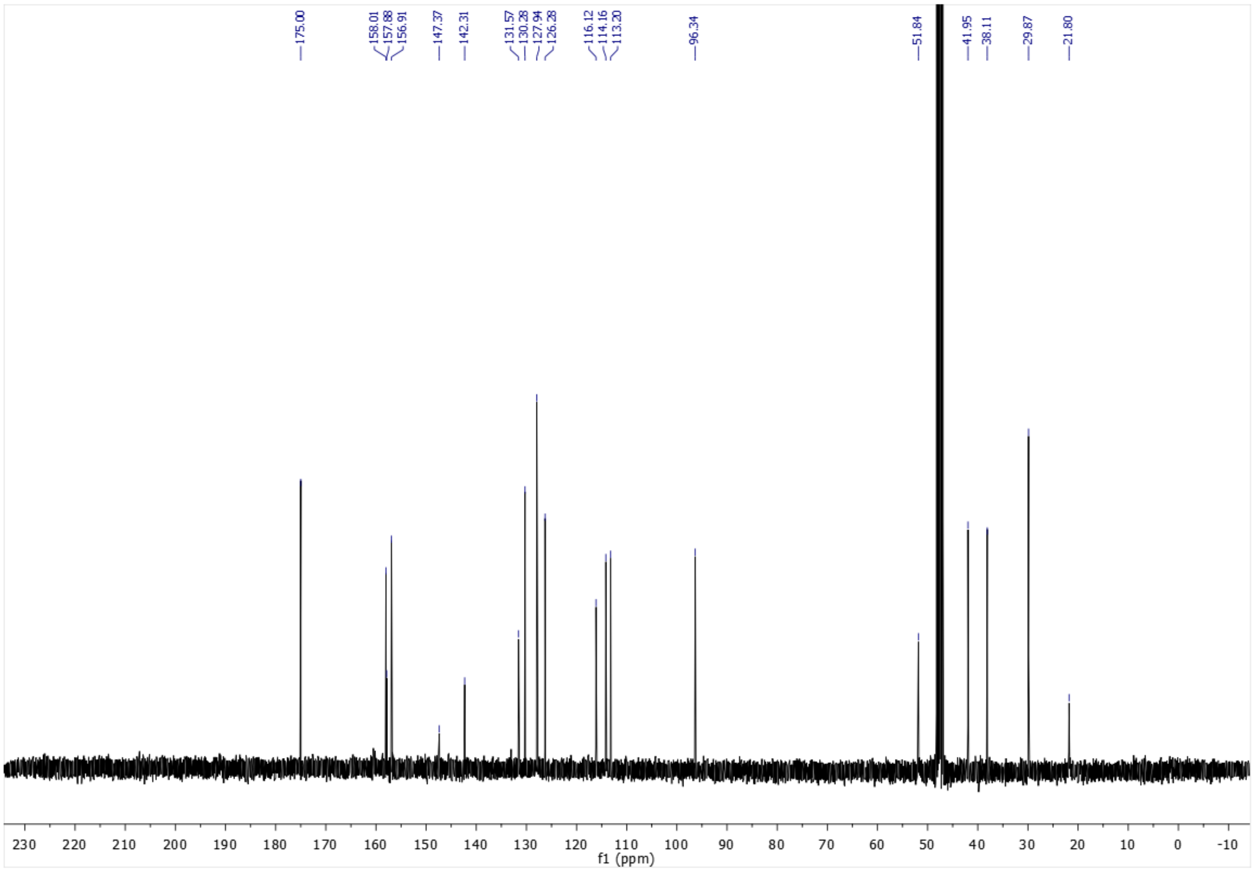

Figure S118. ^{13}C NMR spectrum (CD_3OD) of compound **6-acid**.

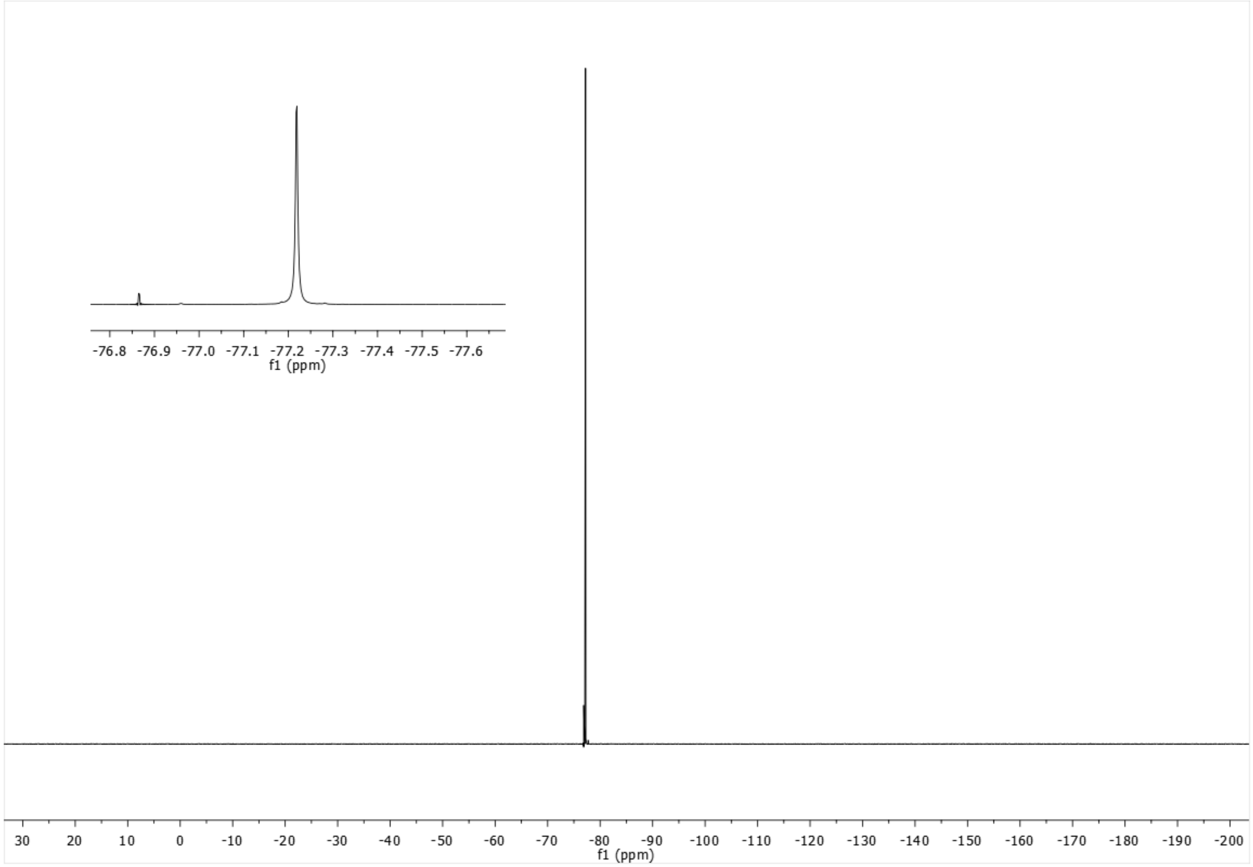

Figure S119. ^{19}F NMR spectrum (CD_3OD) of compound **6-acid**.

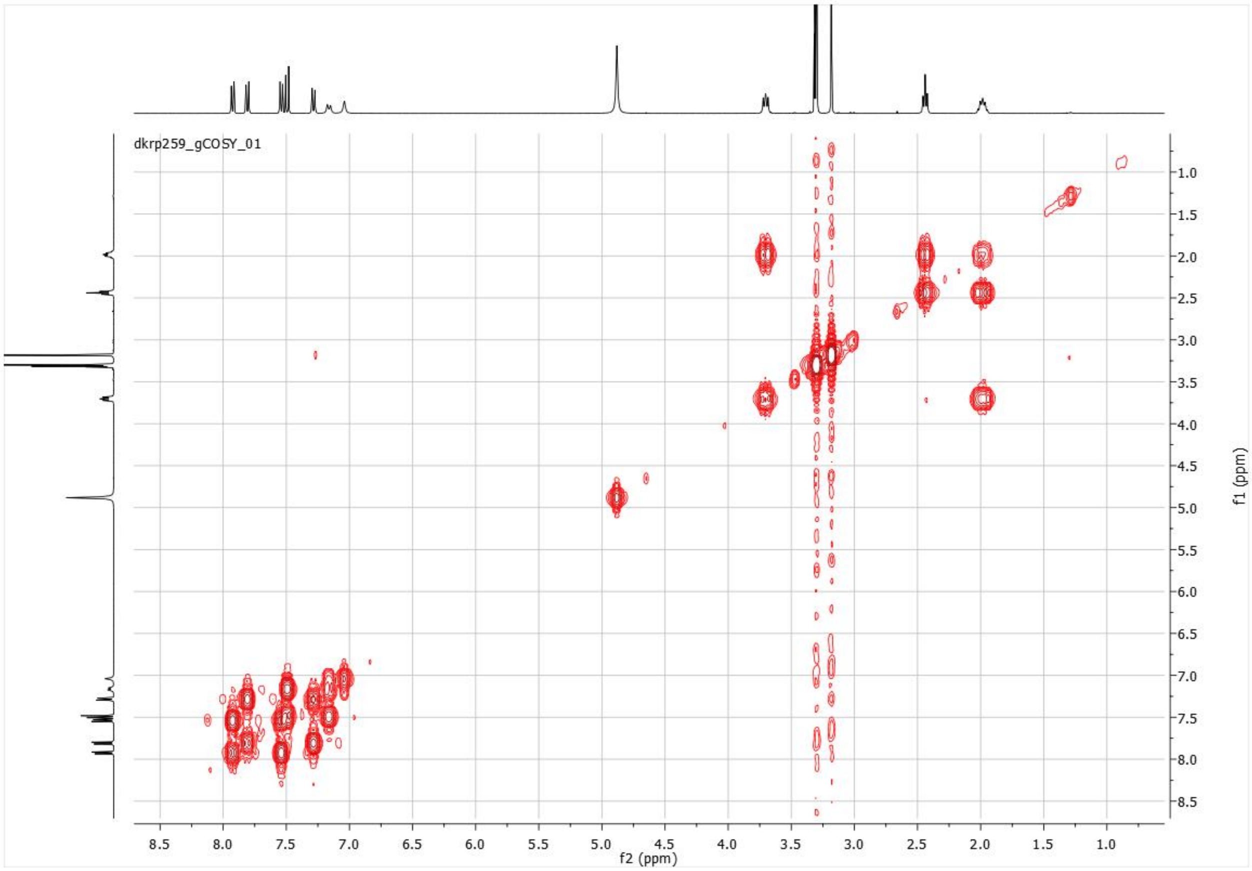

Figure S120. COSY (CD_3OD) of compound **6-acid**.

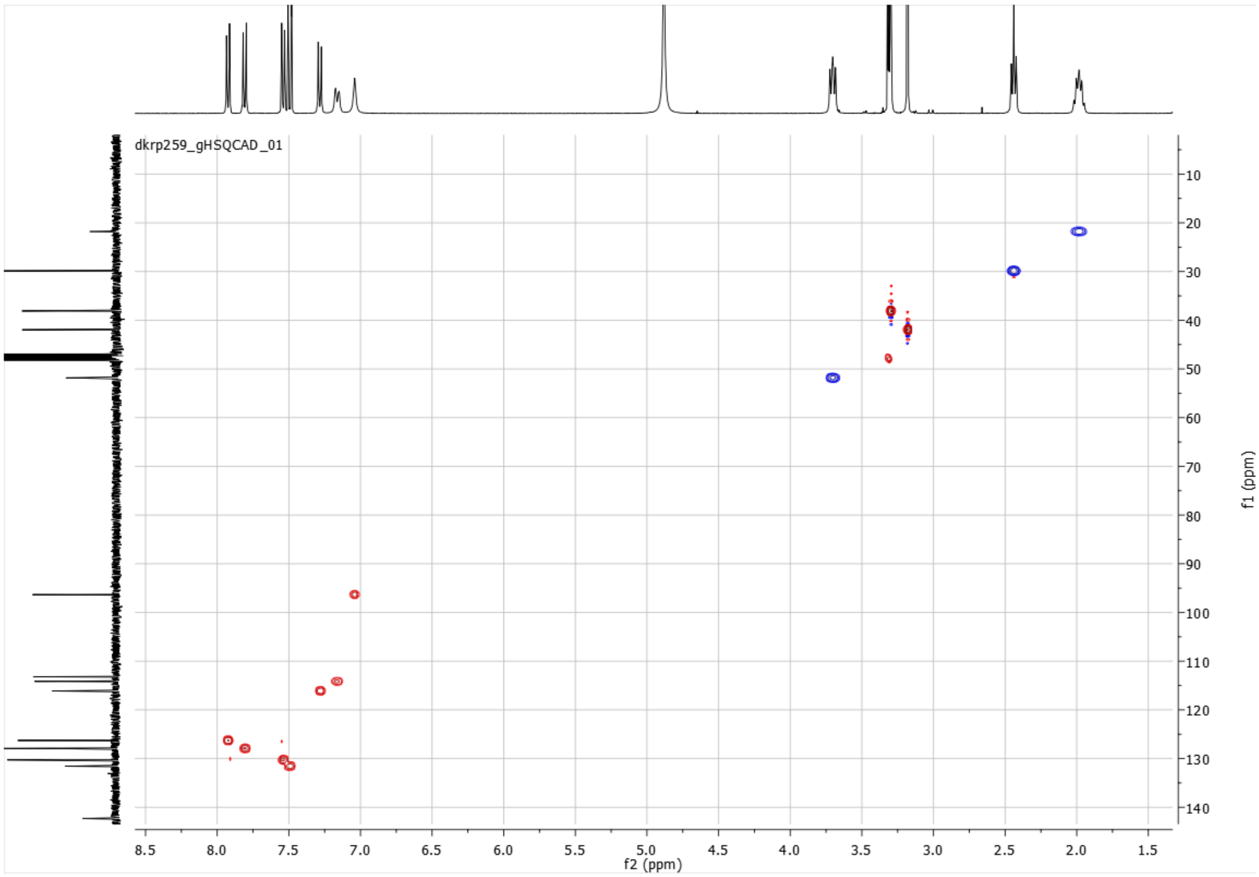

Figure S121. HSQC (CD_3OD) of compound **6-acid**.

Compound 10

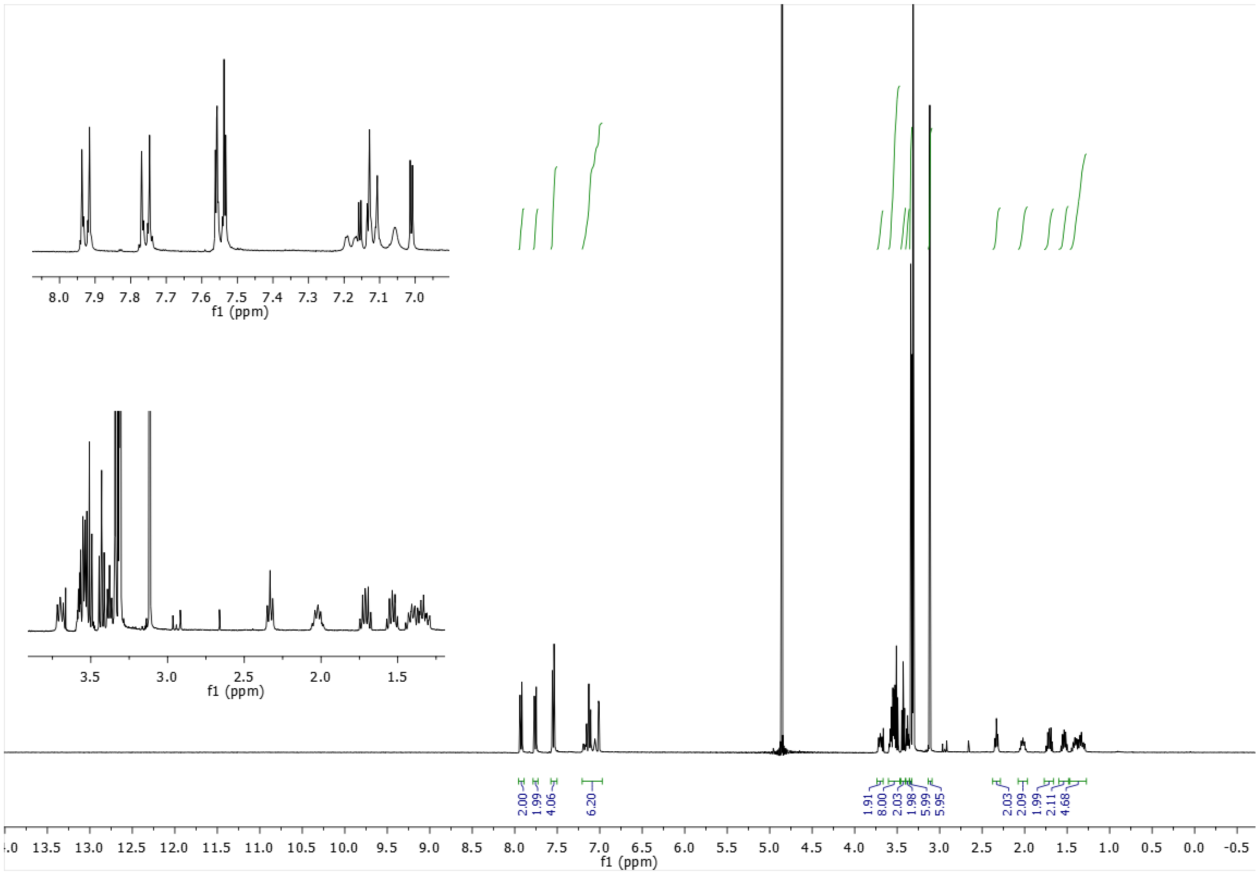

Figure S122. ^{1}H NMR spectrum (CD_3OD) of compound **10**.

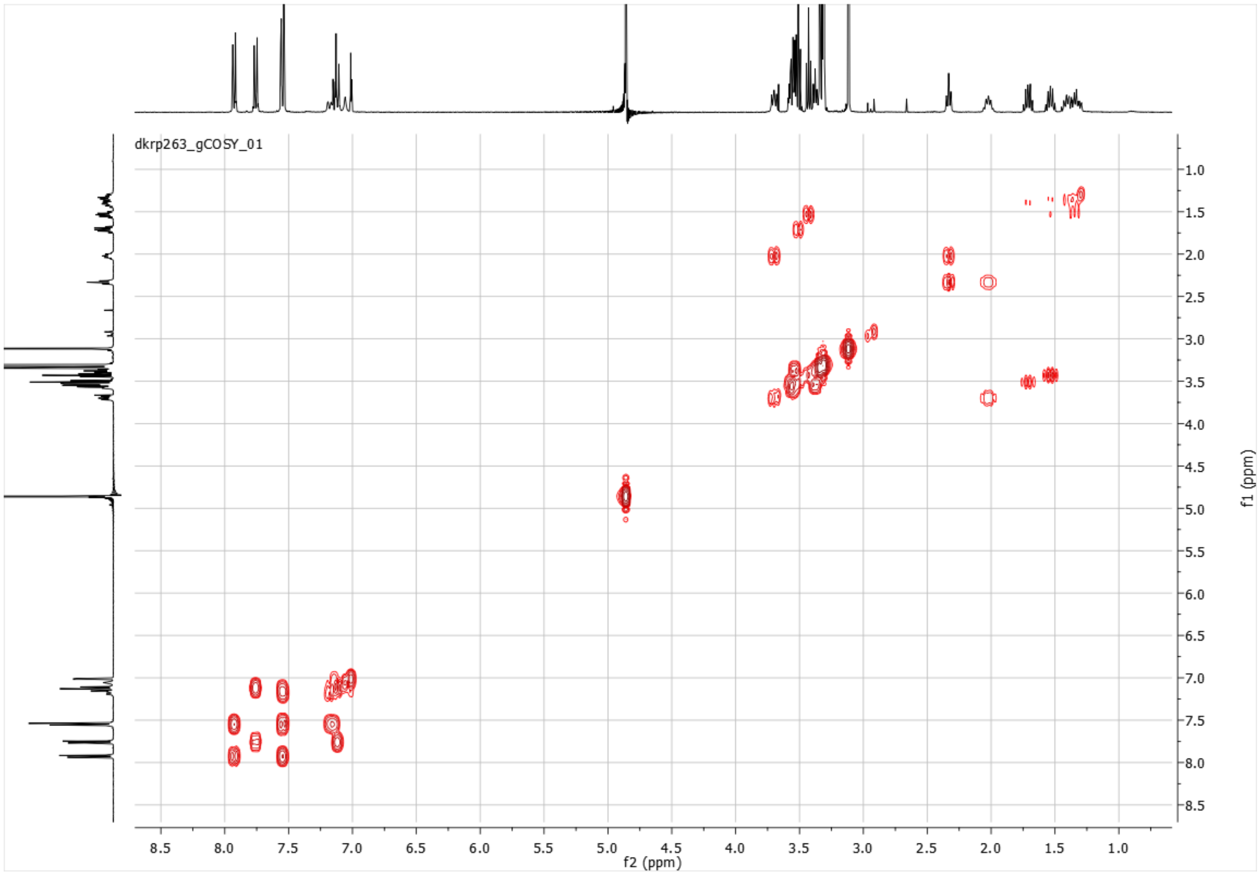

Figure S123. COSY (CD_3OD) of compound **10**.

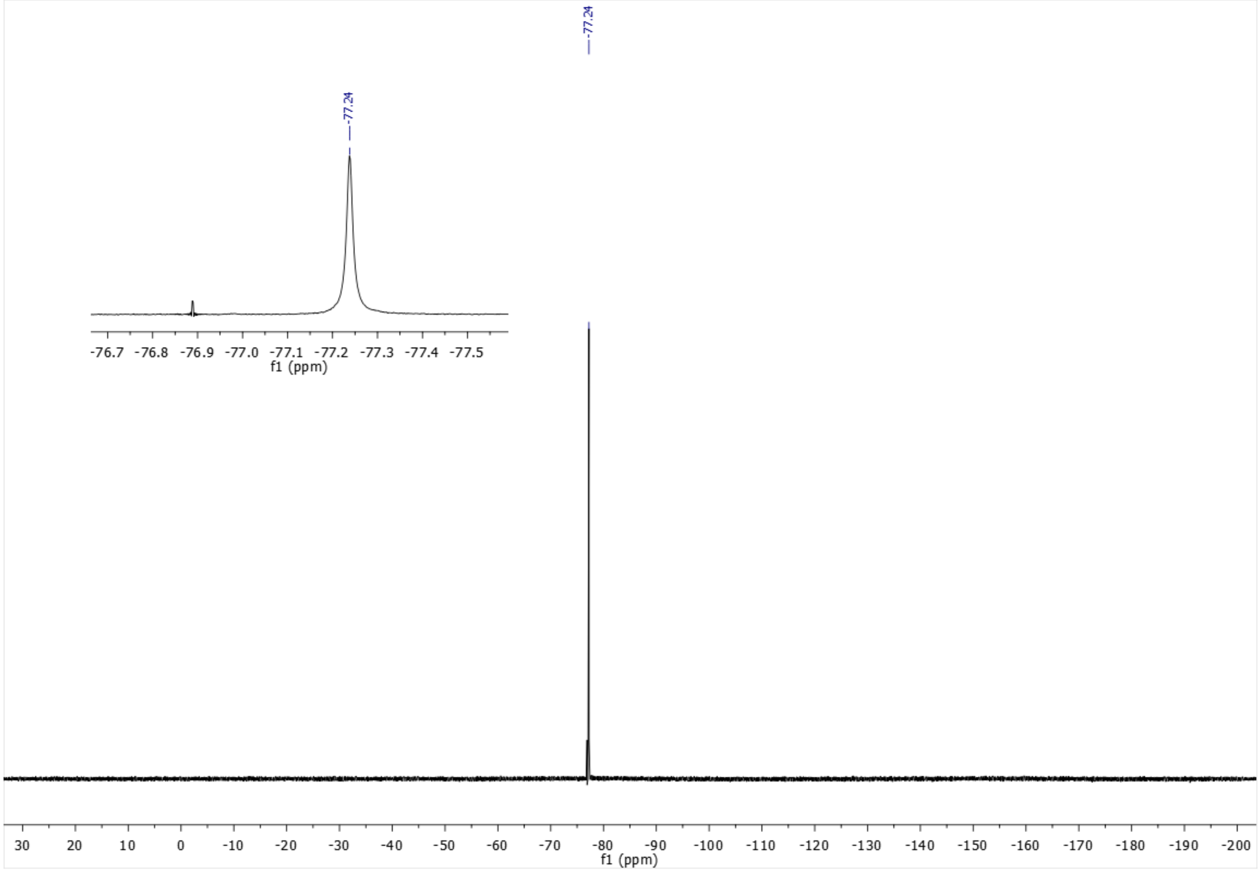

Figure S124. ^{19}F NMR spectrum (CD_3OD) of compound **10**.

Compound 11

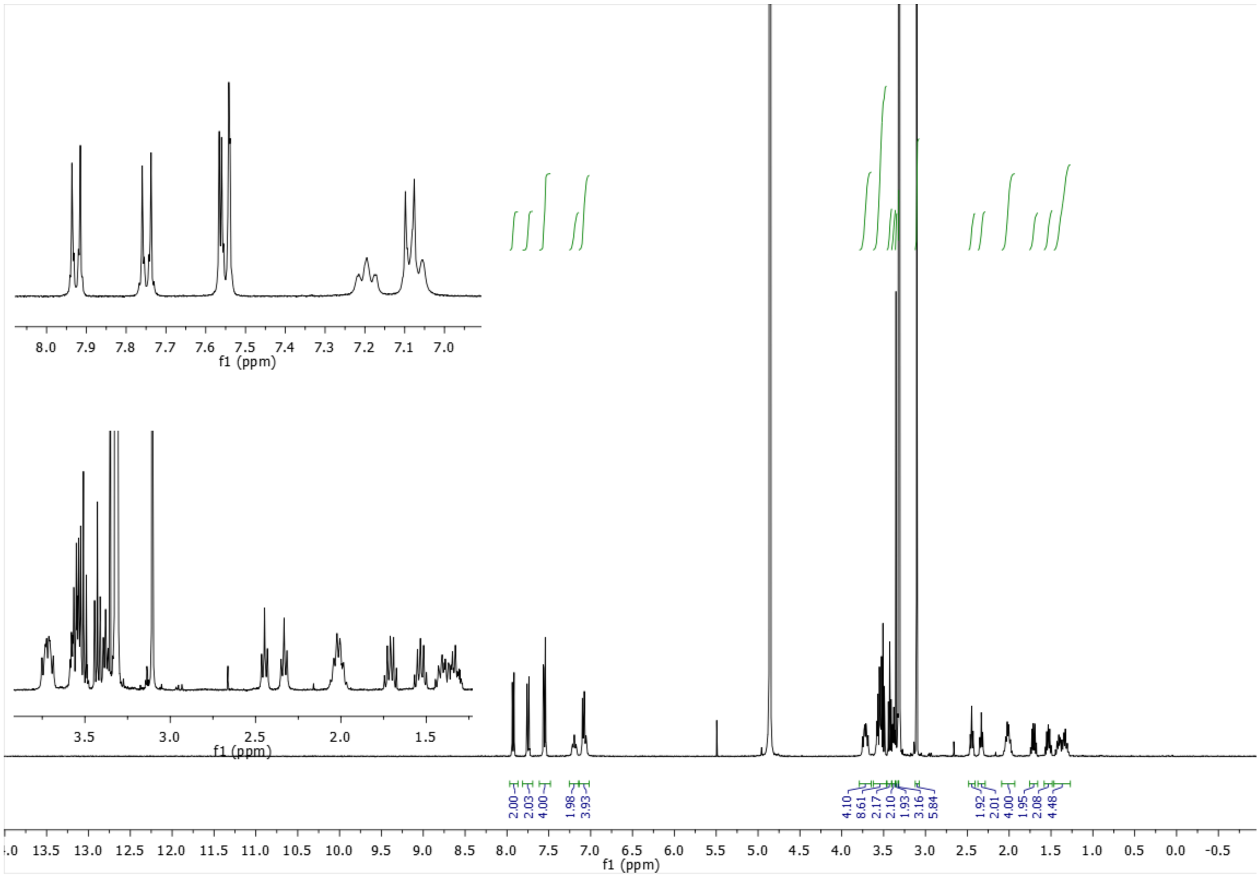

Figure S125. ^{1}H NMR spectrum (CD_3OD) of compound **11**.

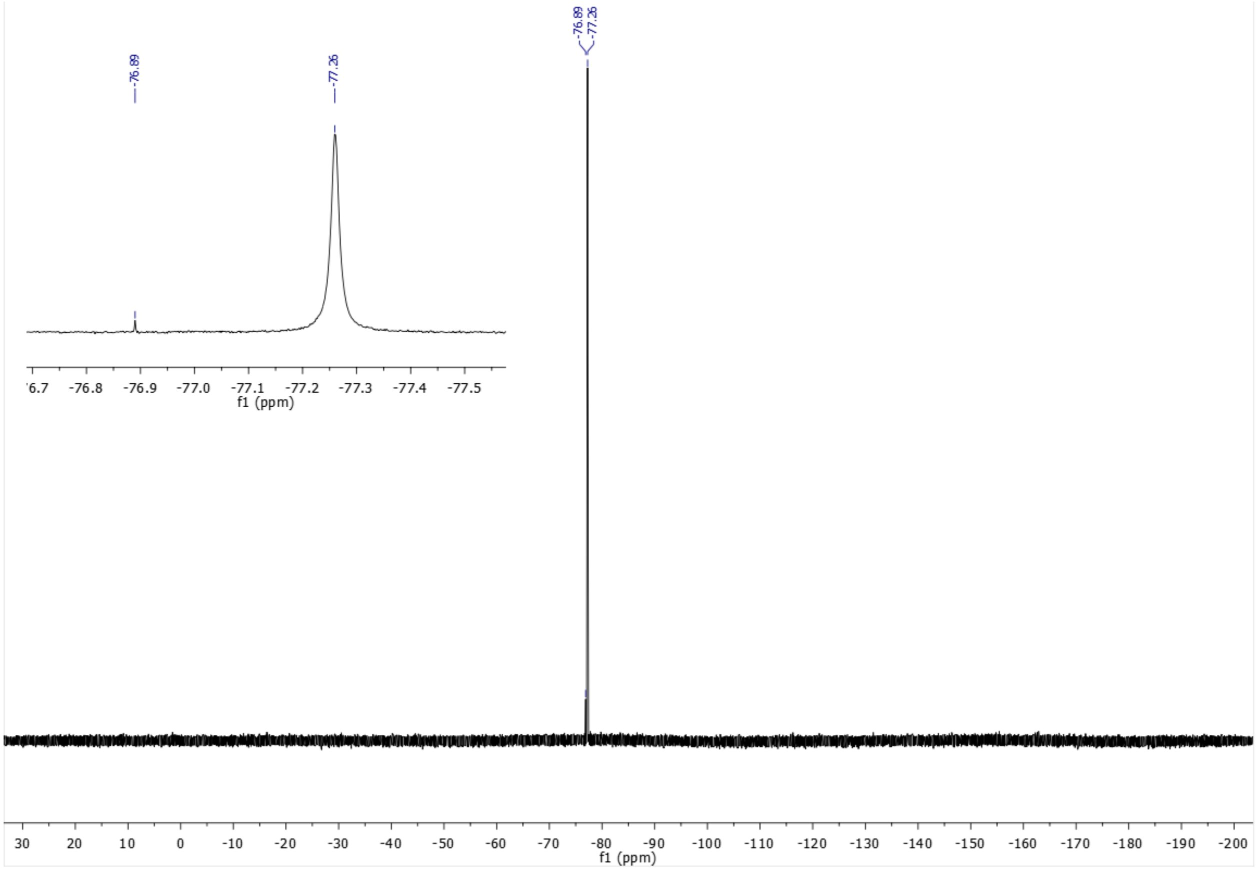

Figure S126. ^{19}F NMR spectrum (CD_3OD) of compound **11**.

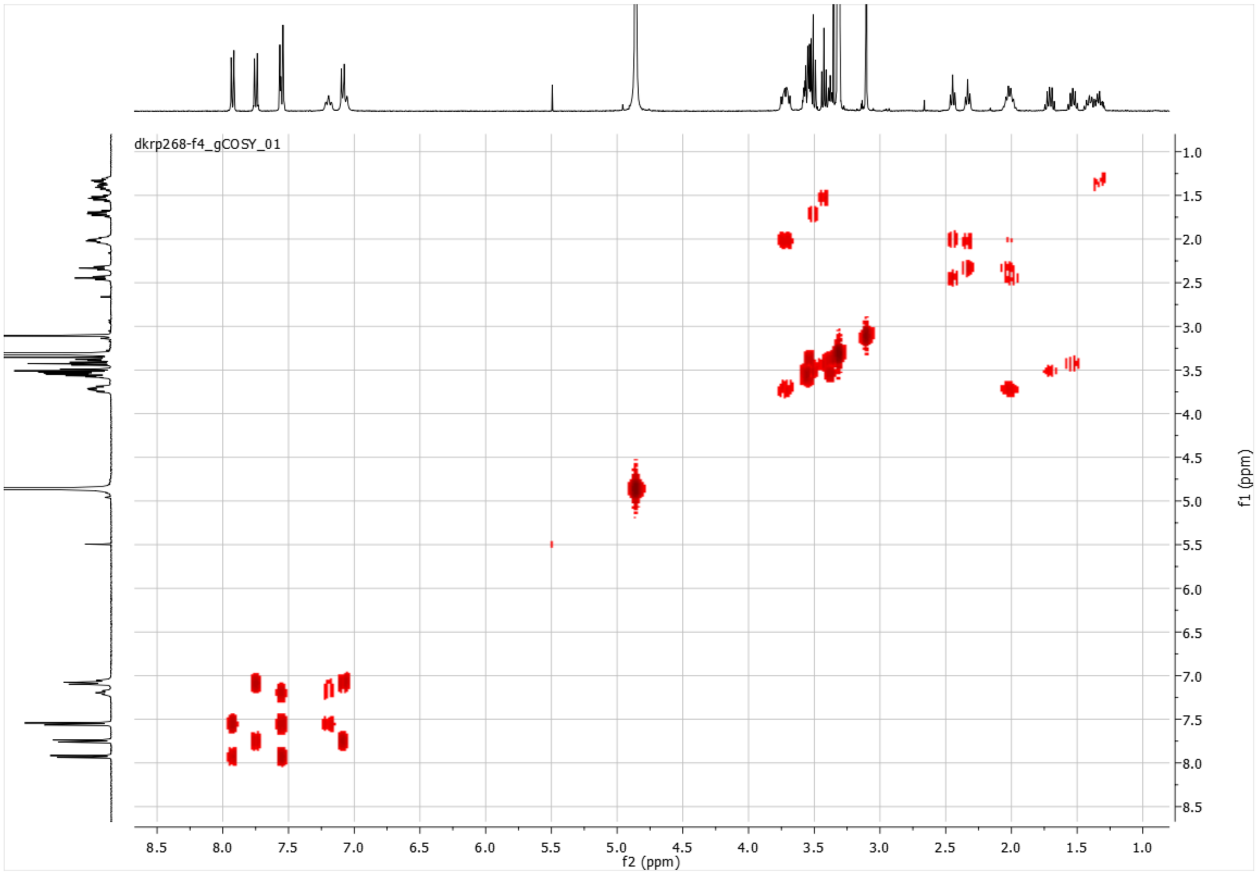

Figure S127. COSY (CD_3OD) of compound **11**.

Compound 12

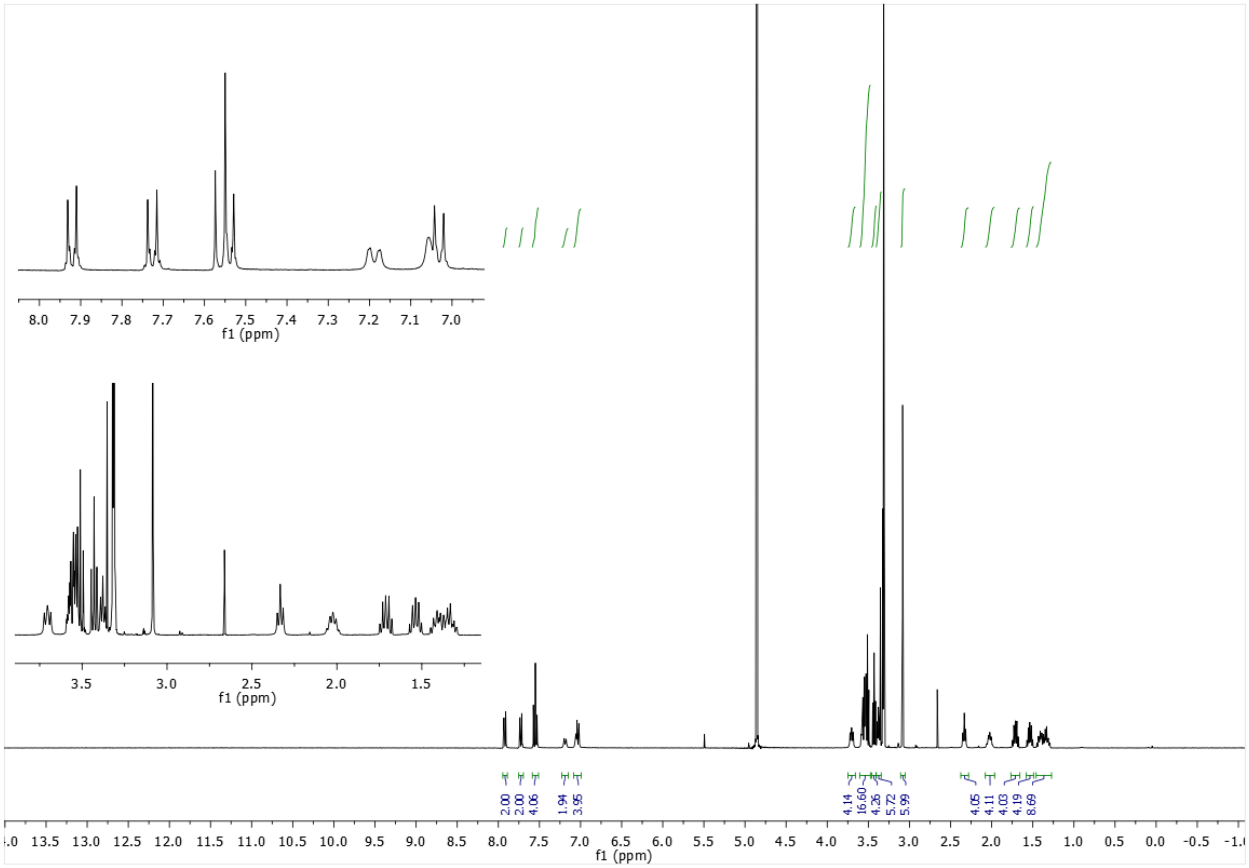

Figure S128. ^{1}H NMR spectrum (CD_3OD) of compound **12**.

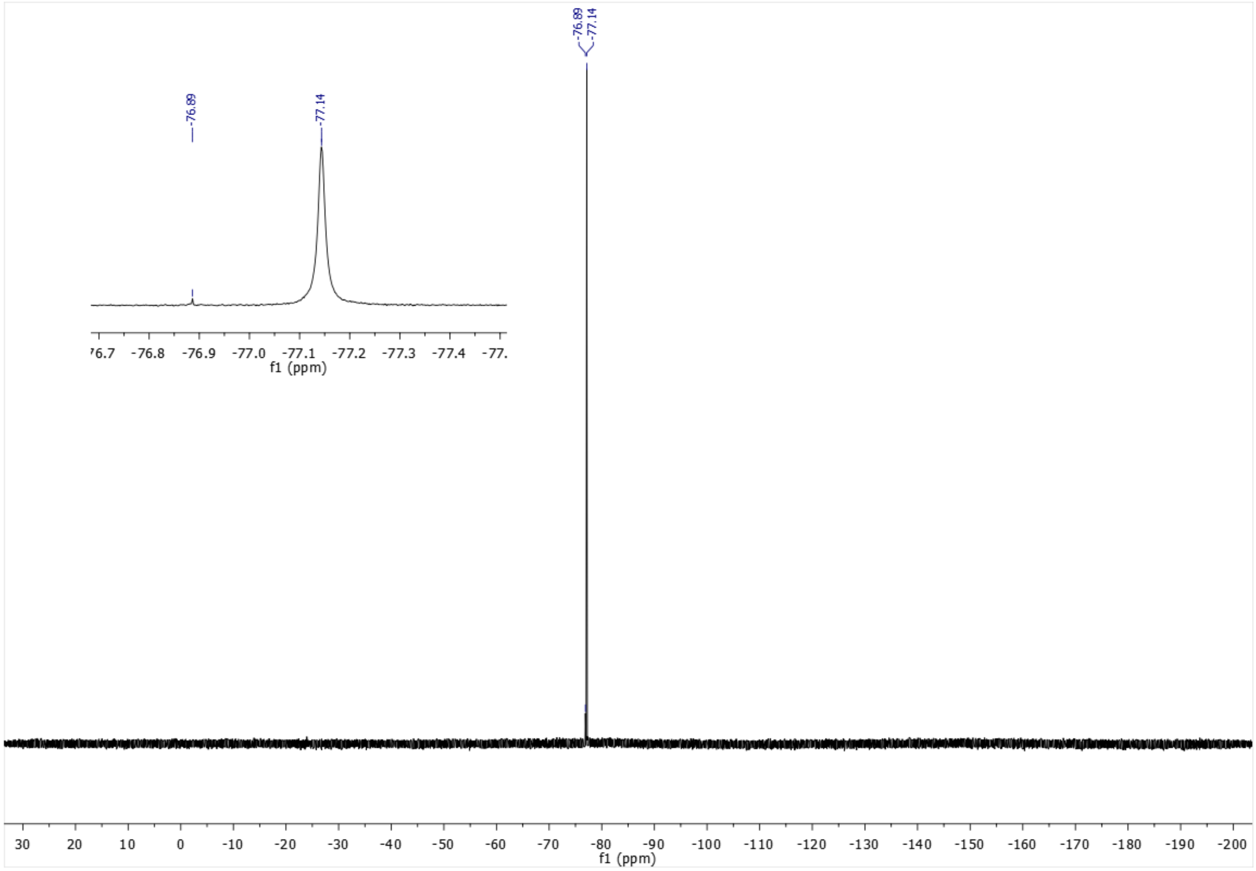

Figure S129. ^{19}F NMR spectrum (CD_3OD) of compound **12**.

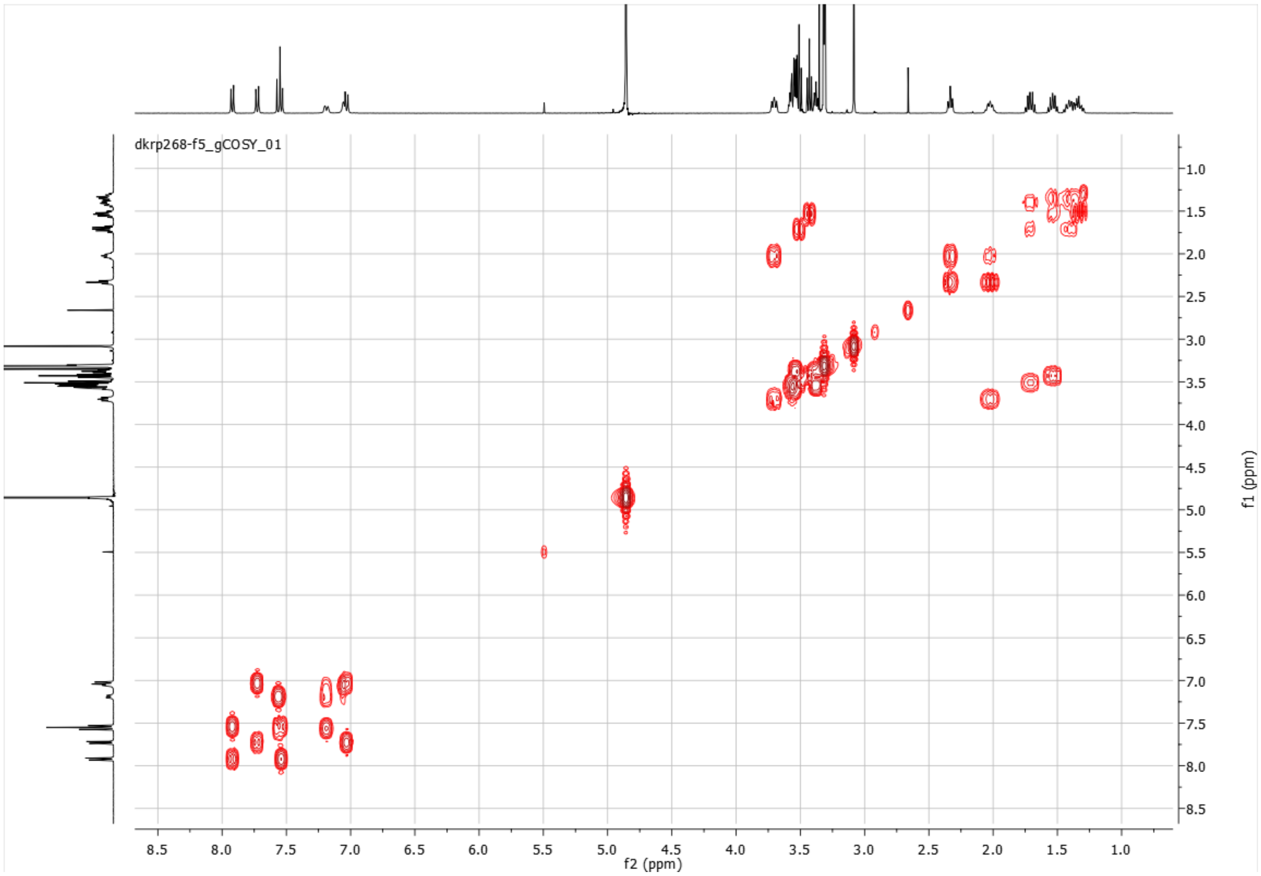

Figure S130. COSY (CD_3OD) of compound **12**.

References

[1] https://gaussian.com/citation/

[2] Sørensen, T. J.; Kilså, K.; Laursen, B. W. Aminorhodamine (ARh): A Bichromophore with Three Emission Bands in Low Temperature Glasses. *Chem.–Eur. J.* **2015**, *21*, 8521–8529.

[3] R. Gerasimaitė, J. Bucevičius, K. A. Kiszka, S. Schnorrenberg, G. Kostiuk, T. Koenen, G. Lukinavičius, *ACS Chem. Bio.l* **2021**, *16*, 2130–2136.

[4] A. N. Butkevich, M. L. Bossi, G. Lukinavičius, S. W. Hell, *J. Am. Chem. Soc.* **2019**, *141*, 981–989.
